# Prediction of Conserved HLA Class I and Class II Epitopes from SARS-CoV-2 Licensed Vaccines Supports T-Cell Cross-Protection against SARS-CoV-1

**DOI:** 10.3390/biomedicines10071622

**Published:** 2022-07-07

**Authors:** Daniel López

**Affiliations:** Centro Nacional de Microbiología, Instituto de Salud Carlos III, 28220 Majadahonda, Spain; dlopez@isciii.es; Tel.: +34-918-223-708

**Keywords:** HLA, vaccines, cross-reactivity, T cells, SARS-CoV-2

## Abstract

Heterologous immunity-inducing vaccines against different pathogens are necessary to deal with new pandemics. In this study, the possible impact of COVID-19 licensed formulations in the cytotoxic and the helper cellular immune responses against SARS-CoV-1 is analyzed for the 567 and 41 most abundant HLA class I and II alleles, respectively. Computational prediction showed that most of these 608 alleles, which cover >90% of the human population, contain enough conserved T-cell epitopes among SARS-CoV-1 and SARS-CoV-2 spike proteins. In addition, the vast majority of these predicted peptides were defined as epitopes recognized by CD4^+^ or CD8^+^ T lymphocytes, showing a very high correlation between the bioinformatics prediction and the experimental assays. These data suggest that both cytotoxic and helper cellular immune protection elicited by the currently licensed COVID-19 vaccines should be effective against SARS-CoV-1 infection. Lastly, this study has potential implications for public health against current and future pandemics, given that the SARS-CoV-1 vaccines in pipeline since the early 20th century could generate similarly cross-protection against COVID-19.

## 1. Introduction

SARS-CoV-2, the etiologic agent of COVID-19, has caused a devastating pandemic resulting in more than 481 million of confirmed cases and 6 million deaths worldwide to date (https://www.who.int/emergencies/diseases/novel-coronavirus-2019/situation-reports accessed on 1 May 2022). Thus, the vaccine prophylaxis against COVID-19 and future pandemics is a key issue in the current highly connected and globalized world. However, although the COVID-19 vaccines were developed in record time, saving the lives of millions of people, could prophylaxis against this pandemic have been developed faster?

Robust activation of the different components of adaptive immunity: Neutralizing antibodies, helper CD4^+^ T lymphocytes, and cytotoxic CD8^+^ T lymphocytes are key against SARS-CoV-2 natural infection and protective immune response after vaccination [1]. The specific interaction of the CD8^+^ or CD4^+^ T lymphocyte receptors with short viral peptides bound to human leukocyte antigen (HLA) class I or II molecules triggers the activity of these T cells, and also initiates, regulates, or suppresses other components of adaptive immune responses [2]. In the absence of correct HLA class I- and II-restricted T-cell recognition, both cellular and humoral immune responses are not activated efficiently and thus, the infectious virus could spread through the organism with fatal results for the host. This highly complex set of immune events can be altered, or even suppressed by single changes in the virus epitope sequences that lead to a complete loss of antigen recognition by either CD4^+^ or CD8^+^ T cells as was previous described for influenza [3], HCV [4], HIV [5], LCMV [6], SIV [5], and even in coronavirus [7] and SARS-CoV-2 [8]. This extremely low tolerance to amino acid changes in the antigen recognition can render ineffective the lymphocytes previously activated by the administration of vaccines if new variants of the virus emerge with multiple changes [9]. In this context, it is unlikely that a vaccine developed against a certain virus will be able to generate a cross-response against another related virus.

All licensed formulations against SARS-CoV-2 are based on the original D614 spike protein sequence of the Wuhan-1 wild-type strain. The main difference is the type of platform vaccine used: messenger RNA, non-replicating viral vector, inactivated SARS-CoV-2, or protein subunit. The main concern of all these vaccines is their cost and that specialized infrastructure is needed; thus, vaccines adapted to low- and lower-middle-income countries would be necessary. Moreover, SARS-CoV-2 is included in the subgenus Sarbecovirus with their highly homolog SARS-CoV-1. Spike proteins from these related viruses differ in 304 amino acids. These changes are not randomly distributed throughout the entire spike protein sequence and, thus, the possible cross-recognition of cytotoxic and/or helper immune responses by conserved epitopes among sarbecovirus remains open. In this study, I approach this aspect focusing on the HLA class I- and II-restricted epitopes conserved among SARS-CoV-1 and SARS-CoV-2 spike proteins. Although a significant loss of HLA class I- and II-restricted epitopes derived from SARS-CoV-2 vaccines was observed, a relevant number of epitopes remained conserved among the sarbecovirus spike proteins, which could generate the global cytotoxic and helper responses against SARS-CoV-1 elicited with the currently licensed vaccines. Additionally, the SARS-CoV-1 vaccines in the pipeline since the early 20th century could have generated cross-protection against SARS-CoV-2 infection.

## 2. Materials and Methods

### 2.1. Selection of Antigenic Proteins

The spike protein of the SARS-CoV-2 reference proteome (Wuhan-1, RefSeq: NC_045512.2) was initially selected. In addition, the modifications of the spike protein added to Moderna mRNA-1273 (ModeRNA Therapeutics, Cambridge, MA, USA), Pfizer BNT162b2 (Pfizer, Inc., New York, NY, USA) and Janssen Ad26.COV2.S (Janssen Pharmaceutica, Beerse, Belgium) vaccines were also included. The spike proteins of the following SARS-CoV-1 proteomes were also selected: Beijing-01, RefSeq: AY278488.2; Beijing-02, RefSeq: AY278487; Beijing-03, RefSeq: AY278490; and Beijing-04, RefSeq: AY279354.

### 2.2. Selection of HLA Class I and II Alleles

HLA class I alleles that share anchor residues of the 551 alleles, including in the twelve HLA class I supertypes (A01, A0103, A0124, A02, A03, A24, B07, B08, B27, B44, B58, and B62) [10] and the 16 most frequent HLA-C alleles, were selected. In addition, 41 alleles, including in the ten HLA class II supertypes (DR1, DR52, DR53, DP1, DP3, DQ2, DQ4, DQ5, DQ7, and DQ8), were also included in the study [11,12]. In addition, these HLA class I and II supertypes cover >90% and >95% of the world population regardless of ethnicity [10,11,12].

### 2.3. HLA Class I Epitope Prediction

Non-redundant HLA class I epitopes between 8–12 residues from the spike protein of the SARS-CoV-2 (including the modifications of the spike protein added to the Moderna mRNA-1273, Pfizer BNT162b2, and Janssen Ad26.COV2.S vaccines) were predicted using the latest versions of the universal and neural-network-based netMHCpan EL and BA algorithms. These bioinformatics tools outperform any other method to date [13] and are recommended by the central Immune Epitope Database and Analysis Resource [14]. First, the peptides considered “Strong Binders” (rank ≤ 0.5) by NetMHCIpan EL 4.1 [13] for HLA class I ligands were selected. For redundant epitopes, those sharing the same binding core for the same allele, only the one with the highest score was considered per allele. As the NetMHCIpan EL 4.1 algorith was trained only with mass spectrometry elution (EL) data, non-redundant epitopes were further verified through the NetMHCIpan BA 4.1 [13] algorithm, which includes binding affinity (BA) data and thus, the combination of both bioinformatics tools yield the most accurate results. These verified non-redundant epitopes did not match any of those predicted for a random sequence with the same length and residue composition than the reference SARS-CoV-2 spike protein generated with the EXPASY RandSeq tool (https://web.expasy.org/randseq/ accessed on 12 May 2022). Predictions were restricted to alleles that share anchor residues of the 567 HLA-A, -B, and -C alleles previously selected. To further test the specificity and sensitivity of the NetMHCIpan EL 4.1 algorithm, the substitution of all Pro and Arg/Gln by Ala yielded no epitopes for the HLA-B*07:02 and HLA-B*27:05 alleles, respectively, as these amino acids are their respective anchor motif residues.

### 2.4. HLA Class II Epitope Prediction

Similar to HLA class I, non-redundant HLA class II epitopes between 12–18 residues were predicted using the NetMHCIIpan EL 4.0 [13]. Only the “Strong Binders” (rank ≤ 0.5) were considered with this algorithm, and later were further verified through the NetMHCIIpan BA 4.0 [13] algorithm. As for HLA class I, these verified non-redundant HLA class II epitopes did not match any of those predicted for a random sequence with the same length and residue composition than the reference SARS-CoV-2 spike protein generated with the EXPASY RandSeq tool (https://web.expasy.org/randseq/, accessed on 15 May 2022). Predictions were restricted to alleles that share anchor residues of the 41 HLA-DR, -DP, and -DQ alleles previously selected.

### 2.5. Other Analyses

A theoretical SARS-CoV-2 spike protein with 304 random changes, the number of changes among spike proteins from SARS-CoV-1 and SARS-CoV-2, was generated. Progressive random position datasets were produced by the selection of numbers between 1 and 1273, the spike protein residue length, by the perl rand function. Iterative selection was carried out on the remaining non-selected positions after completing 304 positions. The data of the experimentally detected epitopes were obtained from Immune Epitope Database and Analysis Resource (IEDB; http://www.iedb.org/ accessed on 15 May 2022) [14]. A search in IEDB of the predicted epitopes for the most abundant HLA class I and II alleles in the population was carried out. Positive response for activation and/or cytokine secretion T-cell assay was manually confirmed in the original article describing each epitope. Data of population coverage by HLA class I and II molecules were obtained from IEDB http://tools.iedb.org/population/; accessed on 15 February 2022) [14].

## 3. Results and Discussion

The extraordinary polymorphism of HLA class I and II molecules, with more than 24,000 and 8000 alleles identified to date, respectively, greatly hinders the experimental study of the cellular immune response at the human population level. However, many of the HLA class I and II molecules identified have been grouped first in families, later in superfamilies, and finally in twelve and ten canonical HLA class I and II supertypes, respectively. These supertypes share strong similarities at the peptide-ligand specificity level and include 551 HLA-A and -B class I alleles and other 41 HLA class II alleles. In addition, these twelve and ten HLA class I and II supertypes cover >90% [10] and >95% [11,12] of the world population regardless of ethnicity, respectively. Thus, the use of supertypes significantly reduces data complexity and facilitates the computational analysis of herd immunity.

**Figure 1:**
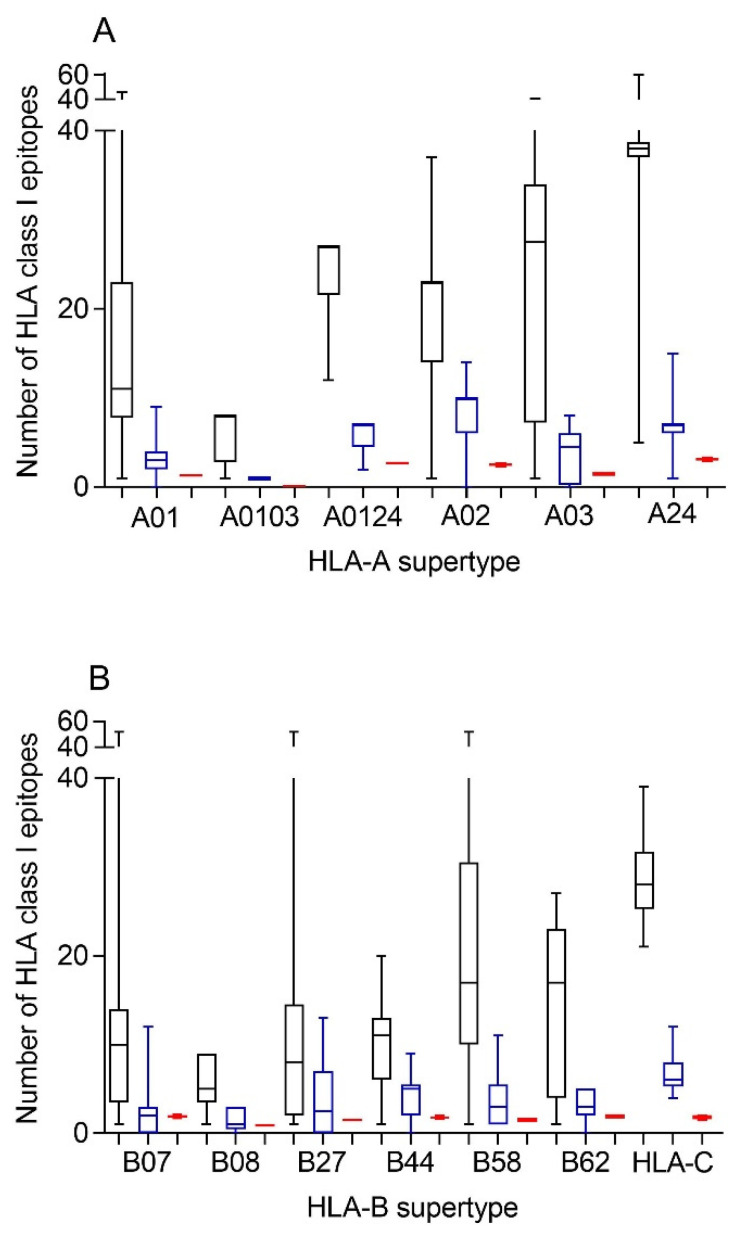
Average number of epitopes in the SARS-CoV-2 spike protein sequence predicted for HLA class I alleles, including the 12 HLA class I supertypes and the 16 most frequent HLA-C alleles and their conservation in SARS-CoV-1. The median value is indicated. Box limits indicate the interquartile range. Whiskers are adjusted to maximal and minimal values. Panel (**A**): number of epitopes in the SARS-CoV-2 (black), the conserved in SARS-CoV-1 (blue), and with 304 random mutations generated over the SARS-CoV-2 spike protein sequence (red) predicted for HLA class I alleles, including in the 6 HLA-A supertypes. Similarly, the epitopes associated with the 6 HLA-B supertypes and the 16 most frequent HLA-C alleles are depicted in panel (**B**).

Therefore, first bioinformatics prediction to the theoretical epitopes from the SARS-CoV-2 spike protein, the viral protein included in internationally licensed vaccines, was carried out as previously described [15,16]. Each of the 551 HLA class I alleles associated with the twelve canonical HLA-A and -B class I supertypes were analyzed, respectively (Figure 1). The predicted ligands for HLA-A class I molecules ranged between nearly 40 epitopes per allele in supertype A24 and less than 10 epitopes per allele for several HLA-A supertypes (Figure 1A, and Supplemental Table S1). Similarly, a predictive analysis of the impact of changes described in the SARS-CoV-1 spike protein over the HLA-A class I supertypes was carried out. As the changes are not randomly distributed throughout the entire spike protein sequences from sarbecoviruses, a significant but not total loss of HLA-A-restricted epitopes derived from SARS-CoV-2 vaccines was detected (Figure 1A and Table 1, Table 2 and Table S1). However, strikingly, for all HLA-A supertypes, the number of HLA-A-restricted epitopes conserved among the spike proteins from both sarbecoviruses was statistically significant versus 304 random mutations generated over the SARS-CoV-2 spike protein sequence (Figure 1A and Table 1). In addition, up to 147 HLA-A class I alleles from all supertypes, except for A0103, retained more than four predicted epitopes conserved among the spike proteins from both SARS-CoVs (Table 3). For example, the HLA-A*26:02 allele from A01 supertype could bind nine conserved epitopes among both spike proteins (Supplemental Table S1). Additionally, seven HLA-A alleles (A*29:01, A*29:02, A*29:06, A*29:09, A*29:10, A*29:11, and A*29:12) from the A0124 supertype retained seven unchanged epitopes (Supplemental Table S1). More strikingly, 31 different HLA-A alleles of the A02 supertype, including HLA-A*02:01, the most prevalent HLA allele in humans, retained more than 10 conserved epitopes on the SARS-CoV-1 spike protein (Table 3 and Table S1). In addition, other five HLA alleles from the A24 supertype retained more than 10 conserved epitopes among the SARS-CoV spike proteins (Table 3 and Table S1).

Similar to the HLA-A locus, a predictive analysis of the impact of changes described in the SARS-CoV-1 spike protein over the HLA class I molecules from the HLA-B supertypes was also carried out. Equal to the HLA-A class I alleles, the changes in the spike protein from SARS-CoV-1 generated a significant, but not total, loss of HLA-B-restricted epitopes derived from SARS-CoV-2 vaccines (Figure 1B and Table 1, Table 2 and Table S1). Newly, all the HLA-B supertypes, except B07, showed a statistically significant difference in the number of HLA-B-restricted epitopes conserved among the spike proteins from both SARS-CoVs versus 304 random mutations generated over the SARS-CoV-2 spike protein sequence (Figure 1B and Table 1). The lack of statistical difference in the B07 supertype among the conserved SARS-CoV-1 epitopes versus random changes was due to the multiple HLA alleles with no or very few conserved epitopes among the spike proteins from SARS-CoVs. However, for the 14 alleles included in this supertype, more than four epitopes conserved among SARS-CoV-1 and SARS-CoV-2 spike proteins were predicted (Table 3). For example, both the HLA-B*35:21 and -B*35:32 alleles could bind seven conserved epitopes among both spike proteins each one, and the HLA-B*35:11 another eight (Supplemental Table S1). Moreover, the HLA-B*35:35 and -B*35:41 alleles from the B07 supertype retained 12 unchanged epitopes among both sarbecoviruses (Table 3 and Table S1). Similarly, another 90 HLA-B alleles from the B27, B44, B58, and B62 supertypes retained more than four predicted epitopes conserved among the spike proteins from SARS-CoV-1 and SARS-CoV-2; of these alleles, 9 even exceed 10 conserved epitopes per HLA-B class I molecule (Table 3). In total, 251 (46%) and 48 (9%) of the HLA-A and -B class I alleles analyzed could bind ≥ 4 or ≥ 10 conserved epitopes among both spike proteins, respectively (Table 3).

Although supertypes have not been defined in HLA-C, 16 alleles from this locus cover >95% of the world population regardless of ethnicity. Thus, similar to the HLA-A and -B loci, a predictive analysis of the impact of changes described in the SARS-CoV-1 spike protein over the SARS-CoV-2 vaccines for these 16 HLA-C class I molecules was carried out. Again, the changes in the spike protein from SARS-CoV-1 generated a significant, but not total, loss of HLA-C-restricted epitopes derived from SARS-CoV-2 vaccines (Figure 1B and Table 1, Table 2 and Table S2). These conserved epitopes among both SARS-CoVs molecules showed a statistically significant versus the 304 random mutations generated over the SARS-CoV-2 spike protein sequence (Figure 1B and Table 1). All HLA-C alleles analyzed retained more than four conserved epitopes among SARS-CoV spike proteins (Table 3 and Table S2). In addition, 3 of these 16 HLA class I molecules (HLA-C*02:02, HLA-C*03:03, and HLA-C*03:04) retained more than 10 conserved epitopes (Table 3 and Table S2).

In summary, 267 (47%) HLA-A, -B, and -C class I alleles analyzed and another 51 (9%) HLA-DR, -DP, and -DQ class II molecules could bind ≥4 or ≥10 conserved epitopes among both spike proteins (Table 3).

**Figure 2:**
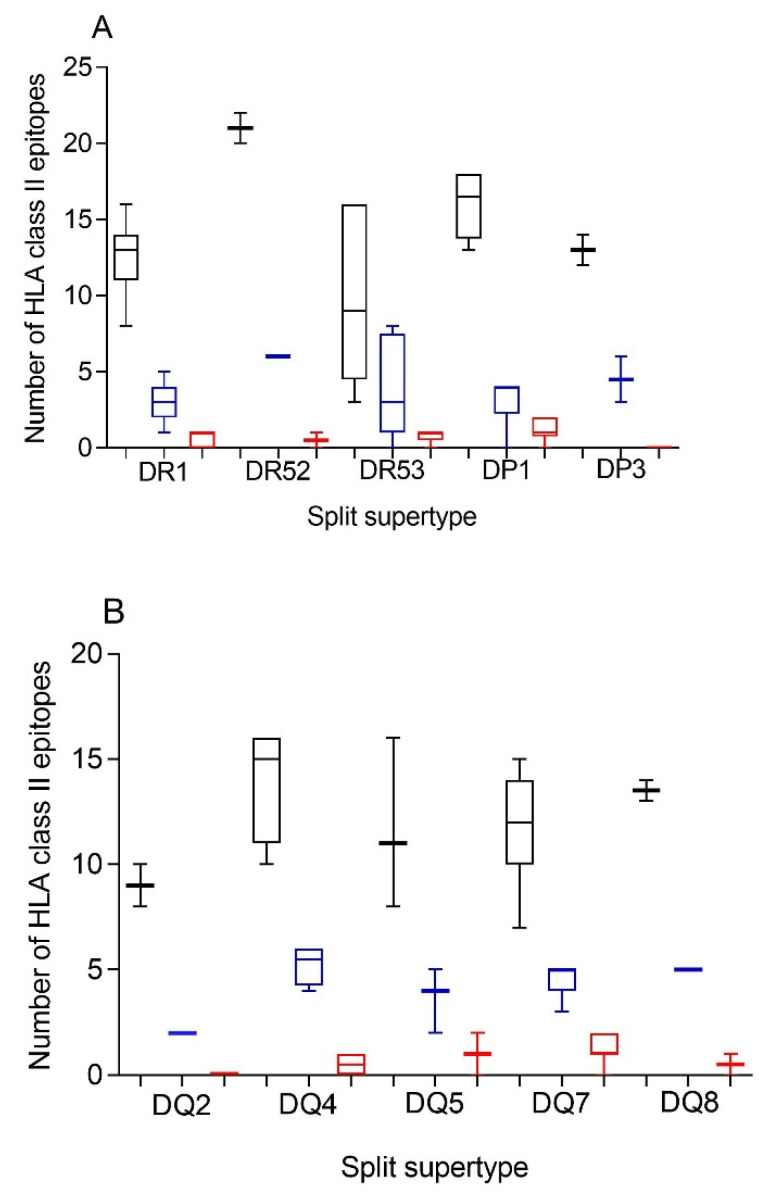
Average number of epitopes in the SARS-CoV-2 spike protein sequence predicted for HLA class II alleles, including in the 10 HLA class II supertypes and their conservation in SARS-CoV-1. The median value is indicated. Box limits indicate the interquartile range. Whiskers are adjusted to maximal and minimal values. The number of epitopes in the SARS-CoV-2 (black), the conserved in SARS-CoV-1 (blue), and with 304 random mutations generated over the SARS-CoV-2 spike protein sequence (red) predicted for HLA class II alleles, including in the 3 HLA-DR, and 2 -DP, or 5 -DQ supertypes are depicted in panels (**A**) or (**B**), respectively.

Additionally, a predictive analysis of the impact of changes described in the SARS-CoV-1 spike protein sequence over the HLA-DR, -DP, and -DQ class II supertypes was also carried out. As Figure 2 and Table 2 and Table S3 show, changes described in the SARS-CoV-1 spike protein versus vaccine sequence generated a significant, but not total, loss of HLA class II-conserved epitopes of both strains for the three DR, two DP, and five DQ supertypes analyzed, similar to the HLA class I-restricted epitopes. These conserved epitopes among both SARS-CoVs molecules showed a statistically significant versus the 304 random mutations generated over the SARS-CoV-2 spike protein sequence for all HLA class II supertypes, except for DR53 (Figure 2 and Table 1). In this supertype, the lack of statistical difference among the conserved SARS-CoV-1 epitopes versus SARS-CoV-2 vaccines and random changes was due to the large dispersion of HLA-DR53 alleles with no or very few conserved epitopes among the spike proteins from SARS-CoVs in some of these HLA class II molecules and others with up to eight conserved epitopes (Figure 2A and Supplemental Table S3). In addition, up to 26 HLA class II alleles from all supertypes, except for DQ2, retained more than four predicted epitopes conserved among the spike proteins from both SARS-CoVs. These alleles were 63% of the total of HLA class II molecules analyzed (Table 3).

The HLA class I frequencies of the 608 HLA class I molecules analyzed in this study range from low prevalence to more than 20% human population for some very frequent alleles as HLA-A*02:01, -A*24:02, or -C*07:02. Thus, to estimate the percentage of human population who might have a sufficient cellular immune response against SARS-CoV-1 with the currently licensed SARS-CoV-2 vaccines, Table 4 shows the HLA class I alleles with a world population coverage >1% that could bind more than four epitopes conserved in SARS-CoV-1. Therefore, seven HLA-A class I alleles, HLA-A*02:01, -A*03:01, -A*11:01, -A*23:01, -A*24:02, -A*29:02, and -A*68:02, cover 80.8% of the human population, regardless of ethnicity (Table 4). Similarly, the other 10 HLA-B and 16 HLA-C alleles with more than four epitopes conserved among SARS-CoV-1 and SARS-CoV-2 cover 40.0% and >95% of the world population, respectively (Table 4). More interesting, the five HLA class I alleles that could bind more than 10 epitopes conserved in SARS-CoV-1, HLA-A*02:01, -B*15:03, -C*02:02, -C*03:03, and -C*03:04, cover 57% of the human population. Similarly, the HLA class II alleles with a world population coverage > 1% that could bind more than four epitopes conserved in SARS-CoV-1 are indicated in Table 5. Thus, five HLA-DR class II alleles, DRB1*07:01, DRB1*09:01, DRB1*16:02, DRB1*13:02, and DRB4*01:01, cover 49.2% of the human population regardless of ethnicity (Table 5). In the other two HLA class II loci, the more frequent 5 HLA-DP and 14 HLA-DQ alleles cover 94.6% and 86.3% of the world population, respectively (Table 5). In summary, the currently licensed vaccines against SARS-CoV-2 could generate enough conserved epitopes in SARS-CoV-1 to trigger a complete cellular immune response restricted by the most frequent HLA class I and class II alleles expressed by the human population.

**Figure 3:**
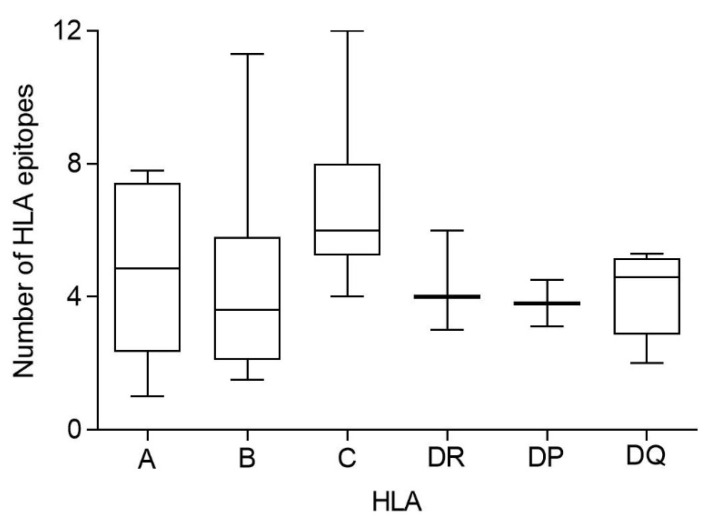
Average number of conserved epitopes among sarbecovirus spike protein sequences predicted for each HLA class I and II locus. The median value is indicated. Box limits indicate the interquartile range. Whiskers are adjusted to maximal and minimal values.

Additionally, the HLA genes are closely linked in the genome and, thus, a set of HLA-A, -B, -C, -DR, -DP, and -DQ genes, called HLA haplotype, is inherited in a Mendelian fashion from each parent. Therefore, the number of conserved epitopes among the SARS-CoV-1 and SARS-CoV-2 spike protein sequences predicted for all HLA class I or II alleles was analyzed by HLA loci. Figure 3 shows an average of five, three, and seven conserved epitopes for HLA-A, -B, and -C loci, respectively, and another four conserved epitopes for each HLA class II locus. Thus, on average, 15 conserved epitopes of the HLA class I and another 12 of the HLA class II could be associated with each individual HLA haplotype (Figure 3), and the different HLA molecules in a homozygous individual would present these 27 conserved epitopes. However, as less of 15% of humans are homozygotes for HLA [17], the currently licensed vaccines against SARS-CoV-2 could generate an average of 30 HLA class I epitopes and another 24 HLA class II epitopes conserved in SARS-CoV-1 for more than 85% of human population. This striking relative abundance of conserved epitopes among sarbecovirus spike proteins is because, unlike random mutations, the entire viral protein sequence cannot change randomly. However, 304 random mutational changes virtually cover the entire protein sequence, destroying almost all epitopes, as indicated in Figure 1 and Figure 2. In contrast, 27 segments that include between 9 and 111 consecutive residues are conserved among sarbecovirus spike proteins, which accumulated 304 changes. Thus, up to 579 amino acids of SARS-CoV-1 can be used by the immune system to generate HLA-restricted epitopes conserved with SARS-CoV-2. In contrast, the spike protein from MERS-CoV, another member of the betacoronavirus genus related to sarbecoviruses, presents only 371 conserved residues with SARS-CoV-2. Additionally, these residues are in practice randomly distributed throughout the protein sequence; thus, there are no conserved epitopes among MERS-CoV and SARS-CoV-2 for any HLA class I and II allele.

Finally, in this study, the viral epitopes were computationally predicted and, therefore, experimental confirmation is needed. In this pandemic context, currently the number of experimentally detected HLA-restricted epitopes from SARS-CoV-2 included in the IEDB database is continuously increased [14]. Obviously, the most abundant HLA-A and -B alleles in the population are also the most extensively studied. For this reason, a search in the IEDB database of the epitopes conserved among both coronaviruses for those alleles of HLA-A and -B with a world population coverage >5% and with ≥4 predicted epitopes conserved among sarbecoviruses was carried out. As shown in Table 6, the vast majority of these predicted epitopes were defined as epitopes recognized by CD8^+^ T cells. The coincidence percentage between the predicted and experimentally detected epitopes ranged from 71% in HLA-A*11:01 to 100% for HLA-A*02:01 and -A*23:01 (Table 6). Among these five HLA-A alleles, which cover the 77.8% of the world population, 89% of the predicted epitopes were functionally detected (Table 6). Additionally, in the four HLA-B alleles analyzed, which cover 28.4% of the world population, 78% of the predicted epitopes are included in the IEDB database as confirmed epitopes (Table 6). Overall, 50 of the 59 predicted epitopes associated with HLA-A and -B class I molecules (83.3%) are defined in the IEDB database as targets recognized by CD8^+^ T cells (Table 6). Similarly, a search in the IEDB database of the epitopes conserved among both coronaviruses for those HLA-DR class II alleles with ≥4 predicted epitopes conserved among sarbecoviruses was carried out. Only 1 of these 27 predicted epitopes, which was associated with HLA-DRB4*01:01, was not included in the IEDB database as a target of CD4^+^ T cells (Table 7). In addition, 11 of the 12 predicted epitopes associated with the three most frequent HLA-DP alleles were experimentally detected as targets of CD4^+^ T cells (Table 7). Lastly, all predicted epitopes associated with the six most frequent HLA-DQ alleles were included in the IEDB database as targets of CD4^+^ T cells (Table 7). Overall, 97% of the predicted epitopes associated with these 14 HLA class II alleles were experimentally identified as targets recognized by CD4^+^ T cells (Table 7). These results indicate a very high correlation between the bioinformatics prediction and the experimental assays, at least for conserved epitopes among coronavirus spike proteins, and validate the methodological approach. Importantly, those predicted epitopes that are not currently included in the IEDB database may not have been tested and could be detected as epitopes recognized by T cells in future studies.

More interesting, the current study has potential implications for public health against current and future pandemics. In addition to humoral response, if many epitopes associated with multiple and frequent HLA class I and II molecules are conserved among sarbecovirus, the currently licensed vaccines against COVID-19 must be effective against SARS-CoV-1 infection. Similarly, SARS-CoV-1 vaccines could generate cross-protection against SARS-CoV-2 infection. In addition, for years, it has been known that different SARS-CoV-1 spike-protein-based vaccines elicit potent immune responses and protective effects in preclinical models [18,19,20], and even in phase I human studies ([21] and Clinicaltrial.gov: NCT00533741 and NCT01376765). However, the first COVID-19 vaccine, the “Pfizer-BioNTech COVID-19 Vaccine”, was approved by the FDA in August 2021. Thus, if while COVID-19-specific vaccines began to be developed, the SARS-CoV-1 vaccines in the pipeline had been included in more advanced phases of clinical trials, then perhaps they could have been available to prevent part of the hundreds of thousands of deaths caused by COVID-19 in 2020. In this context, and in line with the data presented above, a very recent study using a mice model has shown that SARS-CoV-1 vaccination induces cross-reactive antibodies and T cells against SARS-CoV-2 and protects against a SARS-CoV-2 challenge [22]. Thus, the use of bioinformatics analysis such as the one developed in the present study and similar exploration of cross-reactive humoral responses could be a useful rapid response strategy to face future pandemics with the vaccine tools available at that time.

Finally, a computational analysis such as the one carried out in the present study can be extended to analyze the influence of virus molecular evolution on the cellular immune response. Therefore, HLA-restricted epitopes of the different virus variants emerging over time in different countries can be analyzed. These studies are very relevant because the effect of emerging virus variants on vaccine efficacy is of critical importance, and the potential impact of mutations that could facilitate escape from the cellular immune response would allow to check for convenient or optimized vaccine candidates. These studies have recently been carried out in our laboratory with all the relevant SARS-CoV-2 strains up to the Omicron variant of concern, showing that most of the HLA class I and II alleles, which cover >90% of the population, contain enough HLA-restricted epitopes without escape mutations [15,16]. These data previously published by our laboratory indicated that the cellular immune protection elicited by the currently licensed vaccines was not affected by emerging SARS-CoV-2 variants [15,16].

## Figures and Tables

**Table 1:** Statistical significance of the number of predicted HLA class I and class II epitopes.

Supertype	SARS1/SARS2 *p*-Value	SARS1/304 Random Mutation *p*-Value
A01	<0.0001	0.0026
A0103	0.024	0.025
A0124	<0.0001	0.0005
A02	<0.0001	<0.0001
A03	<0.0001	0.0064
A24	<0.0001	<0.0001
B07	<0.0001	n.s. ^a^
B08	0.002	0.04
B27	<0.0001	0.01
B44	<0.0001	<0.0001
B58	<0.0001	0.007
B62	<0.0001	0.002
HLA-C	<0.0001	<0.0001
DR1	<0.0001	0.0003
DR52	0.0044	0.0082
DR53	n.s.	n.s.
DP1	<0.0001	0.02
DP3	0.04	0.04
DQ2	0.01	0.01
DQ4	0.0011	0.0001
DQ5	0.022	0.048
DQ7	<0.0001	<0.0001
DQ8	0.0034	0.01

^a^ Not significant.

**Table 2:** Percentage of predicted HLA class I and class II epitopes conserved among sarbecoviruses’ spike proteins.

Supertype	% of Conserved Epitopes
A01	19
A0103	16
A0124	24
A02	32
A03	17
A24	19
B07	19
B08	30
B27	33
B44	44
B58	19
B64	20
HLA-C	25
DR1	24
DR52	29
DR53	40
DP1	20
DP3	35
DQ2	22
DQ4	38
DQ5	32
DQ7	40
DQ8	37

**Table 3:** Number of HLA alleles with more than 4 or 10 predicted epitopes conserved among sarbecoviruses’ spike proteins.

HLA Superfamily	Number of HLA Alleles with	
	≥4 Epitopes Conserved	≥10 Epitopes Conserved
A01	12	0
A0103	0	0
A0124	7	0
A02	53	31
A03	44	0
A24	31	5
B07	14	2
B08	0	0
B27	22	7
B44	44	1
B58	7	1
B62	17	0
HLA-C	16	3
No. of HLA class I alleles	267 (47%)	51
DR1	3	0
DR52	2	0
DR53	3	0
DP1	4	0
DP3	2	0
DQ2	0	0
DQ4	3	0
DQ5	2	0
DQ7	5	0
DQ8	2	0
No. of HLA class II alleles	26 (63%)	0

**Table 4:** Predicted epitopes conserved in SARS-CoV-1 and % population coverage in the most frequent HLA class I alleles.

HLA Class I Allele	Supertype	Epitopes Conserved in SARS-CoV-1	% Population Coverage ^a^
A*29:02	A0124	7	3.9
A*02:01	A02	10	39.1
A*68:02	A02	7	2.5
A*03:01	A03	6	16.8
A*11:01	A03	7	15.5
A*23:01	A24	6	5.4
A*24:02	A24	7	21.4
7 HLA-A alleles			80.8
B*35:01	B07	6	8.4
B*15:03	B27	11	1.3
B*27:05	B27	8	4.8
B*39:02	B27	7	1.0
B*40:01	B44	9	7.8
B*40:02	B44	8	3.5
B*40:06	B44	4	1.1
B*44:02	B44	4	7.6
B*44:03	B44	4	6.7
B*45:01	B44	5	1.3
10 HLA-B alleles			40.0
17 HLA-A, and -B alleles			86.1
C*01:02		4	10.5
C*02:02		11	9.5
C*03:03		12	8.1
C*03:04		12	12.8
C*04:01		8	20.0
C*05:01		5	7.9
C*06:02		6	15.5
C*07:01		5	19.4
C*07:02		6	21.5
C*08:01		6	4.6
C*08:02		6	4.2
C*12:03		6	10.3
C*14:02		6	3.0
C*15:02		8	4.4
C*16:01		5	4.7
C*17:01		8	3.3
16 HLA-C alleles			>95
33 HLA class I alleles			>95

^a^ Only HLA class I molecules with a world population coverage > 1% were included.

**Table 5:** Predicted epitopes conserved in SARS-CoV-1 and % population coverage in the most frequent HLA class II alleles.

HLA Class II Alelle	Supertype	Epitopes Conserved in SARS-CoV-1	% Population Coverage ^a^
DRB1*07:01	DR1	4	18.2
DRB1*09:01	DR1	5	6.4
DRB1*16:02	DR1	4	2.0
DRB1*13:02	DR52	6	6.7
DRB4*01:01	DR53	8	41.8
5 HLA-DR alleles			49.2
HLA-DPA1*01:03-DPB1*02:01	DP1	4	76.4
HLA-DPA1*01:03-DPB1*04:01	DP1	4	79.2
HLA-DPA1*01:03-DPB1*06:01	DP1	4	70.2
HLA-DPA1*03:01-DPB1*04:02	DP1	4	27.5
HLA-DPA1*02:01-DPB1*14:01	DP3	6	32.8
5 HLA-DP alleles			94.6
HLA-DQA1*02:01-DQB1*04:02	DQ4	6	24.7
HLA-DQA1*03:03-DQB1*04:02	DQ4	6	16.0
HLA-DQA1*05:01-DQB1*04:02	DQ4	4	41.0
HLA-DQA1*06:01-DQB1*04:02	DQ4	5	13.2
HLA-DQA1*01:02-DQB1*05:02	DQ5	4	30.8
HLA-DQA1*01:04-DQB1*05:03	DQ5	5	13.8
HLA-DQA1*01:03-DQB1*06:03	DQ7	4	19.1
HLA-DQA1*02:01-DQB1*03:01	DQ7	5	44.4
HLA-DQA1*02:01-DQB1*03:03	DQ7	5	25.3
HLA-DQA1*05:01-DQB1*03:01	DQ7	5	41.5
HLA-DQA1*05:01-DQB1*03:02	DQ7	5	46.8
HLA-DQA1*05:01-DQB1*03:03	DQ7	5	41.5
HLA-DQA1*03:01-DQB1*03:02	DQ8	5	40.2
HLA-DQA1*04:01-DQB1*04:02	DQ8	5	17.6
14 HLA-DQ alleles			86.3
24 HLA class II alleles			>95

^a^ Only HLA class II molecules with a world population coverage > 1% were included.

**Table 6:** Predicted and experimentally detected HLA class I epitopes conserved among sarbecoviruses.

HLA Class I Allele	HLA Class I Epitopes Conserved among Sarbecoviruses			% Population Coverage ^c^
	Predicted ^a^	Experimentally Confirmed ^b^	% Experimental versus Predicted	
A*02:01	10	10	100	39.1
A*03:01	6	5	83	16.8
A*11:01	7	5	71	15.5
A*23:01	6	6	100	5.4
A*24:02	7	6	86	21.4
5 HLA-A alleles	36	32	89	77.8
B*35:01	6	5	83	8.4
B*40:01	9	7	78	7.8
B*44:02	4	3	75	7.6
B*44:03	4	3	75	6.7
4 HLA-B alleles	23	18	78	28.4
9 HLA-A, and -B alleles	59	50	85	83.3

^a^ From this study (Table 4). ^b^ Positive for activation and/or cytokine secretion T-cell assays obtained from the IEDB database. ^c^ Only HLA-A and -B class I molecules with a world population coverage >5% were included.

**Table 7:** Predicted and experimentally detected HLA class II epitopes conserved among sarbecoviruses.

HLA Class II Allele	HLA Class II Epitopes Conserved among Sarbecoviruses			% Population Coverage ^c^
	Predicted ^a^	Experimentally Confirmed ^b^	% Experimental versus Predicted	
DRB1*07:01	4	4	100	18.2
DRB1*09:01	5	5	100	6.4
DRB1*16:02	4	4	100	2.0
DRB1*13:02	6	6	100	6.7
DRB4*01:01	8	7	88	41.8
5 HLA-DR alleles	27	26	96	49.2
HLA-DPA1*01:03-DPB1*02:01	4	3	75	76.4
HLA-DPA1*01:03-DPB1*04:01	4	4	100	79.2
HLA-DPA1*01:03-DPB1*06:01	4	4	100	70.2
3 HLA-DP alleles	12	11	92	89.8
HLA-DQA1*05:01-DQB1*04:02	4	4	100	41.0
HLA-DQA1*02:01-DQB1*03:01	5	5	100	44.4
HLA-DQA1*05:01-DQB1*03:01	5	5	100	41.5
HLA-DQA1*05:01-DQB1*03:02	5	5	100	46.8
HLA-DQA1*05:01-DQB1*03:03	5	5	100	41.5
HLA-DQA1*03:01-DQB1*03:02	5	5	100	40.2
6 HLA-DQ alleles	29	29	100	87.3
14 HLA class II alleles	68	66	97	>95

^a^ From this study (Table 5). ^b^ Positive for activation and/or cytokine secretion T-cell assays obtained from the IEDB database. ^c^ All HLA-DR alleles with ≥4 predicted epitopes conserved among sarbecoviruses and the HLA-DP and -DQ class II molecules with a world population coverage >40% were included.

## Data Availability

All data are included in Supplementary Tables S1–S3.

## Supplementary Materials

The following supporting information can be downloaded at: https://www.mdpi.com/article/10.3390/biomedicines10071622/s1, Table S1: Predicted ligands for HLA-A, and B class I molecules; Table S2: Predicted ligands for HLA-C class I molecules; Table S3: Predicted ligands for HLA class II molecules.
