# Supplementary material for: Prediction of Conserved HLA Class I and Class II Epitopes from SARS-CoV-2 Licensed Vaccines Supports T-Cell Cross-Protection against SARS-CoV-1
Source: Biomedicines. 2022 Jul 7;10(7):1622. doi: 10.3390/biomedicines10071622 (PMC9313420; doi:10.3390/biomedicines10071622)
Supplement: Supplementary file 1 [file biomedicines-10-01622-s001.zip › Supplemental Table S1.pdf]

Supertype	Allele	Begin positior	End position	Sequence	Score (EL)
A01	HLA-A*01:01	28	37	YTNSFTRGVY	0.8696
A01	HLA-A*01:01	28	38	YTNSFTRGVYY	0.9035
A01	HLA-A*01:01	135	144	FCNDPFLGVY	0.6253
A01	HLA-A*01:01	136	144	CNDPFLGVY	0.6408
A01	HLA-A*01:01	136	145	CNDPFLGVYY	0.6059
A01	HLA-A*01:01	160	170	YSSANNCTFEY	0.7273
A01	HLA-A*01:01	161	170	SSANNCTFEY	0.7703
A01	HLA-A*01:01	162	170	SANNCTFEY	0.6674
A01	HLA-A*01:01	256	266	SGWTAGAAAYY	0.7466
A01	HLA-A*01:01	257	266	GWTAGAAAYY	0.7407
A01	HLA-A*01:01	258	266	WTAGAAAYY	0.9622
A01	HLA-A*01:01	358	369	ISNCVADYSVLY	0.7628
A01	HLA-A*01:01	361	369	CVADYSVLY	0.8233
A01	HLA-A*01:01	362	369	VADYSVLY	0.8361
A01	HLA-A*01:01	370	380	NSASFSTFKCY	0.5177
A01	HLA-A*01:01	440	449	NLDSKVGGNY	0.893
A01	HLA-A*01:01	603	612	NTSNQVAVLY	0.8721
A01	HLA-A*01:01	604	612	TSNQVAVLY	0.9585
A01	HLA-A*01:01	746	756	STECSNLLLQY	0.8875
A01	HLA-A*01:01	828	837	LADAGFIKQY	0.928
A01	HLA-A*01:01	863	873	PLLTDEMIAQY	0.8726
A01	HLA-A*01:01	865	873	LTDEMIAQY	0.9987
A01	HLA-A*01:01	866	873	TDEMIAQY	0.8997
A01	HLA-A*01:01	1197	1206	LIDLQELGKY	0.9031
A01	HLA-A*01:03	28	37	YTNSFTRGVY	0.8142
A01	HLA-A*01:03	28	38	YTNSFTRGVYY	0.8662
A01	HLA-A*01:03	160	170	YSSANNCTFEY	0.6327
A01	HLA-A*01:03	161	170	SSANNCTFEY	0.6876
A01	HLA-A*01:03	258	266	WTAGAAAYY	0.9493
A01	HLA-A*01:03	358	369	ISNCVADYSVLY	0.6647
A01	HLA-A*01:03	361	369	CVADYSVLY	0.7732
A01	HLA-A*01:03	603	612	NTSNQVAVLY	0.8133
A01	HLA-A*01:03	604	612	TSNQVAVLY	0.9385
A01	HLA-A*01:03	746	756	STECSNLLLQY	0.8277
A01	HLA-A*01:03	828	837	LADAGFIKQY	0.8857
A01	HLA-A*01:03	863	873	PLLTDEMIAQY	0.7915
A01	HLA-A*01:03	865	873	LTDEMIAQY	0.9982
A01	HLA-A*01:03	1197	1206	LIDLQELGKY	0.8476
A01	HLA-A*01:06	28	37	YTNSFTRGVY	0.7602
A01	HLA-A*01:06	28	38	YTNSFTRGVYY	0.7892
A01	HLA-A*01:06	29	38	TNSFTRGVYY	0.5721
A01	HLA-A*01:06	30	38	NSFTRGVYY	0.8159
A01	HLA-A*01:06	160	170	YSSANNCTFEY	0.5052
A01	HLA-A*01:06	161	170	SSANNCTFEY	0.7022
A01	HLA-A*01:06	162	170	SANNCTFEY	0.6641
A01	HLA-A*01:06	256	266	SGWTAGAAAYY	0.6894
A01	HLA-A*01:06	257	266	GWTAGAAAYY	0.7487
A01	HLA-A*01:06	258	266	WTAGAAAYY	0.9282
A01	HLA-A*01:06	358	369	ISNCVADYSVLY	0.607

A01	HLA-A*01:06	359	369	SNCVADYSVLY	0.5742
A01	HLA-A*01:06	360	369	NCVADYSVLY	0.6328
A01	HLA-A*01:06	361	369	CVADYSVLY	0.8242
A01	HLA-A*01:06	601	612	GTNTSNQVAVLY	0.5577
A01	HLA-A*01:06	603	612	NTSNQVAVLY	0.7856
A01	HLA-A*01:06	604	612	TSNQVAVLY	0.9241
A01	HLA-A*01:06	733	741	KTSVDCTMY	0.6247
A01	HLA-A*01:06	746	756	STECSNLLLQY	0.5958
A01	HLA-A*01:06	828	837	LADAGFIKQY	0.7349
A01	HLA-A*01:06	865	873	LTDEMIAQY	0.9873
A01	HLA-A*01:06	1197	1206	LIDLQELGKY	0.6364
A01	HLA-A*01:07	28	37	YTNSFTRGVY	0.7994
A01	HLA-A*01:07	28	38	YTNSFTRGVYY	0.7935
A01	HLA-A*01:07	135	144	FCNDPFLGVY	0.5269
A01	HLA-A*01:07	136	144	CNDPFLGVY	0.5497
A01	HLA-A*01:07	136	145	CNDPFLGVYY	0.5021
A01	HLA-A*01:07	160	170	YSSANNCTFEY	0.6031
A01	HLA-A*01:07	161	170	SSANNCTFEY	0.7204
A01	HLA-A*01:07	162	170	SANNCTFEY	0.6395
A01	HLA-A*01:07	256	266	SGWTAGAAAYY	0.6935
A01	HLA-A*01:07	257	266	GWTAGAAAYY	0.7199
A01	HLA-A*01:07	258	266	WTAGAAAYY	0.9322
A01	HLA-A*01:07	358	369	ISNCVADYSVLY	0.6325
A01	HLA-A*01:07	361	369	CVADYSVLY	0.7815
A01	HLA-A*01:07	440	449	NLDSKVGGNY	0.8818
A01	HLA-A*01:07	603	612	NTSNQVAVLY	0.8153
A01	HLA-A*01:07	604	612	TSNQVAVLY	0.9147
A01	HLA-A*01:07	746	756	STECSNLLLQY	0.728
A01	HLA-A*01:07	828	837	LADAGFIKQY	0.8525
A01	HLA-A*01:07	863	873	PLLTDEMIAQY	0.7608
A01	HLA-A*01:07	865	873	LTDEMIAQY	0.9944
A01	HLA-A*01:07	1197	1206	LIDLQELGKY	0.8505
A01	HLA-A*01:08	28	37	YTNSFTRGVY	0.8357
A01	HLA-A*01:08	28	38	YTNSFTRGVYY	0.8794
A01	HLA-A*01:08	135	144	FCNDPFLGVY	0.5792
A01	HLA-A*01:08	136	144	CNDPFLGVY	0.5738
A01	HLA-A*01:08	136	145	CNDPFLGVYY	0.5287
A01	HLA-A*01:08	160	170	YSSANNCTFEY	0.71
A01	HLA-A*01:08	161	170	SSANNCTFEY	0.7846
A01	HLA-A*01:08	162	170	SANNCTFEY	0.6944
A01	HLA-A*01:08	254	265	SSSGWTAGAAAY	0.5093
A01	HLA-A*01:08	256	266	SGWTAGAAAYY	0.748
A01	HLA-A*01:08	257	266	GWTAGAAAYY	0.7602
A01	HLA-A*01:08	258	266	WTAGAAAYY	0.9628
A01	HLA-A*01:08	358	369	ISNCVADYSVLY	0.7216
A01	HLA-A*01:08	361	369	CVADYSVLY	0.8161
A01	HLA-A*01:08	362	369	VADYSVLY	0.7907
A01	HLA-A*01:08	603	612	NTSNQVAVLY	0.8442
A01	HLA-A*01:08	604	612	TSNQVAVLY	0.9539
A01	HLA-A*01:08	697	707	MSLGAENSVAY	0.5924

A01	HLA-A*01:08	733	741	KTSVDCTMY	0.6742
A01	HLA-A*01:08	746	756	STECSNLLLQY	0.8198
A01	HLA-A*01:08	828	837	LADAGFIKQY	0.9
A01	HLA-A*01:08	863	873	PLLTDEMIAQY	0.7815
A01	HLA-A*01:08	865	873	LTDEMIAQY	0.9976
A01	HLA-A*01:08	866	873	TDEMIAQY	0.8381
A01	HLA-A*01:08	1197	1206	LIDLQELGKY	0.8632
A01	HLA-A*01:09	28	37	YTNSFTRGVY	0.8696
A01	HLA-A*01:09	28	38	YTNSFTRGVYY	0.9035
A01	HLA-A*01:09	135	144	FCNDPFLGVY	0.6253
A01	HLA-A*01:09	136	144	CNDPFLGVY	0.6408
A01	HLA-A*01:09	136	145	CNDPFLGVYY	0.6059
A01	HLA-A*01:09	160	170	YSSANNCTFEY	0.7273
A01	HLA-A*01:09	161	170	SSANNCTFEY	0.7703
A01	HLA-A*01:09	162	170	SANNCTFEY	0.6674
A01	HLA-A*01:09	256	266	SGWTAGAAAYY	0.7466
A01	HLA-A*01:09	257	266	GWTAGAAAYY	0.7407
A01	HLA-A*01:09	258	266	WTAGAAAYY	0.9622
A01	HLA-A*01:09	358	369	ISNCVADYSVLY	0.7628
A01	HLA-A*01:09	361	369	CVADYSVLY	0.8233
A01	HLA-A*01:09	362	369	VADYSVLY	0.8361
A01	HLA-A*01:09	370	380	NSASFSTFKCY	0.5177
A01	HLA-A*01:09	440	449	NLDSKVGGNY	0.893
A01	HLA-A*01:09	603	612	NTSNQVAVLY	0.8721
A01	HLA-A*01:09	604	612	TSNQVAVLY	0.9585
A01	HLA-A*01:09	746	756	STECSNLLLQY	0.8875
A01	HLA-A*01:09	828	837	LADAGFIKQY	0.928
A01	HLA-A*01:09	863	873	PLLTDEMIAQY	0.8726
A01	HLA-A*01:09	865	873	LTDEMIAQY	0.9987
A01	HLA-A*01:09	866	873	TDEMIAQY	0.8997
A01	HLA-A*01:09	1197	1206	LIDLQELGKY	0.9031
A01	HLA-A*01:10	28	37	YTNSFTRGVY	0.8782
A01	HLA-A*01:10	28	38	YTNSFTRGVYY	0.8568
A01	HLA-A*01:10	135	144	FCNDPFLGVY	0.5464
A01	HLA-A*01:10	136	144	CNDPFLGVY	0.55
A01	HLA-A*01:10	160	170	YSSANNCTFEY	0.617
A01	HLA-A*01:10	161	170	SSANNCTFEY	0.7739
A01	HLA-A*01:10	162	170	SANNCTFEY	0.7209
A01	HLA-A*01:10	254	265	SSSGWTAGAAAY	0.5306
A01	HLA-A*01:10	256	266	SGWTAGAAAYY	0.7805
A01	HLA-A*01:10	257	266	GWTAGAAAYY	0.801
A01	HLA-A*01:10	258	266	WTAGAAAYY	0.9558
A01	HLA-A*01:10	269	279	YLQPRTFLLKY	0.5653
A01	HLA-A*01:10	358	369	ISNCVADYSVLY	0.7038
A01	HLA-A*01:10	361	369	CVADYSVLY	0.8467
A01	HLA-A*01:10	362	369	VADYSVLY	0.7818
A01	HLA-A*01:10	414	423	QTGKIADYNY	0.7033
A01	HLA-A*01:10	440	449	NLDSKVGGNY	0.8551
A01	HLA-A*01:10	601	612	GTNTSNQVAVLY	0.6737
A01	HLA-A*01:10	603	612	NTSNQVAVLY	0.8545

A01	HLA-A*01:10	604	612	TSNQVAVLY	0.9462
A01	HLA-A*01:10	733	741	KTSVDCTMY	0.7195
A01	HLA-A*01:10	746	756	STECSNLLLQY	0.8093
A01	HLA-A*01:10	828	837	LADAGFIKQY	0.8825
A01	HLA-A*01:10	863	873	PLLTDEMIAQY	0.6841
A01	HLA-A*01:10	865	873	LTDEMIAQY	0.996
A01	HLA-A*01:10	1039	1047	RVDFCGKGY	0.8273
A01	HLA-A*01:10	1197	1206	LIDLQELGKY	0.875
A01	HLA-A*01:12	28	37	YTNSFTRGVY	0.5329
A01	HLA-A*01:12	28	38	YTNSFTRGVYY	0.528
A01	HLA-A*01:12	161	170	SSANNCTFEY	0.5055
A01	HLA-A*01:12	162	170	SANNCTFEY	0.5654
A01	HLA-A*01:12	258	266	WTAGAAAYY	0.8102
A01	HLA-A*01:12	361	369	CVADYSVLY	0.7278
A01	HLA-A*01:12	444	453	KVGGNYNYLY	0.594
A01	HLA-A*01:12	603	612	NTSNQVAVLY	0.5927
A01	HLA-A*01:12	604	612	TSNQVAVLY	0.7812
A01	HLA-A*01:12	865	873	LTDEMIAQY	0.9207
A01	HLA-A*01:14	28	37	YTNSFTRGVY	0.8208
A01	HLA-A*01:14	28	38	YTNSFTRGVYY	0.8729
A01	HLA-A*01:14	135	144	FCNDPFLGVY	0.6119
A01	HLA-A*01:14	136	144	CNDPFLGVY	0.6715
A01	HLA-A*01:14	136	145	CNDPFLGVYY	0.6088
A01	HLA-A*01:14	160	170	YSSANNCTFEY	0.663
A01	HLA-A*01:14	161	170	SSANNCTFEY	0.7519
A01	HLA-A*01:14	162	170	SANNCTFEY	0.6665
A01	HLA-A*01:14	256	266	SGWTAGAAAYY	0.6944
A01	HLA-A*01:14	257	266	GWTAGAAAYY	0.7187
A01	HLA-A*01:14	258	266	WTAGAAAYY	0.9506
A01	HLA-A*01:14	269	279	YLQPRTFLLKY	0.5565
A01	HLA-A*01:14	358	369	ISNCVADYSVLY	0.7087
A01	HLA-A*01:14	361	369	CVADYSVLY	0.8057
A01	HLA-A*01:14	362	369	VADYSVLY	0.7998
A01	HLA-A*01:14	601	612	GTNTSNQVAVLY	0.6797
A01	HLA-A*01:14	603	612	NTSNQVAVLY	0.8237
A01	HLA-A*01:14	604	612	TSNQVAVLY	0.9452
A01	HLA-A*01:14	733	741	KTSVDCTMY	0.7034
A01	HLA-A*01:14	746	756	STECSNLLLQY	0.8081
A01	HLA-A*01:14	828	837	LADAGFIKQY	0.8737
A01	HLA-A*01:14	863	873	PLLTDEMIAQY	0.7423
A01	HLA-A*01:14	865	873	LTDEMIAQY	0.9971
A01	HLA-A*01:14	1039	1047	RVDFCGKGY	0.8252
A01	HLA-A*01:14	1197	1206	LIDLQELGKY	0.8561
A01	HLA-A*26:01	28	37	YTNSFTRGVY	0.5472
A01	HLA-A*26:01	191	200	EFVFKNIDGY	0.8157
A01	HLA-A*26:01	192	200	FVFKNIDGY	0.9119
A01	HLA-A*26:01	258	266	WTAGAAAYY	0.9338
A01	HLA-A*26:01	340	351	EVFNATRFASVY	0.6393
A01	HLA-A*26:01	360	369	NCVADYSVLY	0.7886
A01	HLA-A*26:01	361	369	CVADYSVLY	0.8578

A01	HLA-A*26:01	603	612	NTSNQVAVLY	0.5793
A01	HLA-A*26:01	686	695	SVASQSIIAY	0.6831
A01	HLA-A*26:01	780	789	EVFAQVKQIY	0.7706
A01	HLA-A*26:01	1095	1103	FVSNGTHWF	0.5675
A01	HLA-A*26:02	28	37	YTNSFTRGVY	0.7649
A01	HLA-A*26:02	30	38	NSFTRGVYY	0.7196
A01	HLA-A*26:02	50	58	STQDLFLPF	0.7114
A01	HLA-A*26:02	125	133	NVVIKVCEF	0.6379
A01	HLA-A*26:02	191	200	EFVFKNIDGY	0.9206
A01	HLA-A*26:02	192	200	FVFKNIDGY	0.9721
A01	HLA-A*26:02	192	201	FVFKNIDGYF	0.7022
A01	HLA-A*26:02	215	223	DLPQGFSAL	0.5481
A01	HLA-A*26:02	256	266	SGWTAGAAAYY	0.5903
A01	HLA-A*26:02	257	266	GWTAGAAAYY	0.6462
A01	HLA-A*26:02	258	265	WTAGAAAY	0.5731
A01	HLA-A*26:02	258	266	WTAGAAAYY	0.9678
A01	HLA-A*26:02	261	269	GAAAYYVGY	0.6857
A01	HLA-A*26:02	298	306	ETKCTLKSF	0.7757
A01	HLA-A*26:02	340	347	EVFNATRF	0.5818
A01	HLA-A*26:02	340	351	EVFNATRFASVY	0.834
A01	HLA-A*26:02	343	351	NATRFASVY	0.526
A01	HLA-A*26:02	359	369	SNCVADYSVLY	0.6372
A01	HLA-A*26:02	360	369	NCVADYSVLY	0.9187
A01	HLA-A*26:02	361	369	CVADYSVLY	0.9515
A01	HLA-A*26:02	366	374	SVLYNSASF	0.6928
A01	HLA-A*26:02	392	400	FTNVYADSF	0.6269
A01	HLA-A*26:02	442	451	DSKVGGNYNY	0.6561
A01	HLA-A*26:02	554	562	ESNKKFLPF	0.6456
A01	HLA-A*26:02	568	576	DIADTTDAV	0.5453
A01	HLA-A*26:02	583	592	EILDITPCSF	0.8274
A01	HLA-A*26:02	603	611	NTSNQVAVL	0.5594
A01	HLA-A*26:02	603	612	NTSNQVAVLY	0.7784
A01	HLA-A*26:02	686	695	SVASQSIIAY	0.8793
A01	HLA-A*26:02	691	699	SIIAYTMSL	0.7185
A01	HLA-A*26:02	710	718	NSIAIPTNF	0.7355
A01	HLA-A*26:02	718	726	FTISVTTEI	0.7944
A01	HLA-A*26:02	780	788	EVFAQVKQI	0.672
A01	HLA-A*26:02	780	789	EVFAQVKQIY	0.8897
A01	HLA-A*26:02	865	873	LTDEMIAQY	0.7697
A01	HLA-A*26:02	869	877	MIAQYTSAL	0.7428
A01	HLA-A*26:02	880	888	GTITSGWTF	0.5972
A01	HLA-A*26:02	886	894	WTFGAGAAL	0.7617
A01	HLA-A*26:02	898	906	FAMQMAYRF	0.5082
A01	HLA-A*26:02	940	948	STASALGKL	0.6677
A01	HLA-A*26:02	962	970	LVKQLSSNF	0.536
A01	HLA-A*26:02	1054	1062	QSAPHGVVF	0.6027
A01	HLA-A*26:02	1095	1103	FVSNGTHWF	0.8644
A01	HLA-A*26:02	1113	1121	QIITTDNTF	0.6231
A01	HLA-A*26:02	1168	1176	DISGINASV	0.5184
A01	HLA-A*26:02	1188	1197	EVAKNLNESL	0.5001

A01	HLA-A*26:03	192	200	FVFKNIDGY	0.7319
A01	HLA-A*26:03	258	266	WTAGAAAYY	0.746
A01	HLA-A*26:04	28	37	YTNSFTRGVY	0.5132
A01	HLA-A*26:04	191	200	EFVFKNIDGY	0.6376
A01	HLA-A*26:04	192	200	FVFKNIDGY	0.853
A01	HLA-A*26:04	258	266	WTAGAAAYY	0.8788
A01	HLA-A*26:04	360	369	NCVADYSVLY	0.6391
A01	HLA-A*26:04	361	369	CVADYSVLY	0.75
A01	HLA-A*26:04	686	695	SVASQSIIAY	0.5849
A01	HLA-A*26:04	780	789	EVFAQVKQIY	0.6193
A01	HLA-A*26:05	28	37	YTNSFTRGVY	0.5354
A01	HLA-A*26:05	191	200	EFVFKNIDGY	0.7735
A01	HLA-A*26:05	192	200	FVFKNIDGY	0.8748
A01	HLA-A*26:05	258	266	WTAGAAAYY	0.9178
A01	HLA-A*26:05	340	351	EVFNATRFASVY	0.5592
A01	HLA-A*26:05	360	369	NCVADYSVLY	0.7308
A01	HLA-A*26:05	361	369	CVADYSVLY	0.7925
A01	HLA-A*26:05	603	612	NTSNQVAVLY	0.5637
A01	HLA-A*26:05	686	695	SVASQSIIAY	0.6565
A01	HLA-A*26:05	780	789	EVFAQVKQIY	0.7142
A01	HLA-A*26:06	192	200	FVFKNIDGY	0.7319
A01	HLA-A*26:06	258	266	WTAGAAAYY	0.746
A01	HLA-A*26:07	28	37	YTNSFTRGVY	0.5921
A01	HLA-A*26:07	50	58	STQDLFLPF	0.531
A01	HLA-A*26:07	192	200	FVFKNIDGY	0.929
A01	HLA-A*26:07	257	266	GWTAGAAAYY	0.6545
A01	HLA-A*26:07	258	266	WTAGAAAYY	0.8842
A01	HLA-A*26:07	261	269	GAAAYYVGY	0.7223
A01	HLA-A*26:07	360	369	NCVADYSVLY	0.557
A01	HLA-A*26:07	361	369	CVADYSVLY	0.7863
A01	HLA-A*26:07	366	374	SVLYNSASF	0.546
A01	HLA-A*26:07	686	695	SVASQSIIAY	0.7678
A01	HLA-A*26:07	691	699	SIIAYTMSL	0.5743
A01	HLA-A*26:07	698	707	SLGAENSVAY	0.5027
A01	HLA-A*26:07	718	726	FTISVTTEI	0.5245
A01	HLA-A*26:07	864	873	LLTDEMIAQY	0.554
A01	HLA-A*26:07	865	873	LTDEMIAQY	0.7389
A01	HLA-A*26:07	880	888	GTITSGWTF	0.6723
A01	HLA-A*26:07	976	984	VLNDILSRL	0.5945
A01	HLA-A*26:07	1095	1103	FVSNGTHWF	0.7015
A01	HLA-A*26:07	1196	1206	SLIDLQELGKY	0.516
A01	HLA-A*26:08	28	37	YTNSFTRGVY	0.5682
A01	HLA-A*26:08	50	58	STQDLFLPF	0.5601
A01	HLA-A*26:08	191	200	EFVFKNIDGY	0.6318
A01	HLA-A*26:08	192	200	FVFKNIDGY	0.8385
A01	HLA-A*26:08	258	266	WTAGAAAYY	0.9025
A01	HLA-A*26:08	340	351	EVFNATRFASVY	0.5328
A01	HLA-A*26:08	360	369	NCVADYSVLY	0.766
A01	HLA-A*26:08	361	369	CVADYSVLY	0.819
A01	HLA-A*26:08	603	612	NTSNQVAVLY	0.5863

A01	HLA-A*26:08	686	695	SVASQSIIAY	0.7121
A01	HLA-A*26:08	780	789	EVFAQVKQIY	0.596
A01	HLA-A*26:08	865	873	LTDEMIAQY	0.7329
A01	HLA-A*26:08	1095	1103	FVSNGTHWF	0.5998
A01	HLA-A*26:09	28	37	YTNSFTRGVY	0.6479
A01	HLA-A*26:09	191	200	EFVFKNIDGY	0.751
A01	HLA-A*26:09	192	200	FVFKNIDGY	0.9239
A01	HLA-A*26:09	257	266	GWTAGAAAYY	0.5288
A01	HLA-A*26:09	258	266	WTAGAAAYY	0.9326
A01	HLA-A*26:09	298	306	ETKCTLKSF	0.5067
A01	HLA-A*26:09	340	351	EVFNATRFASVY	0.6007
A01	HLA-A*26:09	360	369	NCVADYSVLY	0.7668
A01	HLA-A*26:09	361	369	CVADYSVLY	0.8649
A01	HLA-A*26:09	603	612	NTSNQVAVLY	0.611
A01	HLA-A*26:09	686	695	SVASQSIIAY	0.7378
A01	HLA-A*26:09	780	789	EVFAQVKQIY	0.7512
A01	HLA-A*26:09	1095	1103	FVSNGTHWF	0.6488
A01	HLA-A*26:10	28	37	YTNSFTRGVY	0.5472
A01	HLA-A*26:10	191	200	EFVFKNIDGY	0.8157
A01	HLA-A*26:10	192	200	FVFKNIDGY	0.9119
A01	HLA-A*26:10	258	266	WTAGAAAYY	0.9338
A01	HLA-A*26:10	340	351	EVFNATRFASVY	0.6393
A01	HLA-A*26:10	360	369	NCVADYSVLY	0.7886
A01	HLA-A*26:10	361	369	CVADYSVLY	0.8578
A01	HLA-A*26:10	603	612	NTSNQVAVLY	0.5793
A01	HLA-A*26:10	686	695	SVASQSIIAY	0.6831
A01	HLA-A*26:10	780	789	EVFAQVKQIY	0.7706
A01	HLA-A*26:10	1095	1103	FVSNGTHWF	0.5675
A01	HLA-A*26:12	191	200	EFVFKNIDGY	0.6509
A01	HLA-A*26:12	192	200	FVFKNIDGY	0.8495
A01	HLA-A*26:12	258	266	WTAGAAAYY	0.8859
A01	HLA-A*26:12	360	369	NCVADYSVLY	0.7498
A01	HLA-A*26:12	361	369	CVADYSVLY	0.7826
A01	HLA-A*26:12	603	612	NTSNQVAVLY	0.5023
A01	HLA-A*26:12	686	695	SVASQSIIAY	0.5107
A01	HLA-A*26:12	780	789	EVFAQVKQIY	0.5545
A01	HLA-A*26:12	1095	1103	FVSNGTHWF	0.5181
A01	HLA-A*26:13	28	37	YTNSFTRGVY	0.5142
A01	HLA-A*26:13	191	200	EFVFKNIDGY	0.7343
A01	HLA-A*26:13	192	200	FVFKNIDGY	0.8696
A01	HLA-A*26:13	258	266	WTAGAAAYY	0.9172
A01	HLA-A*26:13	340	351	EVFNATRFASVY	0.5729
A01	HLA-A*26:13	360	369	NCVADYSVLY	0.7292
A01	HLA-A*26:13	361	369	CVADYSVLY	0.794
A01	HLA-A*26:13	603	612	NTSNQVAVLY	0.561
A01	HLA-A*26:13	686	695	SVASQSIIAY	0.6258
A01	HLA-A*26:13	780	789	EVFAQVKQIY	0.7104
A01	HLA-A*26:13	886	894	WTFGAGAAL	0.5071
A01	HLA-A*26:13	1095	1103	FVSNGTHWF	0.5038
A01	HLA-A*26:14	28	37	YTNSFTRGVY	0.5472

A01	HLA-A*26:14	191	200	EFVFKNIDGY	0.8157
A01	HLA-A*26:14	192	200	FVFKNIDGY	0.9119
A01	HLA-A*26:14	258	266	WTAGAAAYY	0.9338
A01	HLA-A*26:14	340	351	EVFNATRFASVY	0.6393
A01	HLA-A*26:14	360	369	NCVADYSVLY	0.7886
A01	HLA-A*26:14	361	369	CVADYSVLY	0.8578
A01	HLA-A*26:14	603	612	NTSNQVAVLY	0.5793
A01	HLA-A*26:14	686	695	SVASQSIIAY	0.6831
A01	HLA-A*26:14	780	789	EVFAQVKQIY	0.7706
A01	HLA-A*26:14	1095	1103	FVSNGTHWF	0.5675
A01	HLA-A*26:15	28	37	YTNSFTRGVY	0.5472
A01	HLA-A*26:15	191	200	EFVFKNIDGY	0.8157
A01	HLA-A*26:15	192	200	FVFKNIDGY	0.9119
A01	HLA-A*26:15	258	266	WTAGAAAYY	0.9338
A01	HLA-A*26:15	340	351	EVFNATRFASVY	0.6393
A01	HLA-A*26:15	360	369	NCVADYSVLY	0.7886
A01	HLA-A*26:15	361	369	CVADYSVLY	0.8578
A01	HLA-A*26:15	603	612	NTSNQVAVLY	0.5793
A01	HLA-A*26:15	686	695	SVASQSIIAY	0.6831
A01	HLA-A*26:15	780	789	EVFAQVKQIY	0.7706
A01	HLA-A*26:15	1095	1103	FVSNGTHWF	0.5675
A01	HLA-A*26:17	28	37	YTNSFTRGVY	0.5472
A01	HLA-A*26:17	191	200	EFVFKNIDGY	0.8157
A01	HLA-A*26:17	192	200	FVFKNIDGY	0.9119
A01	HLA-A*26:17	258	266	WTAGAAAYY	0.9338
A01	HLA-A*26:17	340	351	EVFNATRFASVY	0.6393
A01	HLA-A*26:17	360	369	NCVADYSVLY	0.7886
A01	HLA-A*26:17	361	369	CVADYSVLY	0.8578
A01	HLA-A*26:17	603	612	NTSNQVAVLY	0.5793
A01	HLA-A*26:17	686	695	SVASQSIIAY	0.6831
A01	HLA-A*26:17	780	789	EVFAQVKQIY	0.7706
A01	HLA-A*26:17	1095	1103	FVSNGTHWF	0.5675
A01	HLA-A*26:18	191	200	EFVFKNIDGY	0.6509
A01	HLA-A*26:18	192	200	FVFKNIDGY	0.8495
A01	HLA-A*26:18	258	266	WTAGAAAYY	0.8859
A01	HLA-A*26:18	360	369	NCVADYSVLY	0.7498
A01	HLA-A*26:18	361	369	CVADYSVLY	0.7826
A01	HLA-A*26:18	603	612	NTSNQVAVLY	0.5023
A01	HLA-A*26:18	686	695	SVASQSIIAY	0.5107
A01	HLA-A*26:18	780	789	EVFAQVKQIY	0.5545
A01	HLA-A*26:18	1095	1103	FVSNGTHWF	0.5181
A01	HLA-A*26:19	28	37	YTNSFTRGVY	0.7228
A01	HLA-A*26:19	28	38	YTNSFTRGVYY	0.5399
A01	HLA-A*26:19	30	38	NSFTRGVYY	0.7407
A01	HLA-A*26:19	50	58	STQDLFLPF	0.6401
A01	HLA-A*26:19	161	170	SSANNCTFEY	0.5341
A01	HLA-A*26:19	162	170	SANNCTFEY	0.5571
A01	HLA-A*26:19	192	200	FVFKNIDGY	0.8978
A01	HLA-A*26:19	256	266	SGWTAGAAAYY	0.6687
A01	HLA-A*26:19	257	266	GWTAGAAAYY	0.8485

A01	HLA-A*26:19	258	266	WTAGAAAYY	0.9258
A01	HLA-A*26:19	261	269	GAAAYYVGY	0.8482
A01	HLA-A*26:19	359	369	SNCVADYSVLY	0.5535
A01	HLA-A*26:19	360	369	NCVADYSVLY	0.613
A01	HLA-A*26:19	361	369	CVADYSVLY	0.8311
A01	HLA-A*26:19	366	374	SVLYNSASF	0.5818
A01	HLA-A*26:19	372	380	ASFSTFKCY	0.5748
A01	HLA-A*26:19	603	612	NTSNQVAVLY	0.5876
A01	HLA-A*26:19	604	612	TSNQVAVLY	0.8069
A01	HLA-A*26:19	634	643	RVYSTGSNVF	0.5845
A01	HLA-A*26:19	686	695	SVASQSIIAY	0.8445
A01	HLA-A*26:19	687	695	VASQSIIAY	0.7283
A01	HLA-A*26:19	691	699	SIIAYTMSL	0.5489
A01	HLA-A*26:19	733	741	KTSVDCTMY	0.5057
A01	HLA-A*26:19	865	873	LTDEMIAQY	0.7578
A01	HLA-A*26:19	880	888	GTITSGWTF	0.7351
A01	HLA-A*26:19	940	948	STASALGKL	0.5468
A01	HLA-A*26:19	1054	1062	QSAPHGVVF	0.5969
A01	HLA-A*26:19	1059	1067	GVVFLHVTY	0.6507
A01	HLA-A*26:19	1095	1103	FVSNGTHWF	0.5261
A01	HLA-A*26:19	1128	1138	VVIGIVNNTVY	0.5268
A01	HLA-A*26:21	192	200	FVFKNIDGY	0.7319
A01	HLA-A*26:21	258	266	WTAGAAAYY	0.746
A01	HLA-A*26:23	28	37	YTNSFTRGVY	0.5472
A01	HLA-A*26:23	191	200	EFVFKNIDGY	0.8157
A01	HLA-A*26:23	192	200	FVFKNIDGY	0.9119
A01	HLA-A*26:23	258	266	WTAGAAAYY	0.9338
A01	HLA-A*26:23	340	351	EVFNATRFASVY	0.6393
A01	HLA-A*26:23	360	369	NCVADYSVLY	0.7886
A01	HLA-A*26:23	361	369	CVADYSVLY	0.8578
A01	HLA-A*26:23	603	612	NTSNQVAVLY	0.5793
A01	HLA-A*26:23	686	695	SVASQSIIAY	0.6831
A01	HLA-A*26:23	780	789	EVFAQVKQIY	0.7706
A01	HLA-A*26:23	1095	1103	FVSNGTHWF	0.5675
A01	HLA-A*26:24	28	37	YTNSFTRGVY	0.5472
A01	HLA-A*26:24	191	200	EFVFKNIDGY	0.8157
A01	HLA-A*26:24	192	200	FVFKNIDGY	0.9119
A01	HLA-A*26:24	258	266	WTAGAAAYY	0.9338
A01	HLA-A*26:24	340	351	EVFNATRFASVY	0.6393
A01	HLA-A*26:24	360	369	NCVADYSVLY	0.7886
A01	HLA-A*26:24	361	369	CVADYSVLY	0.8578
A01	HLA-A*26:24	603	612	NTSNQVAVLY	0.5793
A01	HLA-A*26:24	686	695	SVASQSIIAY	0.6831
A01	HLA-A*26:24	780	789	EVFAQVKQIY	0.7706
A01	HLA-A*26:24	1095	1103	FVSNGTHWF	0.5675
A01	HLA-A*26:26	28	37	YTNSFTRGVY	0.5472
A01	HLA-A*26:26	191	200	EFVFKNIDGY	0.8157
A01	HLA-A*26:26	192	200	FVFKNIDGY	0.9119
A01	HLA-A*26:26	258	266	WTAGAAAYY	0.9338
A01	HLA-A*26:26	340	351	EVFNATRFASVY	0.6393

A01	HLA-A*26:26	360	369	NCVADYSVLY	0.7886
A01	HLA-A*26:26	361	369	CVADYSVLY	0.8578
A01	HLA-A*26:26	603	612	NTSNQVAVLY	0.5793
A01	HLA-A*26:26	686	695	SVASQSIIAY	0.6831
A01	HLA-A*26:26	780	789	EVFAQVKQIY	0.7706
A01	HLA-A*26:26	1095	1103	FVSNGTHWF	0.5675
A01	HLA-A*30:02	28	37	YTNSFTRGVY	0.5478
A01	HLA-A*30:02	30	38	NSFTRGVYY	0.6841
A01	HLA-A*30:02	161	170	SSANNCTFEY	0.5282
A01	HLA-A*30:02	162	170	SANNCTFEY	0.5717
A01	HLA-A*30:02	192	200	FVFKNIDGY	0.5825
A01	HLA-A*30:02	195	204	KNIDGYFKIY	0.5462
A01	HLA-A*30:02	240	248	TLLALHRSY	0.5237
A01	HLA-A*30:02	257	266	GWTAGAAAYY	0.6876
A01	HLA-A*30:02	258	266	WTAGAAAYY	0.7568
A01	HLA-A*30:02	261	269	GAAAYYVGY	0.6441
A01	HLA-A*30:02	304	313	KSFTVEKGIY	0.5482
A01	HLA-A*30:02	357	365	RISNCVADY	0.5878
A01	HLA-A*30:02	361	369	CVADYSVLY	0.5824
A01	HLA-A*30:02	372	380	ASFSTFKCY	0.5884
A01	HLA-A*30:02	413	421	GQTGKIADY	0.581
A01	HLA-A*30:02	444	453	KVGGNYNYLY	0.7271
A01	HLA-A*30:02	445	453	VGGNYNYLY	0.5772
A01	HLA-A*30:02	496	505	GFQPTNGVGY	0.5657
A01	HLA-A*30:02	604	612	TSNQVAVLY	0.8094
A01	HLA-A*30:02	628	636	QLTPTWRVY	0.5588
A01	HLA-A*30:02	666	674	IGAGICASY	0.6063
A01	HLA-A*30:02	686	695	SVASQSIIAY	0.5074
A01	HLA-A*30:02	687	695	VASQSIIAY	0.6482
A01	HLA-A*30:02	733	741	KTSVDCTMY	0.6807
A01	HLA-A*30:02	781	789	VFAQVKQIY	0.6982
A01	HLA-A*30:02	865	873	LTDEMIAQY	0.69
A01	HLA-A*30:02	1039	1047	RVDFCGKGY	0.6059
A01	HLA-A*30:02	1264	1272	VLKGVKLHY	0.8117
A01	HLA-A*30:03	28	37	YTNSFTRGVY	0.5478
A01	HLA-A*30:03	30	38	NSFTRGVYY	0.6841
A01	HLA-A*30:03	161	170	SSANNCTFEY	0.5282
A01	HLA-A*30:03	162	170	SANNCTFEY	0.5717
A01	HLA-A*30:03	192	200	FVFKNIDGY	0.5825
A01	HLA-A*30:03	195	204	KNIDGYFKIY	0.5462
A01	HLA-A*30:03	240	248	TLLALHRSY	0.5237
A01	HLA-A*30:03	257	266	GWTAGAAAYY	0.6876
A01	HLA-A*30:03	258	266	WTAGAAAYY	0.7568
A01	HLA-A*30:03	261	269	GAAAYYVGY	0.6441
A01	HLA-A*30:03	304	313	KSFTVEKGIY	0.5482
A01	HLA-A*30:03	357	365	RISNCVADY	0.5878
A01	HLA-A*30:03	361	369	CVADYSVLY	0.5824
A01	HLA-A*30:03	372	380	ASFSTFKCY	0.5884
A01	HLA-A*30:03	413	421	GQTGKIADY	0.581
A01	HLA-A*30:03	444	453	KVGGNYNYLY	0.7271

A01	HLA-A*30:03	445	453	VGGNYNYLY	0.5772
A01	HLA-A*30:03	496	505	GFQPTNGVGY	0.5657
A01	HLA-A*30:03	604	612	TSNQVAVLY	0.8094
A01	HLA-A*30:03	628	636	QLTPTWRVY	0.5588
A01	HLA-A*30:03	666	674	IGAGICASY	0.6063
A01	HLA-A*30:03	686	695	SVASQSIIAY	0.5074
A01	HLA-A*30:03	687	695	VASQSIIAY	0.6482
A01	HLA-A*30:03	733	741	KTSVDCTMY	0.6807
A01	HLA-A*30:03	781	789	VFAQVKQIY	0.6982
A01	HLA-A*30:03	865	873	LTDEMIAQY	0.69
A01	HLA-A*30:03	1039	1047	RVDFCGKGY	0.6059
A01	HLA-A*30:03	1264	1272	VLKGVKLHY	0.8117
A01	HLA-A*30:04	257	266	GWTAGAAAYY	0.5598
A01	HLA-A*30:04	258	266	WTAGAAAYY	0.6721
A01	HLA-A*30:04	261	269	GAAAYYVGY	0.5257
A01	HLA-A*30:04	444	453	KVGGNYNYLY	0.5264
A01	HLA-A*30:04	445	453	VGGNYNYLY	0.512
A01	HLA-A*30:04	604	612	TSNQVAVLY	0.6629
A01	HLA-A*30:06	257	266	GWTAGAAAYY	0.5598
A01	HLA-A*30:06	258	266	WTAGAAAYY	0.6721
A01	HLA-A*30:06	261	269	GAAAYYVGY	0.5257
A01	HLA-A*30:06	444	453	KVGGNYNYLY	0.5264
A01	HLA-A*30:06	445	453	VGGNYNYLY	0.512
A01	HLA-A*30:06	604	612	TSNQVAVLY	0.6629
A01	HLA-A*30:09	30	38	NSFTRGVYY	0.5993
A01	HLA-A*30:09	162	170	SANNCTFEY	0.5586
A01	HLA-A*30:09	195	204	KNIDGYFKIY	0.5437
A01	HLA-A*30:09	257	266	GWTAGAAAYY	0.5637
A01	HLA-A*30:09	258	266	WTAGAAAYY	0.667
A01	HLA-A*30:09	261	269	GAAAYYVGY	0.6001
A01	HLA-A*30:09	361	369	CVADYSVLY	0.5285
A01	HLA-A*30:09	444	453	KVGGNYNYLY	0.6407
A01	HLA-A*30:09	445	453	VGGNYNYLY	0.6713
A01	HLA-A*30:09	604	612	TSNQVAVLY	0.7365
A01	HLA-A*30:09	666	674	IGAGICASY	0.5459
A01	HLA-A*30:09	733	741	KTSVDCTMY	0.5356
A01	HLA-A*30:09	781	789	VFAQVKQIY	0.6655
A01	HLA-A*30:09	865	873	LTDEMIAQY	0.6628
A01	HLA-A*30:12	28	37	YTNSFTRGVY	0.5478
A01	HLA-A*30:12	30	38	NSFTRGVYY	0.6841
A01	HLA-A*30:12	161	170	SSANNCTFEY	0.5282
A01	HLA-A*30:12	162	170	SANNCTFEY	0.5717
A01	HLA-A*30:12	192	200	FVFKNIDGY	0.5825
A01	HLA-A*30:12	195	204	KNIDGYFKIY	0.5462
A01	HLA-A*30:12	240	248	TLLALHRSY	0.5237
A01	HLA-A*30:12	257	266	GWTAGAAAYY	0.6876
A01	HLA-A*30:12	258	266	WTAGAAAYY	0.7568
A01	HLA-A*30:12	261	269	GAAAYYVGY	0.6441
A01	HLA-A*30:12	304	313	KSFTVEKGIY	0.5482
A01	HLA-A*30:12	357	365	RISNCVADY	0.5878

A01	HLA-A*30:12	361	369	CVADYSVLY	0.5824
A01	HLA-A*30:12	372	380	ASFSTFKCY	0.5884
A01	HLA-A*30:12	413	421	GQTGKIADY	0.581
A01	HLA-A*30:12	444	453	KVGGNYNYLY	0.7271
A01	HLA-A*30:12	445	453	VGGNYNYLY	0.5772
A01	HLA-A*30:12	496	505	GFQPTNGVGY	0.5657
A01	HLA-A*30:12	604	612	TSNQVAVLY	0.8094
A01	HLA-A*30:12	628	636	QLTPTWRVY	0.5588
A01	HLA-A*30:12	666	674	IGAGICASY	0.6063
A01	HLA-A*30:12	686	695	SVASQSIIAY	0.5074
A01	HLA-A*30:12	687	695	VASQSIIAY	0.6482
A01	HLA-A*30:12	733	741	KTSVDCTMY	0.6807
A01	HLA-A*30:12	781	789	VFAQVKQIY	0.6982
A01	HLA-A*30:12	865	873	LTDEMIAQY	0.69
A01	HLA-A*30:12	1039	1047	RVDFCGKGY	0.6059
A01	HLA-A*30:12	1264	1272	VLKGVKLHY	0.8117
A01	HLA-A*32:01	366	374	SVLYNSASF	0.6299
A01	HLA-A*32:01	417	425	KIADYNYKL	0.8445
A01	HLA-A*32:01	634	643	RVYSTGSNVF	0.6705
A01	HLA-A*32:01	691	699	SIIAYTMSL	0.7021
A01	HLA-A*32:01	815	823	RSFIEDLLF	0.5863
A01	HLA-A*32:01	880	888	GTITSGWTF	0.7522
A01	HLA-A*32:01	1185	1193	RLNEVAKNL	0.7116
A01	HLA-A*32:02	366	374	SVLYNSASF	0.5561
A01	HLA-A*32:02	417	425	KIADYNYKL	0.7698
A01	HLA-A*32:02	634	643	RVYSTGSNVF	0.5403
A01	HLA-A*32:02	691	699	SIIAYTMSL	0.62
A01	HLA-A*32:02	880	888	GTITSGWTF	0.6422
A01	HLA-A*32:02	1185	1193	RLNEVAKNL	0.6529
A01	HLA-A*32:05	366	374	SVLYNSASF	0.6066
A01	HLA-A*32:05	417	425	KIADYNYKL	0.7528
A01	HLA-A*32:05	634	643	RVYSTGSNVF	0.5466
A01	HLA-A*32:05	691	699	SIIAYTMSL	0.6692
A01	HLA-A*32:05	815	823	RSFIEDLLF	0.5277
A01	HLA-A*32:05	880	888	GTITSGWTF	0.7114
A01	HLA-A*32:05	1185	1193	RLNEVAKNL	0.5942
A01	HLA-A*32:06	366	374	SVLYNSASF	0.6299
A01	HLA-A*32:06	417	425	KIADYNYKL	0.8445
A01	HLA-A*32:06	634	643	RVYSTGSNVF	0.6705
A01	HLA-A*32:06	691	699	SIIAYTMSL	0.7021
A01	HLA-A*32:06	815	823	RSFIEDLLF	0.5863
A01	HLA-A*32:06	880	888	GTITSGWTF	0.7522
A01	HLA-A*32:06	1185	1193	RLNEVAKNL	0.7116
A01	HLA-A*32:07	50	58	STQDLFLPF	0.5832
A01	HLA-A*32:07	77	86	KRFDNPVLPF	0.6519
A01	HLA-A*32:07	78	86	RFDNPVLPF	0.7048
A01	HLA-A*32:07	144	152	YYHKNNKSW	0.6197
A01	HLA-A*32:07	193	201	VFKNIDGYF	0.6354
A01	HLA-A*32:07	195	203	KNIDGYFKI	0.501
A01	HLA-A*32:07	261	269	GAAAYYVGY	0.5142

A01	HLA-A*32:07	267	275	VGYLQPRTF	0.5924
A01	HLA-A*32:07	268	276	GYLQPRTFL	0.5759
A01	HLA-A*32:07	310	318	KGIYQTSNF	0.5492
A01	HLA-A*32:07	345	353	TRFASVYAW	0.7472
A01	HLA-A*32:07	366	374	SVLYNSASF	0.7088
A01	HLA-A*32:07	417	425	KIADYNYKL	0.6173
A01	HLA-A*32:07	557	565	KKFLPFQQF	0.6339
A01	HLA-A*32:07	634	643	RVYSTGSNVF	0.6399
A01	HLA-A*32:07	635	643	VYSTGSNVF	0.566
A01	HLA-A*32:07	689	697	SQSIIAYTM	0.5018
A01	HLA-A*32:07	710	718	NSIAIPTNF	0.6477
A01	HLA-A*32:07	781	789	VFAQVKQIY	0.715
A01	HLA-A*32:07	815	823	RSFIEDLLF	0.684
A01	HLA-A*32:07	880	888	GTITSGWTF	0.8141
A01	HLA-A*32:07	1054	1062	QSAPHGVVF	0.6406
A01	HLA-A*32:07	1101	1109	HWFVTQRNF	0.7546
A01	HLA-A*32:07	1208	1216	QYIKWPWYI	0.6932
A01	HLA-A*32:09	50	58	STQDLFLPF	0.5147
A01	HLA-A*32:09	366	374	SVLYNSASF	0.6078
A01	HLA-A*32:09	417	425	KIADYNYKL	0.6951
A01	HLA-A*32:09	691	699	SIIAYTMSL	0.552
A01	HLA-A*32:09	815	823	RSFIEDLLF	0.5187
A01	HLA-A*32:09	880	888	GTITSGWTF	0.7219
A01	HLA-A*32:10	634	643	RVYSTGSNVF	0.5972
A01	HLA-A*36:01	28	37	YTNSFTRGVY	0.7119
A01	HLA-A*36:01	28	38	YTNSFTRGVYY	0.7867
A01	HLA-A*36:01	160	170	YSSANNCTFEY	0.5391
A01	HLA-A*36:01	161	170	SSANNCTFEY	0.7073
A01	HLA-A*36:01	162	170	SANNCTFEY	0.6682
A01	HLA-A*36:01	256	266	SGWTAGAAAYY	0.667
A01	HLA-A*36:01	257	266	GWTAGAAAYY	0.7231
A01	HLA-A*36:01	258	266	WTAGAAAYY	0.8964
A01	HLA-A*36:01	358	369	ISNCVADYSVLY	0.6026
A01	HLA-A*36:01	361	369	CVADYSVLY	0.7473
A01	HLA-A*36:01	362	369	VADYSVLY	0.65
A01	HLA-A*36:01	414	423	QTGKIADYNY	0.5523
A01	HLA-A*36:01	601	612	GTNTSNQVAVLY	0.629
A01	HLA-A*36:01	603	612	NTSNQVAVLY	0.7051
A01	HLA-A*36:01	604	612	TSNQVAVLY	0.8841
A01	HLA-A*36:01	733	741	KTSVDCTMY	0.7042
A01	HLA-A*36:01	746	756	STECSNLLLQY	0.6614
A01	HLA-A*36:01	828	837	LADAGFIKQY	0.7384
A01	HLA-A*36:01	863	873	PLLTDEMIAQY	0.5018
A01	HLA-A*36:01	865	873	LTDEMIAQY	0.9845
A01	HLA-A*36:01	1039	1047	RVDFCGKGY	0.8092
A01	HLA-A*36:01	1197	1206	LIDLQELGKY	0.7101
A01	HLA-A*36:02	28	37	YTNSFTRGVY	0.5893
A01	HLA-A*36:02	28	38	YTNSFTRGVYY	0.6541
A01	HLA-A*36:02	161	170	SSANNCTFEY	0.6078
A01	HLA-A*36:02	162	170	SANNCTFEY	0.605

A01	HLA-A*36:02	256	266	SGWTAGAAAYY	0.583
A01	HLA-A*36:02	257	266	GWTAGAAAYY	0.6743
A01	HLA-A*36:02	258	266	WTAGAAAYY	0.8378
A01	HLA-A*36:02	361	369	CVADYSVLY	0.6943
A01	HLA-A*36:02	444	453	KVGGNYNYLY	0.5293
A01	HLA-A*36:02	601	612	GTNTSNQVAVLY	0.5171
A01	HLA-A*36:02	603	612	NTSNQVAVLY	0.5926
A01	HLA-A*36:02	604	612	TSNQVAVLY	0.8154
A01	HLA-A*36:02	733	741	KTSVDCTMY	0.6327
A01	HLA-A*36:02	865	873	LTDEMIAQY	0.95
A01	HLA-A*36:02	1039	1047	RVDFCGKGY	0.7404
A01	HLA-A*36:02	1197	1206	LIDLQELGKY	0.5242
A01	HLA-A*36:04	28	37	YTNSFTRGVY	0.7271
A01	HLA-A*36:04	28	38	YTNSFTRGVYY	0.8143
A01	HLA-A*36:04	30	38	NSFTRGVYY	0.6993
A01	HLA-A*36:04	160	170	YSSANNCTFEY	0.6118
A01	HLA-A*36:04	161	170	SSANNCTFEY	0.727
A01	HLA-A*36:04	162	170	SANNCTFEY	0.6738
A01	HLA-A*36:04	256	266	SGWTAGAAAYY	0.709
A01	HLA-A*36:04	257	266	GWTAGAAAYY	0.7396
A01	HLA-A*36:04	258	266	WTAGAAAYY	0.9128
A01	HLA-A*36:04	358	369	ISNCVADYSVLY	0.652
A01	HLA-A*36:04	361	369	CVADYSVLY	0.7656
A01	HLA-A*36:04	362	369	VADYSVLY	0.6977
A01	HLA-A*36:04	414	423	QTGKIADYNY	0.5768
A01	HLA-A*36:04	601	612	GTNTSNQVAVLY	0.6316
A01	HLA-A*36:04	603	612	NTSNQVAVLY	0.7383
A01	HLA-A*36:04	604	612	TSNQVAVLY	0.9007
A01	HLA-A*36:04	733	741	KTSVDCTMY	0.64
A01	HLA-A*36:04	746	756	STECSNLLLQY	0.7554
A01	HLA-A*36:04	828	837	LADAGFIKQY	0.7998
A01	HLA-A*36:04	863	873	PLLTDEMIAQY	0.6402
A01	HLA-A*36:04	865	873	LTDEMIAQY	0.9936
A01	HLA-A*36:04	1039	1047	RVDFCGKGY	0.7674
A01	HLA-A*36:04	1197	1206	LIDLQELGKY	0.7682
A01 A03	HLA-A*30:01	89	97	GVYFASTEK	0.524
A01 A03	HLA-A*30:01	142	150	GVYYHKNNK	0.5185
A01 A03	HLA-A*30:01	302	310	TLKSFTVEK	0.5401
A01 A03	HLA-A*30:01	344	352	ATRFASVYA	0.8043
A01 A03	HLA-A*30:01	378	386	KCYGVSPTK	0.5092
A01 A03	HLA-A*30:01	454	462	RLFRKSNLK	0.5644
A01 A03	HLA-A*30:01	634	642	RVYSTGSNV	0.632
A01 A03	HLA-A*30:01	1065	1073	VTYVPAQEK	0.5221
A01 A03	HLA-A*30:08	344	352	ATRFASVYA	0.6186
A01 A03	HLA-A*30:11	89	97	GVYFASTEK	0.524
A01 A03	HLA-A*30:11	142	150	GVYYHKNNK	0.5185
A01 A03	HLA-A*30:11	302	310	TLKSFTVEK	0.5401
A01 A03	HLA-A*30:11	344	352	ATRFASVYA	0.8043
A01 A03	HLA-A*30:11	378	386	KCYGVSPTK	0.5092
A01 A03	HLA-A*30:11	454	462	RLFRKSNLK	0.5644

A01 A03	HLA-A*30:11	634	642	RVYSTGSNV	0.632
A01 A03	HLA-A*30:11	1065	1073	VTYVPAQEK	0.5221
A01 A03	HLA-A*30:15	89	97	GVYFASTEK	0.524
A01 A03	HLA-A*30:15	142	150	GVYYHKNNK	0.5185
A01 A03	HLA-A*30:15	302	310	TLKSFTVEK	0.5401
A01 A03	HLA-A*30:15	344	352	ATRFASVYA	0.8043
A01 A03	HLA-A*30:15	378	386	KCYGVSPTK	0.5092
A01 A03	HLA-A*30:15	454	462	RLFRKSNLK	0.5644
A01 A03	HLA-A*30:15	634	642	RVYSTGSNV	0.632
A01 A03	HLA-A*30:15	1065	1073	VTYVPAQEK	0.5221
A01 A24	HLA-A*29:01	30	38	NSFTRGVYY	0.825
A01 A24	HLA-A*29:01	151	160	SWMESEFRVY	0.7114
A01 A24	HLA-A*29:01	162	170	SANNCTFEY	0.7814
A01 A24	HLA-A*29:01	192	200	FVFKNIDGY	0.9181
A01 A24	HLA-A*29:01	240	248	TLLALHRSY	0.6806
A01 A24	HLA-A*29:01	257	265	GWTAGAAAY	0.5826
A01 A24	HLA-A*29:01	257	266	GWTAGAAAYY	0.6803
A01 A24	HLA-A*29:01	258	266	WTAGAAAYY	0.9403
A01 A24	HLA-A*29:01	261	269	GAAAYYVGY	0.7714
A01 A24	HLA-A*29:01	360	369	NCVADYSVLY	0.6347
A01 A24	HLA-A*29:01	361	369	CVADYSVLY	0.9086
A01 A24	HLA-A*29:01	444	453	KVGGNYNYLY	0.5931
A01 A24	HLA-A*29:01	445	453	VGGNYNYLY	0.6436
A01 A24	HLA-A*29:01	496	505	GFQPTNGVGY	0.6858
A01 A24	HLA-A*29:01	604	612	TSNQVAVLY	0.8854
A01 A24	HLA-A*29:01	628	636	QLTPTWRVY	0.51
A01 A24	HLA-A*29:01	686	695	SVASQSIIAY	0.6064
A01 A24	HLA-A*29:01	687	695	VASQSIIAY	0.6791
A01 A24	HLA-A*29:01	781	789	VFAQVKQIY	0.9197
A01 A24	HLA-A*29:01	827	837	TLADAGFIKQY	0.5983
A01 A24	HLA-A*29:01	865	873	LTDEMIAQY	0.819
A01 A24	HLA-A*29:01	896	904	IPFAMQMAY	0.5343
A01 A24	HLA-A*29:01	909	917	IGVTQNVLY	0.5165
A01 A24	HLA-A*29:01	1059	1067	GVVFLHVTY	0.7218
A01 A24	HLA-A*29:01	1102	1110	WFVTQRNFY	0.7439
A01 A24	HLA-A*29:01	1147	1155	SFKEELDKY	0.6923
A01 A24	HLA-A*29:01	1264	1272	VLKGVKLHY	0.8598
A01 A24	HLA-A*29:02	30	38	NSFTRGVYY	0.825
A01 A24	HLA-A*29:02	151	160	SWMESEFRVY	0.7114
A01 A24	HLA-A*29:02	162	170	SANNCTFEY	0.7814
A01 A24	HLA-A*29:02	192	200	FVFKNIDGY	0.9181
A01 A24	HLA-A*29:02	240	248	TLLALHRSY	0.6806
A01 A24	HLA-A*29:02	257	265	GWTAGAAAY	0.5826
A01 A24	HLA-A*29:02	257	266	GWTAGAAAYY	0.6803
A01 A24	HLA-A*29:02	258	266	WTAGAAAYY	0.9403
A01 A24	HLA-A*29:02	261	269	GAAAYYVGY	0.7714
A01 A24	HLA-A*29:02	360	369	NCVADYSVLY	0.6347
A01 A24	HLA-A*29:02	361	369	CVADYSVLY	0.9086
A01 A24	HLA-A*29:02	444	453	KVGGNYNYLY	0.5931
A01 A24	HLA-A*29:02	445	453	VGGNYNYLY	0.6436

A01 A24	HLA-A*29:02	496	505	GFQPTNGVGY	0.6858
A01 A24	HLA-A*29:02	604	612	TSNQVAVLY	0.8854
A01 A24	HLA-A*29:02	628	636	QLTPTWRVY	0.51
A01 A24	HLA-A*29:02	686	695	SVASQSIIAY	0.6064
A01 A24	HLA-A*29:02	687	695	VASQSIIAY	0.6791
A01 A24	HLA-A*29:02	781	789	VFAQVKQIY	0.9197
A01 A24	HLA-A*29:02	827	837	TLADAGFIKQY	0.5983
A01 A24	HLA-A*29:02	865	873	LTDEMIAQY	0.819
A01 A24	HLA-A*29:02	896	904	IPFAMQMAY	0.5343
A01 A24	HLA-A*29:02	909	917	IGVTQNVLY	0.5165
A01 A24	HLA-A*29:02	1059	1067	GVVFLHVTY	0.7218
A01 A24	HLA-A*29:02	1102	1110	WFVTQRNFY	0.7439
A01 A24	HLA-A*29:02	1147	1155	SFKEELDKY	0.6923
A01 A24	HLA-A*29:02	1264	1272	VLKGVKLHY	0.8598
A01 A24	HLA-A*29:03	30	38	NSFTRGVYY	0.7608
A01 A24	HLA-A*29:03	151	160	SWMESEFRVY	0.5544
A01 A24	HLA-A*29:03	152	160	WMESEFRVY	0.5108
A01 A24	HLA-A*29:03	162	170	SANNCTFEY	0.6647
A01 A24	HLA-A*29:03	192	200	FVFKNIDGY	0.814
A01 A24	HLA-A*29:03	240	248	TLLALHRSY	0.5915
A01 A24	HLA-A*29:03	258	266	WTAGAAAYY	0.863
A01 A24	HLA-A*29:03	261	269	GAAAYYVGY	0.648
A01 A24	HLA-A*29:03	360	369	NCVADYSVLY	0.6374
A01 A24	HLA-A*29:03	361	369	CVADYSVLY	0.8638
A01 A24	HLA-A*29:03	445	453	VGGNYNYLY	0.5077
A01 A24	HLA-A*29:03	603	612	NTSNQVAVLY	0.5268
A01 A24	HLA-A*29:03	604	612	TSNQVAVLY	0.8418
A01 A24	HLA-A*29:03	781	789	VFAQVKQIY	0.8384
A01 A24	HLA-A*29:03	865	873	LTDEMIAQY	0.7807
A01 A24	HLA-A*29:03	1102	1110	WFVTQRNFY	0.612
A01 A24	HLA-A*29:05	30	38	NSFTRGVYY	0.6683
A01 A24	HLA-A*29:05	192	200	FVFKNIDGY	0.6624
A01 A24	HLA-A*29:05	240	248	TLLALHRSY	0.5807
A01 A24	HLA-A*29:05	257	266	GWTAGAAAYY	0.595
A01 A24	HLA-A*29:05	258	266	WTAGAAAYY	0.7752
A01 A24	HLA-A*29:05	361	369	CVADYSVLY	0.6495
A01 A24	HLA-A*29:05	496	505	GFQPTNGVGY	0.6148
A01 A24	HLA-A*29:05	604	612	TSNQVAVLY	0.6551
A01 A24	HLA-A*29:05	686	695	SVASQSIIAY	0.547
A01 A24	HLA-A*29:05	781	789	VFAQVKQIY	0.7974
A01 A24	HLA-A*29:05	1102	1110	WFVTQRNFY	0.5904
A01 A24	HLA-A*29:05	1264	1272	VLKGVKLHY	0.8571
A01 A24	HLA-A*29:06	30	38	NSFTRGVYY	0.825
A01 A24	HLA-A*29:06	151	160	SWMESEFRVY	0.7114
A01 A24	HLA-A*29:06	162	170	SANNCTFEY	0.7814
A01 A24	HLA-A*29:06	192	200	FVFKNIDGY	0.9181
A01 A24	HLA-A*29:06	240	248	TLLALHRSY	0.6806
A01 A24	HLA-A*29:06	257	265	GWTAGAAAY	0.5826
A01 A24	HLA-A*29:06	257	266	GWTAGAAAYY	0.6803
A01 A24	HLA-A*29:06	258	266	WTAGAAAYY	0.9403

A01 A24	HLA-A*29:06	261	269	GAAAYYVGY	0.7714
A01 A24	HLA-A*29:06	360	369	NCVADYSVLY	0.6347
A01 A24	HLA-A*29:06	361	369	CVADYSVLY	0.9086
A01 A24	HLA-A*29:06	444	453	KVGGNYNYLY	0.5931
A01 A24	HLA-A*29:06	445	453	VGGNYNYLY	0.6436
A01 A24	HLA-A*29:06	496	505	GFQPTNGVGY	0.6858
A01 A24	HLA-A*29:06	604	612	TSNQVAVLY	0.8854
A01 A24	HLA-A*29:06	628	636	QLTPTWRVY	0.51
A01 A24	HLA-A*29:06	686	695	SVASQSIIAY	0.6064
A01 A24	HLA-A*29:06	687	695	VASQSIIAY	0.6791
A01 A24	HLA-A*29:06	781	789	VFAQVKQIY	0.9197
A01 A24	HLA-A*29:06	827	837	TLADAGFIKQY	0.5983
A01 A24	HLA-A*29:06	865	873	LTDEMIAQY	0.819
A01 A24	HLA-A*29:06	896	904	IPFAMQMAY	0.5343
A01 A24	HLA-A*29:06	909	917	IGVTQNVLY	0.5165
A01 A24	HLA-A*29:06	1059	1067	GVVFLHVTY	0.7218
A01 A24	HLA-A*29:06	1102	1110	WFVTQRNFY	0.7439
A01 A24	HLA-A*29:06	1147	1155	SFKEELDKY	0.6923
A01 A24	HLA-A*29:06	1264	1272	VLKGVKLHY	0.8598
A01 A24	HLA-A*29:09	30	38	NSFTRGVYY	0.825
A01 A24	HLA-A*29:09	151	160	SWMESEFRVY	0.7114
A01 A24	HLA-A*29:09	162	170	SANNCTFEY	0.7814
A01 A24	HLA-A*29:09	192	200	FVFKNIDGY	0.9181
A01 A24	HLA-A*29:09	240	248	TLLALHRSY	0.6806
A01 A24	HLA-A*29:09	257	265	GWTAGAAAY	0.5826
A01 A24	HLA-A*29:09	257	266	GWTAGAAAYY	0.6803
A01 A24	HLA-A*29:09	258	266	WTAGAAAYY	0.9403
A01 A24	HLA-A*29:09	261	269	GAAAYYVGY	0.7714
A01 A24	HLA-A*29:09	360	369	NCVADYSVLY	0.6347
A01 A24	HLA-A*29:09	361	369	CVADYSVLY	0.9086
A01 A24	HLA-A*29:09	444	453	KVGGNYNYLY	0.5931
A01 A24	HLA-A*29:09	445	453	VGGNYNYLY	0.6436
A01 A24	HLA-A*29:09	496	505	GFQPTNGVGY	0.6858
A01 A24	HLA-A*29:09	604	612	TSNQVAVLY	0.8854
A01 A24	HLA-A*29:09	628	636	QLTPTWRVY	0.51
A01 A24	HLA-A*29:09	686	695	SVASQSIIAY	0.6064
A01 A24	HLA-A*29:09	687	695	VASQSIIAY	0.6791
A01 A24	HLA-A*29:09	781	789	VFAQVKQIY	0.9197
A01 A24	HLA-A*29:09	827	837	TLADAGFIKQY	0.5983
A01 A24	HLA-A*29:09	865	873	LTDEMIAQY	0.819
A01 A24	HLA-A*29:09	896	904	IPFAMQMAY	0.5343
A01 A24	HLA-A*29:09	909	917	IGVTQNVLY	0.5165
A01 A24	HLA-A*29:09	1059	1067	GVVFLHVTY	0.7218
A01 A24	HLA-A*29:09	1102	1110	WFVTQRNFY	0.7439
A01 A24	HLA-A*29:09	1147	1155	SFKEELDKY	0.6923
A01 A24	HLA-A*29:09	1264	1272	VLKGVKLHY	0.8598
A01 A24	HLA-A*29:10	30	38	NSFTRGVYY	0.825
A01 A24	HLA-A*29:10	151	160	SWMESEFRVY	0.7114
A01 A24	HLA-A*29:10	162	170	SANNCTFEY	0.7814
A01 A24	HLA-A*29:10	192	200	FVFKNIDGY	0.9181

A01 A24	HLA-A*29:10	240	248	TLLALHRSY	0.6806
A01 A24	HLA-A*29:10	257	265	GWTAGAAAY	0.5826
A01 A24	HLA-A*29:10	257	266	GWTAGAAAYY	0.6803
A01 A24	HLA-A*29:10	258	266	WTAGAAAYY	0.9403
A01 A24	HLA-A*29:10	261	269	GAAAYYVGY	0.7714
A01 A24	HLA-A*29:10	360	369	NCVADYSVLY	0.6347
A01 A24	HLA-A*29:10	361	369	CVADYSVLY	0.9086
A01 A24	HLA-A*29:10	444	453	KVGGNYNYLY	0.5931
A01 A24	HLA-A*29:10	445	453	VGGNYNYLY	0.6436
A01 A24	HLA-A*29:10	496	505	GFQPTNGVGY	0.6858
A01 A24	HLA-A*29:10	604	612	TSNQVAVLY	0.8854
A01 A24	HLA-A*29:10	628	636	QLTPTWRVY	0.51
A01 A24	HLA-A*29:10	686	695	SVASQSIIAY	0.6064
A01 A24	HLA-A*29:10	687	695	VASQSIIAY	0.6791
A01 A24	HLA-A*29:10	781	789	VFAQVKQIY	0.9197
A01 A24	HLA-A*29:10	827	837	TLADAGFIKQY	0.5983
A01 A24	HLA-A*29:10	865	873	LTDEMIAQY	0.819
A01 A24	HLA-A*29:10	896	904	IPFAMQMAY	0.5343
A01 A24	HLA-A*29:10	909	917	IGVTQNVLY	0.5165
A01 A24	HLA-A*29:10	1059	1067	GVVFLHVTY	0.7218
A01 A24	HLA-A*29:10	1102	1110	WFVTQRNFY	0.7439
A01 A24	HLA-A*29:10	1147	1155	SFKEELDKY	0.6923
A01 A24	HLA-A*29:10	1264	1272	VLKGVKLHY	0.8598
A01 A24	HLA-A*29:11	30	38	NSFTRGVYY	0.825
A01 A24	HLA-A*29:11	151	160	SWMESEFRVY	0.7114
A01 A24	HLA-A*29:11	162	170	SANNCTFEY	0.7814
A01 A24	HLA-A*29:11	192	200	FVFKNIDGY	0.9181
A01 A24	HLA-A*29:11	240	248	TLLALHRSY	0.6806
A01 A24	HLA-A*29:11	257	265	GWTAGAAAY	0.5826
A01 A24	HLA-A*29:11	257	266	GWTAGAAAYY	0.6803
A01 A24	HLA-A*29:11	258	266	WTAGAAAYY	0.9403
A01 A24	HLA-A*29:11	261	269	GAAAYYVGY	0.7714
A01 A24	HLA-A*29:11	360	369	NCVADYSVLY	0.6347
A01 A24	HLA-A*29:11	361	369	CVADYSVLY	0.9086
A01 A24	HLA-A*29:11	444	453	KVGGNYNYLY	0.5931
A01 A24	HLA-A*29:11	445	453	VGGNYNYLY	0.6436
A01 A24	HLA-A*29:11	496	505	GFQPTNGVGY	0.6858
A01 A24	HLA-A*29:11	604	612	TSNQVAVLY	0.8854
A01 A24	HLA-A*29:11	628	636	QLTPTWRVY	0.51
A01 A24	HLA-A*29:11	686	695	SVASQSIIAY	0.6064
A01 A24	HLA-A*29:11	687	695	VASQSIIAY	0.6791
A01 A24	HLA-A*29:11	781	789	VFAQVKQIY	0.9197
A01 A24	HLA-A*29:11	827	837	TLADAGFIKQY	0.5983
A01 A24	HLA-A*29:11	865	873	LTDEMIAQY	0.819
A01 A24	HLA-A*29:11	896	904	IPFAMQMAY	0.5343
A01 A24	HLA-A*29:11	909	917	IGVTQNVLY	0.5165
A01 A24	HLA-A*29:11	1059	1067	GVVFLHVTY	0.7218
A01 A24	HLA-A*29:11	1102	1110	WFVTQRNFY	0.7439
A01 A24	HLA-A*29:11	1147	1155	SFKEELDKY	0.6923
A01 A24	HLA-A*29:11	1264	1272	VLKGVKLHY	0.8598

A01 A24	HLA-A*29:12	30	38	NSFTRGVYY	0.825
A01 A24	HLA-A*29:12	151	160	SWMESEFRVY	0.7114
A01 A24	HLA-A*29:12	162	170	SANNCTFEY	0.7814
A01 A24	HLA-A*29:12	192	200	FVFKNIDGY	0.9181
A01 A24	HLA-A*29:12	240	248	TLLALHRSY	0.6806
A01 A24	HLA-A*29:12	257	265	GWTAGAAAY	0.5826
A01 A24	HLA-A*29:12	257	266	GWTAGAAAYY	0.6803
A01 A24	HLA-A*29:12	258	266	WTAGAAAYY	0.9403
A01 A24	HLA-A*29:12	261	269	GAAAYYVGY	0.7714
A01 A24	HLA-A*29:12	360	369	NCVADYSVLY	0.6347
A01 A24	HLA-A*29:12	361	369	CVADYSVLY	0.9086
A01 A24	HLA-A*29:12	444	453	KVGGNYNYLY	0.5931
A01 A24	HLA-A*29:12	445	453	VGGNYNYLY	0.6436
A01 A24	HLA-A*29:12	496	505	GFQPTNGVGY	0.6858
A01 A24	HLA-A*29:12	604	612	TSNQVAVLY	0.8854
A01 A24	HLA-A*29:12	628	636	QLTPTWRVY	0.51
A01 A24	HLA-A*29:12	686	695	SVASQSIIAY	0.6064
A01 A24	HLA-A*29:12	687	695	VASQSIIAY	0.6791
A01 A24	HLA-A*29:12	781	789	VFAQVKQIY	0.9197
A01 A24	HLA-A*29:12	827	837	TLADAGFIKQY	0.5983
A01 A24	HLA-A*29:12	865	873	LTDEMIAQY	0.819
A01 A24	HLA-A*29:12	896	904	IPFAMQMAY	0.5343
A01 A24	HLA-A*29:12	909	917	IGVTQNVLY	0.5165
A01 A24	HLA-A*29:12	1059	1067	GVVFLHVTY	0.7218
A01 A24	HLA-A*29:12	1102	1110	WFVTQRNFY	0.7439
A01 A24	HLA-A*29:12	1147	1155	SFKEELDKY	0.6923
A01 A24	HLA-A*29:12	1264	1272	VLKGVKLHY	0.8598
A02	HLA-A*02:01	109	117	TLDSKTQSL	0.6782
A02	HLA-A*02:01	133	141	FQFCNDPFL	0.518
A02	HLA-A*02:01	269	277	YLQPRTFLL	0.973
A02	HLA-A*02:01	386	395	KLNDLCFTNV	0.7108
A02	HLA-A*02:01	417	425	KIADYNYKL	0.909
A02	HLA-A*02:01	424	433	KLPDDFTGCV	0.5916
A02	HLA-A*02:01	515	524	FELLHAPATV	0.6033
A02	HLA-A*02:01	610	620	VLYQDVNCTEV	0.5092
A02	HLA-A*02:01	691	699	SIIAYTMSL	0.7995
A02	HLA-A*02:01	718	726	FTISVTTEI	0.5257
A02	HLA-A*02:01	821	829	LLFNKVTLA	0.7857
A02	HLA-A*02:01	857	865	GLTVLPPLL	0.6786
A02	HLA-A*02:01	915	923	VLYENQKLI	0.5378
A02	HLA-A*02:01	975	984	SVLNDILSRL	0.5124
A02	HLA-A*02:01	976	984	VLNDILSRL	0.9507
A02	HLA-A*02:01	983	991	RLDKVEAEV	0.8609
A02	HLA-A*02:01	1000	1008	RLQSLQTYV	0.7431
A02	HLA-A*02:01	1047	1056	YHLMSFPQSA	0.5452
A02	HLA-A*02:01	1048	1056	HLMSFPQSA	0.7385
A02	HLA-A*02:01	1060	1068	VVFLHVTYV	0.6948
A02	HLA-A*02:01	1185	1193	RLNEVAKNL	0.6189
A02	HLA-A*02:01	1192	1200	NLNESLIDL	0.6972
A02	HLA-A*02:01	1220	1228	FIAGLIAIV	0.8207

A02	HLA-A*02:02	109	117	TLDSKTQSL	0.825
A02	HLA-A*02:02	133	141	FQFCNDPFL	0.5968
A02	HLA-A*02:02	269	277	YLQPRTFLL	0.9773
A02	HLA-A*02:02	386	395	KLNDLCFTNV	0.8261
A02	HLA-A*02:02	416	425	GKIADYNYKL	0.6474
A02	HLA-A*02:02	417	425	KIADYNYKL	0.9705
A02	HLA-A*02:02	424	433	KLPDDFTGCV	0.8161
A02	HLA-A*02:02	512	520	VLSFELLHA	0.5802
A02	HLA-A*02:02	515	524	FELLHAPATV	0.5863
A02	HLA-A*02:02	691	699	SIIAYTMSL	0.8402
A02	HLA-A*02:02	718	726	FTISVTTEI	0.6992
A02	HLA-A*02:02	821	829	LLFNKVTLA	0.8324
A02	HLA-A*02:02	857	865	GLTVLPPLL	0.7823
A02	HLA-A*02:02	869	877	MIAQYTSAL	0.7746
A02	HLA-A*02:02	915	923	VLYENQKLI	0.6825
A02	HLA-A*02:02	937	945	SLSSTASAL	0.6944
A02	HLA-A*02:02	947	956	KLQDVVNQNA	0.5801
A02	HLA-A*02:02	958	966	ALNTLVKQL	0.8332
A02	HLA-A*02:02	975	984	SVLNDILSRL	0.878
A02	HLA-A*02:02	976	984	VLNDILSRL	0.989
A02	HLA-A*02:02	983	991	RLDKVEAEV	0.8636
A02	HLA-A*02:02	1000	1008	RLQSLQTYV	0.8614
A02	HLA-A*02:02	1047	1056	YHLMSFPQSA	0.6493
A02	HLA-A*02:02	1048	1056	HLMSFPQSA	0.8395
A02	HLA-A*02:02	1060	1068	VVFLHVTYV	0.6976
A02	HLA-A*02:02	1095	1104	FVSNGTHWFV	0.621
A02	HLA-A*02:02	1185	1193	RLNEVAKNL	0.9052
A02	HLA-A*02:02	1192	1200	NLNESLIDL	0.8555
A02	HLA-A*02:02	1196	1203	SLIDLQEL	0.7114
A02	HLA-A*02:02	1220	1228	FIAGLIAIV	0.8951
A02	HLA-A*02:03	28	36	YTNSFTRGV	0.6379
A02	HLA-A*02:03	109	117	TLDSKTQSL	0.7157
A02	HLA-A*02:03	117	126	LLIVNNATNV	0.6411
A02	HLA-A*02:03	118	126	LIVNNATNV	0.6321
A02	HLA-A*02:03	119	127	IVNNATNVV	0.611
A02	HLA-A*02:03	202	210	KIYSKHTPI	0.6197
A02	HLA-A*02:03	241	249	LLALHRSYL	0.6696
A02	HLA-A*02:03	269	277	YLQPRTFLL	0.9474
A02	HLA-A*02:03	386	395	KLNDLCFTNV	0.8966
A02	HLA-A*02:03	417	425	KIADYNYKL	0.8266
A02	HLA-A*02:03	424	433	KLPDDFTGCV	0.8342
A02	HLA-A*02:03	512	520	VLSFELLHA	0.6927
A02	HLA-A*02:03	515	524	FELLHAPATV	0.75
A02	HLA-A*02:03	516	524	ELLHAPATV	0.6201
A02	HLA-A*02:03	610	620	VLYQDVNCTEV	0.6785
A02	HLA-A*02:03	634	642	RVYSTGSNV	0.7597
A02	HLA-A*02:03	691	699	SIIAYTMSL	0.8766
A02	HLA-A*02:03	718	726	FTISVTTEI	0.7603
A02	HLA-A*02:03	762	770	QLNRALTGI	0.7873
A02	HLA-A*02:03	821	829	LLFNKVTLA	0.9477

A02	HLA-A*02:03	869	877	MIAQYTSAL	0.7884
A02	HLA-A*02:03	915	923	VLYENQKLI	0.8141
A02	HLA-A*02:03	937	945	SLSSTASAL	0.7506
A02	HLA-A*02:03	947	956	KLQDVVNQNA	0.6818
A02	HLA-A*02:03	958	966	ALNTLVKQL	0.8303
A02	HLA-A*02:03	975	984	SVLNDILSRL	0.8775
A02	HLA-A*02:03	976	984	VLNDILSRL	0.986
A02	HLA-A*02:03	983	991	RLDKVEAEV	0.7407
A02	HLA-A*02:03	995	1004	RLITGRLQSL	0.6909
A02	HLA-A*02:03	1000	1008	RLQSLQTYV	0.9011
A02	HLA-A*02:03	1047	1056	YHLMSFPQSA	0.7925
A02	HLA-A*02:03	1048	1056	HLMSFPQSA	0.9264
A02	HLA-A*02:03	1060	1068	VVFLHVTYV	0.8543
A02	HLA-A*02:03	1171	1179	GINASVVNI	0.7487
A02	HLA-A*02:03	1185	1193	RLNEVAKNL	0.8898
A02	HLA-A*02:03	1192	1200	NLNESLIDL	0.7558
A02	HLA-A*02:03	1220	1228	FIAGLIAIV	0.9522
A02	HLA-A*02:04	269	277	YLQPRTFLL	0.8826
A02	HLA-A*02:04	417	425	KIADYNYKL	0.7529
A02	HLA-A*02:04	691	699	SIIAYTMSL	0.6521
A02	HLA-A*02:04	821	829	LLFNKVTLA	0.5441
A02	HLA-A*02:04	976	984	VLNDILSRL	0.7971
A02	HLA-A*02:04	1060	1068	VVFLHVTYV	0.5925
A02	HLA-A*02:05	28	36	YTNSFTRGV	0.5152
A02	HLA-A*02:05	133	141	FQFCNDPFL	0.6267
A02	HLA-A*02:05	269	277	YLQPRTFLL	0.8636
A02	HLA-A*02:05	417	425	KIADYNYKL	0.9165
A02	HLA-A*02:05	424	433	KLPDDFTGCV	0.6183
A02	HLA-A*02:05	691	699	SIIAYTMSL	0.7523
A02	HLA-A*02:05	718	726	FTISVTTEI	0.7976
A02	HLA-A*02:05	777	785	NTQEVFAQV	0.5189
A02	HLA-A*02:05	869	877	MIAQYTSAL	0.6311
A02	HLA-A*02:05	975	984	SVLNDILSRL	0.5129
A02	HLA-A*02:05	976	984	VLNDILSRL	0.9314
A02	HLA-A*02:05	1000	1008	RLQSLQTYV	0.5133
A02	HLA-A*02:05	1060	1068	VVFLHVTYV	0.6498
A02	HLA-A*02:05	1185	1193	RLNEVAKNL	0.5946
A02	HLA-A*02:05	1220	1228	FIAGLIAIV	0.7813
A02	HLA-A*02:06	133	141	FQFCNDPFL	0.7104
A02	HLA-A*02:06	133	143	FQFCNDPFLGV	0.5205
A02	HLA-A*02:06	269	277	YLQPRTFLL	0.8955
A02	HLA-A*02:06	417	425	KIADYNYKL	0.9064
A02	HLA-A*02:06	424	433	KLPDDFTGCV	0.5686
A02	HLA-A*02:06	612	620	YQDVNCTEV	0.5256
A02	HLA-A*02:06	691	699	SIIAYTMSL	0.847
A02	HLA-A*02:06	712	720	IAIPTNFTI	0.5224
A02	HLA-A*02:06	718	726	FTISVTTEI	0.8184
A02	HLA-A*02:06	721	729	SVTTEILPV	0.5642
A02	HLA-A*02:06	821	829	LLFNKVTLA	0.5419
A02	HLA-A*02:06	894	902	LQIPFAMQM	0.6147

A02	HLA-A*02:06	976	984	VLNDILSRL	0.8346
A02	HLA-A*02:06	983	991	RLDKVEAEV	0.6135
A02	HLA-A*02:06	1048	1056	HLMSFPQSA	0.5289
A02	HLA-A*02:06	1060	1068	VVFLHVTYV	0.7813
A02	HLA-A*02:06	1136	1145	TVYDPLQPEL	0.5968
A02	HLA-A*02:06	1220	1228	FIAGLIAIV	0.81
A02	HLA-A*02:07	269	277	YLQPRTFLL	0.8775
A02	HLA-A*02:09	109	117	TLDSKTQSL	0.6782
A02	HLA-A*02:09	133	141	FQFCNDPFL	0.518
A02	HLA-A*02:09	269	277	YLQPRTFLL	0.973
A02	HLA-A*02:09	386	395	KLNDLCFTNV	0.7108
A02	HLA-A*02:09	417	425	KIADYNYKL	0.909
A02	HLA-A*02:09	424	433	KLPDDFTGCV	0.5916
A02	HLA-A*02:09	515	524	FELLHAPATV	0.6033
A02	HLA-A*02:09	610	620	VLYQDVNCTEV	0.5092
A02	HLA-A*02:09	691	699	SIIAYTMSL	0.7995
A02	HLA-A*02:09	718	726	FTISVTTEI	0.5257
A02	HLA-A*02:09	821	829	LLFNKVTLA	0.7857
A02	HLA-A*02:09	857	865	GLTVLPPLL	0.6786
A02	HLA-A*02:09	915	923	VLYENQKLI	0.5378
A02	HLA-A*02:09	975	984	SVLNDILSRL	0.5124
A02	HLA-A*02:09	976	984	VLNDILSRL	0.9507
A02	HLA-A*02:09	983	991	RLDKVEAEV	0.8609
A02	HLA-A*02:09	1000	1008	RLQSLQTYV	0.7431
A02	HLA-A*02:09	1047	1056	YHLMSFPQSA	0.5452
A02	HLA-A*02:09	1048	1056	HLMSFPQSA	0.7385
A02	HLA-A*02:09	1060	1068	VVFLHVTYV	0.6948
A02	HLA-A*02:09	1185	1193	RLNEVAKNL	0.6189
A02	HLA-A*02:09	1192	1200	NLNESLIDL	0.6972
A02	HLA-A*02:09	1220	1228	FIAGLIAIV	0.8207
A02	HLA-A*02:11	109	117	TLDSKTQSL	0.8988
A02	HLA-A*02:11	269	277	YLQPRTFLL	0.9932
A02	HLA-A*02:11	386	395	KLNDLCFTNV	0.8871
A02	HLA-A*02:11	417	425	KIADYNYKL	0.97
A02	HLA-A*02:11	424	433	KLPDDFTGCV	0.7417
A02	HLA-A*02:11	515	524	FELLHAPATV	0.7889
A02	HLA-A*02:11	610	620	VLYQDVNCTEV	0.6974
A02	HLA-A*02:11	691	699	SIIAYTMSL	0.9432
A02	HLA-A*02:11	718	726	FTISVTTEI	0.8216
A02	HLA-A*02:11	821	829	LLFNKVTLA	0.9443
A02	HLA-A*02:11	857	865	GLTVLPPLL	0.7856
A02	HLA-A*02:11	915	923	VLYENQKLI	0.8632
A02	HLA-A*02:11	958	966	ALNTLVKQL	0.8141
A02	HLA-A*02:11	975	984	SVLNDILSRL	0.7945
A02	HLA-A*02:11	976	984	VLNDILSRL	0.9917
A02	HLA-A*02:11	983	991	RLDKVEAEV	0.9677
A02	HLA-A*02:11	1000	1008	RLQSLQTYV	0.9419
A02	HLA-A*02:11	1047	1056	YHLMSFPQSA	0.7518
A02	HLA-A*02:11	1048	1056	HLMSFPQSA	0.9136
A02	HLA-A*02:11	1060	1068	VVFLHVTYV	0.8865

A02	HLA-A*02:11	1171	1179	GINASVVNI	0.7552
A02	HLA-A*02:11	1185	1193	RLNEVAKNL	0.9099
A02	HLA-A*02:11	1192	1200	NLNESLIDL	0.8952
A02	HLA-A*02:11	1220	1228	FIAGLIAIV	0.9533
A02	HLA-A*02:12	109	117	TLDSKTQSL	0.8428
A02	HLA-A*02:12	269	277	YLQPRTFLL	0.9818
A02	HLA-A*02:12	386	395	KLNDLCFTNV	0.7871
A02	HLA-A*02:12	417	425	KIADYNYKL	0.9054
A02	HLA-A*02:12	424	433	KLPDDFTGCV	0.6822
A02	HLA-A*02:12	515	524	FELLHAPATV	0.681
A02	HLA-A*02:12	610	620	VLYQDVNCTEV	0.5467
A02	HLA-A*02:12	691	699	SIIAYTMSL	0.7857
A02	HLA-A*02:12	718	726	FTISVTTEI	0.5137
A02	HLA-A*02:12	821	829	LLFNKVTLA	0.8068
A02	HLA-A*02:12	857	865	GLTVLPPLL	0.6662
A02	HLA-A*02:12	915	923	VLYENQKLI	0.6143
A02	HLA-A*02:12	958	966	ALNTLVKQL	0.608
A02	HLA-A*02:12	975	984	SVLNDILSRL	0.6353
A02	HLA-A*02:12	976	984	VLNDILSRL	0.9646
A02	HLA-A*02:12	983	991	RLDKVEAEV	0.9238
A02	HLA-A*02:12	1000	1008	RLQSLQTYV	0.8752
A02	HLA-A*02:12	1047	1056	YHLMSFPQSA	0.6061
A02	HLA-A*02:12	1048	1056	HLMSFPQSA	0.7977
A02	HLA-A*02:12	1060	1068	VVFLHVTYV	0.6387
A02	HLA-A*02:12	1185	1193	RLNEVAKNL	0.7519
A02	HLA-A*02:12	1192	1200	NLNESLIDL	0.8032
A02	HLA-A*02:12	1196	1203	SLIDLQEL	0.5049
A02	HLA-A*02:12	1220	1228	FIAGLIAIV	0.8684
A02	HLA-A*02:13	109	117	TLDSKTQSL	0.6983
A02	HLA-A*02:13	269	277	YLQPRTFLL	0.941
A02	HLA-A*02:13	386	395	KLNDLCFTNV	0.8077
A02	HLA-A*02:13	417	425	KIADYNYKL	0.6473
A02	HLA-A*02:13	424	433	KLPDDFTGCV	0.6563
A02	HLA-A*02:13	515	524	FELLHAPATV	0.6753
A02	HLA-A*02:13	610	620	VLYQDVNCTEV	0.5488
A02	HLA-A*02:13	634	642	RVYSTGSNV	0.5202
A02	HLA-A*02:13	691	699	SIIAYTMSL	0.7124
A02	HLA-A*02:13	762	770	QLNRALTGI	0.5324
A02	HLA-A*02:13	821	829	LLFNKVTLA	0.8697
A02	HLA-A*02:13	915	923	VLYENQKLI	0.7047
A02	HLA-A*02:13	937	945	SLSSTASAL	0.5679
A02	HLA-A*02:13	958	966	ALNTLVKQL	0.6032
A02	HLA-A*02:13	975	984	SVLNDILSRL	0.6032
A02	HLA-A*02:13	976	984	VLNDILSRL	0.945
A02	HLA-A*02:13	983	991	RLDKVEAEV	0.7965
A02	HLA-A*02:13	1000	1008	RLQSLQTYV	0.8865
A02	HLA-A*02:13	1047	1056	YHLMSFPQSA	0.5921
A02	HLA-A*02:13	1048	1056	HLMSFPQSA	0.8168
A02	HLA-A*02:13	1060	1068	VVFLHVTYV	0.6866
A02	HLA-A*02:13	1171	1179	GINASVVNI	0.5495

A02	HLA-A*02:13	1185	1193	RLNEVAKNL	0.7327
A02	HLA-A*02:13	1192	1200	NLNESLIDL	0.6257
A02	HLA-A*02:13	1220	1228	FIAGLIAIV	0.8509
A02	HLA-A*02:14	133	141	FQFCNDPFL	0.6515
A02	HLA-A*02:14	269	277	YLQPRTFLL	0.8753
A02	HLA-A*02:14	417	425	KIADYNYKL	0.8777
A02	HLA-A*02:14	424	433	KLPDDFTGCV	0.5064
A02	HLA-A*02:14	691	699	SIIAYTMSL	0.7378
A02	HLA-A*02:14	718	726	FTISVTTEI	0.6932
A02	HLA-A*02:14	894	902	LQIPFAMQM	0.5806
A02	HLA-A*02:14	976	984	VLNDILSRL	0.8034
A02	HLA-A*02:14	983	991	RLDKVEAEV	0.5388
A02	HLA-A*02:14	1060	1068	VVFLHVTYV	0.6298
A02	HLA-A*02:14	1220	1228	FIAGLIAIV	0.6444
A02	HLA-A*02:16	109	117	TLDSKTQSL	0.7274
A02	HLA-A*02:16	133	141	FQFCNDPFL	0.6062
A02	HLA-A*02:16	222	233	ALEPLVDLPIGI	0.5004
A02	HLA-A*02:16	269	277	YLQPRTFLL	0.9796
A02	HLA-A*02:16	386	395	KLNDLCFTNV	0.7336
A02	HLA-A*02:16	417	425	KIADYNYKL	0.9083
A02	HLA-A*02:16	424	433	KLPDDFTGCV	0.6179
A02	HLA-A*02:16	515	524	FELLHAPATV	0.7196
A02	HLA-A*02:16	610	620	VLYQDVNCTEV	0.6397
A02	HLA-A*02:16	691	699	SIIAYTMSL	0.8424
A02	HLA-A*02:16	718	726	FTISVTTEI	0.6337
A02	HLA-A*02:16	821	829	LLFNKVTLA	0.8492
A02	HLA-A*02:16	857	865	GLTVLPPLL	0.7288
A02	HLA-A*02:16	915	923	VLYENQKLI	0.6401
A02	HLA-A*02:16	958	966	ALNTLVKQL	0.5125
A02	HLA-A*02:16	975	984	SVLNDILSRL	0.55
A02	HLA-A*02:16	976	984	VLNDILSRL	0.953
A02	HLA-A*02:16	983	991	RLDKVEAEV	0.9079
A02	HLA-A*02:16	1000	1008	RLQSLQTYV	0.8152
A02	HLA-A*02:16	1047	1056	YHLMSFPQSA	0.6323
A02	HLA-A*02:16	1048	1056	HLMSFPQSA	0.8072
A02	HLA-A*02:16	1060	1068	VVFLHVTYV	0.7386
A02	HLA-A*02:16	1185	1193	RLNEVAKNL	0.6572
A02	HLA-A*02:16	1192	1200	NLNESLIDL	0.7695
A02	HLA-A*02:16	1220	1228	FIAGLIAIV	0.8906
A02	HLA-A*02:17	269	277	YLQPRTFLL	0.8989
A02	HLA-A*02:17	417	425	KIADYNYKL	0.7084
A02	HLA-A*02:17	691	699	SIIAYTMSL	0.5715
A02	HLA-A*02:17	976	984	VLNDILSRL	0.7759
A02	HLA-A*02:18	269	277	YLQPRTFLL	0.8775
A02	HLA-A*02:19	109	117	TLDSKTQSL	0.8021
A02	HLA-A*02:19	269	277	YLQPRTFLL	0.9638
A02	HLA-A*02:19	386	395	KLNDLCFTNV	0.6484
A02	HLA-A*02:19	417	425	KIADYNYKL	0.7644
A02	HLA-A*02:19	424	433	KLPDDFTGCV	0.5568
A02	HLA-A*02:19	515	524	FELLHAPATV	0.5795

A02	HLA-A*02:19	516	524	ELLHAPATV	0.6189
A02	HLA-A*02:19	691	699	SIIAYTMSL	0.6332
A02	HLA-A*02:19	821	829	LLFNKVTLA	0.6129
A02	HLA-A*02:19	975	984	SVLNDILSRL	0.5189
A02	HLA-A*02:19	976	984	VLNDILSRL	0.9385
A02	HLA-A*02:19	983	991	RLDKVEAEV	0.8427
A02	HLA-A*02:19	1000	1008	RLQSLQTYV	0.7225
A02	HLA-A*02:19	1048	1056	HLMSFPQSA	0.6609
A02	HLA-A*02:19	1185	1193	RLNEVAKNL	0.5986
A02	HLA-A*02:19	1192	1200	NLNESLIDL	0.8007
A02	HLA-A*02:19	1220	1228	FIAGLIAIV	0.8159
A02	HLA-A*02:20	269	277	YLQPRTFLL	0.9372
A02	HLA-A*02:20	386	395	KLNDLCFTNV	0.609
A02	HLA-A*02:20	417	425	KIADYNYKL	0.8535
A02	HLA-A*02:20	691	699	SIIAYTMSL	0.7417
A02	HLA-A*02:20	718	726	FTISVTTEI	0.5048
A02	HLA-A*02:20	821	829	LLFNKVTLA	0.6533
A02	HLA-A*02:20	857	865	GLTVLPPLL	0.5648
A02	HLA-A*02:20	976	984	VLNDILSRL	0.883
A02	HLA-A*02:20	983	991	RLDKVEAEV	0.7865
A02	HLA-A*02:20	1000	1008	RLQSLQTYV	0.6742
A02	HLA-A*02:20	1048	1056	HLMSFPQSA	0.6259
A02	HLA-A*02:20	1060	1068	VVFLHVTYV	0.6374
A02	HLA-A*02:20	1192	1200	NLNESLIDL	0.5432
A02	HLA-A*02:20	1220	1228	FIAGLIAIV	0.6882
A02	HLA-A*02:21	133	141	FQFCNDPFL	0.7104
A02	HLA-A*02:21	133	143	FQFCNDPFLGV	0.5205
A02	HLA-A*02:21	269	277	YLQPRTFLL	0.8955
A02	HLA-A*02:21	417	425	KIADYNYKL	0.9064
A02	HLA-A*02:21	424	433	KLPDDFTGCV	0.5686
A02	HLA-A*02:21	612	620	YQDVNCTEV	0.5256
A02	HLA-A*02:21	691	699	SIIAYTMSL	0.847
A02	HLA-A*02:21	712	720	IAIPTNFTI	0.5224
A02	HLA-A*02:21	718	726	FTISVTTEI	0.8184
A02	HLA-A*02:21	721	729	SVTTEILPV	0.5642
A02	HLA-A*02:21	821	829	LLFNKVTLA	0.5419
A02	HLA-A*02:21	894	902	LQIPFAMQM	0.6147
A02	HLA-A*02:21	976	984	VLNDILSRL	0.8346
A02	HLA-A*02:21	983	991	RLDKVEAEV	0.6135
A02	HLA-A*02:21	1048	1056	HLMSFPQSA	0.5289
A02	HLA-A*02:21	1060	1068	VVFLHVTYV	0.7813
A02	HLA-A*02:21	1136	1145	TVYDPLQPEL	0.5968
A02	HLA-A*02:21	1220	1228	FIAGLIAIV	0.81
A02	HLA-A*02:22	109	117	TLDSKTQSL	0.8377
A02	HLA-A*02:22	133	141	FQFCNDPFL	0.6508
A02	HLA-A*02:22	269	277	YLQPRTFLL	0.9799
A02	HLA-A*02:22	386	395	KLNDLCFTNV	0.8656
A02	HLA-A*02:22	417	425	KIADYNYKL	0.9698
A02	HLA-A*02:22	424	433	KLPDDFTGCV	0.831
A02	HLA-A*02:22	512	520	VLSFELLHA	0.665

A02	HLA-A*02:22	515	524	FELLHAPATV	0.7224
A02	HLA-A*02:22	610	620	VLYQDVNCTEV	0.651
A02	HLA-A*02:22	691	699	SIIAYTMSL	0.882
A02	HLA-A*02:22	718	726	FTISVTTEI	0.8007
A02	HLA-A*02:22	762	770	QLNRALTGI	0.6498
A02	HLA-A*02:22	821	829	LLFNKVTLA	0.9056
A02	HLA-A*02:22	857	865	GLTVLPPLL	0.7806
A02	HLA-A*02:22	869	877	MIAQYTSAL	0.8034
A02	HLA-A*02:22	915	923	VLYENQKLI	0.746
A02	HLA-A*02:22	937	945	SLSSTASAL	0.7146
A02	HLA-A*02:22	947	956	KLQDVVNQNA	0.6761
A02	HLA-A*02:22	958	966	ALNTLVKQL	0.8347
A02	HLA-A*02:22	975	984	SVLNDILSRL	0.8914
A02	HLA-A*02:22	976	984	VLNDILSRL	0.9899
A02	HLA-A*02:22	983	991	RLDKVEAEV	0.8989
A02	HLA-A*02:22	1000	1008	RLQSLQTYV	0.9071
A02	HLA-A*02:22	1047	1056	YHLMSFPQSA	0.7592
A02	HLA-A*02:22	1048	1056	HLMSFPQSA	0.902
A02	HLA-A*02:22	1060	1068	VVFLHVTYV	0.7982
A02	HLA-A*02:22	1095	1104	FVSNGTHWFV	0.6858
A02	HLA-A*02:22	1171	1179	GINASVVNI	0.6497
A02	HLA-A*02:22	1185	1193	RLNEVAKNL	0.8958
A02	HLA-A*02:22	1192	1200	NLNESLIDL	0.8894
A02	HLA-A*02:22	1196	1203	SLIDLQEL	0.7342
A02	HLA-A*02:22	1220	1228	FIAGLIAIV	0.9461
A02	HLA-A*02:24	109	117	TLDSKTQSL	0.6782
A02	HLA-A*02:24	133	141	FQFCNDPFL	0.518
A02	HLA-A*02:24	269	277	YLQPRTFLL	0.973
A02	HLA-A*02:24	386	395	KLNDLCFTNV	0.7108
A02	HLA-A*02:24	417	425	KIADYNYKL	0.909
A02	HLA-A*02:24	424	433	KLPDDFTGCV	0.5916
A02	HLA-A*02:24	515	524	FELLHAPATV	0.6033
A02	HLA-A*02:24	610	620	VLYQDVNCTEV	0.5092
A02	HLA-A*02:24	691	699	SIIAYTMSL	0.7995
A02	HLA-A*02:24	718	726	FTISVTTEI	0.5257
A02	HLA-A*02:24	821	829	LLFNKVTLA	0.7857
A02	HLA-A*02:24	857	865	GLTVLPPLL	0.6786
A02	HLA-A*02:24	915	923	VLYENQKLI	0.5378
A02	HLA-A*02:24	975	984	SVLNDILSRL	0.5124
A02	HLA-A*02:24	976	984	VLNDILSRL	0.9507
A02	HLA-A*02:24	983	991	RLDKVEAEV	0.8609
A02	HLA-A*02:24	1000	1008	RLQSLQTYV	0.7431
A02	HLA-A*02:24	1047	1056	YHLMSFPQSA	0.5452
A02	HLA-A*02:24	1048	1056	HLMSFPQSA	0.7385
A02	HLA-A*02:24	1060	1068	VVFLHVTYV	0.6948
A02	HLA-A*02:24	1185	1193	RLNEVAKNL	0.6189
A02	HLA-A*02:24	1192	1200	NLNESLIDL	0.6972
A02	HLA-A*02:24	1220	1228	FIAGLIAIV	0.8207
A02	HLA-A*02:25	109	117	TLDSKTQSL	0.6782
A02	HLA-A*02:25	133	141	FQFCNDPFL	0.518

A02	HLA-A*02:25	269	277	YLQPRTFLL	0.973
A02	HLA-A*02:25	386	395	KLNDLCFTNV	0.7108
A02	HLA-A*02:25	417	425	KIADYNYKL	0.909
A02	HLA-A*02:25	424	433	KLPDDFTGCV	0.5916
A02	HLA-A*02:25	515	524	FELLHAPATV	0.6033
A02	HLA-A*02:25	610	620	VLYQDVNCTEV	0.5092
A02	HLA-A*02:25	691	699	SIIAYTMSL	0.7995
A02	HLA-A*02:25	718	726	FTISVTTEI	0.5257
A02	HLA-A*02:25	821	829	LLFNKVTLA	0.7857
A02	HLA-A*02:25	857	865	GLTVLPPLL	0.6786
A02	HLA-A*02:25	915	923	VLYENQKLI	0.5378
A02	HLA-A*02:25	975	984	SVLNDILSRL	0.5124
A02	HLA-A*02:25	976	984	VLNDILSRL	0.9507
A02	HLA-A*02:25	983	991	RLDKVEAEV	0.8609
A02	HLA-A*02:25	1000	1008	RLQSLQTYV	0.7431
A02	HLA-A*02:25	1047	1056	YHLMSFPQSA	0.5452
A02	HLA-A*02:25	1048	1056	HLMSFPQSA	0.7385
A02	HLA-A*02:25	1060	1068	VVFLHVTYV	0.6948
A02	HLA-A*02:25	1185	1193	RLNEVAKNL	0.6189
A02	HLA-A*02:25	1192	1200	NLNESLIDL	0.6972
A02	HLA-A*02:25	1220	1228	FIAGLIAIV	0.8207
A02	HLA-A*02:26	269	277	YLQPRTFLL	0.9227
A02	HLA-A*02:26	386	395	KLNDLCFTNV	0.779
A02	HLA-A*02:26	417	425	KIADYNYKL	0.6418
A02	HLA-A*02:26	424	433	KLPDDFTGCV	0.6197
A02	HLA-A*02:26	515	524	FELLHAPATV	0.6456
A02	HLA-A*02:26	610	620	VLYQDVNCTEV	0.564
A02	HLA-A*02:26	634	642	RVYSTGSNV	0.5791
A02	HLA-A*02:26	691	699	SIIAYTMSL	0.75
A02	HLA-A*02:26	718	726	FTISVTTEI	0.5223
A02	HLA-A*02:26	821	829	LLFNKVTLA	0.8849
A02	HLA-A*02:26	915	923	VLYENQKLI	0.6862
A02	HLA-A*02:26	975	984	SVLNDILSRL	0.5439
A02	HLA-A*02:26	976	984	VLNDILSRL	0.9394
A02	HLA-A*02:26	983	991	RLDKVEAEV	0.7333
A02	HLA-A*02:26	1000	1008	RLQSLQTYV	0.7922
A02	HLA-A*02:26	1047	1056	YHLMSFPQSA	0.5858
A02	HLA-A*02:26	1048	1056	HLMSFPQSA	0.8161
A02	HLA-A*02:26	1060	1068	VVFLHVTYV	0.7683
A02	HLA-A*02:26	1171	1179	GINASVVNI	0.5171
A02	HLA-A*02:26	1185	1193	RLNEVAKNL	0.664
A02	HLA-A*02:26	1192	1200	NLNESLIDL	0.524
A02	HLA-A*02:26	1220	1228	FIAGLIAIV	0.8354
A02	HLA-A*02:27	109	117	TLDSKTQSL	0.7308
A02	HLA-A*02:27	269	277	YLQPRTFLL	0.9647
A02	HLA-A*02:27	386	395	KLNDLCFTNV	0.7459
A02	HLA-A*02:27	417	425	KIADYNYKL	0.7613
A02	HLA-A*02:27	424	433	KLPDDFTGCV	0.5772
A02	HLA-A*02:27	515	524	FELLHAPATV	0.627
A02	HLA-A*02:27	610	620	VLYQDVNCTEV	0.514

A02	HLA-A*02:27	691	699	SIIAYTMSL	0.6826
A02	HLA-A*02:27	821	829	LLFNKVTLA	0.8062
A02	HLA-A*02:27	857	865	GLTVLPPLL	0.5289
A02	HLA-A*02:27	915	923	VLYENQKLI	0.6196
A02	HLA-A*02:27	958	966	ALNTLVKQL	0.5442
A02	HLA-A*02:27	975	984	SVLNDILSRL	0.5493
A02	HLA-A*02:27	976	984	VLNDILSRL	0.9341
A02	HLA-A*02:27	983	991	RLDKVEAEV	0.8729
A02	HLA-A*02:27	1000	1008	RLQSLQTYV	0.8703
A02	HLA-A*02:27	1047	1056	YHLMSFPQSA	0.5242
A02	HLA-A*02:27	1048	1056	HLMSFPQSA	0.7531
A02	HLA-A*02:27	1060	1068	VVFLHVTYV	0.5815
A02	HLA-A*02:27	1185	1193	RLNEVAKNL	0.685
A02	HLA-A*02:27	1192	1200	NLNESLIDL	0.6819
A02	HLA-A*02:27	1220	1228	FIAGLIAIV	0.798
A02	HLA-A*02:28	133	141	FQFCNDPFL	0.7104
A02	HLA-A*02:28	133	143	FQFCNDPFLGV	0.5205
A02	HLA-A*02:28	269	277	YLQPRTFLL	0.8955
A02	HLA-A*02:28	417	425	KIADYNYKL	0.9064
A02	HLA-A*02:28	424	433	KLPDDFTGCV	0.5686
A02	HLA-A*02:28	612	620	YQDVNCTEV	0.5256
A02	HLA-A*02:28	691	699	SIIAYTMSL	0.847
A02	HLA-A*02:28	712	720	IAIPTNFTI	0.5224
A02	HLA-A*02:28	718	726	FTISVTTEI	0.8184
A02	HLA-A*02:28	721	729	SVTTEILPV	0.5642
A02	HLA-A*02:28	821	829	LLFNKVTLA	0.5419
A02	HLA-A*02:28	894	902	LQIPFAMQM	0.6147
A02	HLA-A*02:28	976	984	VLNDILSRL	0.8346
A02	HLA-A*02:28	983	991	RLDKVEAEV	0.6135
A02	HLA-A*02:28	1048	1056	HLMSFPQSA	0.5289
A02	HLA-A*02:28	1060	1068	VVFLHVTYV	0.7813
A02	HLA-A*02:28	1136	1145	TVYDPLQPEL	0.5968
A02	HLA-A*02:28	1220	1228	FIAGLIAIV	0.81
A02	HLA-A*02:30	109	117	TLDSKTQSL	0.6782
A02	HLA-A*02:30	133	141	FQFCNDPFL	0.518
A02	HLA-A*02:30	269	277	YLQPRTFLL	0.973
A02	HLA-A*02:30	386	395	KLNDLCFTNV	0.7108
A02	HLA-A*02:30	417	425	KIADYNYKL	0.909
A02	HLA-A*02:30	424	433	KLPDDFTGCV	0.5916
A02	HLA-A*02:30	515	524	FELLHAPATV	0.6033
A02	HLA-A*02:30	610	620	VLYQDVNCTEV	0.5092
A02	HLA-A*02:30	691	699	SIIAYTMSL	0.7995
A02	HLA-A*02:30	718	726	FTISVTTEI	0.5257
A02	HLA-A*02:30	821	829	LLFNKVTLA	0.7857
A02	HLA-A*02:30	857	865	GLTVLPPLL	0.6786
A02	HLA-A*02:30	915	923	VLYENQKLI	0.5378
A02	HLA-A*02:30	975	984	SVLNDILSRL	0.5124
A02	HLA-A*02:30	976	984	VLNDILSRL	0.9507
A02	HLA-A*02:30	983	991	RLDKVEAEV	0.8609
A02	HLA-A*02:30	1000	1008	RLQSLQTYV	0.7431

A02	HLA-A*02:30	1047	1056	YHLMSFPQSA	0.5452
A02	HLA-A*02:30	1048	1056	HLMSFPQSA	0.7385
A02	HLA-A*02:30	1060	1068	VVFLHVTYV	0.6948
A02	HLA-A*02:30	1185	1193	RLNEVAKNL	0.6189
A02	HLA-A*02:30	1192	1200	NLNESLIDL	0.6972
A02	HLA-A*02:30	1220	1228	FIAGLIAIV	0.8207
A02	HLA-A*02:31	109	117	TLDSKTQSL	0.6782
A02	HLA-A*02:31	133	141	FQFCNDPFL	0.518
A02	HLA-A*02:31	269	277	YLQPRTFLL	0.973
A02	HLA-A*02:31	386	395	KLNDLCFTNV	0.7108
A02	HLA-A*02:31	417	425	KIADYNYKL	0.909
A02	HLA-A*02:31	424	433	KLPDDFTGCV	0.5916
A02	HLA-A*02:31	515	524	FELLHAPATV	0.6033
A02	HLA-A*02:31	610	620	VLYQDVNCTEV	0.5092
A02	HLA-A*02:31	691	699	SIIAYTMSL	0.7995
A02	HLA-A*02:31	718	726	FTISVTTEI	0.5257
A02	HLA-A*02:31	821	829	LLFNKVTLA	0.7857
A02	HLA-A*02:31	857	865	GLTVLPPLL	0.6786
A02	HLA-A*02:31	915	923	VLYENQKLI	0.5378
A02	HLA-A*02:31	975	984	SVLNDILSRL	0.5124
A02	HLA-A*02:31	976	984	VLNDILSRL	0.9507
A02	HLA-A*02:31	983	991	RLDKVEAEV	0.8609
A02	HLA-A*02:31	1000	1008	RLQSLQTYV	0.7431
A02	HLA-A*02:31	1047	1056	YHLMSFPQSA	0.5452
A02	HLA-A*02:31	1048	1056	HLMSFPQSA	0.7385
A02	HLA-A*02:31	1060	1068	VVFLHVTYV	0.6948
A02	HLA-A*02:31	1185	1193	RLNEVAKNL	0.6189
A02	HLA-A*02:31	1192	1200	NLNESLIDL	0.6972
A02	HLA-A*02:31	1220	1228	FIAGLIAIV	0.8207
A02	HLA-A*02:36	109	117	TLDSKTQSL	0.6446
A02	HLA-A*02:36	269	277	YLQPRTFLL	0.9384
A02	HLA-A*02:36	386	395	KLNDLCFTNV	0.5465
A02	HLA-A*02:36	417	425	KIADYNYKL	0.763
A02	HLA-A*02:36	515	524	FELLHAPATV	0.5014
A02	HLA-A*02:36	516	524	ELLHAPATV	0.5347
A02	HLA-A*02:36	691	699	SIIAYTMSL	0.6521
A02	HLA-A*02:36	821	829	LLFNKVTLA	0.595
A02	HLA-A*02:36	976	984	VLNDILSRL	0.9103
A02	HLA-A*02:36	983	991	RLDKVEAEV	0.7362
A02	HLA-A*02:36	1000	1008	RLQSLQTYV	0.5633
A02	HLA-A*02:36	1048	1056	HLMSFPQSA	0.5791
A02	HLA-A*02:36	1060	1068	VVFLHVTYV	0.5499
A02	HLA-A*02:36	1192	1200	NLNESLIDL	0.6714
A02	HLA-A*02:36	1220	1228	FIAGLIAIV	0.7437
A02	HLA-A*02:38	109	117	TLDSKTQSL	0.6763
A02	HLA-A*02:38	269	277	YLQPRTFLL	0.8978
A02	HLA-A*02:38	386	395	KLNDLCFTNV	0.6697
A02	HLA-A*02:38	417	425	KIADYNYKL	0.6147
A02	HLA-A*02:38	424	433	KLPDDFTGCV	0.5937
A02	HLA-A*02:38	515	524	FELLHAPATV	0.6135

A02	HLA-A*02:38	610	620	VLYQDVNCTEV	0.5165
A02	HLA-A*02:38	691	699	SIIAYTMSL	0.7292
A02	HLA-A*02:38	718	726	FTISVTTEI	0.5114
A02	HLA-A*02:38	821	829	LLFNKVTLA	0.7991
A02	HLA-A*02:38	915	923	VLYENQKLI	0.6879
A02	HLA-A*02:38	937	945	SLSSTASAL	0.5212
A02	HLA-A*02:38	958	966	ALNTLVKQL	0.5209
A02	HLA-A*02:38	975	984	SVLNDILSRL	0.5586
A02	HLA-A*02:38	976	984	VLNDILSRL	0.9012
A02	HLA-A*02:38	983	991	RLDKVEAEV	0.7049
A02	HLA-A*02:38	1000	1008	RLQSLQTYV	0.7606
A02	HLA-A*02:38	1047	1056	YHLMSFPQSA	0.5778
A02	HLA-A*02:38	1048	1056	HLMSFPQSA	0.7518
A02	HLA-A*02:38	1060	1068	VVFLHVTYV	0.6814
A02	HLA-A*02:38	1171	1179	GINASVVNI	0.5375
A02	HLA-A*02:38	1185	1193	RLNEVAKNL	0.6207
A02	HLA-A*02:38	1192	1200	NLNESLIDL	0.6694
A02	HLA-A*02:38	1220	1228	FIAGLIAIV	0.8041
A02	HLA-A*02:39	269	277	YLQPRTFLL	0.9532
A02	HLA-A*02:39	417	425	KIADYNYKL	0.7841
A02	HLA-A*02:39	691	699	SIIAYTMSL	0.5907
A02	HLA-A*02:39	821	829	LLFNKVTLA	0.5231
A02	HLA-A*02:39	857	865	GLTVLPPLL	0.5068
A02	HLA-A*02:39	976	984	VLNDILSRL	0.8817
A02	HLA-A*02:39	983	991	RLDKVEAEV	0.6488
A02	HLA-A*02:39	1000	1008	RLQSLQTYV	0.5067
A02	HLA-A*02:39	1192	1200	NLNESLIDL	0.5275
A02	HLA-A*02:39	1220	1228	FIAGLIAIV	0.6464
A02	HLA-A*02:40	109	117	TLDSKTQSL	0.6782
A02	HLA-A*02:40	133	141	FQFCNDPFL	0.518
A02	HLA-A*02:40	269	277	YLQPRTFLL	0.973
A02	HLA-A*02:40	386	395	KLNDLCFTNV	0.7108
A02	HLA-A*02:40	417	425	KIADYNYKL	0.909
A02	HLA-A*02:40	424	433	KLPDDFTGCV	0.5916
A02	HLA-A*02:40	515	524	FELLHAPATV	0.6033
A02	HLA-A*02:40	610	620	VLYQDVNCTEV	0.5092
A02	HLA-A*02:40	691	699	SIIAYTMSL	0.7995
A02	HLA-A*02:40	718	726	FTISVTTEI	0.5257
A02	HLA-A*02:40	821	829	LLFNKVTLA	0.7857
A02	HLA-A*02:40	857	865	GLTVLPPLL	0.6786
A02	HLA-A*02:40	915	923	VLYENQKLI	0.5378
A02	HLA-A*02:40	975	984	SVLNDILSRL	0.5124
A02	HLA-A*02:40	976	984	VLNDILSRL	0.9507
A02	HLA-A*02:40	983	991	RLDKVEAEV	0.8609
A02	HLA-A*02:40	1000	1008	RLQSLQTYV	0.7431
A02	HLA-A*02:40	1047	1056	YHLMSFPQSA	0.5452
A02	HLA-A*02:40	1048	1056	HLMSFPQSA	0.7385
A02	HLA-A*02:40	1060	1068	VVFLHVTYV	0.6948
A02	HLA-A*02:40	1185	1193	RLNEVAKNL	0.6189
A02	HLA-A*02:40	1192	1200	NLNESLIDL	0.6972

A02	HLA-A*02:40	1220	1228	FIAGLIAIV	0.8207
A02	HLA-A*02:44	109	117	TLDSKTQSL	0.722
A02	HLA-A*02:44	133	141	FQFCNDPFL	0.6901
A02	HLA-A*02:44	269	277	YLQPRTFLL	0.9483
A02	HLA-A*02:44	386	395	KLNDLCFTNV	0.6273
A02	HLA-A*02:44	417	425	KIADYNYKL	0.9374
A02	HLA-A*02:44	424	433	KLPDDFTGCV	0.713
A02	HLA-A*02:44	612	620	YQDVNCTEV	0.6481
A02	HLA-A*02:44	691	699	SIIAYTMSL	0.873
A02	HLA-A*02:44	718	726	FTISVTTEI	0.8345
A02	HLA-A*02:44	721	729	SVTTEILPV	0.6283
A02	HLA-A*02:44	777	785	NTQEVFAQV	0.5675
A02	HLA-A*02:44	786	794	KQIYKTPPI	0.5169
A02	HLA-A*02:44	821	829	LLFNKVTLA	0.6213
A02	HLA-A*02:44	869	877	MIAQYTSAL	0.5812
A02	HLA-A*02:44	976	984	VLNDILSRL	0.9188
A02	HLA-A*02:44	983	991	RLDKVEAEV	0.7861
A02	HLA-A*02:44	1000	1008	RLQSLQTYV	0.7229
A02	HLA-A*02:44	1048	1056	HLMSFPQSA	0.6725
A02	HLA-A*02:44	1060	1068	VVFLHVTYV	0.7638
A02	HLA-A*02:44	1095	1104	FVSNGTHWFV	0.5522
A02	HLA-A*02:44	1136	1145	TVYDPLQPEL	0.5349
A02	HLA-A*02:44	1171	1179	GINASVVNI	0.5357
A02	HLA-A*02:44	1185	1193	RLNEVAKNL	0.5876
A02	HLA-A*02:44	1192	1200	NLNESLIDL	0.6815
A02	HLA-A*02:44	1196	1203	SLIDLQEL	0.5121
A02	HLA-A*02:44	1220	1228	FIAGLIAIV	0.868
A02	HLA-A*02:45	269	277	YLQPRTFLL	0.87
A02	HLA-A*02:45	417	425	KIADYNYKL	0.7495
A02	HLA-A*02:45	691	699	SIIAYTMSL	0.5724
A02	HLA-A*02:45	976	984	VLNDILSRL	0.7909
A02	HLA-A*02:45	983	991	RLDKVEAEV	0.6527
A02	HLA-A*02:46	269	277	YLQPRTFLL	0.897
A02	HLA-A*02:46	417	425	KIADYNYKL	0.7857
A02	HLA-A*02:46	691	699	SIIAYTMSL	0.5832
A02	HLA-A*02:46	976	984	VLNDILSRL	0.8016
A02	HLA-A*02:46	983	991	RLDKVEAEV	0.6558
A02	HLA-A*02:47	109	117	TLDSKTQSL	0.8176
A02	HLA-A*02:47	133	141	FQFCNDPFL	0.5963
A02	HLA-A*02:47	225	233	PLVDLPIGI	0.5831
A02	HLA-A*02:47	269	277	YLQPRTFLL	0.9772
A02	HLA-A*02:47	386	395	KLNDLCFTNV	0.8117
A02	HLA-A*02:47	416	425	GKIADYNYKL	0.6744
A02	HLA-A*02:47	417	425	KIADYNYKL	0.9749
A02	HLA-A*02:47	424	433	KLPDDFTGCV	0.8227
A02	HLA-A*02:47	691	699	SIIAYTMSL	0.8546
A02	HLA-A*02:47	718	726	FTISVTTEI	0.7036
A02	HLA-A*02:47	821	829	LLFNKVTLA	0.8262
A02	HLA-A*02:47	857	865	GLTVLPPLL	0.7895
A02	HLA-A*02:47	869	877	MIAQYTSAL	0.7897

A02	HLA-A*02:47	915	923	VLYENQKLI	0.6812
A02	HLA-A*02:47	937	945	SLSSTASAL	0.6691
A02	HLA-A*02:47	958	966	ALNTLVKQL	0.8279
A02	HLA-A*02:47	975	984	SVLNDILSRL	0.8782
A02	HLA-A*02:47	976	984	VLNDILSRL	0.9886
A02	HLA-A*02:47	983	991	RLDKVEAEV	0.849
A02	HLA-A*02:47	1000	1008	RLQSLQTYV	0.8402
A02	HLA-A*02:47	1047	1056	YHLMSFPQSA	0.6304
A02	HLA-A*02:47	1048	1056	HLMSFPQSA	0.8224
A02	HLA-A*02:47	1060	1068	VVFLHVTYV	0.7118
A02	HLA-A*02:47	1095	1104	FVSNGTHWFV	0.6164
A02	HLA-A*02:47	1185	1193	RLNEVAKNL	0.9004
A02	HLA-A*02:47	1192	1200	NLNESLIDL	0.8471
A02	HLA-A*02:47	1196	1203	SLIDLQEL	0.7353
A02	HLA-A*02:47	1220	1228	FIAGLIAIV	0.8847
A02	HLA-A*02:48	109	117	TLDSKTQSL	0.52
A02	HLA-A*02:48	269	277	YLQPRTFLL	0.9298
A02	HLA-A*02:48	417	425	KIADYNYKL	0.8626
A02	HLA-A*02:48	691	699	SIIAYTMSL	0.69
A02	HLA-A*02:48	976	984	VLNDILSRL	0.8779
A02	HLA-A*02:48	983	991	RLDKVEAEV	0.7153
A02	HLA-A*02:48	1000	1008	RLQSLQTYV	0.6002
A02	HLA-A*02:48	1060	1068	VVFLHVTYV	0.5094
A02	HLA-A*02:48	1185	1193	RLNEVAKNL	0.5841
A02	HLA-A*02:49	109	117	TLDSKTQSL	0.8693
A02	HLA-A*02:49	269	277	YLQPRTFLL	0.9729
A02	HLA-A*02:49	386	395	KLNDLCFTNV	0.6741
A02	HLA-A*02:49	417	425	KIADYNYKL	0.8873
A02	HLA-A*02:49	424	433	KLPDDFTGCV	0.6103
A02	HLA-A*02:49	515	524	FELLHAPATV	0.5508
A02	HLA-A*02:49	612	620	YQDVNCTEV	0.5076
A02	HLA-A*02:49	691	699	SIIAYTMSL	0.6942
A02	HLA-A*02:49	821	829	LLFNKVTLA	0.6712
A02	HLA-A*02:49	857	865	GLTVLPPLL	0.5991
A02	HLA-A*02:49	975	984	SVLNDILSRL	0.5907
A02	HLA-A*02:49	976	984	VLNDILSRL	0.9452
A02	HLA-A*02:49	982	991	SRLDKVEAEV	0.5478
A02	HLA-A*02:49	983	991	RLDKVEAEV	0.9308
A02	HLA-A*02:49	1000	1008	RLQSLQTYV	0.8043
A02	HLA-A*02:49	1048	1056	HLMSFPQSA	0.6591
A02	HLA-A*02:49	1060	1068	VVFLHVTYV	0.571
A02	HLA-A*02:49	1185	1193	RLNEVAKNL	0.6724
A02	HLA-A*02:49	1192	1200	NLNESLIDL	0.7823
A02	HLA-A*02:49	1220	1228	FIAGLIAIV	0.7966
A02	HLA-A*02:51	133	141	FQFCNDPFL	0.7104
A02	HLA-A*02:51	133	143	FQFCNDPFLGV	0.5205
A02	HLA-A*02:51	269	277	YLQPRTFLL	0.8955
A02	HLA-A*02:51	417	425	KIADYNYKL	0.9064
A02	HLA-A*02:51	424	433	KLPDDFTGCV	0.5686
A02	HLA-A*02:51	612	620	YQDVNCTEV	0.5256

A02	HLA-A*02:51	691	699	SIIAYTMSL	0.847
A02	HLA-A*02:51	712	720	IAIPTNFTI	0.5224
A02	HLA-A*02:51	718	726	FTISVTTEI	0.8184
A02	HLA-A*02:51	721	729	SVTTEILPV	0.5642
A02	HLA-A*02:51	821	829	LLFNKVTLA	0.5419
A02	HLA-A*02:51	894	902	LQIPFAMQM	0.6147
A02	HLA-A*02:51	976	984	VLNDILSRL	0.8346
A02	HLA-A*02:51	983	991	RLDKVEAEV	0.6135
A02	HLA-A*02:51	1048	1056	HLMSFPQSA	0.5289
A02	HLA-A*02:51	1060	1068	VVFLHVTYV	0.7813
A02	HLA-A*02:51	1136	1145	TVYDPLQPEL	0.5968
A02	HLA-A*02:51	1220	1228	FIAGLIAIV	0.81
A02	HLA-A*02:54	109	117	TLDSKTQSL	0.6942
A02	HLA-A*02:54	269	277	YLQPRTFLL	0.9082
A02	HLA-A*02:54	417	425	KIADYNYKL	0.8163
A02	HLA-A*02:54	424	433	KLPDDFTGCV	0.5607
A02	HLA-A*02:54	516	524	ELLHAPATV	0.5493
A02	HLA-A*02:54	612	620	YQDVNCTEV	0.5542
A02	HLA-A*02:54	691	699	SIIAYTMSL	0.7472
A02	HLA-A*02:54	718	726	FTISVTTEI	0.731
A02	HLA-A*02:54	721	729	SVTTEILPV	0.5157
A02	HLA-A*02:54	777	785	NTQEVFAQV	0.6031
A02	HLA-A*02:54	976	984	VLNDILSRL	0.8799
A02	HLA-A*02:54	983	991	RLDKVEAEV	0.6743
A02	HLA-A*02:54	1000	1008	RLQSLQTYV	0.5446
A02	HLA-A*02:54	1048	1056	HLMSFPQSA	0.532
A02	HLA-A*02:54	1060	1068	VVFLHVTYV	0.6044
A02	HLA-A*02:54	1192	1200	NLNESLIDL	0.6437
A02	HLA-A*02:54	1220	1228	FIAGLIAIV	0.7718
A02	HLA-A*02:56	269	277	YLQPRTFLL	0.6126
A02	HLA-A*02:56	417	425	KIADYNYKL	0.6147
A02	HLA-A*02:56	691	699	SIIAYTMSL	0.5351
A02	HLA-A*02:56	1060	1068	VVFLHVTYV	0.5227
A02	HLA-A*02:57	269	277	YLQPRTFLL	0.7289
A02	HLA-A*02:57	417	425	KIADYNYKL	0.7431
A02	HLA-A*02:57	691	699	SIIAYTMSL	0.6331
A02	HLA-A*02:57	976	984	VLNDILSRL	0.5643
A02	HLA-A*02:57	1060	1068	VVFLHVTYV	0.5368
A02	HLA-A*02:58	109	117	TLDSKTQSL	0.6907
A02	HLA-A*02:58	269	277	YLQPRTFLL	0.9723
A02	HLA-A*02:58	386	395	KLNDLCFTNV	0.6236
A02	HLA-A*02:58	417	425	KIADYNYKL	0.9119
A02	HLA-A*02:58	424	433	KLPDDFTGCV	0.5701
A02	HLA-A*02:58	691	699	SIIAYTMSL	0.7337
A02	HLA-A*02:58	821	829	LLFNKVTLA	0.6551
A02	HLA-A*02:58	857	865	GLTVLPPLL	0.6884
A02	HLA-A*02:58	975	984	SVLNDILSRL	0.5159
A02	HLA-A*02:58	976	984	VLNDILSRL	0.95
A02	HLA-A*02:58	983	991	RLDKVEAEV	0.8408
A02	HLA-A*02:58	1000	1008	RLQSLQTYV	0.656

A02	HLA-A*02:58	1048	1056	HLMSFPQSA	0.623
A02	HLA-A*02:58	1060	1068	VVFLHVTYV	0.5564
A02	HLA-A*02:58	1185	1193	RLNEVAKNL	0.6577
A02	HLA-A*02:58	1192	1200	NLNESLIDL	0.6601
A02	HLA-A*02:58	1220	1228	FIAGLIAIV	0.707
A02	HLA-A*02:59	109	117	TLDSKTQSL	0.6782
A02	HLA-A*02:59	133	141	FQFCNDPFL	0.518
A02	HLA-A*02:59	269	277	YLQPRTFLL	0.973
A02	HLA-A*02:59	386	395	KLNDLCFTNV	0.7108
A02	HLA-A*02:59	417	425	KIADYNYKL	0.909
A02	HLA-A*02:59	424	433	KLPDDFTGCV	0.5916
A02	HLA-A*02:59	515	524	FELLHAPATV	0.6033
A02	HLA-A*02:59	610	620	VLYQDVNCTEV	0.5092
A02	HLA-A*02:59	691	699	SIIAYTMSL	0.7995
A02	HLA-A*02:59	718	726	FTISVTTEI	0.5257
A02	HLA-A*02:59	821	829	LLFNKVTLA	0.7857
A02	HLA-A*02:59	857	865	GLTVLPPLL	0.6786
A02	HLA-A*02:59	915	923	VLYENQKLI	0.5378
A02	HLA-A*02:59	975	984	SVLNDILSRL	0.5124
A02	HLA-A*02:59	976	984	VLNDILSRL	0.9507
A02	HLA-A*02:59	983	991	RLDKVEAEV	0.8609
A02	HLA-A*02:59	1000	1008	RLQSLQTYV	0.7431
A02	HLA-A*02:59	1047	1056	YHLMSFPQSA	0.5452
A02	HLA-A*02:59	1048	1056	HLMSFPQSA	0.7385
A02	HLA-A*02:59	1060	1068	VVFLHVTYV	0.6948
A02	HLA-A*02:59	1185	1193	RLNEVAKNL	0.6189
A02	HLA-A*02:59	1192	1200	NLNESLIDL	0.6972
A02	HLA-A*02:59	1220	1228	FIAGLIAIV	0.8207
A02	HLA-A*02:61	133	141	FQFCNDPFL	0.7104
A02	HLA-A*02:61	133	143	FQFCNDPFLGV	0.5205
A02	HLA-A*02:61	269	277	YLQPRTFLL	0.8955
A02	HLA-A*02:61	417	425	KIADYNYKL	0.9064
A02	HLA-A*02:61	424	433	KLPDDFTGCV	0.5686
A02	HLA-A*02:61	612	620	YQDVNCTEV	0.5256
A02	HLA-A*02:61	691	699	SIIAYTMSL	0.847
A02	HLA-A*02:61	712	720	IAIPTNFTI	0.5224
A02	HLA-A*02:61	718	726	FTISVTTEI	0.8184
A02	HLA-A*02:61	721	729	SVTTEILPV	0.5642
A02	HLA-A*02:61	821	829	LLFNKVTLA	0.5419
A02	HLA-A*02:61	894	902	LQIPFAMQM	0.6147
A02	HLA-A*02:61	976	984	VLNDILSRL	0.8346
A02	HLA-A*02:61	983	991	RLDKVEAEV	0.6135
A02	HLA-A*02:61	1048	1056	HLMSFPQSA	0.5289
A02	HLA-A*02:61	1060	1068	VVFLHVTYV	0.7813
A02	HLA-A*02:61	1136	1145	TVYDPLQPEL	0.5968
A02	HLA-A*02:61	1220	1228	FIAGLIAIV	0.81
A02	HLA-A*02:62	269	277	YLQPRTFLL	0.778
A02	HLA-A*02:62	417	425	KIADYNYKL	0.6875
A02	HLA-A*02:62	691	699	SIIAYTMSL	0.6033
A02	HLA-A*02:62	821	829	LLFNKVTLA	0.5193

A02	HLA-A*02:62	976	984	VLNDILSRL	0.681
A02	HLA-A*02:62	983	991	RLDKVEAEV	0.636
A02	HLA-A*02:62	1000	1008	RLQSLQTYV	0.5378
A02	HLA-A*02:62	1060	1068	VVFLHVTYV	0.6207
A02	HLA-A*02:62	1220	1228	FIAGLIAIV	0.5269
A02	HLA-A*02:63	109	117	TLDSKTQSL	0.825
A02	HLA-A*02:63	133	141	FQFCNDPFL	0.5968
A02	HLA-A*02:63	269	277	YLQPRTFLL	0.9773
A02	HLA-A*02:63	386	395	KLNDLCFTNV	0.8261
A02	HLA-A*02:63	416	425	GKIADYNYKL	0.6474
A02	HLA-A*02:63	417	425	KIADYNYKL	0.9705
A02	HLA-A*02:63	424	433	KLPDDFTGCV	0.8161
A02	HLA-A*02:63	512	520	VLSFELLHA	0.5802
A02	HLA-A*02:63	515	524	FELLHAPATV	0.5863
A02	HLA-A*02:63	691	699	SIIAYTMSL	0.8402
A02	HLA-A*02:63	718	726	FTISVTTEI	0.6992
A02	HLA-A*02:63	821	829	LLFNKVTLA	0.8324
A02	HLA-A*02:63	857	865	GLTVLPPLL	0.7823
A02	HLA-A*02:63	869	877	MIAQYTSAL	0.7746
A02	HLA-A*02:63	915	923	VLYENQKLI	0.6825
A02	HLA-A*02:63	937	945	SLSSTASAL	0.6944
A02	HLA-A*02:63	947	956	KLQDVVNQNA	0.5801
A02	HLA-A*02:63	958	966	ALNTLVKQL	0.8332
A02	HLA-A*02:63	975	984	SVLNDILSRL	0.878
A02	HLA-A*02:63	976	984	VLNDILSRL	0.989
A02	HLA-A*02:63	983	991	RLDKVEAEV	0.8636
A02	HLA-A*02:63	1000	1008	RLQSLQTYV	0.8614
A02	HLA-A*02:63	1047	1056	YHLMSFPQSA	0.6493
A02	HLA-A*02:63	1048	1056	HLMSFPQSA	0.8395
A02	HLA-A*02:63	1060	1068	VVFLHVTYV	0.6976
A02	HLA-A*02:63	1095	1104	FVSNGTHWFV	0.621
A02	HLA-A*02:63	1185	1193	RLNEVAKNL	0.9052
A02	HLA-A*02:63	1192	1200	NLNESLIDL	0.8555
A02	HLA-A*02:63	1196	1203	SLIDLQEL	0.7114
A02	HLA-A*02:63	1220	1228	FIAGLIAIV	0.8951
A02	HLA-A*02:66	109	117	TLDSKTQSL	0.6782
A02	HLA-A*02:66	133	141	FQFCNDPFL	0.518
A02	HLA-A*02:66	269	277	YLQPRTFLL	0.973
A02	HLA-A*02:66	386	395	KLNDLCFTNV	0.7108
A02	HLA-A*02:66	417	425	KIADYNYKL	0.909
A02	HLA-A*02:66	424	433	KLPDDFTGCV	0.5916
A02	HLA-A*02:66	515	524	FELLHAPATV	0.6033
A02	HLA-A*02:66	610	620	VLYQDVNCTEV	0.5092
A02	HLA-A*02:66	691	699	SIIAYTMSL	0.7995
A02	HLA-A*02:66	718	726	FTISVTTEI	0.5257
A02	HLA-A*02:66	821	829	LLFNKVTLA	0.7857
A02	HLA-A*02:66	857	865	GLTVLPPLL	0.6786
A02	HLA-A*02:66	915	923	VLYENQKLI	0.5378
A02	HLA-A*02:66	975	984	SVLNDILSRL	0.5124
A02	HLA-A*02:66	976	984	VLNDILSRL	0.9507

A02	HLA-A*02:66	983	991	RLDKVEAEV	0.8609
A02	HLA-A*02:66	1000	1008	RLQSLQTYV	0.7431
A02	HLA-A*02:66	1047	1056	YHLMSFPQSA	0.5452
A02	HLA-A*02:66	1048	1056	HLMSFPQSA	0.7385
A02	HLA-A*02:66	1060	1068	VVFLHVTYV	0.6948
A02	HLA-A*02:66	1185	1193	RLNEVAKNL	0.6189
A02	HLA-A*02:66	1192	1200	NLNESLIDL	0.6972
A02	HLA-A*02:66	1220	1228	FIAGLIAIV	0.8207
A02	HLA-A*02:67	109	117	TLDSKTQSL	0.6782
A02	HLA-A*02:67	133	141	FQFCNDPFL	0.518
A02	HLA-A*02:67	269	277	YLQPRTFLL	0.973
A02	HLA-A*02:67	386	395	KLNDLCFTNV	0.7108
A02	HLA-A*02:67	417	425	KIADYNYKL	0.909
A02	HLA-A*02:67	424	433	KLPDDFTGCV	0.5916
A02	HLA-A*02:67	515	524	FELLHAPATV	0.6033
A02	HLA-A*02:67	610	620	VLYQDVNCTEV	0.5092
A02	HLA-A*02:67	691	699	SIIAYTMSL	0.7995
A02	HLA-A*02:67	718	726	FTISVTTEI	0.5257
A02	HLA-A*02:67	821	829	LLFNKVTLA	0.7857
A02	HLA-A*02:67	857	865	GLTVLPPLL	0.6786
A02	HLA-A*02:67	915	923	VLYENQKLI	0.5378
A02	HLA-A*02:67	975	984	SVLNDILSRL	0.5124
A02	HLA-A*02:67	976	984	VLNDILSRL	0.9507
A02	HLA-A*02:67	983	991	RLDKVEAEV	0.8609
A02	HLA-A*02:67	1000	1008	RLQSLQTYV	0.7431
A02	HLA-A*02:67	1047	1056	YHLMSFPQSA	0.5452
A02	HLA-A*02:67	1048	1056	HLMSFPQSA	0.7385
A02	HLA-A*02:67	1060	1068	VVFLHVTYV	0.6948
A02	HLA-A*02:67	1185	1193	RLNEVAKNL	0.6189
A02	HLA-A*02:67	1192	1200	NLNESLIDL	0.6972
A02	HLA-A*02:67	1220	1228	FIAGLIAIV	0.8207
A02	HLA-A*02:68	109	117	TLDSKTQSL	0.6782
A02	HLA-A*02:68	133	141	FQFCNDPFL	0.518
A02	HLA-A*02:68	269	277	YLQPRTFLL	0.973
A02	HLA-A*02:68	386	395	KLNDLCFTNV	0.7108
A02	HLA-A*02:68	417	425	KIADYNYKL	0.909
A02	HLA-A*02:68	424	433	KLPDDFTGCV	0.5916
A02	HLA-A*02:68	515	524	FELLHAPATV	0.6033
A02	HLA-A*02:68	610	620	VLYQDVNCTEV	0.5092
A02	HLA-A*02:68	691	699	SIIAYTMSL	0.7995
A02	HLA-A*02:68	718	726	FTISVTTEI	0.5257
A02	HLA-A*02:68	821	829	LLFNKVTLA	0.7857
A02	HLA-A*02:68	857	865	GLTVLPPLL	0.6786
A02	HLA-A*02:68	915	923	VLYENQKLI	0.5378
A02	HLA-A*02:68	975	984	SVLNDILSRL	0.5124
A02	HLA-A*02:68	976	984	VLNDILSRL	0.9507
A02	HLA-A*02:68	983	991	RLDKVEAEV	0.8609
A02	HLA-A*02:68	1000	1008	RLQSLQTYV	0.7431
A02	HLA-A*02:68	1047	1056	YHLMSFPQSA	0.5452
A02	HLA-A*02:68	1048	1056	HLMSFPQSA	0.7385

A02	HLA-A*02:68	1060	1068	VVFLHVTYV	0.6948
A02	HLA-A*02:68	1185	1193	RLNEVAKNL	0.6189
A02	HLA-A*02:68	1192	1200	NLNESLIDL	0.6972
A02	HLA-A*02:68	1220	1228	FIAGLIAIV	0.8207
A02	HLA-A*02:69	109	117	TLDSKTQSL	0.8988
A02	HLA-A*02:69	269	277	YLQPRTFLL	0.9932
A02	HLA-A*02:69	386	395	KLNDLCFTNV	0.8871
A02	HLA-A*02:69	417	425	KIADYNYKL	0.97
A02	HLA-A*02:69	424	433	KLPDDFTGCV	0.7417
A02	HLA-A*02:69	515	524	FELLHAPATV	0.7889
A02	HLA-A*02:69	610	620	VLYQDVNCTEV	0.6974
A02	HLA-A*02:69	691	699	SIIAYTMSL	0.9432
A02	HLA-A*02:69	718	726	FTISVTTEI	0.8216
A02	HLA-A*02:69	821	829	LLFNKVTLA	0.9443
A02	HLA-A*02:69	857	865	GLTVLPPLL	0.7856
A02	HLA-A*02:69	915	923	VLYENQKLI	0.8632
A02	HLA-A*02:69	958	966	ALNTLVKQL	0.8141
A02	HLA-A*02:69	975	984	SVLNDILSRL	0.7945
A02	HLA-A*02:69	976	984	VLNDILSRL	0.9917
A02	HLA-A*02:69	983	991	RLDKVEAEV	0.9677
A02	HLA-A*02:69	1000	1008	RLQSLQTYV	0.9419
A02	HLA-A*02:69	1047	1056	YHLMSFPQSA	0.7518
A02	HLA-A*02:69	1048	1056	HLMSFPQSA	0.9136
A02	HLA-A*02:69	1060	1068	VVFLHVTYV	0.8865
A02	HLA-A*02:69	1171	1179	GINASVVNI	0.7552
A02	HLA-A*02:69	1185	1193	RLNEVAKNL	0.9099
A02	HLA-A*02:69	1192	1200	NLNESLIDL	0.8952
A02	HLA-A*02:69	1220	1228	FIAGLIAIV	0.9533
A02	HLA-A*02:70	109	117	TLDSKTQSL	0.6782
A02	HLA-A*02:70	133	141	FQFCNDPFL	0.518
A02	HLA-A*02:70	269	277	YLQPRTFLL	0.973
A02	HLA-A*02:70	386	395	KLNDLCFTNV	0.7108
A02	HLA-A*02:70	417	425	KIADYNYKL	0.909
A02	HLA-A*02:70	424	433	KLPDDFTGCV	0.5916
A02	HLA-A*02:70	515	524	FELLHAPATV	0.6033
A02	HLA-A*02:70	610	620	VLYQDVNCTEV	0.5092
A02	HLA-A*02:70	691	699	SIIAYTMSL	0.7995
A02	HLA-A*02:70	718	726	FTISVTTEI	0.5257
A02	HLA-A*02:70	821	829	LLFNKVTLA	0.7857
A02	HLA-A*02:70	857	865	GLTVLPPLL	0.6786
A02	HLA-A*02:70	915	923	VLYENQKLI	0.5378
A02	HLA-A*02:70	975	984	SVLNDILSRL	0.5124
A02	HLA-A*02:70	976	984	VLNDILSRL	0.9507
A02	HLA-A*02:70	983	991	RLDKVEAEV	0.8609
A02	HLA-A*02:70	1000	1008	RLQSLQTYV	0.7431
A02	HLA-A*02:70	1047	1056	YHLMSFPQSA	0.5452
A02	HLA-A*02:70	1048	1056	HLMSFPQSA	0.7385
A02	HLA-A*02:70	1060	1068	VVFLHVTYV	0.6948
A02	HLA-A*02:70	1185	1193	RLNEVAKNL	0.6189
A02	HLA-A*02:70	1192	1200	NLNESLIDL	0.6972

A02	HLA-A*02:70	1220	1228	FIAGLIAIV	0.8207
A02	HLA-A*02:71	109	117	TLDSKTQSL	0.6782
A02	HLA-A*02:71	133	141	FQFCNDPFL	0.518
A02	HLA-A*02:71	269	277	YLQPRTFLL	0.973
A02	HLA-A*02:71	386	395	KLNDLCFTNV	0.7108
A02	HLA-A*02:71	417	425	KIADYNYKL	0.909
A02	HLA-A*02:71	424	433	KLPDDFTGCV	0.5916
A02	HLA-A*02:71	515	524	FELLHAPATV	0.6033
A02	HLA-A*02:71	610	620	VLYQDVNCTEV	0.5092
A02	HLA-A*02:71	691	699	SIIAYTMSL	0.7995
A02	HLA-A*02:71	718	726	FTISVTTEI	0.5257
A02	HLA-A*02:71	821	829	LLFNKVTLA	0.7857
A02	HLA-A*02:71	857	865	GLTVLPPLL	0.6786
A02	HLA-A*02:71	915	923	VLYENQKLI	0.5378
A02	HLA-A*02:71	975	984	SVLNDILSRL	0.5124
A02	HLA-A*02:71	976	984	VLNDILSRL	0.9507
A02	HLA-A*02:71	983	991	RLDKVEAEV	0.8609
A02	HLA-A*02:71	1000	1008	RLQSLQTYV	0.7431
A02	HLA-A*02:71	1047	1056	YHLMSFPQSA	0.5452
A02	HLA-A*02:71	1048	1056	HLMSFPQSA	0.7385
A02	HLA-A*02:71	1060	1068	VVFLHVTYV	0.6948
A02	HLA-A*02:71	1185	1193	RLNEVAKNL	0.6189
A02	HLA-A*02:71	1192	1200	NLNESLIDL	0.6972
A02	HLA-A*02:71	1220	1228	FIAGLIAIV	0.8207
A02	HLA-A*02:72	133	141	FQFCNDPFL	0.7104
A02	HLA-A*02:72	133	143	FQFCNDPFLGV	0.5205
A02	HLA-A*02:72	269	277	YLQPRTFLL	0.8955
A02	HLA-A*02:72	417	425	KIADYNYKL	0.9064
A02	HLA-A*02:72	424	433	KLPDDFTGCV	0.5686
A02	HLA-A*02:72	612	620	YQDVNCTEV	0.5256
A02	HLA-A*02:72	691	699	SIIAYTMSL	0.847
A02	HLA-A*02:72	712	720	IAIPTNFTI	0.5224
A02	HLA-A*02:72	718	726	FTISVTTEI	0.8184
A02	HLA-A*02:72	721	729	SVTTEILPV	0.5642
A02	HLA-A*02:72	821	829	LLFNKVTLA	0.5419
A02	HLA-A*02:72	894	902	LQIPFAMQM	0.6147
A02	HLA-A*02:72	976	984	VLNDILSRL	0.8346
A02	HLA-A*02:72	983	991	RLDKVEAEV	0.6135
A02	HLA-A*02:72	1048	1056	HLMSFPQSA	0.5289
A02	HLA-A*02:72	1060	1068	VVFLHVTYV	0.7813
A02	HLA-A*02:72	1136	1145	TVYDPLQPEL	0.5968
A02	HLA-A*02:72	1220	1228	FIAGLIAIV	0.81
A02	HLA-A*02:74	109	117	TLDSKTQSL	0.6782
A02	HLA-A*02:74	133	141	FQFCNDPFL	0.518
A02	HLA-A*02:74	269	277	YLQPRTFLL	0.973
A02	HLA-A*02:74	386	395	KLNDLCFTNV	0.7108
A02	HLA-A*02:74	417	425	KIADYNYKL	0.909
A02	HLA-A*02:74	424	433	KLPDDFTGCV	0.5916
A02	HLA-A*02:74	515	524	FELLHAPATV	0.6033
A02	HLA-A*02:74	610	620	VLYQDVNCTEV	0.5092

A02	HLA-A*02:74	691	699	SIIAYTMSL	0.7995
A02	HLA-A*02:74	718	726	FTISVTTEI	0.5257
A02	HLA-A*02:74	821	829	LLFNKVTLA	0.7857
A02	HLA-A*02:74	857	865	GLTVLPPLL	0.6786
A02	HLA-A*02:74	915	923	VLYENQKLI	0.5378
A02	HLA-A*02:74	975	984	SVLNDILSRL	0.5124
A02	HLA-A*02:74	976	984	VLNDILSRL	0.9507
A02	HLA-A*02:74	983	991	RLDKVEAEV	0.8609
A02	HLA-A*02:74	1000	1008	RLQSLQTYV	0.7431
A02	HLA-A*02:74	1047	1056	YHLMSFPQSA	0.5452
A02	HLA-A*02:74	1048	1056	HLMSFPQSA	0.7385
A02	HLA-A*02:74	1060	1068	VVFLHVTYV	0.6948
A02	HLA-A*02:74	1185	1193	RLNEVAKNL	0.6189
A02	HLA-A*02:74	1192	1200	NLNESLIDL	0.6972
A02	HLA-A*02:74	1220	1228	FIAGLIAIV	0.8207
A02	HLA-A*02:75	109	117	TLDSKTQSL	0.6782
A02	HLA-A*02:75	133	141	FQFCNDPFL	0.518
A02	HLA-A*02:75	269	277	YLQPRTFLL	0.973
A02	HLA-A*02:75	386	395	KLNDLCFTNV	0.7108
A02	HLA-A*02:75	417	425	KIADYNYKL	0.909
A02	HLA-A*02:75	424	433	KLPDDFTGCV	0.5916
A02	HLA-A*02:75	515	524	FELLHAPATV	0.6033
A02	HLA-A*02:75	610	620	VLYQDVNCTEV	0.5092
A02	HLA-A*02:75	691	699	SIIAYTMSL	0.7995
A02	HLA-A*02:75	718	726	FTISVTTEI	0.5257
A02	HLA-A*02:75	821	829	LLFNKVTLA	0.7857
A02	HLA-A*02:75	857	865	GLTVLPPLL	0.6786
A02	HLA-A*02:75	915	923	VLYENQKLI	0.5378
A02	HLA-A*02:75	975	984	SVLNDILSRL	0.5124
A02	HLA-A*02:75	976	984	VLNDILSRL	0.9507
A02	HLA-A*02:75	983	991	RLDKVEAEV	0.8609
A02	HLA-A*02:75	1000	1008	RLQSLQTYV	0.7431
A02	HLA-A*02:75	1047	1056	YHLMSFPQSA	0.5452
A02	HLA-A*02:75	1048	1056	HLMSFPQSA	0.7385
A02	HLA-A*02:75	1060	1068	VVFLHVTYV	0.6948
A02	HLA-A*02:75	1185	1193	RLNEVAKNL	0.6189
A02	HLA-A*02:75	1192	1200	NLNESLIDL	0.6972
A02	HLA-A*02:75	1220	1228	FIAGLIAIV	0.8207
A02	HLA-A*02:77	109	117	TLDSKTQSL	0.6782
A02	HLA-A*02:77	133	141	FQFCNDPFL	0.518
A02	HLA-A*02:77	269	277	YLQPRTFLL	0.973
A02	HLA-A*02:77	386	395	KLNDLCFTNV	0.7108
A02	HLA-A*02:77	417	425	KIADYNYKL	0.909
A02	HLA-A*02:77	424	433	KLPDDFTGCV	0.5916
A02	HLA-A*02:77	515	524	FELLHAPATV	0.6033
A02	HLA-A*02:77	610	620	VLYQDVNCTEV	0.5092
A02	HLA-A*02:77	691	699	SIIAYTMSL	0.7995
A02	HLA-A*02:77	718	726	FTISVTTEI	0.5257
A02	HLA-A*02:77	821	829	LLFNKVTLA	0.7857
A02	HLA-A*02:77	857	865	GLTVLPPLL	0.6786

A02	HLA-A*02:77	915	923	VLYENQKLI	0.5378
A02	HLA-A*02:77	975	984	SVLNDILSRL	0.5124
A02	HLA-A*02:77	976	984	VLNDILSRL	0.9507
A02	HLA-A*02:77	983	991	RLDKVEAEV	0.8609
A02	HLA-A*02:77	1000	1008	RLQSLQTYV	0.7431
A02	HLA-A*02:77	1047	1056	YHLMSFPQSA	0.5452
A02	HLA-A*02:77	1048	1056	HLMSFPQSA	0.7385
A02	HLA-A*02:77	1060	1068	VVFLHVTYV	0.6948
A02	HLA-A*02:77	1185	1193	RLNEVAKNL	0.6189
A02	HLA-A*02:77	1192	1200	NLNESLIDL	0.6972
A02	HLA-A*02:77	1220	1228	FIAGLIAIV	0.8207
A02	HLA-A*02:78	28	36	YTNSFTRGV	0.6301
A02	HLA-A*02:78	62	70	VTWFHAIHV	0.7122
A02	HLA-A*02:78	269	277	YLQPRTFLL	0.5176
A02	HLA-A*02:78	417	425	KIADYNYKL	0.8511
A02	HLA-A*02:78	634	642	RVYSTGSNV	0.6719
A02	HLA-A*02:78	691	699	SIIAYTMSL	0.7755
A02	HLA-A*02:78	712	720	IAIPTNFTI	0.5958
A02	HLA-A*02:78	718	726	FTISVTTEI	0.6858
A02	HLA-A*02:78	721	729	SVTTEILPV	0.5092
A02	HLA-A*02:78	777	785	NTQEVFAQV	0.5878
A02	HLA-A*02:78	1060	1068	VVFLHVTYV	0.7321
A02	HLA-A*02:78	1136	1145	TVYDPLQPEL	0.5843
A02	HLA-A*02:78	1171	1179	GINASVVNI	0.5371
A02	HLA-A*02:79	133	141	FQFCNDPFL	0.7104
A02	HLA-A*02:79	133	143	FQFCNDPFLGV	0.5205
A02	HLA-A*02:79	269	277	YLQPRTFLL	0.8955
A02	HLA-A*02:79	417	425	KIADYNYKL	0.9064
A02	HLA-A*02:79	424	433	KLPDDFTGCV	0.5686
A02	HLA-A*02:79	612	620	YQDVNCTEV	0.5256
A02	HLA-A*02:79	691	699	SIIAYTMSL	0.847
A02	HLA-A*02:79	712	720	IAIPTNFTI	0.5224
A02	HLA-A*02:79	718	726	FTISVTTEI	0.8184
A02	HLA-A*02:79	721	729	SVTTEILPV	0.5642
A02	HLA-A*02:79	821	829	LLFNKVTLA	0.5419
A02	HLA-A*02:79	894	902	LQIPFAMQM	0.6147
A02	HLA-A*02:79	976	984	VLNDILSRL	0.8346
A02	HLA-A*02:79	983	991	RLDKVEAEV	0.6135
A02	HLA-A*02:79	1048	1056	HLMSFPQSA	0.5289
A02	HLA-A*02:79	1060	1068	VVFLHVTYV	0.7813
A02	HLA-A*02:79	1136	1145	TVYDPLQPEL	0.5968
A02	HLA-A*02:79	1220	1228	FIAGLIAIV	0.81
A02	HLA-A*02:85	109	117	TLDSKTQSL	0.6782
A02	HLA-A*02:85	133	141	FQFCNDPFL	0.518
A02	HLA-A*02:85	269	277	YLQPRTFLL	0.973
A02	HLA-A*02:85	386	395	KLNDLCFTNV	0.7108
A02	HLA-A*02:85	417	425	KIADYNYKL	0.909
A02	HLA-A*02:85	424	433	KLPDDFTGCV	0.5916
A02	HLA-A*02:85	515	524	FELLHAPATV	0.6033
A02	HLA-A*02:85	610	620	VLYQDVNCTEV	0.5092

A02	HLA-A*02:85	691	699	SIIAYTMSL	0.7995
A02	HLA-A*02:85	718	726	FTISVTTEI	0.5257
A02	HLA-A*02:85	821	829	LLFNKVTLA	0.7857
A02	HLA-A*02:85	857	865	GLTVLPPLL	0.6786
A02	HLA-A*02:85	915	923	VLYENQKLI	0.5378
A02	HLA-A*02:85	975	984	SVLNDILSRL	0.5124
A02	HLA-A*02:85	976	984	VLNDILSRL	0.9507
A02	HLA-A*02:85	983	991	RLDKVEAEV	0.8609
A02	HLA-A*02:85	1000	1008	RLQSLQTYV	0.7431
A02	HLA-A*02:85	1047	1056	YHLMSFPQSA	0.5452
A02	HLA-A*02:85	1048	1056	HLMSFPQSA	0.7385
A02	HLA-A*02:85	1060	1068	VVFLHVTYV	0.6948
A02	HLA-A*02:85	1185	1193	RLNEVAKNL	0.6189
A02	HLA-A*02:85	1192	1200	NLNESLIDL	0.6972
A02	HLA-A*02:85	1220	1228	FIAGLIAIV	0.8207
A02	HLA-A*02:86	109	117	TLDSKTQSL	0.6782
A02	HLA-A*02:86	133	141	FQFCNDPFL	0.518
A02	HLA-A*02:86	269	277	YLQPRTFLL	0.973
A02	HLA-A*02:86	386	395	KLNDLCFTNV	0.7108
A02	HLA-A*02:86	417	425	KIADYNYKL	0.909
A02	HLA-A*02:86	424	433	KLPDDFTGCV	0.5916
A02	HLA-A*02:86	515	524	FELLHAPATV	0.6033
A02	HLA-A*02:86	610	620	VLYQDVNCTEV	0.5092
A02	HLA-A*02:86	691	699	SIIAYTMSL	0.7995
A02	HLA-A*02:86	718	726	FTISVTTEI	0.5257
A02	HLA-A*02:86	821	829	LLFNKVTLA	0.7857
A02	HLA-A*02:86	857	865	GLTVLPPLL	0.6786
A02	HLA-A*02:86	915	923	VLYENQKLI	0.5378
A02	HLA-A*02:86	975	984	SVLNDILSRL	0.5124
A02	HLA-A*02:86	976	984	VLNDILSRL	0.9507
A02	HLA-A*02:86	983	991	RLDKVEAEV	0.8609
A02	HLA-A*02:86	1000	1008	RLQSLQTYV	0.7431
A02	HLA-A*02:86	1047	1056	YHLMSFPQSA	0.5452
A02	HLA-A*02:86	1048	1056	HLMSFPQSA	0.7385
A02	HLA-A*02:86	1060	1068	VVFLHVTYV	0.6948
A02	HLA-A*02:86	1185	1193	RLNEVAKNL	0.6189
A02	HLA-A*02:86	1192	1200	NLNESLIDL	0.6972
A02	HLA-A*02:86	1220	1228	FIAGLIAIV	0.8207
A02	HLA-A*68:02	28	36	YTNSFTRGV	0.845
A02	HLA-A*68:02	122	130	NATNVVIKV	0.7315
A02	HLA-A*68:02	258	267	WTAGAAAYYV	0.5761
A02	HLA-A*68:02	259	267	TAGAAAYYV	0.5379
A02	HLA-A*68:02	340	348	EVFNATRFA	0.7317
A02	HLA-A*68:02	394	402	NVYADSFVI	0.6993
A02	HLA-A*68:02	495	503	YGFQPTNGV	0.5183
A02	HLA-A*68:02	568	576	DIADTTDAV	0.7805
A02	HLA-A*68:02	603	611	NTSNQVAVL	0.5558
A02	HLA-A*68:02	691	699	SIIAYTMSL	0.6292
A02	HLA-A*68:02	704	712	SVAYSNNSI	0.7306
A02	HLA-A*68:02	717	726	NFTISVTTEI	0.6193

A02	HLA-A*68:02	718	726	FTISVTTEI	0.9208
A02	HLA-A*68:02	734	742	TSVDCTMYI	0.5115
A02	HLA-A*68:02	777	785	NTQEVFAQV	0.9221
A02	HLA-A*68:02	780	788	EVFAQVKQI	0.6531
A02	HLA-A*68:02	869	877	MIAQYTSAL	0.6202
A02	HLA-A*68:02	886	894	WTFGAGAAL	0.732
A02	HLA-A*68:02	907	915	NGIGVTQNV	0.5186
A02	HLA-A*68:02	940	948	STASALGKL	0.5142
A02	HLA-A*68:02	1060	1068	VVFLHVTYV	0.6649
A02	HLA-A*68:02	1095	1104	FVSNGTHWFV	0.5041
A02	HLA-A*68:02	1128	1137	VVIGIVNNTV	0.5513
A02	HLA-A*68:02	1136	1145	TVYDPLQPEL	0.6066
A02	HLA-A*68:02	1168	1176	DISGINASV	0.8721
A02	HLA-A*68:02	1220	1228	FIAGLIAIV	0.636
A02	HLA-A*68:27	28	36	YTNSFTRGV	0.845
A02	HLA-A*68:27	122	130	NATNVVIKV	0.7315
A02	HLA-A*68:27	258	267	WTAGAAAYYV	0.5761
A02	HLA-A*68:27	259	267	TAGAAAYYV	0.5379
A02	HLA-A*68:27	340	348	EVFNATRFA	0.7317
A02	HLA-A*68:27	394	402	NVYADSFVI	0.6993
A02	HLA-A*68:27	495	503	YGFQPTNGV	0.5183
A02	HLA-A*68:27	568	576	DIADTTDAV	0.7805
A02	HLA-A*68:27	603	611	NTSNQVAVL	0.5558
A02	HLA-A*68:27	691	699	SIIAYTMSL	0.6292
A02	HLA-A*68:27	704	712	SVAYSNNSI	0.7306
A02	HLA-A*68:27	717	726	NFTISVTTEI	0.6193
A02	HLA-A*68:27	718	726	FTISVTTEI	0.9208
A02	HLA-A*68:27	734	742	TSVDCTMYI	0.5115
A02	HLA-A*68:27	777	785	NTQEVFAQV	0.9221
A02	HLA-A*68:27	780	788	EVFAQVKQI	0.6531
A02	HLA-A*68:27	869	877	MIAQYTSAL	0.6202
A02	HLA-A*68:27	886	894	WTFGAGAAL	0.732
A02	HLA-A*68:27	907	915	NGIGVTQNV	0.5186
A02	HLA-A*68:27	940	948	STASALGKL	0.5142
A02	HLA-A*68:27	1060	1068	VVFLHVTYV	0.6649
A02	HLA-A*68:27	1095	1104	FVSNGTHWFV	0.5041
A02	HLA-A*68:27	1128	1137	VVIGIVNNTV	0.5513
A02	HLA-A*68:27	1136	1145	TVYDPLQPEL	0.6066
A02	HLA-A*68:27	1168	1176	DISGINASV	0.8721
A02	HLA-A*68:27	1220	1228	FIAGLIAIV	0.636
A02	HLA-A*68:28	28	36	YTNSFTRGV	0.5476
A02	HLA-A*68:28	568	576	DIADTTDAV	0.6854
A02	HLA-A*68:28	718	726	FTISVTTEI	0.6638
A02	HLA-A*68:28	777	785	NTQEVFAQV	0.7667
A02	HLA-A*68:28	1168	1176	DISGINASV	0.5957
A02	HLA-A*69:01	28	36	YTNSFTRGV	0.6754
A02	HLA-A*69:01	62	70	VTWFHAIHV	0.5639
A02	HLA-A*69:01	122	130	NATNVVIKV	0.6388
A02	HLA-A*69:01	394	402	NVYADSFVI	0.702
A02	HLA-A*69:01	568	576	DIADTTDAV	0.62

A02	HLA-A*69:01	691	699	SIIAYTMSL	0.6455
A02	HLA-A*69:01	718	726	FTISVTTEI	0.8233
A02	HLA-A*69:01	777	785	NTQEVFAQV	0.8811
A02	HLA-A*69:01	780	788	EVFAQVKQI	0.5161
A02	HLA-A*69:01	886	894	WTFGAGAAL	0.6113
A02	HLA-A*69:01	1060	1068	VVFLHVTYV	0.6659
A02	HLA-A*69:01	1136	1145	TVYDPLQPEL	0.6066
A02	HLA-A*69:01	1168	1176	DISGINASV	0.7179
A02	HLA-A*69:01	1220	1228	FIAGLIAIV	0.5133
A03	HLA-A*03:01	35	44	GVYYPDKVFR	0.568
A03	HLA-A*03:01	41	49	KVFRSSVLH	0.6944
A03	HLA-A*03:01	89	97	GVYFASTEK	0.9487
A03	HLA-A*03:01	142	150	GVYYHKNNK	0.8871
A03	HLA-A*03:01	269	278	YLQPRTFLLK	0.5577
A03	HLA-A*03:01	302	310	TLKSFTVEK	0.8988
A03	HLA-A*03:01	311	319	GIYQTSNFR	0.6304
A03	HLA-A*03:01	349	357	SVYAWNRKR	0.6333
A03	HLA-A*03:01	367	378	VLYNSASFSTFK	0.5358
A03	HLA-A*03:01	378	386	KCYGVSPTK	0.5631
A03	HLA-A*03:01	408	417	RQIAPGQTGK	0.8957
A03	HLA-A*03:01	409	417	QIAPGQTGK	0.6982
A03	HLA-A*03:01	454	462	RLFRKSNLK	0.9589
A03	HLA-A*03:01	529	537	KSTNLVKNK	0.5122
A03	HLA-A*03:01	724	733	TEILPVSMTK	0.6838
A03	HLA-A*03:01	786	795	KQIYKTPPIK	0.8503
A03	HLA-A*03:01	787	795	QIYKTPPIK	0.8934
A03	HLA-A*03:01	805	814	ILPDPSKPSK	0.5588
A03	HLA-A*03:01	826	835	VTLADAGFIK	0.5395
A03	HLA-A*03:01	827	835	TLADAGFIK	0.7267
A03	HLA-A*03:01	924	933	ANQFNSAIGK	0.5091
A03	HLA-A*03:01	939	947	SSTASALGK	0.5175
A03	HLA-A*03:01	1019	1028	RASANLAATK	0.5521
A03	HLA-A*03:01	1020	1028	ASANLAATK	0.7894
A03	HLA-A*03:01	1064	1073	HVTYVPAQEK	0.6951
A03	HLA-A*03:01	1065	1073	VTYVPAQEK	0.9087
A03	HLA-A*03:01	1099	1107	GTHWFVTQR	0.5011
A03	HLA-A*03:01	1196	1205	SLIDLQELGK	0.6419
A03	HLA-A*03:01	1264	1272	VLKGVKLHY	0.6718
A03	HLA-A*03:02	35	44	GVYYPDKVFR	0.6162
A03	HLA-A*03:02	89	97	GVYFASTEK	0.8282
A03	HLA-A*03:02	142	150	GVYYHKNNK	0.6008
A03	HLA-A*03:02	269	278	YLQPRTFLLK	0.5505
A03	HLA-A*03:02	292	300	ALDPLSETK	0.7332
A03	HLA-A*03:02	302	310	TLKSFTVEK	0.8355
A03	HLA-A*03:02	311	319	GIYQTSNFR	0.6485
A03	HLA-A*03:02	349	357	SVYAWNRKR	0.5515
A03	HLA-A*03:02	408	417	RQIAPGQTGK	0.607
A03	HLA-A*03:02	454	462	RLFRKSNLK	0.8224
A03	HLA-A*03:02	724	733	TEILPVSMTK	0.5896
A03	HLA-A*03:02	725	733	EILPVSMTK	0.5252

A03	HLA-A*03:02	787	795	QIYKTPPIK	0.6603
A03	HLA-A*03:02	805	814	ILPDPSKPSK	0.52
A03	HLA-A*03:02	826	835	VTLADAGFIK	0.5723
A03	HLA-A*03:02	827	835	TLADAGFIK	0.8003
A03	HLA-A*03:02	975	983	SVLNDILSR	0.6428
A03	HLA-A*03:02	1020	1028	ASANLAATK	0.6797
A03	HLA-A*03:02	1065	1073	VTYVPAQEK	0.744
A03	HLA-A*03:02	1099	1107	GTHWFVTQR	0.5647
A03	HLA-A*03:02	1196	1205	SLIDLQELGK	0.5484
A03	HLA-A*03:04	35	44	GVYYPDKVFR	0.568
A03	HLA-A*03:04	41	49	KVFRSSVLH	0.6944
A03	HLA-A*03:04	89	97	GVYFASTEK	0.9487
A03	HLA-A*03:04	142	150	GVYYHKNNK	0.8871
A03	HLA-A*03:04	269	278	YLQPRTFLLK	0.5577
A03	HLA-A*03:04	302	310	TLKSFTVEK	0.8988
A03	HLA-A*03:04	311	319	GIYQTSNFR	0.6304
A03	HLA-A*03:04	349	357	SVYAWNRKR	0.6333
A03	HLA-A*03:04	367	378	VLYNSASFSTFK	0.5358
A03	HLA-A*03:04	378	386	KCYGVSPTK	0.5631
A03	HLA-A*03:04	408	417	RQIAPGQTGK	0.8957
A03	HLA-A*03:04	409	417	QIAPGQTGK	0.6982
A03	HLA-A*03:04	454	462	RLFRKSNLK	0.9589
A03	HLA-A*03:04	529	537	KSTNLVKNK	0.5122
A03	HLA-A*03:04	724	733	TEILPVSMTK	0.6838
A03	HLA-A*03:04	786	795	KQIYKTPPIK	0.8503
A03	HLA-A*03:04	787	795	QIYKTPPIK	0.8934
A03	HLA-A*03:04	805	814	ILPDPSKPSK	0.5588
A03	HLA-A*03:04	826	835	VTLADAGFIK	0.5395
A03	HLA-A*03:04	827	835	TLADAGFIK	0.7267
A03	HLA-A*03:04	924	933	ANQFNSAIGK	0.5091
A03	HLA-A*03:04	939	947	SSTASALGK	0.5175
A03	HLA-A*03:04	1019	1028	RASANLAATK	0.5521
A03	HLA-A*03:04	1020	1028	ASANLAATK	0.7894
A03	HLA-A*03:04	1064	1073	HVTYVPAQEK	0.6951
A03	HLA-A*03:04	1065	1073	VTYVPAQEK	0.9087
A03	HLA-A*03:04	1099	1107	GTHWFVTQR	0.5011
A03	HLA-A*03:04	1196	1205	SLIDLQELGK	0.6419
A03	HLA-A*03:04	1264	1272	VLKGVKLHY	0.6718
A03	HLA-A*03:05	35	44	GVYYPDKVFR	0.568
A03	HLA-A*03:05	41	49	KVFRSSVLH	0.6944
A03	HLA-A*03:05	89	97	GVYFASTEK	0.9487
A03	HLA-A*03:05	142	150	GVYYHKNNK	0.8871
A03	HLA-A*03:05	269	278	YLQPRTFLLK	0.5577
A03	HLA-A*03:05	302	310	TLKSFTVEK	0.8988
A03	HLA-A*03:05	311	319	GIYQTSNFR	0.6304
A03	HLA-A*03:05	349	357	SVYAWNRKR	0.6333
A03	HLA-A*03:05	367	378	VLYNSASFSTFK	0.5358
A03	HLA-A*03:05	378	386	KCYGVSPTK	0.5631
A03	HLA-A*03:05	408	417	RQIAPGQTGK	0.8957
A03	HLA-A*03:05	409	417	QIAPGQTGK	0.6982

A03	HLA-A*03:05	454	462	RLFRKSNLK	0.9589
A03	HLA-A*03:05	529	537	KSTNLVKNK	0.5122
A03	HLA-A*03:05	724	733	TEILPVSMTK	0.6838
A03	HLA-A*03:05	786	795	KQIYKTPPIK	0.8503
A03	HLA-A*03:05	787	795	QIYKTPPIK	0.8934
A03	HLA-A*03:05	805	814	ILPDPSKPSK	0.5588
A03	HLA-A*03:05	826	835	VTLADAGFIK	0.5395
A03	HLA-A*03:05	827	835	TLADAGFIK	0.7267
A03	HLA-A*03:05	924	933	ANQFNSAIGK	0.5091
A03	HLA-A*03:05	939	947	SSTASALGK	0.5175
A03	HLA-A*03:05	1019	1028	RASANLAATK	0.5521
A03	HLA-A*03:05	1020	1028	ASANLAATK	0.7894
A03	HLA-A*03:05	1064	1073	HVTYVPAQEK	0.6951
A03	HLA-A*03:05	1065	1073	VTYVPAQEK	0.9087
A03	HLA-A*03:05	1099	1107	GTHWFVTQR	0.5011
A03	HLA-A*03:05	1196	1205	SLIDLQELGK	0.6419
A03	HLA-A*03:05	1264	1272	VLKGVKLHY	0.6718
A03	HLA-A*03:06	35	44	GVYYPDKVFR	0.568
A03	HLA-A*03:06	41	49	KVFRSSVLH	0.6944
A03	HLA-A*03:06	89	97	GVYFASTEK	0.9487
A03	HLA-A*03:06	142	150	GVYYHKNNK	0.8871
A03	HLA-A*03:06	269	278	YLQPRTFLLK	0.5577
A03	HLA-A*03:06	302	310	TLKSFTVEK	0.8988
A03	HLA-A*03:06	311	319	GIYQTSNFR	0.6304
A03	HLA-A*03:06	349	357	SVYAWNRKR	0.6333
A03	HLA-A*03:06	367	378	VLYNSASFSTFK	0.5358
A03	HLA-A*03:06	378	386	KCYGVSPTK	0.5631
A03	HLA-A*03:06	408	417	RQIAPGQTGK	0.8957
A03	HLA-A*03:06	409	417	QIAPGQTGK	0.6982
A03	HLA-A*03:06	454	462	RLFRKSNLK	0.9589
A03	HLA-A*03:06	529	537	KSTNLVKNK	0.5122
A03	HLA-A*03:06	724	733	TEILPVSMTK	0.6838
A03	HLA-A*03:06	786	795	KQIYKTPPIK	0.8503
A03	HLA-A*03:06	787	795	QIYKTPPIK	0.8934
A03	HLA-A*03:06	805	814	ILPDPSKPSK	0.5588
A03	HLA-A*03:06	826	835	VTLADAGFIK	0.5395
A03	HLA-A*03:06	827	835	TLADAGFIK	0.7267
A03	HLA-A*03:06	924	933	ANQFNSAIGK	0.5091
A03	HLA-A*03:06	939	947	SSTASALGK	0.5175
A03	HLA-A*03:06	1019	1028	RASANLAATK	0.5521
A03	HLA-A*03:06	1020	1028	ASANLAATK	0.7894
A03	HLA-A*03:06	1064	1073	HVTYVPAQEK	0.6951
A03	HLA-A*03:06	1065	1073	VTYVPAQEK	0.9087
A03	HLA-A*03:06	1099	1107	GTHWFVTQR	0.5011
A03	HLA-A*03:06	1196	1205	SLIDLQELGK	0.6419
A03	HLA-A*03:06	1264	1272	VLKGVKLHY	0.6718
A03	HLA-A*03:07	35	44	GVYYPDKVFR	0.7348
A03	HLA-A*03:07	89	97	GVYFASTEK	0.8648
A03	HLA-A*03:07	142	150	GVYYHKNNK	0.6702
A03	HLA-A*03:07	292	300	ALDPLSETK	0.6955

A03	HLA-A*03:07	302	310	TLKSFTVEK	0.8407
A03	HLA-A*03:07	311	319	GIYQTSNFR	0.7033
A03	HLA-A*03:07	349	357	SVYAWNRKR	0.6804
A03	HLA-A*03:07	370	378	NSASFSTFK	0.5695
A03	HLA-A*03:07	408	417	RQIAPGQTGK	0.6507
A03	HLA-A*03:07	454	462	RLFRKSNLK	0.8435
A03	HLA-A*03:07	458	466	KSNLKPFER	0.5539
A03	HLA-A*03:07	724	733	TEILPVSMTK	0.6479
A03	HLA-A*03:07	725	733	EILPVSMTK	0.6008
A03	HLA-A*03:07	786	795	KQIYKTPPIK	0.5627
A03	HLA-A*03:07	787	795	QIYKTPPIK	0.757
A03	HLA-A*03:07	805	814	ILPDPSKPSK	0.5158
A03	HLA-A*03:07	826	835	VTLADAGFIK	0.6103
A03	HLA-A*03:07	827	835	TLADAGFIK	0.8004
A03	HLA-A*03:07	975	983	SVLNDILSR	0.744
A03	HLA-A*03:07	1020	1028	ASANLAATK	0.7263
A03	HLA-A*03:07	1064	1073	HVTYVPAQEK	0.5071
A03	HLA-A*03:07	1065	1073	VTYVPAQEK	0.8254
A03	HLA-A*03:07	1099	1107	GTHWFVTQR	0.6685
A03	HLA-A*03:07	1196	1205	SLIDLQELGK	0.6149
A03	HLA-A*03:08	35	44	GVYYPDKVFR	0.5777
A03	HLA-A*03:08	41	49	KVFRSSVLH	0.5336
A03	HLA-A*03:08	89	97	GVYFASTEK	0.8234
A03	HLA-A*03:08	142	150	GVYYHKNNK	0.7338
A03	HLA-A*03:08	302	310	TLKSFTVEK	0.7823
A03	HLA-A*03:08	311	319	GIYQTSNFR	0.623
A03	HLA-A*03:08	349	357	SVYAWNRKR	0.6585
A03	HLA-A*03:08	408	417	RQIAPGQTGK	0.7314
A03	HLA-A*03:08	454	462	RLFRKSNLK	0.9008
A03	HLA-A*03:08	786	795	KQIYKTPPIK	0.6142
A03	HLA-A*03:08	787	795	QIYKTPPIK	0.679
A03	HLA-A*03:08	1020	1028	ASANLAATK	0.5793
A03	HLA-A*03:08	1065	1073	VTYVPAQEK	0.7028
A03	HLA-A*03:08	1099	1107	GTHWFVTQR	0.5336
A03	HLA-A*03:10	35	44	GVYYPDKVFR	0.6162
A03	HLA-A*03:10	89	97	GVYFASTEK	0.8282
A03	HLA-A*03:10	142	150	GVYYHKNNK	0.6008
A03	HLA-A*03:10	269	278	YLQPRTFLLK	0.5505
A03	HLA-A*03:10	292	300	ALDPLSETK	0.7332
A03	HLA-A*03:10	302	310	TLKSFTVEK	0.8355
A03	HLA-A*03:10	311	319	GIYQTSNFR	0.6485
A03	HLA-A*03:10	349	357	SVYAWNRKR	0.5515
A03	HLA-A*03:10	408	417	RQIAPGQTGK	0.607
A03	HLA-A*03:10	454	462	RLFRKSNLK	0.8224
A03	HLA-A*03:10	724	733	TEILPVSMTK	0.5896
A03	HLA-A*03:10	725	733	EILPVSMTK	0.5252
A03	HLA-A*03:10	787	795	QIYKTPPIK	0.6603
A03	HLA-A*03:10	805	814	ILPDPSKPSK	0.52
A03	HLA-A*03:10	826	835	VTLADAGFIK	0.5723
A03	HLA-A*03:10	827	835	TLADAGFIK	0.8003

A03	HLA-A*03:10	975	983	SVLNDILSR	0.6428
A03	HLA-A*03:10	1020	1028	ASANLAATK	0.6797
A03	HLA-A*03:10	1065	1073	VTYVPAQEK	0.744
A03	HLA-A*03:10	1099	1107	GTHWFVTQR	0.5647
A03	HLA-A*03:10	1196	1205	SLIDLQELGK	0.5484
A03	HLA-A*03:12	35	44	GVYYPDKVFR	0.6548
A03	HLA-A*03:12	41	49	KVFRSSVLH	0.7788
A03	HLA-A*03:12	69	77	HVSGTNGTK	0.5995
A03	HLA-A*03:12	89	97	GVYFASTEK	0.9495
A03	HLA-A*03:12	142	150	GVYYHKNNK	0.8976
A03	HLA-A*03:12	302	310	TLKSFTVEK	0.8641
A03	HLA-A*03:12	311	319	GIYQTSNFR	0.6653
A03	HLA-A*03:12	348	356	ASVYAWNRK	0.6546
A03	HLA-A*03:12	349	357	SVYAWNRKR	0.7468
A03	HLA-A*03:12	370	378	NSASFSTFK	0.6426
A03	HLA-A*03:12	375	386	STFKCYGVSPTK	0.5581
A03	HLA-A*03:12	378	386	KCYGVSPTK	0.6783
A03	HLA-A*03:12	408	417	RQIAPGQTGK	0.8931
A03	HLA-A*03:12	409	417	QIAPGQTGK	0.7544
A03	HLA-A*03:12	454	462	RLFRKSNLK	0.9223
A03	HLA-A*03:12	458	466	KSNLKPFER	0.5408
A03	HLA-A*03:12	529	537	KSTNLVKNK	0.7666
A03	HLA-A*03:12	550	558	GVLTESNKK	0.6481
A03	HLA-A*03:12	724	733	TEILPVSMTK	0.6899
A03	HLA-A*03:12	725	733	EILPVSMTK	0.7052
A03	HLA-A*03:12	786	795	KQIYKTPPIK	0.7729
A03	HLA-A*03:12	787	795	QIYKTPPIK	0.8972
A03	HLA-A*03:12	803	811	SQILPDPSK	0.5075
A03	HLA-A*03:12	826	835	VTLADAGFIK	0.5973
A03	HLA-A*03:12	827	835	TLADAGFIK	0.6931
A03	HLA-A*03:12	845	854	AARDLICAQK	0.5375
A03	HLA-A*03:12	924	933	ANQFNSAIGK	0.679
A03	HLA-A*03:12	925	933	NQFNSAIGK	0.6061
A03	HLA-A*03:12	939	947	SSTASALGK	0.7158
A03	HLA-A*03:12	956	964	AQALNTLVK	0.5799
A03	HLA-A*03:12	975	983	SVLNDILSR	0.7042
A03	HLA-A*03:12	1019	1028	RASANLAATK	0.6895
A03	HLA-A*03:12	1020	1028	ASANLAATK	0.8887
A03	HLA-A*03:12	1064	1073	HVTYVPAQEK	0.7577
A03	HLA-A*03:12	1065	1073	VTYVPAQEK	0.9408
A03	HLA-A*03:12	1099	1107	GTHWFVTQR	0.7035
A03	HLA-A*03:12	1196	1205	SLIDLQELGK	0.5515
A03	HLA-A*03:12	1264	1272	VLKGVKLHY	0.6239
A03	HLA-A*03:13	35	44	GVYYPDKVFR	0.568
A03	HLA-A*03:13	41	49	KVFRSSVLH	0.6944
A03	HLA-A*03:13	89	97	GVYFASTEK	0.9487
A03	HLA-A*03:13	142	150	GVYYHKNNK	0.8871
A03	HLA-A*03:13	269	278	YLQPRTFLLK	0.5577
A03	HLA-A*03:13	302	310	TLKSFTVEK	0.8988
A03	HLA-A*03:13	311	319	GIYQTSNFR	0.6304

A03	HLA-A*03:13	349	357	SVYAWNRKR	0.6333
A03	HLA-A*03:13	367	378	VLYNSASFSTFK	0.5358
A03	HLA-A*03:13	378	386	KCYGVSPTK	0.5631
A03	HLA-A*03:13	408	417	RQIAPGQTGK	0.8957
A03	HLA-A*03:13	409	417	QIAPGQTGK	0.6982
A03	HLA-A*03:13	454	462	RLFRKSNLK	0.9589
A03	HLA-A*03:13	529	537	KSTNLVKNK	0.5122
A03	HLA-A*03:13	724	733	TEILPVSMTK	0.6838
A03	HLA-A*03:13	786	795	KQIYKTPPIK	0.8503
A03	HLA-A*03:13	787	795	QIYKTPPIK	0.8934
A03	HLA-A*03:13	805	814	ILPDPSKPSK	0.5588
A03	HLA-A*03:13	826	835	VTLADAGFIK	0.5395
A03	HLA-A*03:13	827	835	TLADAGFIK	0.7267
A03	HLA-A*03:13	924	933	ANQFNSAIGK	0.5091
A03	HLA-A*03:13	939	947	SSTASALGK	0.5175
A03	HLA-A*03:13	1019	1028	RASANLAATK	0.5521
A03	HLA-A*03:13	1020	1028	ASANLAATK	0.7894
A03	HLA-A*03:13	1064	1073	HVTYVPAQEK	0.6951
A03	HLA-A*03:13	1065	1073	VTYVPAQEK	0.9087
A03	HLA-A*03:13	1099	1107	GTHWFVTQR	0.5011
A03	HLA-A*03:13	1196	1205	SLIDLQELGK	0.6419
A03	HLA-A*03:13	1264	1272	VLKGVKLHY	0.6718
A03	HLA-A*03:14	35	44	GVYYPDKVFR	0.568
A03	HLA-A*03:14	41	49	KVFRSSVLH	0.6944
A03	HLA-A*03:14	89	97	GVYFASTEK	0.9487
A03	HLA-A*03:14	142	150	GVYYHKNNK	0.8871
A03	HLA-A*03:14	269	278	YLQPRTFLLK	0.5577
A03	HLA-A*03:14	302	310	TLKSFTVEK	0.8988
A03	HLA-A*03:14	311	319	GIYQTSNFR	0.6304
A03	HLA-A*03:14	349	357	SVYAWNRKR	0.6333
A03	HLA-A*03:14	367	378	VLYNSASFSTFK	0.5358
A03	HLA-A*03:14	378	386	KCYGVSPTK	0.5631
A03	HLA-A*03:14	408	417	RQIAPGQTGK	0.8957
A03	HLA-A*03:14	409	417	QIAPGQTGK	0.6982
A03	HLA-A*03:14	454	462	RLFRKSNLK	0.9589
A03	HLA-A*03:14	529	537	KSTNLVKNK	0.5122
A03	HLA-A*03:14	724	733	TEILPVSMTK	0.6838
A03	HLA-A*03:14	786	795	KQIYKTPPIK	0.8503
A03	HLA-A*03:14	787	795	QIYKTPPIK	0.8934
A03	HLA-A*03:14	805	814	ILPDPSKPSK	0.5588
A03	HLA-A*03:14	826	835	VTLADAGFIK	0.5395
A03	HLA-A*03:14	827	835	TLADAGFIK	0.7267
A03	HLA-A*03:14	924	933	ANQFNSAIGK	0.5091
A03	HLA-A*03:14	939	947	SSTASALGK	0.5175
A03	HLA-A*03:14	1019	1028	RASANLAATK	0.5521
A03	HLA-A*03:14	1020	1028	ASANLAATK	0.7894
A03	HLA-A*03:14	1064	1073	HVTYVPAQEK	0.6951
A03	HLA-A*03:14	1065	1073	VTYVPAQEK	0.9087
A03	HLA-A*03:14	1099	1107	GTHWFVTQR	0.5011
A03	HLA-A*03:14	1196	1205	SLIDLQELGK	0.6419

A03	HLA-A*03:14	1264	1272	VLKGVKLHY	0.6718
A03	HLA-A*03:16	35	44	GVYYPDKVFR	0.568
A03	HLA-A*03:16	41	49	KVFRSSVLH	0.6944
A03	HLA-A*03:16	89	97	GVYFASTEK	0.9487
A03	HLA-A*03:16	142	150	GVYYHKNNK	0.8871
A03	HLA-A*03:16	269	278	YLQPRTFLLK	0.5577
A03	HLA-A*03:16	302	310	TLKSFTVEK	0.8988
A03	HLA-A*03:16	311	319	GIYQTSNFR	0.6304
A03	HLA-A*03:16	349	357	SVYAWNRKR	0.6333
A03	HLA-A*03:16	367	378	VLYNSASFSTFK	0.5358
A03	HLA-A*03:16	378	386	KCYGVSPTK	0.5631
A03	HLA-A*03:16	408	417	RQIAPGQTGK	0.8957
A03	HLA-A*03:16	409	417	QIAPGQTGK	0.6982
A03	HLA-A*03:16	454	462	RLFRKSNLK	0.9589
A03	HLA-A*03:16	529	537	KSTNLVKNK	0.5122
A03	HLA-A*03:16	724	733	TEILPVSMTK	0.6838
A03	HLA-A*03:16	786	795	KQIYKTPPIK	0.8503
A03	HLA-A*03:16	787	795	QIYKTPPIK	0.8934
A03	HLA-A*03:16	805	814	ILPDPSKPSK	0.5588
A03	HLA-A*03:16	826	835	VTLADAGFIK	0.5395
A03	HLA-A*03:16	827	835	TLADAGFIK	0.7267
A03	HLA-A*03:16	924	933	ANQFNSAIGK	0.5091
A03	HLA-A*03:16	939	947	SSTASALGK	0.5175
A03	HLA-A*03:16	1019	1028	RASANLAATK	0.5521
A03	HLA-A*03:16	1020	1028	ASANLAATK	0.7894
A03	HLA-A*03:16	1064	1073	HVTYVPAQEK	0.6951
A03	HLA-A*03:16	1065	1073	VTYVPAQEK	0.9087
A03	HLA-A*03:16	1099	1107	GTHWFVTQR	0.5011
A03	HLA-A*03:16	1196	1205	SLIDLQELGK	0.6419
A03	HLA-A*03:16	1264	1272	VLKGVKLHY	0.6718
A03	HLA-A*03:17	35	44	GVYYPDKVFR	0.568
A03	HLA-A*03:17	41	49	KVFRSSVLH	0.6944
A03	HLA-A*03:17	89	97	GVYFASTEK	0.9487
A03	HLA-A*03:17	142	150	GVYYHKNNK	0.8871
A03	HLA-A*03:17	269	278	YLQPRTFLLK	0.5577
A03	HLA-A*03:17	302	310	TLKSFTVEK	0.8988
A03	HLA-A*03:17	311	319	GIYQTSNFR	0.6304
A03	HLA-A*03:17	349	357	SVYAWNRKR	0.6333
A03	HLA-A*03:17	367	378	VLYNSASFSTFK	0.5358
A03	HLA-A*03:17	378	386	KCYGVSPTK	0.5631
A03	HLA-A*03:17	408	417	RQIAPGQTGK	0.8957
A03	HLA-A*03:17	409	417	QIAPGQTGK	0.6982
A03	HLA-A*03:17	454	462	RLFRKSNLK	0.9589
A03	HLA-A*03:17	529	537	KSTNLVKNK	0.5122
A03	HLA-A*03:17	724	733	TEILPVSMTK	0.6838
A03	HLA-A*03:17	786	795	KQIYKTPPIK	0.8503
A03	HLA-A*03:17	787	795	QIYKTPPIK	0.8934
A03	HLA-A*03:17	805	814	ILPDPSKPSK	0.5588
A03	HLA-A*03:17	826	835	VTLADAGFIK	0.5395
A03	HLA-A*03:17	827	835	TLADAGFIK	0.7267

A03	HLA-A*03:17	924	933	ANQFNSAIGK	0.5091
A03	HLA-A*03:17	939	947	SSTASALGK	0.5175
A03	HLA-A*03:17	1019	1028	RASANLAATK	0.5521
A03	HLA-A*03:17	1020	1028	ASANLAATK	0.7894
A03	HLA-A*03:17	1064	1073	HVTYVPAQEK	0.6951
A03	HLA-A*03:17	1065	1073	VTYVPAQEK	0.9087
A03	HLA-A*03:17	1099	1107	GTHWFVTQR	0.5011
A03	HLA-A*03:17	1196	1205	SLIDLQELGK	0.6419
A03	HLA-A*03:17	1264	1272	VLKGVKLHY	0.6718
A03	HLA-A*11:01	35	44	GVYYPDKVFR	0.6555
A03	HLA-A*11:01	89	97	GVYFASTEK	0.9386
A03	HLA-A*11:01	142	150	GVYYHKNNK	0.7229
A03	HLA-A*11:01	292	300	ALDPLSETK	0.6795
A03	HLA-A*11:01	302	310	TLKSFTVEK	0.819
A03	HLA-A*11:01	311	319	GIYQTSNFR	0.5901
A03	HLA-A*11:01	348	356	ASVYAWNRK	0.6551
A03	HLA-A*11:01	349	357	SVYAWNRKR	0.576
A03	HLA-A*11:01	369	378	YNSASFSTFK	0.5561
A03	HLA-A*11:01	370	378	NSASFSTFK	0.8025
A03	HLA-A*11:01	375	386	STFKCYGVSPTK	0.6638
A03	HLA-A*11:01	408	417	RQIAPGQTGK	0.6249
A03	HLA-A*11:01	409	417	QIAPGQTGK	0.6748
A03	HLA-A*11:01	454	462	RLFRKSNLK	0.6772
A03	HLA-A*11:01	550	558	GVLTESNKK	0.7312
A03	HLA-A*11:01	686	695	SVASQSIIAY	0.5292
A03	HLA-A*11:01	723	733	TTEILPVSMTK	0.5964
A03	HLA-A*11:01	724	733	TEILPVSMTK	0.7926
A03	HLA-A*11:01	725	733	EILPVSMTK	0.7733
A03	HLA-A*11:01	787	795	QIYKTPPIK	0.7378
A03	HLA-A*11:01	825	835	KVTLADAGFIK	0.5709
A03	HLA-A*11:01	826	835	VTLADAGFIK	0.7264
A03	HLA-A*11:01	827	835	TLADAGFIK	0.8305
A03	HLA-A*11:01	925	933	NQFNSAIGK	0.5165
A03	HLA-A*11:01	939	947	SSTASALGK	0.7797
A03	HLA-A*11:01	956	964	AQALNTLVK	0.5151
A03	HLA-A*11:01	975	983	SVLNDILSR	0.8499
A03	HLA-A*11:01	975	986	SVLNDILSRLDK	0.5938
A03	HLA-A*11:01	1019	1028	RASANLAATK	0.5185
A03	HLA-A*11:01	1020	1028	ASANLAATK	0.9119
A03	HLA-A*11:01	1064	1073	HVTYVPAQEK	0.6484
A03	HLA-A*11:01	1065	1073	VTYVPAQEK	0.9153
A03	HLA-A*11:01	1099	1107	GTHWFVTQR	0.7768
A03	HLA-A*11:01	1196	1205	SLIDLQELGK	0.5993
A03	HLA-A*11:02	35	44	GVYYPDKVFR	0.6555
A03	HLA-A*11:02	89	97	GVYFASTEK	0.9386
A03	HLA-A*11:02	142	150	GVYYHKNNK	0.7229
A03	HLA-A*11:02	292	300	ALDPLSETK	0.6795
A03	HLA-A*11:02	302	310	TLKSFTVEK	0.819
A03	HLA-A*11:02	311	319	GIYQTSNFR	0.5901
A03	HLA-A*11:02	348	356	ASVYAWNRK	0.6551

A03	HLA-A*11:02	349	357	SVYAWNRKR	0.576
A03	HLA-A*11:02	369	378	YNSASFSTFK	0.5561
A03	HLA-A*11:02	370	378	NSASFSTFK	0.8025
A03	HLA-A*11:02	375	386	STFKCYGVSPTK	0.6638
A03	HLA-A*11:02	408	417	RQIAPGQTGK	0.6249
A03	HLA-A*11:02	409	417	QIAPGQTGK	0.6748
A03	HLA-A*11:02	454	462	RLFRKSNLK	0.6772
A03	HLA-A*11:02	550	558	GVLTESNKK	0.7312
A03	HLA-A*11:02	686	695	SVASQSIIAY	0.5292
A03	HLA-A*11:02	723	733	TTEILPVSMTK	0.5964
A03	HLA-A*11:02	724	733	TEILPVSMTK	0.7926
A03	HLA-A*11:02	725	733	EILPVSMTK	0.7733
A03	HLA-A*11:02	787	795	QIYKTPPIK	0.7378
A03	HLA-A*11:02	825	835	KVTLADAGFIK	0.5709
A03	HLA-A*11:02	826	835	VTLADAGFIK	0.7264
A03	HLA-A*11:02	827	835	TLADAGFIK	0.8305
A03	HLA-A*11:02	925	933	NQFNSAIGK	0.5165
A03	HLA-A*11:02	939	947	SSTASALGK	0.7797
A03	HLA-A*11:02	956	964	AQALNTLVK	0.5151
A03	HLA-A*11:02	975	983	SVLNDILSR	0.8499
A03	HLA-A*11:02	975	986	SVLNDILSRLDK	0.5938
A03	HLA-A*11:02	1019	1028	RASANLAATK	0.5185
A03	HLA-A*11:02	1020	1028	ASANLAATK	0.9119
A03	HLA-A*11:02	1064	1073	HVTYVPAQEK	0.6484
A03	HLA-A*11:02	1065	1073	VTYVPAQEK	0.9153
A03	HLA-A*11:02	1099	1107	GTHWFVTQR	0.7768
A03	HLA-A*11:02	1196	1205	SLIDLQELGK	0.5993
A03	HLA-A*11:03	35	44	GVYYPDKVFR	0.5729
A03	HLA-A*11:03	41	49	KVFRSSVLH	0.6359
A03	HLA-A*11:03	69	77	HVSGTNGTK	0.5574
A03	HLA-A*11:03	89	97	GVYFASTEK	0.9479
A03	HLA-A*11:03	142	150	GVYYHKNNK	0.8636
A03	HLA-A*11:03	257	266	GWTAGAAAYY	0.5027
A03	HLA-A*11:03	292	300	ALDPLSETK	0.6109
A03	HLA-A*11:03	302	310	TLKSFTVEK	0.8758
A03	HLA-A*11:03	311	319	GIYQTSNFR	0.6106
A03	HLA-A*11:03	348	356	ASVYAWNRK	0.6559
A03	HLA-A*11:03	349	357	SVYAWNRKR	0.6192
A03	HLA-A*11:03	369	378	YNSASFSTFK	0.5161
A03	HLA-A*11:03	370	378	NSASFSTFK	0.7603
A03	HLA-A*11:03	375	386	STFKCYGVSPTK	0.6584
A03	HLA-A*11:03	408	417	RQIAPGQTGK	0.8115
A03	HLA-A*11:03	409	417	QIAPGQTGK	0.7924
A03	HLA-A*11:03	454	462	RLFRKSNLK	0.839
A03	HLA-A*11:03	529	537	KSTNLVKNK	0.6205
A03	HLA-A*11:03	550	558	GVLTESNKK	0.7419
A03	HLA-A*11:03	686	695	SVASQSIIAY	0.5979
A03	HLA-A*11:03	723	733	TTEILPVSMTK	0.5555
A03	HLA-A*11:03	724	733	TEILPVSMTK	0.7868
A03	HLA-A*11:03	725	733	EILPVSMTK	0.7674

A03	HLA-A*11:03	786	795	KQIYKTPPIK	0.5919
A03	HLA-A*11:03	787	795	QIYKTPPIK	0.8358
A03	HLA-A*11:03	825	835	KVTLADAGFIK	0.5148
A03	HLA-A*11:03	826	835	VTLADAGFIK	0.6588
A03	HLA-A*11:03	827	835	TLADAGFIK	0.7979
A03	HLA-A*11:03	924	933	ANQFNSAIGK	0.5982
A03	HLA-A*11:03	925	933	NQFNSAIGK	0.5795
A03	HLA-A*11:03	939	947	SSTASALGK	0.7993
A03	HLA-A*11:03	956	964	AQALNTLVK	0.5795
A03	HLA-A*11:03	975	983	SVLNDILSR	0.7656
A03	HLA-A*11:03	975	986	SVLNDILSRLDK	0.5176
A03	HLA-A*11:03	1019	1028	RASANLAATK	0.6171
A03	HLA-A*11:03	1020	1028	ASANLAATK	0.9033
A03	HLA-A*11:03	1064	1073	HVTYVPAQEK	0.7064
A03	HLA-A*11:03	1065	1073	VTYVPAQEK	0.9204
A03	HLA-A*11:03	1099	1107	GTHWFVTQR	0.7038
A03	HLA-A*11:03	1196	1205	SLIDLQELGK	0.6467
A03	HLA-A*11:04	35	44	GVYYPDKVFR	0.6723
A03	HLA-A*11:04	89	97	GVYFASTEK	0.9041
A03	HLA-A*11:04	142	150	GVYYHKNNK	0.7342
A03	HLA-A*11:04	292	300	ALDPLSETK	0.5968
A03	HLA-A*11:04	302	310	TLKSFTVEK	0.8186
A03	HLA-A*11:04	311	319	GIYQTSNFR	0.6771
A03	HLA-A*11:04	319	328	RVQPTESIVR	0.5363
A03	HLA-A*11:04	348	356	ASVYAWNRK	0.5541
A03	HLA-A*11:04	349	357	SVYAWNRKR	0.646
A03	HLA-A*11:04	370	378	NSASFSTFK	0.6355
A03	HLA-A*11:04	375	386	STFKCYGVSPTK	0.5031
A03	HLA-A*11:04	408	417	RQIAPGQTGK	0.7357
A03	HLA-A*11:04	409	417	QIAPGQTGK	0.6296
A03	HLA-A*11:04	454	462	RLFRKSNLK	0.8162
A03	HLA-A*11:04	458	466	KSNLKPFER	0.6282
A03	HLA-A*11:04	529	537	KSTNLVKNK	0.5664
A03	HLA-A*11:04	550	558	GVLTESNKK	0.6125
A03	HLA-A*11:04	724	733	TEILPVSMTK	0.653
A03	HLA-A*11:04	725	733	EILPVSMTK	0.6293
A03	HLA-A*11:04	786	795	KQIYKTPPIK	0.5121
A03	HLA-A*11:04	787	795	QIYKTPPIK	0.7537
A03	HLA-A*11:04	825	835	KVTLADAGFIK	0.5571
A03	HLA-A*11:04	826	835	VTLADAGFIK	0.5926
A03	HLA-A*11:04	827	835	TLADAGFIK	0.7565
A03	HLA-A*11:04	939	947	SSTASALGK	0.6578
A03	HLA-A*11:04	956	964	AQALNTLVK	0.5269
A03	HLA-A*11:04	975	983	SVLNDILSR	0.7843
A03	HLA-A*11:04	1019	1028	RASANLAATK	0.5722
A03	HLA-A*11:04	1020	1028	ASANLAATK	0.8712
A03	HLA-A*11:04	1064	1073	HVTYVPAQEK	0.5792
A03	HLA-A*11:04	1065	1073	VTYVPAQEK	0.8811
A03	HLA-A*11:04	1099	1107	GTHWFVTQR	0.7363
A03	HLA-A*11:05	35	44	GVYYPDKVFR	0.6555

A03	HLA-A*11:05	89	97	GVYFASTEK	0.9386
A03	HLA-A*11:05	142	150	GVYYHKNNK	0.7229
A03	HLA-A*11:05	292	300	ALDPLSETK	0.6795
A03	HLA-A*11:05	302	310	TLKSFTVEK	0.819
A03	HLA-A*11:05	311	319	GIYQTSNFR	0.5901
A03	HLA-A*11:05	348	356	ASVYAWNRK	0.6551
A03	HLA-A*11:05	349	357	SVYAWNRKR	0.576
A03	HLA-A*11:05	369	378	YNSASFSTFK	0.5561
A03	HLA-A*11:05	370	378	NSASFSTFK	0.8025
A03	HLA-A*11:05	375	386	STFKCYGVSPTK	0.6638
A03	HLA-A*11:05	408	417	RQIAPGQTGK	0.6249
A03	HLA-A*11:05	409	417	QIAPGQTGK	0.6748
A03	HLA-A*11:05	454	462	RLFRKSNLK	0.6772
A03	HLA-A*11:05	550	558	GVLTESNKK	0.7312
A03	HLA-A*11:05	686	695	SVASQSIIAY	0.5292
A03	HLA-A*11:05	723	733	TTEILPVSMTK	0.5964
A03	HLA-A*11:05	724	733	TEILPVSMTK	0.7926
A03	HLA-A*11:05	725	733	EILPVSMTK	0.7733
A03	HLA-A*11:05	787	795	QIYKTPPIK	0.7378
A03	HLA-A*11:05	825	835	KVTLADAGFIK	0.5709
A03	HLA-A*11:05	826	835	VTLADAGFIK	0.7264
A03	HLA-A*11:05	827	835	TLADAGFIK	0.8305
A03	HLA-A*11:05	925	933	NQFNSAIGK	0.5165
A03	HLA-A*11:05	939	947	SSTASALGK	0.7797
A03	HLA-A*11:05	956	964	AQALNTLVK	0.5151
A03	HLA-A*11:05	975	983	SVLNDILSR	0.8499
A03	HLA-A*11:05	975	986	SVLNDILSRLDK	0.5938
A03	HLA-A*11:05	1019	1028	RASANLAATK	0.5185
A03	HLA-A*11:05	1020	1028	ASANLAATK	0.9119
A03	HLA-A*11:05	1064	1073	HVTYVPAQEK	0.6484
A03	HLA-A*11:05	1065	1073	VTYVPAQEK	0.9153
A03	HLA-A*11:05	1099	1107	GTHWFVTQR	0.7768
A03	HLA-A*11:05	1196	1205	SLIDLQELGK	0.5993
A03	HLA-A*11:07	35	44	GVYYPDKVFR	0.6555
A03	HLA-A*11:07	89	97	GVYFASTEK	0.9386
A03	HLA-A*11:07	142	150	GVYYHKNNK	0.7229
A03	HLA-A*11:07	292	300	ALDPLSETK	0.6795
A03	HLA-A*11:07	302	310	TLKSFTVEK	0.819
A03	HLA-A*11:07	311	319	GIYQTSNFR	0.5901
A03	HLA-A*11:07	348	356	ASVYAWNRK	0.6551
A03	HLA-A*11:07	349	357	SVYAWNRKR	0.576
A03	HLA-A*11:07	369	378	YNSASFSTFK	0.5561
A03	HLA-A*11:07	370	378	NSASFSTFK	0.8025
A03	HLA-A*11:07	375	386	STFKCYGVSPTK	0.6638
A03	HLA-A*11:07	408	417	RQIAPGQTGK	0.6249
A03	HLA-A*11:07	409	417	QIAPGQTGK	0.6748
A03	HLA-A*11:07	454	462	RLFRKSNLK	0.6772
A03	HLA-A*11:07	550	558	GVLTESNKK	0.7312
A03	HLA-A*11:07	686	695	SVASQSIIAY	0.5292
A03	HLA-A*11:07	723	733	TTEILPVSMTK	0.5964

A03	HLA-A*11:07	724	733	TEILPVSMTK	0.7926
A03	HLA-A*11:07	725	733	EILPVSMTK	0.7733
A03	HLA-A*11:07	787	795	QIYKTPPIK	0.7378
A03	HLA-A*11:07	825	835	KVTLADAGFIK	0.5709
A03	HLA-A*11:07	826	835	VTLADAGFIK	0.7264
A03	HLA-A*11:07	827	835	TLADAGFIK	0.8305
A03	HLA-A*11:07	925	933	NQFNSAIGK	0.5165
A03	HLA-A*11:07	939	947	SSTASALGK	0.7797
A03	HLA-A*11:07	956	964	AQALNTLVK	0.5151
A03	HLA-A*11:07	975	983	SVLNDILSR	0.8499
A03	HLA-A*11:07	975	986	SVLNDILSRLDK	0.5938
A03	HLA-A*11:07	1019	1028	RASANLAATK	0.5185
A03	HLA-A*11:07	1020	1028	ASANLAATK	0.9119
A03	HLA-A*11:07	1064	1073	HVTYVPAQEK	0.6484
A03	HLA-A*11:07	1065	1073	VTYVPAQEK	0.9153
A03	HLA-A*11:07	1099	1107	GTHWFVTQR	0.7768
A03	HLA-A*11:07	1196	1205	SLIDLQELGK	0.5993
A03	HLA-A*11:08	30	38	NSFTRGVYY	0.6147
A03	HLA-A*11:08	41	49	KVFRSSVLH	0.5716
A03	HLA-A*11:08	69	77	HVSGTNGTK	0.5285
A03	HLA-A*11:08	89	97	GVYFASTEK	0.9181
A03	HLA-A*11:08	142	150	GVYYHKNNK	0.7168
A03	HLA-A*11:08	257	266	GWTAGAAAYY	0.6585
A03	HLA-A*11:08	258	266	WTAGAAAYY	0.5874
A03	HLA-A*11:08	261	269	GAAAYYVGY	0.5405
A03	HLA-A*11:08	292	300	ALDPLSETK	0.7101
A03	HLA-A*11:08	302	310	TLKSFTVEK	0.8239
A03	HLA-A*11:08	311	319	GIYQTSNFR	0.5198
A03	HLA-A*11:08	348	356	ASVYAWNRK	0.6142
A03	HLA-A*11:08	349	357	SVYAWNRKR	0.5031
A03	HLA-A*11:08	361	369	CVADYSVLY	0.5983
A03	HLA-A*11:08	369	378	YNSASFSTFK	0.5604
A03	HLA-A*11:08	370	378	NSASFSTFK	0.8068
A03	HLA-A*11:08	375	386	STFKCYGVSPTK	0.5765
A03	HLA-A*11:08	408	417	RQIAPGQTGK	0.7216
A03	HLA-A*11:08	409	417	QIAPGQTGK	0.7553
A03	HLA-A*11:08	454	462	RLFRKSNLK	0.707
A03	HLA-A*11:08	550	558	GVLTESNKK	0.7019
A03	HLA-A*11:08	604	612	TSNQVAVLY	0.6574
A03	HLA-A*11:08	686	695	SVASQSIIAY	0.7162
A03	HLA-A*11:08	723	733	TTEILPVSMTK	0.519
A03	HLA-A*11:08	724	733	TEILPVSMTK	0.7064
A03	HLA-A*11:08	725	733	EILPVSMTK	0.7265
A03	HLA-A*11:08	787	795	QIYKTPPIK	0.7324
A03	HLA-A*11:08	805	814	ILPDPSKPSK	0.536
A03	HLA-A*11:08	825	835	KVTLADAGFIK	0.5029
A03	HLA-A*11:08	826	835	VTLADAGFIK	0.6667
A03	HLA-A*11:08	827	835	TLADAGFIK	0.8043
A03	HLA-A*11:08	925	933	NQFNSAIGK	0.5146
A03	HLA-A*11:08	939	947	SSTASALGK	0.7826

A03	HLA-A*11:08	956	964	AQALNTLVK	0.5229
A03	HLA-A*11:08	975	983	SVLNDILSR	0.7316
A03	HLA-A*11:08	1019	1028	RASANLAATK	0.5652
A03	HLA-A*11:08	1020	1028	ASANLAATK	0.8837
A03	HLA-A*11:08	1064	1073	HVTYVPAQEK	0.6371
A03	HLA-A*11:08	1065	1073	VTYVPAQEK	0.8948
A03	HLA-A*11:08	1099	1107	GTHWFVTQR	0.6403
A03	HLA-A*11:08	1196	1205	SLIDLQELGK	0.5719
A03	HLA-A*11:09	35	44	GVYYPDKVFR	0.6555
A03	HLA-A*11:09	89	97	GVYFASTEK	0.9386
A03	HLA-A*11:09	142	150	GVYYHKNNK	0.7229
A03	HLA-A*11:09	292	300	ALDPLSETK	0.6795
A03	HLA-A*11:09	302	310	TLKSFTVEK	0.819
A03	HLA-A*11:09	311	319	GIYQTSNFR	0.5901
A03	HLA-A*11:09	348	356	ASVYAWNRK	0.6551
A03	HLA-A*11:09	349	357	SVYAWNRKR	0.576
A03	HLA-A*11:09	369	378	YNSASFSTFK	0.5561
A03	HLA-A*11:09	370	378	NSASFSTFK	0.8025
A03	HLA-A*11:09	375	386	STFKCYGVSPTK	0.6638
A03	HLA-A*11:09	408	417	RQIAPGQTGK	0.6249
A03	HLA-A*11:09	409	417	QIAPGQTGK	0.6748
A03	HLA-A*11:09	454	462	RLFRKSNLK	0.6772
A03	HLA-A*11:09	550	558	GVLTESNKK	0.7312
A03	HLA-A*11:09	686	695	SVASQSIIAY	0.5292
A03	HLA-A*11:09	723	733	TTEILPVSMTK	0.5964
A03	HLA-A*11:09	724	733	TEILPVSMTK	0.7926
A03	HLA-A*11:09	725	733	EILPVSMTK	0.7733
A03	HLA-A*11:09	787	795	QIYKTPPIK	0.7378
A03	HLA-A*11:09	825	835	KVTLADAGFIK	0.5709
A03	HLA-A*11:09	826	835	VTLADAGFIK	0.7264
A03	HLA-A*11:09	827	835	TLADAGFIK	0.8305
A03	HLA-A*11:09	925	933	NQFNSAIGK	0.5165
A03	HLA-A*11:09	939	947	SSTASALGK	0.7797
A03	HLA-A*11:09	956	964	AQALNTLVK	0.5151
A03	HLA-A*11:09	975	983	SVLNDILSR	0.8499
A03	HLA-A*11:09	975	986	SVLNDILSRLDK	0.5938
A03	HLA-A*11:09	1019	1028	RASANLAATK	0.5185
A03	HLA-A*11:09	1020	1028	ASANLAATK	0.9119
A03	HLA-A*11:09	1064	1073	HVTYVPAQEK	0.6484
A03	HLA-A*11:09	1065	1073	VTYVPAQEK	0.9153
A03	HLA-A*11:09	1099	1107	GTHWFVTQR	0.7768
A03	HLA-A*11:09	1196	1205	SLIDLQELGK	0.5993
A03	HLA-A*11:10	30	38	NSFTRGVYY	0.5648
A03	HLA-A*11:10	88	97	DGVYFASTEK	0.5092
A03	HLA-A*11:10	89	97	GVYFASTEK	0.6152
A03	HLA-A*11:10	258	266	WTAGAAAYY	0.5276
A03	HLA-A*11:10	361	369	CVADYSVLY	0.5044
A03	HLA-A*11:10	370	378	NSASFSTFK	0.7786
A03	HLA-A*11:10	724	733	TEILPVSMTK	0.5103
A03	HLA-A*11:10	725	733	EILPVSMTK	0.8498

A03	HLA-A*11:10	817	825	FIEDLLFNK	0.5044
A03	HLA-A*11:10	827	835	TLADAGFIK	0.6366
A03	HLA-A*11:10	975	983	SVLNDILSR	0.6653
A03	HLA-A*11:10	1065	1073	VTYVPAQEK	0.5627
A03	HLA-A*11:10	1173	1181	NASVVNIQK	0.5907
A03	HLA-A*11:12	35	44	GVYYPDKVFR	0.6555
A03	HLA-A*11:12	89	97	GVYFASTEK	0.9386
A03	HLA-A*11:12	142	150	GVYYHKNNK	0.7229
A03	HLA-A*11:12	292	300	ALDPLSETK	0.6795
A03	HLA-A*11:12	302	310	TLKSFTVEK	0.819
A03	HLA-A*11:12	311	319	GIYQTSNFR	0.5901
A03	HLA-A*11:12	348	356	ASVYAWNRK	0.6551
A03	HLA-A*11:12	349	357	SVYAWNRKR	0.576
A03	HLA-A*11:12	369	378	YNSASFSTFK	0.5561
A03	HLA-A*11:12	370	378	NSASFSTFK	0.8025
A03	HLA-A*11:12	375	386	STFKCYGVSPTK	0.6638
A03	HLA-A*11:12	408	417	RQIAPGQTGK	0.6249
A03	HLA-A*11:12	409	417	QIAPGQTGK	0.6748
A03	HLA-A*11:12	454	462	RLFRKSNLK	0.6772
A03	HLA-A*11:12	550	558	GVLTESNKK	0.7312
A03	HLA-A*11:12	686	695	SVASQSIIAY	0.5292
A03	HLA-A*11:12	723	733	TTEILPVSMTK	0.5964
A03	HLA-A*11:12	724	733	TEILPVSMTK	0.7926
A03	HLA-A*11:12	725	733	EILPVSMTK	0.7733
A03	HLA-A*11:12	787	795	QIYKTPPIK	0.7378
A03	HLA-A*11:12	825	835	KVTLADAGFIK	0.5709
A03	HLA-A*11:12	826	835	VTLADAGFIK	0.7264
A03	HLA-A*11:12	827	835	TLADAGFIK	0.8305
A03	HLA-A*11:12	925	933	NQFNSAIGK	0.5165
A03	HLA-A*11:12	939	947	SSTASALGK	0.7797
A03	HLA-A*11:12	956	964	AQALNTLVK	0.5151
A03	HLA-A*11:12	975	983	SVLNDILSR	0.8499
A03	HLA-A*11:12	975	986	SVLNDILSRLDK	0.5938
A03	HLA-A*11:12	1019	1028	RASANLAATK	0.5185
A03	HLA-A*11:12	1020	1028	ASANLAATK	0.9119
A03	HLA-A*11:12	1064	1073	HVTYVPAQEK	0.6484
A03	HLA-A*11:12	1065	1073	VTYVPAQEK	0.9153
A03	HLA-A*11:12	1099	1107	GTHWFVTQR	0.7768
A03	HLA-A*11:12	1196	1205	SLIDLQELGK	0.5993
A03	HLA-A*11:13	35	44	GVYYPDKVFR	0.6555
A03	HLA-A*11:13	89	97	GVYFASTEK	0.9386
A03	HLA-A*11:13	142	150	GVYYHKNNK	0.7229
A03	HLA-A*11:13	292	300	ALDPLSETK	0.6795
A03	HLA-A*11:13	302	310	TLKSFTVEK	0.819
A03	HLA-A*11:13	311	319	GIYQTSNFR	0.5901
A03	HLA-A*11:13	348	356	ASVYAWNRK	0.6551
A03	HLA-A*11:13	349	357	SVYAWNRKR	0.576
A03	HLA-A*11:13	369	378	YNSASFSTFK	0.5561
A03	HLA-A*11:13	370	378	NSASFSTFK	0.8025
A03	HLA-A*11:13	375	386	STFKCYGVSPTK	0.6638

A03	HLA-A*11:13	408	417	RQIAPGQTGK	0.6249
A03	HLA-A*11:13	409	417	QIAPGQTGK	0.6748
A03	HLA-A*11:13	454	462	RLFRKSNLK	0.6772
A03	HLA-A*11:13	550	558	GVLTESNKK	0.7312
A03	HLA-A*11:13	686	695	SVASQSIIAY	0.5292
A03	HLA-A*11:13	723	733	TTEILPVSMTK	0.5964
A03	HLA-A*11:13	724	733	TEILPVSMTK	0.7926
A03	HLA-A*11:13	725	733	EILPVSMTK	0.7733
A03	HLA-A*11:13	787	795	QIYKTPPIK	0.7378
A03	HLA-A*11:13	825	835	KVTLADAGFIK	0.5709
A03	HLA-A*11:13	826	835	VTLADAGFIK	0.7264
A03	HLA-A*11:13	827	835	TLADAGFIK	0.8305
A03	HLA-A*11:13	925	933	NQFNSAIGK	0.5165
A03	HLA-A*11:13	939	947	SSTASALGK	0.7797
A03	HLA-A*11:13	956	964	AQALNTLVK	0.5151
A03	HLA-A*11:13	975	983	SVLNDILSR	0.8499
A03	HLA-A*11:13	975	986	SVLNDILSRLDK	0.5938
A03	HLA-A*11:13	1019	1028	RASANLAATK	0.5185
A03	HLA-A*11:13	1020	1028	ASANLAATK	0.9119
A03	HLA-A*11:13	1064	1073	HVTYVPAQEK	0.6484
A03	HLA-A*11:13	1065	1073	VTYVPAQEK	0.9153
A03	HLA-A*11:13	1099	1107	GTHWFVTQR	0.7768
A03	HLA-A*11:13	1196	1205	SLIDLQELGK	0.5993
A03	HLA-A*11:14	41	49	KVFRSSVLH	0.5801
A03	HLA-A*11:14	89	97	GVYFASTEK	0.9113
A03	HLA-A*11:14	142	150	GVYYHKNNK	0.7242
A03	HLA-A*11:14	292	300	ALDPLSETK	0.552
A03	HLA-A*11:14	302	310	TLKSFTVEK	0.7933
A03	HLA-A*11:14	311	319	GIYQTSNFR	0.5287
A03	HLA-A*11:14	348	356	ASVYAWNRK	0.5921
A03	HLA-A*11:14	349	357	SVYAWNRKR	0.524
A03	HLA-A*11:14	370	378	NSASFSTFK	0.7046
A03	HLA-A*11:14	375	386	STFKCYGVSPTK	0.5354
A03	HLA-A*11:14	408	417	RQIAPGQTGK	0.6772
A03	HLA-A*11:14	409	417	QIAPGQTGK	0.6664
A03	HLA-A*11:14	454	462	RLFRKSNLK	0.7323
A03	HLA-A*11:14	550	558	GVLTESNKK	0.6399
A03	HLA-A*11:14	686	695	SVASQSIIAY	0.5437
A03	HLA-A*11:14	724	733	TEILPVSMTK	0.6944
A03	HLA-A*11:14	725	733	EILPVSMTK	0.6759
A03	HLA-A*11:14	787	795	QIYKTPPIK	0.728
A03	HLA-A*11:14	826	835	VTLADAGFIK	0.595
A03	HLA-A*11:14	827	835	TLADAGFIK	0.7304
A03	HLA-A*11:14	924	933	ANQFNSAIGK	0.5078
A03	HLA-A*11:14	925	933	NQFNSAIGK	0.519
A03	HLA-A*11:14	939	947	SSTASALGK	0.7137
A03	HLA-A*11:14	956	964	AQALNTLVK	0.5035
A03	HLA-A*11:14	975	983	SVLNDILSR	0.743
A03	HLA-A*11:14	1019	1028	RASANLAATK	0.5006
A03	HLA-A*11:14	1020	1028	ASANLAATK	0.857

A03	HLA-A*11:14	1064	1073	HVTYVPAQEK	0.5851
A03	HLA-A*11:14	1065	1073	VTYVPAQEK	0.8766
A03	HLA-A*11:14	1099	1107	GTHWFVTQR	0.6316
A03	HLA-A*11:14	1196	1205	SLIDLQELGK	0.536
A03	HLA-A*11:15	35	44	GVYYPDKVFR	0.6555
A03	HLA-A*11:15	89	97	GVYFASTEK	0.9386
A03	HLA-A*11:15	142	150	GVYYHKNNK	0.7229
A03	HLA-A*11:15	292	300	ALDPLSETK	0.6795
A03	HLA-A*11:15	302	310	TLKSFTVEK	0.819
A03	HLA-A*11:15	311	319	GIYQTSNFR	0.5901
A03	HLA-A*11:15	348	356	ASVYAWNRK	0.6551
A03	HLA-A*11:15	349	357	SVYAWNRKR	0.576
A03	HLA-A*11:15	369	378	YNSASFSTFK	0.5561
A03	HLA-A*11:15	370	378	NSASFSTFK	0.8025
A03	HLA-A*11:15	375	386	STFKCYGVSPTK	0.6638
A03	HLA-A*11:15	408	417	RQIAPGQTGK	0.6249
A03	HLA-A*11:15	409	417	QIAPGQTGK	0.6748
A03	HLA-A*11:15	454	462	RLFRKSNLK	0.6772
A03	HLA-A*11:15	550	558	GVLTESNKK	0.7312
A03	HLA-A*11:15	686	695	SVASQSIIAY	0.5292
A03	HLA-A*11:15	723	733	TTEILPVSMTK	0.5964
A03	HLA-A*11:15	724	733	TEILPVSMTK	0.7926
A03	HLA-A*11:15	725	733	EILPVSMTK	0.7733
A03	HLA-A*11:15	787	795	QIYKTPPIK	0.7378
A03	HLA-A*11:15	825	835	KVTLADAGFIK	0.5709
A03	HLA-A*11:15	826	835	VTLADAGFIK	0.7264
A03	HLA-A*11:15	827	835	TLADAGFIK	0.8305
A03	HLA-A*11:15	925	933	NQFNSAIGK	0.5165
A03	HLA-A*11:15	939	947	SSTASALGK	0.7797
A03	HLA-A*11:15	956	964	AQALNTLVK	0.5151
A03	HLA-A*11:15	975	983	SVLNDILSR	0.8499
A03	HLA-A*11:15	975	986	SVLNDILSRLDK	0.5938
A03	HLA-A*11:15	1019	1028	RASANLAATK	0.5185
A03	HLA-A*11:15	1020	1028	ASANLAATK	0.9119
A03	HLA-A*11:15	1064	1073	HVTYVPAQEK	0.6484
A03	HLA-A*11:15	1065	1073	VTYVPAQEK	0.9153
A03	HLA-A*11:15	1099	1107	GTHWFVTQR	0.7768
A03	HLA-A*11:15	1196	1205	SLIDLQELGK	0.5993
A03	HLA-A*11:16	35	44	GVYYPDKVFR	0.6555
A03	HLA-A*11:16	89	97	GVYFASTEK	0.9386
A03	HLA-A*11:16	142	150	GVYYHKNNK	0.7229
A03	HLA-A*11:16	292	300	ALDPLSETK	0.6795
A03	HLA-A*11:16	302	310	TLKSFTVEK	0.819
A03	HLA-A*11:16	311	319	GIYQTSNFR	0.5901
A03	HLA-A*11:16	348	356	ASVYAWNRK	0.6551
A03	HLA-A*11:16	349	357	SVYAWNRKR	0.576
A03	HLA-A*11:16	369	378	YNSASFSTFK	0.5561
A03	HLA-A*11:16	370	378	NSASFSTFK	0.8025
A03	HLA-A*11:16	375	386	STFKCYGVSPTK	0.6638
A03	HLA-A*11:16	408	417	RQIAPGQTGK	0.6249

A03	HLA-A*11:16	409	417	QIAPGQTGK	0.6748
A03	HLA-A*11:16	454	462	RLFRKSNLK	0.6772
A03	HLA-A*11:16	550	558	GVLTESNKK	0.7312
A03	HLA-A*11:16	686	695	SVASQSIIAY	0.5292
A03	HLA-A*11:16	723	733	TTEILPVSMTK	0.5964
A03	HLA-A*11:16	724	733	TEILPVSMTK	0.7926
A03	HLA-A*11:16	725	733	EILPVSMTK	0.7733
A03	HLA-A*11:16	787	795	QIYKTPPIK	0.7378
A03	HLA-A*11:16	825	835	KVTLADAGFIK	0.5709
A03	HLA-A*11:16	826	835	VTLADAGFIK	0.7264
A03	HLA-A*11:16	827	835	TLADAGFIK	0.8305
A03	HLA-A*11:16	925	933	NQFNSAIGK	0.5165
A03	HLA-A*11:16	939	947	SSTASALGK	0.7797
A03	HLA-A*11:16	956	964	AQALNTLVK	0.5151
A03	HLA-A*11:16	975	983	SVLNDILSR	0.8499
A03	HLA-A*11:16	975	986	SVLNDILSRLDK	0.5938
A03	HLA-A*11:16	1019	1028	RASANLAATK	0.5185
A03	HLA-A*11:16	1020	1028	ASANLAATK	0.9119
A03	HLA-A*11:16	1064	1073	HVTYVPAQEK	0.6484
A03	HLA-A*11:16	1065	1073	VTYVPAQEK	0.9153
A03	HLA-A*11:16	1099	1107	GTHWFVTQR	0.7768
A03	HLA-A*11:16	1196	1205	SLIDLQELGK	0.5993
A03	HLA-A*11:20	35	44	GVYYPDKVFR	0.5729
A03	HLA-A*11:20	41	49	KVFRSSVLH	0.6359
A03	HLA-A*11:20	69	77	HVSGTNGTK	0.5574
A03	HLA-A*11:20	89	97	GVYFASTEK	0.9479
A03	HLA-A*11:20	142	150	GVYYHKNNK	0.8636
A03	HLA-A*11:20	257	266	GWTAGAAAYY	0.5027
A03	HLA-A*11:20	292	300	ALDPLSETK	0.6109
A03	HLA-A*11:20	302	310	TLKSFTVEK	0.8758
A03	HLA-A*11:20	311	319	GIYQTSNFR	0.6106
A03	HLA-A*11:20	348	356	ASVYAWNRK	0.6559
A03	HLA-A*11:20	349	357	SVYAWNRKR	0.6192
A03	HLA-A*11:20	369	378	YNSASFSTFK	0.5161
A03	HLA-A*11:20	370	378	NSASFSTFK	0.7603
A03	HLA-A*11:20	375	386	STFKCYGVSPTK	0.6584
A03	HLA-A*11:20	408	417	RQIAPGQTGK	0.8115
A03	HLA-A*11:20	409	417	QIAPGQTGK	0.7924
A03	HLA-A*11:20	454	462	RLFRKSNLK	0.839
A03	HLA-A*11:20	529	537	KSTNLVKNK	0.6205
A03	HLA-A*11:20	550	558	GVLTESNKK	0.7419
A03	HLA-A*11:20	686	695	SVASQSIIAY	0.5979
A03	HLA-A*11:20	723	733	TTEILPVSMTK	0.5555
A03	HLA-A*11:20	724	733	TEILPVSMTK	0.7868
A03	HLA-A*11:20	725	733	EILPVSMTK	0.7674
A03	HLA-A*11:20	786	795	KQIYKTPPIK	0.5919
A03	HLA-A*11:20	787	795	QIYKTPPIK	0.8358
A03	HLA-A*11:20	825	835	KVTLADAGFIK	0.5148
A03	HLA-A*11:20	826	835	VTLADAGFIK	0.6588
A03	HLA-A*11:20	827	835	TLADAGFIK	0.7979

A03	HLA-A*11:20	924	933	ANQFNSAIGK	0.5982
A03	HLA-A*11:20	925	933	NQFNSAIGK	0.5795
A03	HLA-A*11:20	939	947	SSTASALGK	0.7993
A03	HLA-A*11:20	956	964	AQALNTLVK	0.5795
A03	HLA-A*11:20	975	983	SVLNDILSR	0.7656
A03	HLA-A*11:20	975	986	SVLNDILSRLDK	0.5176
A03	HLA-A*11:20	1019	1028	RASANLAATK	0.6171
A03	HLA-A*11:20	1020	1028	ASANLAATK	0.9033
A03	HLA-A*11:20	1064	1073	HVTYVPAQEK	0.7064
A03	HLA-A*11:20	1065	1073	VTYVPAQEK	0.9204
A03	HLA-A*11:20	1099	1107	GTHWFVTQR	0.7038
A03	HLA-A*11:20	1196	1205	SLIDLQELGK	0.6467
A03	HLA-A*11:23	35	44	GVYYPDKVFR	0.6555
A03	HLA-A*11:23	89	97	GVYFASTEK	0.9386
A03	HLA-A*11:23	142	150	GVYYHKNNK	0.7229
A03	HLA-A*11:23	292	300	ALDPLSETK	0.6795
A03	HLA-A*11:23	302	310	TLKSFTVEK	0.819
A03	HLA-A*11:23	311	319	GIYQTSNFR	0.5901
A03	HLA-A*11:23	348	356	ASVYAWNRK	0.6551
A03	HLA-A*11:23	349	357	SVYAWNRKR	0.576
A03	HLA-A*11:23	369	378	YNSASFSTFK	0.5561
A03	HLA-A*11:23	370	378	NSASFSTFK	0.8025
A03	HLA-A*11:23	375	386	STFKCYGVSPTK	0.6638
A03	HLA-A*11:23	408	417	RQIAPGQTGK	0.6249
A03	HLA-A*11:23	409	417	QIAPGQTGK	0.6748
A03	HLA-A*11:23	454	462	RLFRKSNLK	0.6772
A03	HLA-A*11:23	550	558	GVLTESNKK	0.7312
A03	HLA-A*11:23	686	695	SVASQSIIAY	0.5292
A03	HLA-A*11:23	723	733	TTEILPVSMTK	0.5964
A03	HLA-A*11:23	724	733	TEILPVSMTK	0.7926
A03	HLA-A*11:23	725	733	EILPVSMTK	0.7733
A03	HLA-A*11:23	787	795	QIYKTPPIK	0.7378
A03	HLA-A*11:23	825	835	KVTLADAGFIK	0.5709
A03	HLA-A*11:23	826	835	VTLADAGFIK	0.7264
A03	HLA-A*11:23	827	835	TLADAGFIK	0.8305
A03	HLA-A*11:23	925	933	NQFNSAIGK	0.5165
A03	HLA-A*11:23	939	947	SSTASALGK	0.7797
A03	HLA-A*11:23	956	964	AQALNTLVK	0.5151
A03	HLA-A*11:23	975	983	SVLNDILSR	0.8499
A03	HLA-A*11:23	975	986	SVLNDILSRLDK	0.5938
A03	HLA-A*11:23	1019	1028	RASANLAATK	0.5185
A03	HLA-A*11:23	1020	1028	ASANLAATK	0.9119
A03	HLA-A*11:23	1064	1073	HVTYVPAQEK	0.6484
A03	HLA-A*11:23	1065	1073	VTYVPAQEK	0.9153
A03	HLA-A*11:23	1099	1107	GTHWFVTQR	0.7768
A03	HLA-A*11:23	1196	1205	SLIDLQELGK	0.5993
A03	HLA-A*31:01	34	44	RGVYYPDKVFR	0.5574
A03	HLA-A*31:01	35	44	GVYYPDKVFR	0.7294
A03	HLA-A*31:01	36	44	VYYPDKVFR	0.8085
A03	HLA-A*31:01	150	158	KSWMESEFR	0.5583

A03	HLA-A*31:01	182	190	KQGNFKNLR	0.6717
A03	HLA-A*31:01	310	319	KGIYQTSNFR	0.5075
A03	HLA-A*31:01	311	319	GIYQTSNFR	0.6612
A03	HLA-A*31:01	319	328	RVQPTESIVR	0.5738
A03	HLA-A*31:01	346	355	RFASVYAWNR	0.7025
A03	HLA-A*31:01	349	357	SVYAWNRKR	0.8646
A03	HLA-A*31:01	395	403	VYADSFVIR	0.575
A03	HLA-A*31:01	446	454	GGNYNYLYR	0.5086
A03	HLA-A*31:01	454	462	RLFRKSNLK	0.5237
A03	HLA-A*31:01	458	466	KSNLKPFER	0.8687
A03	HLA-A*31:01	558	567	KFLPFQQFGR	0.6235
A03	HLA-A*31:01	677	685	QTNSPRRAR	0.6557
A03	HLA-A*31:01	975	983	SVLNDILSR	0.6352
A03	HLA-A*31:01	1099	1107	GTHWFVTQR	0.837
A03	HLA-A*31:03	34	44	RGVYYPDKVFR	0.6638
A03	HLA-A*31:03	35	44	GVYYPDKVFR	0.8323
A03	HLA-A*31:03	36	44	VYYPDKVFR	0.882
A03	HLA-A*31:03	142	150	GVYYHKNNK	0.5143
A03	HLA-A*31:03	150	158	KSWMESEFR	0.7064
A03	HLA-A*31:03	182	190	KQGNFKNLR	0.768
A03	HLA-A*31:03	193	202	VFKNIDGYFK	0.6092
A03	HLA-A*31:03	237	246	RFQTLLALHR	0.5604
A03	HLA-A*31:03	264	273	AYYVGYLQPR	0.5113
A03	HLA-A*31:03	302	310	TLKSFTVEK	0.6023
A03	HLA-A*31:03	310	319	KGIYQTSNFR	0.6
A03	HLA-A*31:03	311	319	GIYQTSNFR	0.7783
A03	HLA-A*31:03	319	328	RVQPTESIVR	0.6913
A03	HLA-A*31:03	346	355	RFASVYAWNR	0.73
A03	HLA-A*31:03	348	357	ASVYAWNRKR	0.5374
A03	HLA-A*31:03	349	357	SVYAWNRKR	0.8673
A03	HLA-A*31:03	394	403	NVYADSFVIR	0.5006
A03	HLA-A*31:03	395	403	VYADSFVIR	0.708
A03	HLA-A*31:03	446	454	GGNYNYLYR	0.7092
A03	HLA-A*31:03	449	457	YNYLYRLFR	0.5488
A03	HLA-A*31:03	454	462	RLFRKSNLK	0.6239
A03	HLA-A*31:03	457	466	RKSNLKPFER	0.5092
A03	HLA-A*31:03	458	466	KSNLKPFER	0.9139
A03	HLA-A*31:03	558	567	KFLPFQQFGR	0.7268
A03	HLA-A*31:03	637	646	STGSNVFQTR	0.6297
A03	HLA-A*31:03	638	646	TGSNVFQTR	0.5408
A03	HLA-A*31:03	677	685	QTNSPRRAR	0.6697
A03	HLA-A*31:03	757	765	GSFCTQLNR	0.5163
A03	HLA-A*31:03	975	983	SVLNDILSR	0.8099
A03	HLA-A*31:03	991	1000	VQIDRLITGR	0.5083
A03	HLA-A*31:03	1099	1107	GTHWFVTQR	0.8669
A03	HLA-A*31:04	34	44	RGVYYPDKVFR	0.6638
A03	HLA-A*31:04	35	44	GVYYPDKVFR	0.8323
A03	HLA-A*31:04	36	44	VYYPDKVFR	0.882
A03	HLA-A*31:04	142	150	GVYYHKNNK	0.5143
A03	HLA-A*31:04	150	158	KSWMESEFR	0.7064

A03	HLA-A*31:04	182	190	KQGNFKNLR	0.768
A03	HLA-A*31:04	193	202	VFKNIDGYFK	0.6092
A03	HLA-A*31:04	237	246	RFQTLLALHR	0.5604
A03	HLA-A*31:04	264	273	AYYVGYLQPR	0.5113
A03	HLA-A*31:04	302	310	TLKSFTVEK	0.6023
A03	HLA-A*31:04	310	319	KGIYQTSNFR	0.6
A03	HLA-A*31:04	311	319	GIYQTSNFR	0.7783
A03	HLA-A*31:04	319	328	RVQPTESIVR	0.6913
A03	HLA-A*31:04	346	355	RFASVYAWNR	0.73
A03	HLA-A*31:04	348	357	ASVYAWNRKR	0.5374
A03	HLA-A*31:04	349	357	SVYAWNRKR	0.8673
A03	HLA-A*31:04	394	403	NVYADSFVIR	0.5006
A03	HLA-A*31:04	395	403	VYADSFVIR	0.708
A03	HLA-A*31:04	446	454	GGNYNYLYR	0.7092
A03	HLA-A*31:04	449	457	YNYLYRLFR	0.5488
A03	HLA-A*31:04	454	462	RLFRKSNLK	0.6239
A03	HLA-A*31:04	457	466	RKSNLKPFER	0.5092
A03	HLA-A*31:04	458	466	KSNLKPFER	0.9139
A03	HLA-A*31:04	558	567	KFLPFQQFGR	0.7268
A03	HLA-A*31:04	637	646	STGSNVFQTR	0.6297
A03	HLA-A*31:04	638	646	TGSNVFQTR	0.5408
A03	HLA-A*31:04	677	685	QTNSPRRAR	0.6697
A03	HLA-A*31:04	757	765	GSFCTQLNR	0.5163
A03	HLA-A*31:04	975	983	SVLNDILSR	0.8099
A03	HLA-A*31:04	991	1000	VQIDRLITGR	0.5083
A03	HLA-A*31:04	1099	1107	GTHWFVTQR	0.8669
A03	HLA-A*31:05	35	44	GVYYPDKVFR	0.5445
A03	HLA-A*31:05	36	44	VYYPDKVFR	0.7165
A03	HLA-A*31:05	346	355	RFASVYAWNR	0.6108
A03	HLA-A*31:05	349	357	SVYAWNRKR	0.6829
A03	HLA-A*31:05	395	403	VYADSFVIR	0.5499
A03	HLA-A*31:05	458	466	KSNLKPFER	0.6747
A03	HLA-A*31:05	1099	1107	GTHWFVTQR	0.6122
A03	HLA-A*31:06	34	44	RGVYYPDKVFR	0.5993
A03	HLA-A*31:06	35	44	GVYYPDKVFR	0.8058
A03	HLA-A*31:06	36	44	VYYPDKVFR	0.8626
A03	HLA-A*31:06	150	158	KSWMESEFR	0.6719
A03	HLA-A*31:06	182	190	KQGNFKNLR	0.7159
A03	HLA-A*31:06	193	202	VFKNIDGYFK	0.5689
A03	HLA-A*31:06	237	246	RFQTLLALHR	0.5435
A03	HLA-A*31:06	302	310	TLKSFTVEK	0.5191
A03	HLA-A*31:06	310	319	KGIYQTSNFR	0.5598
A03	HLA-A*31:06	311	319	GIYQTSNFR	0.7562
A03	HLA-A*31:06	319	328	RVQPTESIVR	0.6572
A03	HLA-A*31:06	346	355	RFASVYAWNR	0.7268
A03	HLA-A*31:06	349	357	SVYAWNRKR	0.8534
A03	HLA-A*31:06	395	403	VYADSFVIR	0.6684
A03	HLA-A*31:06	446	454	GGNYNYLYR	0.647
A03	HLA-A*31:06	449	457	YNYLYRLFR	0.5126
A03	HLA-A*31:06	454	462	RLFRKSNLK	0.5816

A03	HLA-A*31:06	458	466	KSNLKPFER	0.8983
A03	HLA-A*31:06	558	567	KFLPFQQFGR	0.6977
A03	HLA-A*31:06	637	646	STGSNVFQTR	0.5673
A03	HLA-A*31:06	677	685	QTNSPRRAR	0.5945
A03	HLA-A*31:06	975	983	SVLNDILSR	0.7759
A03	HLA-A*31:06	1099	1107	GTHWFVTQR	0.856
A03	HLA-A*31:09	34	44	RGVYYPDKVFR	0.5141
A03	HLA-A*31:09	35	44	GVYYPDKVFR	0.663
A03	HLA-A*31:09	36	44	VYYPDKVFR	0.7682
A03	HLA-A*31:09	150	158	KSWMESEFR	0.517
A03	HLA-A*31:09	182	190	KQGNFKNLR	0.6571
A03	HLA-A*31:09	311	319	GIYQTSNFR	0.5434
A03	HLA-A*31:09	346	355	RFASVYAWNR	0.6162
A03	HLA-A*31:09	349	357	SVYAWNRKR	0.8004
A03	HLA-A*31:09	395	403	VYADSFVIR	0.5259
A03	HLA-A*31:09	458	466	KSNLKPFER	0.8223
A03	HLA-A*31:09	558	567	KFLPFQQFGR	0.5629
A03	HLA-A*31:09	677	685	QTNSPRRAR	0.5307
A03	HLA-A*31:09	975	983	SVLNDILSR	0.5479
A03	HLA-A*31:09	1099	1107	GTHWFVTQR	0.7562
A03	HLA-A*31:11	34	44	RGVYYPDKVFR	0.5574
A03	HLA-A*31:11	35	44	GVYYPDKVFR	0.7294
A03	HLA-A*31:11	36	44	VYYPDKVFR	0.8085
A03	HLA-A*31:11	150	158	KSWMESEFR	0.5583
A03	HLA-A*31:11	182	190	KQGNFKNLR	0.6717
A03	HLA-A*31:11	310	319	KGIYQTSNFR	0.5075
A03	HLA-A*31:11	311	319	GIYQTSNFR	0.6612
A03	HLA-A*31:11	319	328	RVQPTESIVR	0.5738
A03	HLA-A*31:11	346	355	RFASVYAWNR	0.7025
A03	HLA-A*31:11	349	357	SVYAWNRKR	0.8646
A03	HLA-A*31:11	395	403	VYADSFVIR	0.575
A03	HLA-A*31:11	446	454	GGNYNYLYR	0.5086
A03	HLA-A*31:11	454	462	RLFRKSNLK	0.5237
A03	HLA-A*31:11	458	466	KSNLKPFER	0.8687
A03	HLA-A*31:11	558	567	KFLPFQQFGR	0.6235
A03	HLA-A*31:11	677	685	QTNSPRRAR	0.6557
A03	HLA-A*31:11	975	983	SVLNDILSR	0.6352
A03	HLA-A*31:11	1099	1107	GTHWFVTQR	0.837
A03	HLA-A*33:01	36	44	VYYPDKVFR	0.6674
A03	HLA-A*33:01	265	273	YYVGYLQPR	0.6111
A03	HLA-A*33:01	349	357	SVYAWNRKR	0.5816
A03	HLA-A*33:01	394	403	NVYADSFVIR	0.575
A03	HLA-A*33:01	395	403	VYADSFVIR	0.5728
A03	HLA-A*33:01	448	457	NYNYLYRLFR	0.5281
A03	HLA-A*33:04	36	44	VYYPDKVFR	0.6674
A03	HLA-A*33:04	265	273	YYVGYLQPR	0.6111
A03	HLA-A*33:04	349	357	SVYAWNRKR	0.5816
A03	HLA-A*33:04	394	403	NVYADSFVIR	0.575
A03	HLA-A*33:04	395	403	VYADSFVIR	0.5728
A03	HLA-A*33:04	448	457	NYNYLYRLFR	0.5281

A03	HLA-A*33:05	36	44	VYYPDKVFR	0.6674
A03	HLA-A*33:05	265	273	YYVGYLQPR	0.6111
A03	HLA-A*33:05	349	357	SVYAWNRKR	0.5816
A03	HLA-A*33:05	394	403	NVYADSFVIR	0.575
A03	HLA-A*33:05	395	403	VYADSFVIR	0.5728
A03	HLA-A*33:05	448	457	NYNYLYRLFR	0.5281
A03	HLA-A*33:06	36	44	VYYPDKVFR	0.6561
A03	HLA-A*33:06	265	273	YYVGYLQPR	0.5103
A03	HLA-A*33:06	349	357	SVYAWNRKR	0.7608
A03	HLA-A*33:06	394	403	NVYADSFVIR	0.5705
A03	HLA-A*33:06	395	403	VYADSFVIR	0.5307
A03	HLA-A*33:06	677	685	QTNSPRRAR	0.5806
A03	HLA-A*33:06	975	983	SVLNDILSR	0.5342
A03	HLA-A*33:06	1099	1107	GTHWFVTQR	0.5592
A03	HLA-A*33:07	36	44	VYYPDKVFR	0.6674
A03	HLA-A*33:07	265	273	YYVGYLQPR	0.6111
A03	HLA-A*33:07	349	357	SVYAWNRKR	0.5816
A03	HLA-A*33:07	394	403	NVYADSFVIR	0.575
A03	HLA-A*33:07	395	403	VYADSFVIR	0.5728
A03	HLA-A*33:07	448	457	NYNYLYRLFR	0.5281
A03	HLA-A*34:02	30	38	NSFTRGVYY	0.7033
A03	HLA-A*34:02	35	44	GVYYPDKVFR	0.5826
A03	HLA-A*34:02	69	77	HVSGTNGTK	0.6254
A03	HLA-A*34:02	88	97	DGVYFASTEK	0.5696
A03	HLA-A*34:02	89	97	GVYFASTEK	0.81
A03	HLA-A*34:02	142	150	GVYYHKNNK	0.7107
A03	HLA-A*34:02	192	200	FVFKNIDGY	0.5463
A03	HLA-A*34:02	198	206	DGYFKIYSK	0.5064
A03	HLA-A*34:02	258	266	WTAGAAAYY	0.585
A03	HLA-A*34:02	302	310	TLKSFTVEK	0.7513
A03	HLA-A*34:02	311	319	GIYQTSNFR	0.5989
A03	HLA-A*34:02	349	357	SVYAWNRKR	0.7569
A03	HLA-A*34:02	370	378	NSASFSTFK	0.7929
A03	HLA-A*34:02	394	403	NVYADSFVIR	0.553
A03	HLA-A*34:02	409	417	QIAPGQTGK	0.7709
A03	HLA-A*34:02	724	733	TEILPVSMTK	0.6467
A03	HLA-A*34:02	725	733	EILPVSMTK	0.8798
A03	HLA-A*34:02	782	790	FAQVKQIYK	0.569
A03	HLA-A*34:02	787	795	QIYKTPPIK	0.744
A03	HLA-A*34:02	827	835	TLADAGFIK	0.6884
A03	HLA-A*34:02	975	983	SVLNDILSR	0.7688
A03	HLA-A*34:02	1020	1028	ASANLAATK	0.5816
A03	HLA-A*34:02	1064	1073	HVTYVPAQEK	0.7046
A03	HLA-A*34:02	1065	1073	VTYVPAQEK	0.8154
A03	HLA-A*34:02	1099	1107	GTHWFVTQR	0.5926
A03	HLA-A*34:02	1173	1181	NASVVNIQK	0.5921
A03	HLA-A*34:03	30	38	NSFTRGVYY	0.7033
A03	HLA-A*34:03	35	44	GVYYPDKVFR	0.5826
A03	HLA-A*34:03	69	77	HVSGTNGTK	0.6254
A03	HLA-A*34:03	88	97	DGVYFASTEK	0.5696

A03	HLA-A*34:03	89	97	GVYFASTEK	0.81
A03	HLA-A*34:03	142	150	GVYYHKNNK	0.7107
A03	HLA-A*34:03	192	200	FVFKNIDGY	0.5463
A03	HLA-A*34:03	198	206	DGYFKIYSK	0.5064
A03	HLA-A*34:03	258	266	WTAGAAAYY	0.585
A03	HLA-A*34:03	302	310	TLKSFTVEK	0.7513
A03	HLA-A*34:03	311	319	GIYQTSNFR	0.5989
A03	HLA-A*34:03	349	357	SVYAWNRKR	0.7569
A03	HLA-A*34:03	370	378	NSASFSTFK	0.7929
A03	HLA-A*34:03	394	403	NVYADSFVIR	0.553
A03	HLA-A*34:03	409	417	QIAPGQTGK	0.7709
A03	HLA-A*34:03	724	733	TEILPVSMTK	0.6467
A03	HLA-A*34:03	725	733	EILPVSMTK	0.8798
A03	HLA-A*34:03	782	790	FAQVKQIYK	0.569
A03	HLA-A*34:03	787	795	QIYKTPPIK	0.744
A03	HLA-A*34:03	827	835	TLADAGFIK	0.6884
A03	HLA-A*34:03	975	983	SVLNDILSR	0.7688
A03	HLA-A*34:03	1020	1028	ASANLAATK	0.5816
A03	HLA-A*34:03	1064	1073	HVTYVPAQEK	0.7046
A03	HLA-A*34:03	1065	1073	VTYVPAQEK	0.8154
A03	HLA-A*34:03	1099	1107	GTHWFVTQR	0.5926
A03	HLA-A*34:03	1173	1181	NASVVNIQK	0.5921
A03	HLA-A*34:04	30	38	NSFTRGVYY	0.7033
A03	HLA-A*34:04	35	44	GVYYPDKVFR	0.5826
A03	HLA-A*34:04	69	77	HVSGTNGTK	0.6254
A03	HLA-A*34:04	88	97	DGVYFASTEK	0.5696
A03	HLA-A*34:04	89	97	GVYFASTEK	0.81
A03	HLA-A*34:04	142	150	GVYYHKNNK	0.7107
A03	HLA-A*34:04	192	200	FVFKNIDGY	0.5463
A03	HLA-A*34:04	198	206	DGYFKIYSK	0.5064
A03	HLA-A*34:04	258	266	WTAGAAAYY	0.585
A03	HLA-A*34:04	302	310	TLKSFTVEK	0.7513
A03	HLA-A*34:04	311	319	GIYQTSNFR	0.5989
A03	HLA-A*34:04	349	357	SVYAWNRKR	0.7569
A03	HLA-A*34:04	370	378	NSASFSTFK	0.7929
A03	HLA-A*34:04	394	403	NVYADSFVIR	0.553
A03	HLA-A*34:04	409	417	QIAPGQTGK	0.7709
A03	HLA-A*34:04	724	733	TEILPVSMTK	0.6467
A03	HLA-A*34:04	725	733	EILPVSMTK	0.8798
A03	HLA-A*34:04	782	790	FAQVKQIYK	0.569
A03	HLA-A*34:04	787	795	QIYKTPPIK	0.744
A03	HLA-A*34:04	827	835	TLADAGFIK	0.6884
A03	HLA-A*34:04	975	983	SVLNDILSR	0.7688
A03	HLA-A*34:04	1020	1028	ASANLAATK	0.5816
A03	HLA-A*34:04	1064	1073	HVTYVPAQEK	0.7046
A03	HLA-A*34:04	1065	1073	VTYVPAQEK	0.8154
A03	HLA-A*34:04	1099	1107	GTHWFVTQR	0.5926
A03	HLA-A*34:04	1173	1181	NASVVNIQK	0.5921
A03	HLA-A*34:06	30	38	NSFTRGVYY	0.6353
A03	HLA-A*34:06	192	200	FVFKNIDGY	0.6689

A03	HLA-A*34:06	258	266	WTAGAAAYY	0.787
A03	HLA-A*34:06	349	357	SVYAWNRKR	0.6008
A03	HLA-A*34:06	360	369	NCVADYSVLY	0.5204
A03	HLA-A*34:06	361	369	CVADYSVLY	0.6106
A03	HLA-A*34:06	604	612	TSNQVAVLY	0.5152
A03	HLA-A*34:06	725	733	EILPVSMTK	0.7361
A03	HLA-A*34:06	865	873	LTDEMIAQY	0.516
A03	HLA-A*34:06	975	983	SVLNDILSR	0.591
A03	HLA-A*66:01	192	200	FVFKNIDGY	0.6341
A03	HLA-A*66:01	258	266	WTAGAAAYY	0.7115
A03	HLA-A*66:01	725	733	EILPVSMTK	0.6045
A03	HLA-A*66:02	69	77	HVSGTNGTK	0.5701
A03	HLA-A*66:02	192	200	FVFKNIDGY	0.6529
A03	HLA-A*66:02	258	266	WTAGAAAYY	0.7098
A03	HLA-A*66:02	349	357	SVYAWNRKR	0.538
A03	HLA-A*66:02	725	733	EILPVSMTK	0.694
A03	HLA-A*66:03	192	200	FVFKNIDGY	0.6992
A03	HLA-A*66:03	258	266	WTAGAAAYY	0.7192
A03	HLA-A*66:03	349	357	SVYAWNRKR	0.5229
A03	HLA-A*66:04	192	200	FVFKNIDGY	0.5137
A03	HLA-A*66:04	258	266	WTAGAAAYY	0.5766
A03	HLA-A*66:04	725	733	EILPVSMTK	0.603
A03	HLA-A*68:01	30	38	NSFTRGVYY	0.668
A03	HLA-A*68:01	35	44	GVYYPDKVFR	0.7011
A03	HLA-A*68:01	69	77	HVSGTNGTK	0.6905
A03	HLA-A*68:01	69	78	HVSGTNGTKR	0.6265
A03	HLA-A*68:01	88	97	DGVYFASTEK	0.6542
A03	HLA-A*68:01	89	97	GVYFASTEK	0.6612
A03	HLA-A*68:01	94	102	STEKSNIIR	0.5226
A03	HLA-A*68:01	192	200	FVFKNIDGY	0.6109
A03	HLA-A*68:01	198	206	DGYFKIYSK	0.5463
A03	HLA-A*68:01	228	237	DLPIGINITR	0.6101
A03	HLA-A*68:01	258	266	WTAGAAAYY	0.739
A03	HLA-A*68:01	302	310	TLKSFTVEK	0.5381
A03	HLA-A*68:01	311	319	GIYQTSNFR	0.6323
A03	HLA-A*68:01	347	355	FASVYAWNR	0.6502
A03	HLA-A*68:01	349	357	SVYAWNRKR	0.7833
A03	HLA-A*68:01	361	369	CVADYSVLY	0.538
A03	HLA-A*68:01	369	378	YNSASFSTFK	0.5211
A03	HLA-A*68:01	370	378	NSASFSTFK	0.8693
A03	HLA-A*68:01	394	403	NVYADSFVIR	0.8218
A03	HLA-A*68:01	400	408	FVIRGDEVR	0.7176
A03	HLA-A*68:01	409	417	QIAPGQTGK	0.6056
A03	HLA-A*68:01	501	509	NGVGYQPYR	0.5133
A03	HLA-A*68:01	568	577	DIADTTDAVR	0.5526
A03	HLA-A*68:01	604	612	TSNQVAVLY	0.5237
A03	HLA-A*68:01	637	646	STGSNVFQTR	0.6834
A03	HLA-A*68:01	677	685	QTNSPRRAR	0.5628
A03	HLA-A*68:01	725	733	EILPVSMTK	0.8883
A03	HLA-A*68:01	777	786	NTQEVFAQVK	0.5821

A03	HLA-A*68:01	782	790	FAQVKQIYK	0.557
A03	HLA-A*68:01	817	825	FIEDLLFNK	0.5167
A03	HLA-A*68:01	827	835	TLADAGFIK	0.6674
A03	HLA-A*68:01	975	983	SVLNDILSR	0.7755
A03	HLA-A*68:01	1020	1028	ASANLAATK	0.5422
A03	HLA-A*68:01	1064	1073	HVTYVPAQEK	0.6592
A03	HLA-A*68:01	1065	1073	VTYVPAQEK	0.6604
A03	HLA-A*68:01	1099	1107	GTHWFVTQR	0.7781
A03	HLA-A*68:01	1173	1181	NASVVNIQK	0.8236
A03	HLA-A*68:03	30	38	NSFTRGVYY	0.52
A03	HLA-A*68:03	35	44	GVYYPDKVFR	0.583
A03	HLA-A*68:03	69	78	HVSGTNGTKR	0.5195
A03	HLA-A*68:03	192	200	FVFKNIDGY	0.5247
A03	HLA-A*68:03	228	237	DLPIGINITR	0.5626
A03	HLA-A*68:03	258	266	WTAGAAAYY	0.6587
A03	HLA-A*68:03	311	319	GIYQTSNFR	0.5487
A03	HLA-A*68:03	347	355	FASVYAWNR	0.5769
A03	HLA-A*68:03	349	357	SVYAWNRKR	0.6915
A03	HLA-A*68:03	370	378	NSASFSTFK	0.6811
A03	HLA-A*68:03	394	403	NVYADSFVIR	0.7431
A03	HLA-A*68:03	400	408	FVIRGDEVR	0.6382
A03	HLA-A*68:03	637	646	STGSNVFQTR	0.5981
A03	HLA-A*68:03	725	733	EILPVSMTK	0.7062
A03	HLA-A*68:03	975	983	SVLNDILSR	0.7019
A03	HLA-A*68:03	1099	1107	GTHWFVTQR	0.6828
A03	HLA-A*68:03	1173	1181	NASVVNIQK	0.5167
A03	HLA-A*68:04	35	44	GVYYPDKVFR	0.5995
A03	HLA-A*68:04	69	78	HVSGTNGTKR	0.5678
A03	HLA-A*68:04	228	237	DLPIGINITR	0.5843
A03	HLA-A*68:04	258	266	WTAGAAAYY	0.5018
A03	HLA-A*68:04	311	319	GIYQTSNFR	0.5866
A03	HLA-A*68:04	347	355	FASVYAWNR	0.6029
A03	HLA-A*68:04	349	357	SVYAWNRKR	0.7626
A03	HLA-A*68:04	370	378	NSASFSTFK	0.6296
A03	HLA-A*68:04	394	403	NVYADSFVIR	0.7377
A03	HLA-A*68:04	400	408	FVIRGDEVR	0.6688
A03	HLA-A*68:04	568	577	DIADTTDAVR	0.5213
A03	HLA-A*68:04	637	646	STGSNVFQTR	0.6194
A03	HLA-A*68:04	677	685	QTNSPRRAR	0.5976
A03	HLA-A*68:04	725	733	EILPVSMTK	0.6512
A03	HLA-A*68:04	975	983	SVLNDILSR	0.7053
A03	HLA-A*68:04	1099	1107	GTHWFVTQR	0.6927
A03	HLA-A*68:08	30	38	NSFTRGVYY	0.6319
A03	HLA-A*68:08	35	44	GVYYPDKVFR	0.6146
A03	HLA-A*68:08	89	97	GVYFASTEK	0.5888
A03	HLA-A*68:08	192	200	FVFKNIDGY	0.5341
A03	HLA-A*68:08	258	266	WTAGAAAYY	0.6296
A03	HLA-A*68:08	311	319	GIYQTSNFR	0.5259
A03	HLA-A*68:08	349	357	SVYAWNRKR	0.7003
A03	HLA-A*68:08	361	369	CVADYSVLY	0.5107

A03	HLA-A*68:08	370	378	NSASFSTFK	0.7548
A03	HLA-A*68:08	394	403	NVYADSFVIR	0.7003
A03	HLA-A*68:08	725	733	EILPVSMTK	0.8497
A03	HLA-A*68:08	817	825	FIEDLLFNK	0.54
A03	HLA-A*68:08	827	835	TLADAGFIK	0.6538
A03	HLA-A*68:08	975	983	SVLNDILSR	0.7898
A03	HLA-A*68:08	1065	1073	VTYVPAQEK	0.5951
A03	HLA-A*68:08	1099	1107	GTHWFVTQR	0.627
A03	HLA-A*68:08	1173	1181	NASVVNIQK	0.7036
A03	HLA-A*68:09	30	38	NSFTRGVYY	0.5551
A03	HLA-A*68:09	35	44	GVYYPDKVFR	0.5657
A03	HLA-A*68:09	89	97	GVYFASTEK	0.5852
A03	HLA-A*68:09	94	102	STEKSNIIR	0.5242
A03	HLA-A*68:09	258	266	WTAGAAAYY	0.6062
A03	HLA-A*68:09	302	310	TLKSFTVEK	0.5606
A03	HLA-A*68:09	311	319	GIYQTSNFR	0.5449
A03	HLA-A*68:09	349	357	SVYAWNRKR	0.6482
A03	HLA-A*68:09	370	378	NSASFSTFK	0.7717
A03	HLA-A*68:09	394	403	NVYADSFVIR	0.6549
A03	HLA-A*68:09	409	417	QIAPGQTGK	0.5013
A03	HLA-A*68:09	725	733	EILPVSMTK	0.8372
A03	HLA-A*68:09	782	790	FAQVKQIYK	0.5222
A03	HLA-A*68:09	817	825	FIEDLLFNK	0.5307
A03	HLA-A*68:09	827	835	TLADAGFIK	0.7002
A03	HLA-A*68:09	975	983	SVLNDILSR	0.773
A03	HLA-A*68:09	1065	1073	VTYVPAQEK	0.5868
A03	HLA-A*68:09	1099	1107	GTHWFVTQR	0.6191
A03	HLA-A*68:09	1173	1181	NASVVNIQK	0.6784
A03	HLA-A*68:10	35	44	GVYYPDKVFR	0.8083
A03	HLA-A*68:10	69	77	HVSGTNGTK	0.53
A03	HLA-A*68:10	89	97	GVYFASTEK	0.8449
A03	HLA-A*68:10	142	150	GVYYHKNNK	0.6813
A03	HLA-A*68:10	150	158	KSWMESEFR	0.5571
A03	HLA-A*68:10	302	310	TLKSFTVEK	0.7187
A03	HLA-A*68:10	310	319	KGIYQTSNFR	0.5025
A03	HLA-A*68:10	311	319	GIYQTSNFR	0.7383
A03	HLA-A*68:10	319	328	RVQPTESIVR	0.5288
A03	HLA-A*68:10	348	356	ASVYAWNRK	0.6444
A03	HLA-A*68:10	349	357	SVYAWNRKR	0.7606
A03	HLA-A*68:10	369	378	YNSASFSTFK	0.5505
A03	HLA-A*68:10	370	378	NSASFSTFK	0.782
A03	HLA-A*68:10	394	403	NVYADSFVIR	0.5174
A03	HLA-A*68:10	408	417	RQIAPGQTGK	0.6091
A03	HLA-A*68:10	409	417	QIAPGQTGK	0.6387
A03	HLA-A*68:10	454	462	RLFRKSNLK	0.6427
A03	HLA-A*68:10	458	466	KSNLKPFER	0.7028
A03	HLA-A*68:10	529	537	KSTNLVKNK	0.5693
A03	HLA-A*68:10	550	558	GVLTESNKK	0.5463
A03	HLA-A*68:10	637	646	STGSNVFQTR	0.6581
A03	HLA-A*68:10	724	733	TEILPVSMTK	0.5558

A03	HLA-A*68:10	725	733	EILPVSMTK	0.6932
A03	HLA-A*68:10	757	765	GSFCTQLNR	0.5531
A03	HLA-A*68:10	782	790	FAQVKQIYK	0.5621
A03	HLA-A*68:10	787	795	QIYKTPPIK	0.5872
A03	HLA-A*68:10	826	835	VTLADAGFIK	0.6153
A03	HLA-A*68:10	827	835	TLADAGFIK	0.7116
A03	HLA-A*68:10	939	947	SSTASALGK	0.5627
A03	HLA-A*68:10	975	983	SVLNDILSR	0.813
A03	HLA-A*68:10	1019	1028	RASANLAATK	0.5906
A03	HLA-A*68:10	1020	1028	ASANLAATK	0.8515
A03	HLA-A*68:10	1064	1073	HVTYVPAQEK	0.6145
A03	HLA-A*68:10	1065	1073	VTYVPAQEK	0.8181
A03	HLA-A*68:10	1099	1107	GTHWFVTQR	0.8652
A03	HLA-A*68:10	1173	1181	NASVVNIQK	0.5721
A03	HLA-A*68:12	30	38	NSFTRGVYY	0.668
A03	HLA-A*68:12	35	44	GVYYPDKVFR	0.7011
A03	HLA-A*68:12	69	77	HVSGTNGTK	0.6905
A03	HLA-A*68:12	69	78	HVSGTNGTKR	0.6265
A03	HLA-A*68:12	88	97	DGVYFASTEK	0.6542
A03	HLA-A*68:12	89	97	GVYFASTEK	0.6612
A03	HLA-A*68:12	94	102	STEKSNIIR	0.5226
A03	HLA-A*68:12	192	200	FVFKNIDGY	0.6109
A03	HLA-A*68:12	198	206	DGYFKIYSK	0.5463
A03	HLA-A*68:12	228	237	DLPIGINITR	0.6101
A03	HLA-A*68:12	258	266	WTAGAAAYY	0.739
A03	HLA-A*68:12	302	310	TLKSFTVEK	0.5381
A03	HLA-A*68:12	311	319	GIYQTSNFR	0.6323
A03	HLA-A*68:12	347	355	FASVYAWNR	0.6502
A03	HLA-A*68:12	349	357	SVYAWNRKR	0.7833
A03	HLA-A*68:12	361	369	CVADYSVLY	0.538
A03	HLA-A*68:12	369	378	YNSASFSTFK	0.5211
A03	HLA-A*68:12	370	378	NSASFSTFK	0.8693
A03	HLA-A*68:12	394	403	NVYADSFVIR	0.8218
A03	HLA-A*68:12	400	408	FVIRGDEVR	0.7176
A03	HLA-A*68:12	409	417	QIAPGQTGK	0.6056
A03	HLA-A*68:12	501	509	NGVGYQPYR	0.5133
A03	HLA-A*68:12	568	577	DIADTTDAVR	0.5526
A03	HLA-A*68:12	604	612	TSNQVAVLY	0.5237
A03	HLA-A*68:12	637	646	STGSNVFQTR	0.6834
A03	HLA-A*68:12	677	685	QTNSPRRAR	0.5628
A03	HLA-A*68:12	725	733	EILPVSMTK	0.8883
A03	HLA-A*68:12	777	786	NTQEVFAQVK	0.5821
A03	HLA-A*68:12	782	790	FAQVKQIYK	0.557
A03	HLA-A*68:12	817	825	FIEDLLFNK	0.5167
A03	HLA-A*68:12	827	835	TLADAGFIK	0.6674
A03	HLA-A*68:12	975	983	SVLNDILSR	0.7755
A03	HLA-A*68:12	1020	1028	ASANLAATK	0.5422
A03	HLA-A*68:12	1064	1073	HVTYVPAQEK	0.6592
A03	HLA-A*68:12	1065	1073	VTYVPAQEK	0.6604
A03	HLA-A*68:12	1099	1107	GTHWFVTQR	0.7781

A03	HLA-A*68:12	1173	1181	NASVVNIQK	0.8236
A03	HLA-A*68:13	35	44	GVYYPDKVFR	0.8316
A03	HLA-A*68:13	69	77	HVSGTNGTK	0.5191
A03	HLA-A*68:13	89	97	GVYFASTEK	0.8528
A03	HLA-A*68:13	142	150	GVYYHKNNK	0.6657
A03	HLA-A*68:13	258	266	WTAGAAAYY	0.5568
A03	HLA-A*68:13	302	310	TLKSFTVEK	0.7097
A03	HLA-A*68:13	311	319	GIYQTSNFR	0.7417
A03	HLA-A*68:13	348	356	ASVYAWNRK	0.588
A03	HLA-A*68:13	349	357	SVYAWNRKR	0.7769
A03	HLA-A*68:13	369	378	YNSASFSTFK	0.5945
A03	HLA-A*68:13	370	378	NSASFSTFK	0.7984
A03	HLA-A*68:13	394	403	NVYADSFVIR	0.6004
A03	HLA-A*68:13	400	408	FVIRGDEVR	0.5034
A03	HLA-A*68:13	409	417	QIAPGQTGK	0.6035
A03	HLA-A*68:13	454	462	RLFRKSNLK	0.5075
A03	HLA-A*68:13	458	466	KSNLKPFER	0.5406
A03	HLA-A*68:13	637	646	STGSNVFQTR	0.6827
A03	HLA-A*68:13	724	733	TEILPVSMTK	0.541
A03	HLA-A*68:13	725	733	EILPVSMTK	0.7319
A03	HLA-A*68:13	757	765	GSFCTQLNR	0.5489
A03	HLA-A*68:13	782	790	FAQVKQIYK	0.5799
A03	HLA-A*68:13	787	795	QIYKTPPIK	0.5619
A03	HLA-A*68:13	826	835	VTLADAGFIK	0.6185
A03	HLA-A*68:13	827	835	TLADAGFIK	0.6995
A03	HLA-A*68:13	939	947	SSTASALGK	0.5057
A03	HLA-A*68:13	975	983	SVLNDILSR	0.8131
A03	HLA-A*68:13	1020	1028	ASANLAATK	0.8167
A03	HLA-A*68:13	1064	1073	HVTYVPAQEK	0.6451
A03	HLA-A*68:13	1065	1073	VTYVPAQEK	0.823
A03	HLA-A*68:13	1099	1107	GTHWFVTQR	0.8791
A03	HLA-A*68:13	1173	1181	NASVVNIQK	0.58
A03	HLA-A*68:14	35	44	GVYYPDKVFR	0.8083
A03	HLA-A*68:14	69	77	HVSGTNGTK	0.53
A03	HLA-A*68:14	89	97	GVYFASTEK	0.8449
A03	HLA-A*68:14	142	150	GVYYHKNNK	0.6813
A03	HLA-A*68:14	150	158	KSWMESEFR	0.5571
A03	HLA-A*68:14	302	310	TLKSFTVEK	0.7187
A03	HLA-A*68:14	310	319	KGIYQTSNFR	0.5025
A03	HLA-A*68:14	311	319	GIYQTSNFR	0.7383
A03	HLA-A*68:14	319	328	RVQPTESIVR	0.5288
A03	HLA-A*68:14	348	356	ASVYAWNRK	0.6444
A03	HLA-A*68:14	349	357	SVYAWNRKR	0.7606
A03	HLA-A*68:14	369	378	YNSASFSTFK	0.5505
A03	HLA-A*68:14	370	378	NSASFSTFK	0.782
A03	HLA-A*68:14	394	403	NVYADSFVIR	0.5174
A03	HLA-A*68:14	408	417	RQIAPGQTGK	0.6091
A03	HLA-A*68:14	409	417	QIAPGQTGK	0.6387
A03	HLA-A*68:14	454	462	RLFRKSNLK	0.6427
A03	HLA-A*68:14	458	466	KSNLKPFER	0.7028

A03	HLA-A*68:14	529	537	KSTNLVKNK	0.5693
A03	HLA-A*68:14	550	558	GVLTESNKK	0.5463
A03	HLA-A*68:14	637	646	STGSNVFQTR	0.6581
A03	HLA-A*68:14	724	733	TEILPVSMTK	0.5558
A03	HLA-A*68:14	725	733	EILPVSMTK	0.6932
A03	HLA-A*68:14	757	765	GSFCTQLNR	0.5531
A03	HLA-A*68:14	782	790	FAQVKQIYK	0.5621
A03	HLA-A*68:14	787	795	QIYKTPPIK	0.5872
A03	HLA-A*68:14	826	835	VTLADAGFIK	0.6153
A03	HLA-A*68:14	827	835	TLADAGFIK	0.7116
A03	HLA-A*68:14	939	947	SSTASALGK	0.5627
A03	HLA-A*68:14	975	983	SVLNDILSR	0.813
A03	HLA-A*68:14	1019	1028	RASANLAATK	0.5906
A03	HLA-A*68:14	1020	1028	ASANLAATK	0.8515
A03	HLA-A*68:14	1064	1073	HVTYVPAQEK	0.6145
A03	HLA-A*68:14	1065	1073	VTYVPAQEK	0.8181
A03	HLA-A*68:14	1099	1107	GTHWFVTQR	0.8652
A03	HLA-A*68:14	1173	1181	NASVVNIQK	0.5721
A03	HLA-A*68:16	30	38	NSFTRGVYY	0.668
A03	HLA-A*68:16	35	44	GVYYPDKVFR	0.7011
A03	HLA-A*68:16	69	77	HVSGTNGTK	0.6905
A03	HLA-A*68:16	69	78	HVSGTNGTKR	0.6265
A03	HLA-A*68:16	88	97	DGVYFASTEK	0.6542
A03	HLA-A*68:16	89	97	GVYFASTEK	0.6612
A03	HLA-A*68:16	94	102	STEKSNIIR	0.5226
A03	HLA-A*68:16	192	200	FVFKNIDGY	0.6109
A03	HLA-A*68:16	198	206	DGYFKIYSK	0.5463
A03	HLA-A*68:16	228	237	DLPIGINITR	0.6101
A03	HLA-A*68:16	258	266	WTAGAAAYY	0.739
A03	HLA-A*68:16	302	310	TLKSFTVEK	0.5381
A03	HLA-A*68:16	311	319	GIYQTSNFR	0.6323
A03	HLA-A*68:16	347	355	FASVYAWNR	0.6502
A03	HLA-A*68:16	349	357	SVYAWNRKR	0.7833
A03	HLA-A*68:16	361	369	CVADYSVLY	0.538
A03	HLA-A*68:16	369	378	YNSASFSTFK	0.5211
A03	HLA-A*68:16	370	378	NSASFSTFK	0.8693
A03	HLA-A*68:16	394	403	NVYADSFVIR	0.8218
A03	HLA-A*68:16	400	408	FVIRGDEVR	0.7176
A03	HLA-A*68:16	409	417	QIAPGQTGK	0.6056
A03	HLA-A*68:16	501	509	NGVGYQPYR	0.5133
A03	HLA-A*68:16	568	577	DIADTTDAVR	0.5526
A03	HLA-A*68:16	604	612	TSNQVAVLY	0.5237
A03	HLA-A*68:16	637	646	STGSNVFQTR	0.6834
A03	HLA-A*68:16	677	685	QTNSPRRAR	0.5628
A03	HLA-A*68:16	725	733	EILPVSMTK	0.8883
A03	HLA-A*68:16	777	786	NTQEVFAQVK	0.5821
A03	HLA-A*68:16	782	790	FAQVKQIYK	0.557
A03	HLA-A*68:16	817	825	FIEDLLFNK	0.5167
A03	HLA-A*68:16	827	835	TLADAGFIK	0.6674
A03	HLA-A*68:16	975	983	SVLNDILSR	0.7755

A03	HLA-A*68:16	1020	1028	ASANLAATK	0.5422
A03	HLA-A*68:16	1064	1073	HVTYVPAQEK	0.6592
A03	HLA-A*68:16	1065	1073	VTYVPAQEK	0.6604
A03	HLA-A*68:16	1099	1107	GTHWFVTQR	0.7781
A03	HLA-A*68:16	1173	1181	NASVVNIQK	0.8236
A03	HLA-A*68:19	30	38	NSFTRGVYY	0.668
A03	HLA-A*68:19	35	44	GVYYPDKVFR	0.7011
A03	HLA-A*68:19	69	77	HVSGTNGTK	0.6905
A03	HLA-A*68:19	69	78	HVSGTNGTKR	0.6265
A03	HLA-A*68:19	88	97	DGVYFASTEK	0.6542
A03	HLA-A*68:19	89	97	GVYFASTEK	0.6612
A03	HLA-A*68:19	94	102	STEKSNIIR	0.5226
A03	HLA-A*68:19	192	200	FVFKNIDGY	0.6109
A03	HLA-A*68:19	198	206	DGYFKIYSK	0.5463
A03	HLA-A*68:19	228	237	DLPIGINITR	0.6101
A03	HLA-A*68:19	258	266	WTAGAAAYY	0.739
A03	HLA-A*68:19	302	310	TLKSFTVEK	0.5381
A03	HLA-A*68:19	311	319	GIYQTSNFR	0.6323
A03	HLA-A*68:19	347	355	FASVYAWNR	0.6502
A03	HLA-A*68:19	349	357	SVYAWNRKR	0.7833
A03	HLA-A*68:19	361	369	CVADYSVLY	0.538
A03	HLA-A*68:19	369	378	YNSASFSTFK	0.5211
A03	HLA-A*68:19	370	378	NSASFSTFK	0.8693
A03	HLA-A*68:19	394	403	NVYADSFVIR	0.8218
A03	HLA-A*68:19	400	408	FVIRGDEVR	0.7176
A03	HLA-A*68:19	409	417	QIAPGQTGK	0.6056
A03	HLA-A*68:19	501	509	NGVGYQPYR	0.5133
A03	HLA-A*68:19	568	577	DIADTTDAVR	0.5526
A03	HLA-A*68:19	604	612	TSNQVAVLY	0.5237
A03	HLA-A*68:19	637	646	STGSNVFQTR	0.6834
A03	HLA-A*68:19	677	685	QTNSPRRAR	0.5628
A03	HLA-A*68:19	725	733	EILPVSMTK	0.8883
A03	HLA-A*68:19	777	786	NTQEVFAQVK	0.5821
A03	HLA-A*68:19	782	790	FAQVKQIYK	0.557
A03	HLA-A*68:19	817	825	FIEDLLFNK	0.5167
A03	HLA-A*68:19	827	835	TLADAGFIK	0.6674
A03	HLA-A*68:19	975	983	SVLNDILSR	0.7755
A03	HLA-A*68:19	1020	1028	ASANLAATK	0.5422
A03	HLA-A*68:19	1064	1073	HVTYVPAQEK	0.6592
A03	HLA-A*68:19	1065	1073	VTYVPAQEK	0.6604
A03	HLA-A*68:19	1099	1107	GTHWFVTQR	0.7781
A03	HLA-A*68:19	1173	1181	NASVVNIQK	0.8236
A03	HLA-A*68:21	30	38	NSFTRGVYY	0.668
A03	HLA-A*68:21	35	44	GVYYPDKVFR	0.7011
A03	HLA-A*68:21	69	77	HVSGTNGTK	0.6905
A03	HLA-A*68:21	69	78	HVSGTNGTKR	0.6265
A03	HLA-A*68:21	88	97	DGVYFASTEK	0.6542
A03	HLA-A*68:21	89	97	GVYFASTEK	0.6612
A03	HLA-A*68:21	94	102	STEKSNIIR	0.5226
A03	HLA-A*68:21	192	200	FVFKNIDGY	0.6109

A03	HLA-A*68:21	198	206	DGYFKIYSK	0.5463
A03	HLA-A*68:21	228	237	DLPIGINITR	0.6101
A03	HLA-A*68:21	258	266	WTAGAAAYY	0.739
A03	HLA-A*68:21	302	310	TLKSFTVEK	0.5381
A03	HLA-A*68:21	311	319	GIYQTSNFR	0.6323
A03	HLA-A*68:21	347	355	FASVYAWNR	0.6502
A03	HLA-A*68:21	349	357	SVYAWNRKR	0.7833
A03	HLA-A*68:21	361	369	CVADYSVLY	0.538
A03	HLA-A*68:21	369	378	YNSASFSTFK	0.5211
A03	HLA-A*68:21	370	378	NSASFSTFK	0.8693
A03	HLA-A*68:21	394	403	NVYADSFVIR	0.8218
A03	HLA-A*68:21	400	408	FVIRGDEVR	0.7176
A03	HLA-A*68:21	409	417	QIAPGQTGK	0.6056
A03	HLA-A*68:21	501	509	NGVGYQPYR	0.5133
A03	HLA-A*68:21	568	577	DIADTTDAVR	0.5526
A03	HLA-A*68:21	604	612	TSNQVAVLY	0.5237
A03	HLA-A*68:21	637	646	STGSNVFQTR	0.6834
A03	HLA-A*68:21	677	685	QTNSPRRAR	0.5628
A03	HLA-A*68:21	725	733	EILPVSMTK	0.8883
A03	HLA-A*68:21	777	786	NTQEVFAQVK	0.5821
A03	HLA-A*68:21	782	790	FAQVKQIYK	0.557
A03	HLA-A*68:21	817	825	FIEDLLFNK	0.5167
A03	HLA-A*68:21	827	835	TLADAGFIK	0.6674
A03	HLA-A*68:21	975	983	SVLNDILSR	0.7755
A03	HLA-A*68:21	1020	1028	ASANLAATK	0.5422
A03	HLA-A*68:21	1064	1073	HVTYVPAQEK	0.6592
A03	HLA-A*68:21	1065	1073	VTYVPAQEK	0.6604
A03	HLA-A*68:21	1099	1107	GTHWFVTQR	0.7781
A03	HLA-A*68:21	1173	1181	NASVVNIQK	0.8236
A03	HLA-A*68:22	30	38	NSFTRGVYY	0.668
A03	HLA-A*68:22	35	44	GVYYPDKVFR	0.7011
A03	HLA-A*68:22	69	77	HVSGTNGTK	0.6905
A03	HLA-A*68:22	69	78	HVSGTNGTKR	0.6265
A03	HLA-A*68:22	88	97	DGVYFASTEK	0.6542
A03	HLA-A*68:22	89	97	GVYFASTEK	0.6612
A03	HLA-A*68:22	94	102	STEKSNIIR	0.5226
A03	HLA-A*68:22	192	200	FVFKNIDGY	0.6109
A03	HLA-A*68:22	198	206	DGYFKIYSK	0.5463
A03	HLA-A*68:22	228	237	DLPIGINITR	0.6101
A03	HLA-A*68:22	258	266	WTAGAAAYY	0.739
A03	HLA-A*68:22	302	310	TLKSFTVEK	0.5381
A03	HLA-A*68:22	311	319	GIYQTSNFR	0.6323
A03	HLA-A*68:22	347	355	FASVYAWNR	0.6502
A03	HLA-A*68:22	349	357	SVYAWNRKR	0.7833
A03	HLA-A*68:22	361	369	CVADYSVLY	0.538
A03	HLA-A*68:22	369	378	YNSASFSTFK	0.5211
A03	HLA-A*68:22	370	378	NSASFSTFK	0.8693
A03	HLA-A*68:22	394	403	NVYADSFVIR	0.8218
A03	HLA-A*68:22	400	408	FVIRGDEVR	0.7176
A03	HLA-A*68:22	409	417	QIAPGQTGK	0.6056

A03	HLA-A*68:22	501	509	NGVGYQPYR	0.5133
A03	HLA-A*68:22	568	577	DIADTTDAVR	0.5526
A03	HLA-A*68:22	604	612	TSNQVAVLY	0.5237
A03	HLA-A*68:22	637	646	STGSNVFQTR	0.6834
A03	HLA-A*68:22	677	685	QTNSPRRAR	0.5628
A03	HLA-A*68:22	725	733	EILPVSMTK	0.8883
A03	HLA-A*68:22	777	786	NTQEVFAQVK	0.5821
A03	HLA-A*68:22	782	790	FAQVKQIYK	0.557
A03	HLA-A*68:22	817	825	FIEDLLFNK	0.5167
A03	HLA-A*68:22	827	835	TLADAGFIK	0.6674
A03	HLA-A*68:22	975	983	SVLNDILSR	0.7755
A03	HLA-A*68:22	1020	1028	ASANLAATK	0.5422
A03	HLA-A*68:22	1064	1073	HVTYVPAQEK	0.6592
A03	HLA-A*68:22	1065	1073	VTYVPAQEK	0.6604
A03	HLA-A*68:22	1099	1107	GTHWFVTQR	0.7781
A03	HLA-A*68:22	1173	1181	NASVVNIQK	0.8236
A03	HLA-A*68:24	30	38	NSFTRGVYY	0.668
A03	HLA-A*68:24	35	44	GVYYPDKVFR	0.7011
A03	HLA-A*68:24	69	77	HVSGTNGTK	0.6905
A03	HLA-A*68:24	69	78	HVSGTNGTKR	0.6265
A03	HLA-A*68:24	88	97	DGVYFASTEK	0.6542
A03	HLA-A*68:24	89	97	GVYFASTEK	0.6612
A03	HLA-A*68:24	94	102	STEKSNIIR	0.5226
A03	HLA-A*68:24	192	200	FVFKNIDGY	0.6109
A03	HLA-A*68:24	198	206	DGYFKIYSK	0.5463
A03	HLA-A*68:24	228	237	DLPIGINITR	0.6101
A03	HLA-A*68:24	258	266	WTAGAAAYY	0.739
A03	HLA-A*68:24	302	310	TLKSFTVEK	0.5381
A03	HLA-A*68:24	311	319	GIYQTSNFR	0.6323
A03	HLA-A*68:24	347	355	FASVYAWNR	0.6502
A03	HLA-A*68:24	349	357	SVYAWNRKR	0.7833
A03	HLA-A*68:24	361	369	CVADYSVLY	0.538
A03	HLA-A*68:24	369	378	YNSASFSTFK	0.5211
A03	HLA-A*68:24	370	378	NSASFSTFK	0.8693
A03	HLA-A*68:24	394	403	NVYADSFVIR	0.8218
A03	HLA-A*68:24	400	408	FVIRGDEVR	0.7176
A03	HLA-A*68:24	409	417	QIAPGQTGK	0.6056
A03	HLA-A*68:24	501	509	NGVGYQPYR	0.5133
A03	HLA-A*68:24	568	577	DIADTTDAVR	0.5526
A03	HLA-A*68:24	604	612	TSNQVAVLY	0.5237
A03	HLA-A*68:24	637	646	STGSNVFQTR	0.6834
A03	HLA-A*68:24	677	685	QTNSPRRAR	0.5628
A03	HLA-A*68:24	725	733	EILPVSMTK	0.8883
A03	HLA-A*68:24	777	786	NTQEVFAQVK	0.5821
A03	HLA-A*68:24	782	790	FAQVKQIYK	0.557
A03	HLA-A*68:24	817	825	FIEDLLFNK	0.5167
A03	HLA-A*68:24	827	835	TLADAGFIK	0.6674
A03	HLA-A*68:24	975	983	SVLNDILSR	0.7755
A03	HLA-A*68:24	1020	1028	ASANLAATK	0.5422
A03	HLA-A*68:24	1064	1073	HVTYVPAQEK	0.6592

A03	HLA-A*68:24	1065	1073	VTYVPAQEK	0.6604
A03	HLA-A*68:24	1099	1107	GTHWFVTQR	0.7781
A03	HLA-A*68:24	1173	1181	NASVVNIQK	0.8236
A03	HLA-A*68:25	30	38	NSFTRGVYY	0.668
A03	HLA-A*68:25	35	44	GVYYPDKVFR	0.7011
A03	HLA-A*68:25	69	77	HVSGTNGTK	0.6905
A03	HLA-A*68:25	69	78	HVSGTNGTKR	0.6265
A03	HLA-A*68:25	88	97	DGVYFASTEK	0.6542
A03	HLA-A*68:25	89	97	GVYFASTEK	0.6612
A03	HLA-A*68:25	94	102	STEKSNIIR	0.5226
A03	HLA-A*68:25	192	200	FVFKNIDGY	0.6109
A03	HLA-A*68:25	198	206	DGYFKIYSK	0.5463
A03	HLA-A*68:25	228	237	DLPIGINITR	0.6101
A03	HLA-A*68:25	258	266	WTAGAAAYY	0.739
A03	HLA-A*68:25	302	310	TLKSFTVEK	0.5381
A03	HLA-A*68:25	311	319	GIYQTSNFR	0.6323
A03	HLA-A*68:25	347	355	FASVYAWNR	0.6502
A03	HLA-A*68:25	349	357	SVYAWNRKR	0.7833
A03	HLA-A*68:25	361	369	CVADYSVLY	0.538
A03	HLA-A*68:25	369	378	YNSASFSTFK	0.5211
A03	HLA-A*68:25	370	378	NSASFSTFK	0.8693
A03	HLA-A*68:25	394	403	NVYADSFVIR	0.8218
A03	HLA-A*68:25	400	408	FVIRGDEVR	0.7176
A03	HLA-A*68:25	409	417	QIAPGQTGK	0.6056
A03	HLA-A*68:25	501	509	NGVGYQPYR	0.5133
A03	HLA-A*68:25	568	577	DIADTTDAVR	0.5526
A03	HLA-A*68:25	604	612	TSNQVAVLY	0.5237
A03	HLA-A*68:25	637	646	STGSNVFQTR	0.6834
A03	HLA-A*68:25	677	685	QTNSPRRAR	0.5628
A03	HLA-A*68:25	725	733	EILPVSMTK	0.8883
A03	HLA-A*68:25	777	786	NTQEVFAQVK	0.5821
A03	HLA-A*68:25	782	790	FAQVKQIYK	0.557
A03	HLA-A*68:25	817	825	FIEDLLFNK	0.5167
A03	HLA-A*68:25	827	835	TLADAGFIK	0.6674
A03	HLA-A*68:25	975	983	SVLNDILSR	0.7755
A03	HLA-A*68:25	1020	1028	ASANLAATK	0.5422
A03	HLA-A*68:25	1064	1073	HVTYVPAQEK	0.6592
A03	HLA-A*68:25	1065	1073	VTYVPAQEK	0.6604
A03	HLA-A*68:25	1099	1107	GTHWFVTQR	0.7781
A03	HLA-A*68:25	1173	1181	NASVVNIQK	0.8236
A03	HLA-A*68:26	258	266	WTAGAAAYY	0.5231
A03	HLA-A*68:26	370	378	NSASFSTFK	0.5588
A03	HLA-A*68:26	725	733	EILPVSMTK	0.699
A03	HLA-A*68:26	827	835	TLADAGFIK	0.5161
A03	HLA-A*74:01	349	357	SVYAWNRKR	0.536
A03	HLA-A*74:02	349	357	SVYAWNRKR	0.536
A03	HLA-A*74:03	349	357	SVYAWNRKR	0.536
A03	HLA-A*74:05	35	44	GVYYPDKVFR	0.5051
A03	HLA-A*74:05	349	357	SVYAWNRKR	0.5895
A03	HLA-A*74:05	454	462	RLFRKSNLK	0.5208

A03	HLA-A*74:05	1099	1107	GTHWFVTQR	0.5226
A03	HLA-A*74:07	35	44	GVYYPDKVFR	0.5118
A03	HLA-A*74:07	349	357	SVYAWNRKR	0.6319
A03	HLA-A*74:07	1099	1107	GTHWFVTQR	0.5316
A03	HLA-A*74:08	349	357	SVYAWNRKR	0.536
A03	HLA-A*74:09	349	357	SVYAWNRKR	0.536
A03	HLA-A*74:11	349	357	SVYAWNRKR	0.536
A24	HLA-A*23:01	57	65	PFFSNVTWF	0.9141
A24	HLA-A*23:01	78	86	RFDNPVLPF	0.8396
A24	HLA-A*23:01	143	152	VYYHKNNKSW	0.7142
A24	HLA-A*23:01	144	152	YYHKNNKSW	0.8036
A24	HLA-A*23:01	159	168	VYSSANNCTF	0.917
A24	HLA-A*23:01	169	177	EYVSQPFLM	0.8499
A24	HLA-A*23:01	193	201	VFKNIDGYF	0.7818
A24	HLA-A*23:01	203	212	IYSKHTPINL	0.6028
A24	HLA-A*23:01	264	275	AYYVGYLQPRTF	0.7912
A24	HLA-A*23:01	265	275	YYVGYLQPRTF	0.879
A24	HLA-A*23:01	268	277	GYLQPRTFLL	0.7046
A24	HLA-A*23:01	269	277	YLQPRTFLL	0.5573
A24	HLA-A*23:01	312	320	IYQTSNFRV	0.6966
A24	HLA-A*23:01	328	338	RFPNITNLCPF	0.8158
A24	HLA-A*23:01	368	377	LYNSASFSTF	0.8553
A24	HLA-A*23:01	395	402	VYADSFVI	0.5149
A24	HLA-A*23:01	448	456	NYNYLYRLF	0.8881
A24	HLA-A*23:01	488	497	CYFPLQSYGF	0.6136
A24	HLA-A*23:01	489	497	YFPLQSYGF	0.9108
A24	HLA-A*23:01	504	515	GYQPYRVVVLSF	0.667
A24	HLA-A*23:01	507	515	PYRVVVLSF	0.8298
A24	HLA-A*23:01	558	565	KFLPFQQF	0.7881
A24	HLA-A*23:01	634	643	RVYSTGSNVF	0.5782
A24	HLA-A*23:01	635	643	VYSTGSNVF	0.934
A24	HLA-A*23:01	788	797	IYKTPPIKDF	0.9066
A24	HLA-A*23:01	816	823	SFIEDLLF	0.7909
A24	HLA-A*23:01	880	888	GTITSGWTF	0.5317
A24	HLA-A*23:01	898	906	FAMQMAYRF	0.5028
A24	HLA-A*23:01	1066	1075	TYVPAQEKNF	0.9478
A24	HLA-A*23:01	1094	1102	VFVSNGTHW	0.8761
A24	HLA-A*23:01	1094	1103	VFVSNGTHWF	0.8258
A24	HLA-A*23:01	1101	1109	HWFVTQRNF	0.7845
A24	HLA-A*23:01	1137	1148	VYDPLQPELDSF	0.7515
A24	HLA-A*23:01	1208	1216	QYIKWPWYI	0.9638
A24	HLA-A*23:01	1208	1217	QYIKWPWYIW	0.7814
A24	HLA-A*23:01	1211	1220	KWPWYIWLGF	0.6037
A24	HLA-A*23:01	1216	1224	IWLGFIAGL	0.5925
A24	HLA-A*23:02	57	65	PFFSNVTWF	0.8811
A24	HLA-A*23:02	143	152	VYYHKNNKSW	0.7728
A24	HLA-A*23:02	144	152	YYHKNNKSW	0.8613
A24	HLA-A*23:02	159	168	VYSSANNCTF	0.9503
A24	HLA-A*23:02	169	177	EYVSQPFLM	0.8797
A24	HLA-A*23:02	193	201	VFKNIDGYF	0.8456

A24	HLA-A*23:02	203	212	IYSKHTPINL	0.6962
A24	HLA-A*23:02	264	275	AYYVGYLQPRTF	0.759
A24	HLA-A*23:02	265	275	YYVGYLQPRTF	0.9004
A24	HLA-A*23:02	268	277	GYLQPRTFLL	0.6697
A24	HLA-A*23:02	269	277	YLQPRTFLL	0.5368
A24	HLA-A*23:02	312	320	IYQTSNFRV	0.6415
A24	HLA-A*23:02	328	338	RFPNITNLCPF	0.8669
A24	HLA-A*23:02	368	377	LYNSASFSTF	0.901
A24	HLA-A*23:02	448	456	NYNYLYRLF	0.9202
A24	HLA-A*23:02	488	497	CYFPLQSYGF	0.6273
A24	HLA-A*23:02	489	497	YFPLQSYGF	0.9384
A24	HLA-A*23:02	504	515	GYQPYRVVVLSF	0.6274
A24	HLA-A*23:02	507	515	PYRVVVLSF	0.8272
A24	HLA-A*23:02	634	643	RVYSTGSNVF	0.7093
A24	HLA-A*23:02	635	643	VYSTGSNVF	0.9475
A24	HLA-A*23:02	755	763	QYGSFCTQL	0.5573
A24	HLA-A*23:02	788	797	IYKTPPIKDF	0.9187
A24	HLA-A*23:02	1066	1075	TYVPAQEKNF	0.9671
A24	HLA-A*23:02	1094	1102	VFVSNGTHW	0.8829
A24	HLA-A*23:02	1094	1103	VFVSNGTHWF	0.8701
A24	HLA-A*23:02	1101	1109	HWFVTQRNF	0.7966
A24	HLA-A*23:02	1208	1216	QYIKWPWYI	0.9423
A24	HLA-A*23:02	1208	1217	QYIKWPWYIW	0.7679
A24	HLA-A*23:02	1211	1220	KWPWYIWLGF	0.5976
A24	HLA-A*23:03	57	65	PFFSNVTWF	0.9141
A24	HLA-A*23:03	78	86	RFDNPVLPF	0.8396
A24	HLA-A*23:03	143	152	VYYHKNNKSW	0.7142
A24	HLA-A*23:03	144	152	YYHKNNKSW	0.8036
A24	HLA-A*23:03	159	168	VYSSANNCTF	0.917
A24	HLA-A*23:03	169	177	EYVSQPFLM	0.8499
A24	HLA-A*23:03	193	201	VFKNIDGYF	0.7818
A24	HLA-A*23:03	203	212	IYSKHTPINL	0.6028
A24	HLA-A*23:03	264	275	AYYVGYLQPRTF	0.7912
A24	HLA-A*23:03	265	275	YYVGYLQPRTF	0.879
A24	HLA-A*23:03	268	277	GYLQPRTFLL	0.7046
A24	HLA-A*23:03	269	277	YLQPRTFLL	0.5573
A24	HLA-A*23:03	312	320	IYQTSNFRV	0.6966
A24	HLA-A*23:03	328	338	RFPNITNLCPF	0.8158
A24	HLA-A*23:03	368	377	LYNSASFSTF	0.8553
A24	HLA-A*23:03	395	402	VYADSFVI	0.5149
A24	HLA-A*23:03	448	456	NYNYLYRLF	0.8881
A24	HLA-A*23:03	488	497	CYFPLQSYGF	0.6136
A24	HLA-A*23:03	489	497	YFPLQSYGF	0.9108
A24	HLA-A*23:03	504	515	GYQPYRVVVLSF	0.667
A24	HLA-A*23:03	507	515	PYRVVVLSF	0.8298
A24	HLA-A*23:03	558	565	KFLPFQQF	0.7881
A24	HLA-A*23:03	634	643	RVYSTGSNVF	0.5782
A24	HLA-A*23:03	635	643	VYSTGSNVF	0.934
A24	HLA-A*23:03	788	797	IYKTPPIKDF	0.9066
A24	HLA-A*23:03	816	823	SFIEDLLF	0.7909

A24	HLA-A*23:03	880	888	GTITSGWTF	0.5317
A24	HLA-A*23:03	898	906	FAMQMAYRF	0.5028
A24	HLA-A*23:03	1066	1075	TYVPAQEKNF	0.9478
A24	HLA-A*23:03	1094	1102	VFVSNGTHW	0.8761
A24	HLA-A*23:03	1094	1103	VFVSNGTHWF	0.8258
A24	HLA-A*23:03	1101	1109	HWFVTQRNF	0.7845
A24	HLA-A*23:03	1137	1148	VYDPLQPELDSF	0.7515
A24	HLA-A*23:03	1208	1216	QYIKWPWYI	0.9638
A24	HLA-A*23:03	1208	1217	QYIKWPWYIW	0.7814
A24	HLA-A*23:03	1211	1220	KWPWYIWLGF	0.6037
A24	HLA-A*23:03	1216	1224	IWLGFIAGL	0.5925
A24	HLA-A*23:04	36	43	VYYPDKVF	0.9008
A24	HLA-A*23:04	37	48	YYPDKVFRSSVL	0.5679
A24	HLA-A*23:04	57	65	PFFSNVTWF	0.9013
A24	HLA-A*23:04	77	86	KRFDNPVLPF	0.6675
A24	HLA-A*23:04	78	86	RFDNPVLPF	0.9308
A24	HLA-A*23:04	143	152	VYYHKNNKSW	0.7934
A24	HLA-A*23:04	144	152	YYHKNNKSW	0.9179
A24	HLA-A*23:04	151	159	SWMESEFRV	0.667
A24	HLA-A*23:04	159	168	VYSSANNCTF	0.9321
A24	HLA-A*23:04	167	175	TFEYVSQPF	0.5826
A24	HLA-A*23:04	168	177	FEYVSQPFLM	0.5386
A24	HLA-A*23:04	169	177	EYVSQPFLM	0.8708
A24	HLA-A*23:04	193	201	VFKNIDGYF	0.8925
A24	HLA-A*23:04	203	212	IYSKHTPINL	0.679
A24	HLA-A*23:04	247	258	SYLTPGDSSSGW	0.6431
A24	HLA-A*23:04	264	275	AYYVGYLQPRTF	0.8651
A24	HLA-A*23:04	265	275	YYVGYLQPRTF	0.926
A24	HLA-A*23:04	265	276	YYVGYLQPRTFL	0.5396
A24	HLA-A*23:04	267	276	VGYLQPRTFL	0.6172
A24	HLA-A*23:04	268	275	GYLQPRTF	0.7597
A24	HLA-A*23:04	268	276	GYLQPRTFL	0.9029
A24	HLA-A*23:04	268	277	GYLQPRTFLL	0.8435
A24	HLA-A*23:04	269	277	YLQPRTFLL	0.5793
A24	HLA-A*23:04	312	320	IYQTSNFRV	0.807
A24	HLA-A*23:04	328	338	RFPNITNLCPF	0.8874
A24	HLA-A*23:04	345	353	TRFASVYAW	0.5445
A24	HLA-A*23:04	346	353	RFASVYAW	0.6882
A24	HLA-A*23:04	368	377	LYNSASFSTF	0.8917
A24	HLA-A*23:04	395	402	VYADSFVI	0.5709
A24	HLA-A*23:04	448	456	NYNYLYRLF	0.8977
A24	HLA-A*23:04	488	497	CYFPLQSYGF	0.7405
A24	HLA-A*23:04	489	497	YFPLQSYGF	0.9665
A24	HLA-A*23:04	504	512	GYQPYRVVV	0.6984
A24	HLA-A*23:04	504	513	GYQPYRVVVL	0.6433
A24	HLA-A*23:04	504	515	GYQPYRVVVLSF	0.8025
A24	HLA-A*23:04	507	515	PYRVVVLSF	0.8366
A24	HLA-A*23:04	558	565	KFLPFQQF	0.8975
A24	HLA-A*23:04	634	643	RVYSTGSNVF	0.7676
A24	HLA-A*23:04	635	643	VYSTGSNVF	0.9621

A24	HLA-A*23:04	659	670	SYECDIPIGAGI	0.5668
A24	HLA-A*23:04	706	714	AYSNNSIAI	0.7351
A24	HLA-A*23:04	755	763	QYGSFCTQL	0.6733
A24	HLA-A*23:04	788	797	IYKTPPIKDF	0.9258
A24	HLA-A*23:04	816	823	SFIEDLLF	0.8315
A24	HLA-A*23:04	880	888	GTITSGWTF	0.6365
A24	HLA-A*23:04	898	906	FAMQMAYRF	0.5384
A24	HLA-A*23:04	1051	1062	SFPQSAPHGVVF	0.7156
A24	HLA-A*23:04	1066	1075	TYVPAQEKNF	0.9578
A24	HLA-A*23:04	1094	1102	VFVSNGTHW	0.9161
A24	HLA-A*23:04	1094	1103	VFVSNGTHWF	0.829
A24	HLA-A*23:04	1101	1109	HWFVTQRNF	0.9099
A24	HLA-A*23:04	1137	1145	VYDPLQPEL	0.9435
A24	HLA-A*23:04	1137	1148	VYDPLQPELDSF	0.802
A24	HLA-A*23:04	1147	1156	SFKEELDKYF	0.5695
A24	HLA-A*23:04	1205	1212	KYEQYIKW	0.612
A24	HLA-A*23:04	1208	1216	QYIKWPWYI	0.9656
A24	HLA-A*23:04	1208	1217	QYIKWPWYIW	0.7453
A24	HLA-A*23:04	1211	1220	KWPWYIWLGF	0.7165
A24	HLA-A*23:04	1216	1224	IWLGFIAGL	0.7942
A24	HLA-A*23:04	1219	1227	GFIAGLIAI	0.5361
A24	HLA-A*23:06	57	65	PFFSNVTWF	0.9141
A24	HLA-A*23:06	78	86	RFDNPVLPF	0.8396
A24	HLA-A*23:06	143	152	VYYHKNNKSW	0.7142
A24	HLA-A*23:06	144	152	YYHKNNKSW	0.8036
A24	HLA-A*23:06	159	168	VYSSANNCTF	0.917
A24	HLA-A*23:06	169	177	EYVSQPFLM	0.8499
A24	HLA-A*23:06	193	201	VFKNIDGYF	0.7818
A24	HLA-A*23:06	203	212	IYSKHTPINL	0.6028
A24	HLA-A*23:06	264	275	AYYVGYLQPRTF	0.7912
A24	HLA-A*23:06	265	275	YYVGYLQPRTF	0.879
A24	HLA-A*23:06	268	277	GYLQPRTFLL	0.7046
A24	HLA-A*23:06	269	277	YLQPRTFLL	0.5573
A24	HLA-A*23:06	312	320	IYQTSNFRV	0.6966
A24	HLA-A*23:06	328	338	RFPNITNLCPF	0.8158
A24	HLA-A*23:06	368	377	LYNSASFSTF	0.8553
A24	HLA-A*23:06	395	402	VYADSFVI	0.5149
A24	HLA-A*23:06	448	456	NYNYLYRLF	0.8881
A24	HLA-A*23:06	488	497	CYFPLQSYGF	0.6136
A24	HLA-A*23:06	489	497	YFPLQSYGF	0.9108
A24	HLA-A*23:06	504	515	GYQPYRVVVLSF	0.667
A24	HLA-A*23:06	507	515	PYRVVVLSF	0.8298
A24	HLA-A*23:06	558	565	KFLPFQQF	0.7881
A24	HLA-A*23:06	634	643	RVYSTGSNVF	0.5782
A24	HLA-A*23:06	635	643	VYSTGSNVF	0.934
A24	HLA-A*23:06	788	797	IYKTPPIKDF	0.9066
A24	HLA-A*23:06	816	823	SFIEDLLF	0.7909
A24	HLA-A*23:06	880	888	GTITSGWTF	0.5317
A24	HLA-A*23:06	898	906	FAMQMAYRF	0.5028
A24	HLA-A*23:06	1066	1075	TYVPAQEKNF	0.9478

A24	HLA-A*23:06	1094	1102	VFVSNGTHW	0.8761
A24	HLA-A*23:06	1094	1103	VFVSNGTHWF	0.8258
A24	HLA-A*23:06	1101	1109	HWFVTQRNF	0.7845
A24	HLA-A*23:06	1137	1148	VYDPLQPELDSF	0.7515
A24	HLA-A*23:06	1208	1216	QYIKWPWYI	0.9638
A24	HLA-A*23:06	1208	1217	QYIKWPWYIW	0.7814
A24	HLA-A*23:06	1211	1220	KWPWYIWLGF	0.6037
A24	HLA-A*23:06	1216	1224	IWLGFIAGL	0.5925
A24	HLA-A*23:10	57	65	PFFSNVTWF	0.8852
A24	HLA-A*23:10	78	86	RFDNPVLPF	0.8057
A24	HLA-A*23:10	159	168	VYSSANNCTF	0.8567
A24	HLA-A*23:10	169	177	EYVSQPFLM	0.8502
A24	HLA-A*23:10	264	275	AYYVGYLQPRTF	0.6408
A24	HLA-A*23:10	265	275	YYVGYLQPRTF	0.7982
A24	HLA-A*23:10	268	277	GYLQPRTFLL	0.6349
A24	HLA-A*23:10	269	277	YLQPRTFLL	0.5339
A24	HLA-A*23:10	312	320	IYQTSNFRV	0.5674
A24	HLA-A*23:10	328	338	RFPNITNLCPF	0.6995
A24	HLA-A*23:10	368	377	LYNSASFSTF	0.7785
A24	HLA-A*23:10	448	456	NYNYLYRLF	0.8162
A24	HLA-A*23:10	488	497	CYFPLQSYGF	0.5004
A24	HLA-A*23:10	489	497	YFPLQSYGF	0.8984
A24	HLA-A*23:10	504	515	GYQPYRVVVLSF	0.5485
A24	HLA-A*23:10	507	515	PYRVVVLSF	0.7181
A24	HLA-A*23:10	635	643	VYSTGSNVF	0.8708
A24	HLA-A*23:10	816	823	SFIEDLLF	0.7518
A24	HLA-A*23:10	880	888	GTITSGWTF	0.6006
A24	HLA-A*23:10	898	906	FAMQMAYRF	0.5102
A24	HLA-A*23:10	1066	1075	TYVPAQEKNF	0.9105
A24	HLA-A*23:10	1094	1102	VFVSNGTHW	0.8044
A24	HLA-A*23:10	1094	1103	VFVSNGTHWF	0.7522
A24	HLA-A*23:10	1101	1109	HWFVTQRNF	0.6918
A24	HLA-A*23:10	1137	1148	VYDPLQPELDSF	0.682
A24	HLA-A*23:10	1208	1216	QYIKWPWYI	0.9438
A24	HLA-A*23:10	1208	1217	QYIKWPWYIW	0.6787
A24	HLA-A*23:10	1216	1224	IWLGFIAGL	0.5657
A24	HLA-A*24:02	37	48	YYPDKVFRSSVL	0.601
A24	HLA-A*24:02	57	65	PFFSNVTWF	0.8728
A24	HLA-A*24:02	78	86	RFDNPVLPF	0.8538
A24	HLA-A*24:02	143	152	VYYHKNNKSW	0.7378
A24	HLA-A*24:02	144	152	YYHKNNKSW	0.8416
A24	HLA-A*24:02	151	159	SWMESEFRV	0.5046
A24	HLA-A*24:02	159	168	VYSSANNCTF	0.9582
A24	HLA-A*24:02	167	175	TFEYVSQPF	0.5365
A24	HLA-A*24:02	169	177	EYVSQPFLM	0.8484
A24	HLA-A*24:02	203	212	IYSKHTPINL	0.7085
A24	HLA-A*24:02	264	275	AYYVGYLQPRTF	0.8037
A24	HLA-A*24:02	265	275	YYVGYLQPRTF	0.9005
A24	HLA-A*24:02	268	277	GYLQPRTFLL	0.731
A24	HLA-A*24:02	269	277	YLQPRTFLL	0.5359

A24	HLA-A*24:02	312	320	IYQTSNFRV	0.785
A24	HLA-A*24:02	328	338	RFPNITNLCPF	0.8789
A24	HLA-A*24:02	346	353	RFASVYAW	0.521
A24	HLA-A*24:02	368	377	LYNSASFSTF	0.914
A24	HLA-A*24:02	395	402	VYADSFVI	0.6394
A24	HLA-A*24:02	448	456	NYNYLYRLF	0.9353
A24	HLA-A*24:02	488	497	CYFPLQSYGF	0.5885
A24	HLA-A*24:02	489	497	YFPLQSYGF	0.9442
A24	HLA-A*24:02	504	515	GYQPYRVVVLSF	0.6707
A24	HLA-A*24:02	507	515	PYRVVVLSF	0.8166
A24	HLA-A*24:02	558	565	KFLPFQQF	0.6948
A24	HLA-A*24:02	634	643	RVYSTGSNVF	0.7146
A24	HLA-A*24:02	635	643	VYSTGSNVF	0.9696
A24	HLA-A*24:02	755	763	QYGSFCTQL	0.5614
A24	HLA-A*24:02	788	797	IYKTPPIKDF	0.9226
A24	HLA-A*24:02	816	823	SFIEDLLF	0.7253
A24	HLA-A*24:02	1066	1075	TYVPAQEKNF	0.9479
A24	HLA-A*24:02	1094	1102	VFVSNGTHW	0.8658
A24	HLA-A*24:02	1094	1103	VFVSNGTHWF	0.8335
A24	HLA-A*24:02	1101	1109	HWFVTQRNF	0.754
A24	HLA-A*24:02	1137	1148	VYDPLQPELDSF	0.8174
A24	HLA-A*24:02	1208	1216	QYIKWPWYI	0.9613
A24	HLA-A*24:02	1208	1217	QYIKWPWYIW	0.7582
A24	HLA-A*24:02	1211	1220	KWPWYIWLGF	0.6582
A24	HLA-A*24:03	36	43	VYYPDKVF	0.8969
A24	HLA-A*24:03	37	48	YYPDKVFRSSVL	0.7149
A24	HLA-A*24:03	57	65	PFFSNVTWF	0.8822
A24	HLA-A*24:03	77	86	KRFDNPVLPF	0.6134
A24	HLA-A*24:03	78	86	RFDNPVLPF	0.9523
A24	HLA-A*24:03	143	152	VYYHKNNKSW	0.8351
A24	HLA-A*24:03	144	152	YYHKNNKSW	0.9523
A24	HLA-A*24:03	151	159	SWMESEFRV	0.7048
A24	HLA-A*24:03	159	168	VYSSANNCTF	0.9732
A24	HLA-A*24:03	167	175	TFEYVSQPF	0.6889
A24	HLA-A*24:03	169	177	EYVSQPFLM	0.8798
A24	HLA-A*24:03	193	201	VFKNIDGYF	0.8984
A24	HLA-A*24:03	203	212	IYSKHTPINL	0.8029
A24	HLA-A*24:03	247	258	SYLTPGDSSSGW	0.7197
A24	HLA-A*24:03	264	275	AYYVGYLQPRTF	0.8966
A24	HLA-A*24:03	265	275	YYVGYLQPRTF	0.9506
A24	HLA-A*24:03	265	276	YYVGYLQPRTFL	0.6242
A24	HLA-A*24:03	267	276	VGYLQPRTFL	0.7184
A24	HLA-A*24:03	268	275	GYLQPRTF	0.7874
A24	HLA-A*24:03	268	276	GYLQPRTFL	0.9337
A24	HLA-A*24:03	268	277	GYLQPRTFLL	0.8923
A24	HLA-A*24:03	312	320	IYQTSNFRV	0.8841
A24	HLA-A*24:03	328	338	RFPNITNLCPF	0.9389
A24	HLA-A*24:03	346	353	RFASVYAW	0.7335
A24	HLA-A*24:03	350	358	VYAWNRKRI	0.6686
A24	HLA-A*24:03	368	377	LYNSASFSTF	0.9453

A24	HLA-A*24:03	379	387	CYGVSPTKL	0.6202
A24	HLA-A*24:03	395	402	VYADSFVI	0.6956
A24	HLA-A*24:03	448	456	NYNYLYRLF	0.9379
A24	HLA-A*24:03	488	497	CYFPLQSYGF	0.7404
A24	HLA-A*24:03	489	497	YFPLQSYGF	0.9778
A24	HLA-A*24:03	504	512	GYQPYRVVV	0.7982
A24	HLA-A*24:03	504	513	GYQPYRVVVL	0.7444
A24	HLA-A*24:03	504	515	GYQPYRVVVLSF	0.8581
A24	HLA-A*24:03	507	515	PYRVVVLSF	0.8533
A24	HLA-A*24:03	558	565	KFLPFQQF	0.8636
A24	HLA-A*24:03	632	643	TWRVYSTGSNVF	0.5957
A24	HLA-A*24:03	634	643	RVYSTGSNVF	0.8822
A24	HLA-A*24:03	635	643	VYSTGSNVF	0.9872
A24	HLA-A*24:03	659	670	SYECDIPIGAGI	0.6187
A24	HLA-A*24:03	706	714	AYSNNSIAI	0.8828
A24	HLA-A*24:03	755	763	QYGSFCTQL	0.7929
A24	HLA-A*24:03	788	797	IYKTPPIKDF	0.9538
A24	HLA-A*24:03	816	823	SFIEDLLF	0.8244
A24	HLA-A*24:03	1051	1062	SFPQSAPHGVVF	0.8397
A24	HLA-A*24:03	1066	1075	TYVPAQEKNF	0.97
A24	HLA-A*24:03	1094	1102	VFVSNGTHW	0.9343
A24	HLA-A*24:03	1094	1103	VFVSNGTHWF	0.8723
A24	HLA-A*24:03	1101	1109	HWFVTQRNF	0.9047
A24	HLA-A*24:03	1137	1145	VYDPLQPEL	0.9496
A24	HLA-A*24:03	1137	1148	VYDPLQPELDSF	0.8822
A24	HLA-A*24:03	1147	1156	SFKEELDKYF	0.6438
A24	HLA-A*24:03	1205	1212	KYEQYIKW	0.6328
A24	HLA-A*24:03	1208	1216	QYIKWPWYI	0.9753
A24	HLA-A*24:03	1208	1217	QYIKWPWYIW	0.7703
A24	HLA-A*24:03	1211	1220	KWPWYIWLGF	0.7747
A24	HLA-A*24:03	1216	1224	IWLGFIAGL	0.7773
A24	HLA-A*24:05	37	48	YYPDKVFRSSVL	0.601
A24	HLA-A*24:05	57	65	PFFSNVTWF	0.8728
A24	HLA-A*24:05	78	86	RFDNPVLPF	0.8538
A24	HLA-A*24:05	143	152	VYYHKNNKSW	0.7378
A24	HLA-A*24:05	144	152	YYHKNNKSW	0.8416
A24	HLA-A*24:05	151	159	SWMESEFRV	0.5046
A24	HLA-A*24:05	159	168	VYSSANNCTF	0.9582
A24	HLA-A*24:05	167	175	TFEYVSQPF	0.5365
A24	HLA-A*24:05	169	177	EYVSQPFLM	0.8484
A24	HLA-A*24:05	203	212	IYSKHTPINL	0.7085
A24	HLA-A*24:05	264	275	AYYVGYLQPRTF	0.8037
A24	HLA-A*24:05	265	275	YYVGYLQPRTF	0.9005
A24	HLA-A*24:05	268	277	GYLQPRTFLL	0.731
A24	HLA-A*24:05	269	277	YLQPRTFLL	0.5359
A24	HLA-A*24:05	312	320	IYQTSNFRV	0.785
A24	HLA-A*24:05	328	338	RFPNITNLCPF	0.8789
A24	HLA-A*24:05	346	353	RFASVYAW	0.521
A24	HLA-A*24:05	368	377	LYNSASFSTF	0.914
A24	HLA-A*24:05	395	402	VYADSFVI	0.6394

A24	HLA-A*24:05	448	456	NYNYLYRLF	0.9353
A24	HLA-A*24:05	488	497	CYFPLQSYGF	0.5885
A24	HLA-A*24:05	489	497	YFPLQSYGF	0.9442
A24	HLA-A*24:05	504	515	GYQPYRVVVLSF	0.6707
A24	HLA-A*24:05	507	515	PYRVVVLSF	0.8166
A24	HLA-A*24:05	558	565	KFLPFQQF	0.6948
A24	HLA-A*24:05	634	643	RVYSTGSNVF	0.7146
A24	HLA-A*24:05	635	643	VYSTGSNVF	0.9696
A24	HLA-A*24:05	755	763	QYGSFCTQL	0.5614
A24	HLA-A*24:05	788	797	IYKTPPIKDF	0.9226
A24	HLA-A*24:05	816	823	SFIEDLLF	0.7253
A24	HLA-A*24:05	1066	1075	TYVPAQEKNF	0.9479
A24	HLA-A*24:05	1094	1102	VFVSNGTHW	0.8658
A24	HLA-A*24:05	1094	1103	VFVSNGTHWF	0.8335
A24	HLA-A*24:05	1101	1109	HWFVTQRNF	0.754
A24	HLA-A*24:05	1137	1148	VYDPLQPELDSF	0.8174
A24	HLA-A*24:05	1208	1216	QYIKWPWYI	0.9613
A24	HLA-A*24:05	1208	1217	QYIKWPWYIW	0.7582
A24	HLA-A*24:05	1211	1220	KWPWYIWLGF	0.6582
A24	HLA-A*24:06	57	65	PFFSNVTWF	0.8811
A24	HLA-A*24:06	143	152	VYYHKNNKSW	0.7728
A24	HLA-A*24:06	144	152	YYHKNNKSW	0.8613
A24	HLA-A*24:06	159	168	VYSSANNCTF	0.9503
A24	HLA-A*24:06	169	177	EYVSQPFLM	0.8797
A24	HLA-A*24:06	193	201	VFKNIDGYF	0.8456
A24	HLA-A*24:06	203	212	IYSKHTPINL	0.6962
A24	HLA-A*24:06	264	275	AYYVGYLQPRTF	0.759
A24	HLA-A*24:06	265	275	YYVGYLQPRTF	0.9004
A24	HLA-A*24:06	268	277	GYLQPRTFLL	0.6697
A24	HLA-A*24:06	269	277	YLQPRTFLL	0.5368
A24	HLA-A*24:06	312	320	IYQTSNFRV	0.6415
A24	HLA-A*24:06	328	338	RFPNITNLCPF	0.8669
A24	HLA-A*24:06	368	377	LYNSASFSTF	0.901
A24	HLA-A*24:06	448	456	NYNYLYRLF	0.9202
A24	HLA-A*24:06	488	497	CYFPLQSYGF	0.6273
A24	HLA-A*24:06	489	497	YFPLQSYGF	0.9384
A24	HLA-A*24:06	504	515	GYQPYRVVVLSF	0.6274
A24	HLA-A*24:06	507	515	PYRVVVLSF	0.8272
A24	HLA-A*24:06	634	643	RVYSTGSNVF	0.7093
A24	HLA-A*24:06	635	643	VYSTGSNVF	0.9475
A24	HLA-A*24:06	755	763	QYGSFCTQL	0.5573
A24	HLA-A*24:06	788	797	IYKTPPIKDF	0.9187
A24	HLA-A*24:06	1066	1075	TYVPAQEKNF	0.9671
A24	HLA-A*24:06	1094	1102	VFVSNGTHW	0.8829
A24	HLA-A*24:06	1094	1103	VFVSNGTHWF	0.8701
A24	HLA-A*24:06	1101	1109	HWFVTQRNF	0.7966
A24	HLA-A*24:06	1208	1216	QYIKWPWYI	0.9423
A24	HLA-A*24:06	1208	1217	QYIKWPWYIW	0.7679
A24	HLA-A*24:06	1211	1220	KWPWYIWLGF	0.5976
A24	HLA-A*24:08	144	152	YYHKNNKSW	0.7358

A24	HLA-A*24:08	159	168	VYSSANNCTF	0.8525
A24	HLA-A*24:08	265	275	YYVGYLQPRTF	0.757
A24	HLA-A*24:08	268	277	GYLQPRTFLL	0.6018
A24	HLA-A*24:08	269	277	YLQPRTFLL	0.6645
A24	HLA-A*24:08	312	320	IYQTSNFRV	0.6407
A24	HLA-A*24:08	328	338	RFPNITNLCPF	0.7387
A24	HLA-A*24:08	368	377	LYNSASFSTF	0.7867
A24	HLA-A*24:08	448	456	NYNYLYRLF	0.827
A24	HLA-A*24:08	489	497	YFPLQSYGF	0.8868
A24	HLA-A*24:08	635	643	VYSTGSNVF	0.8901
A24	HLA-A*24:08	1094	1103	VFVSNGTHWF	0.7487
A24	HLA-A*24:08	1208	1216	QYIKWPWYI	0.9046
A24	HLA-A*24:08	1208	1217	QYIKWPWYIW	0.6181
A24	HLA-A*24:10	23	32	QLPPAYTNSF	0.6067
A24	HLA-A*24:10	36	43	VYYPDKVF	0.8127
A24	HLA-A*24:10	37	48	YYPDKVFRSSVL	0.7336
A24	HLA-A*24:10	57	65	PFFSNVTWF	0.8735
A24	HLA-A*24:10	77	86	KRFDNPVLPF	0.571
A24	HLA-A*24:10	78	86	RFDNPVLPF	0.9417
A24	HLA-A*24:10	143	152	VYYHKNNKSW	0.6813
A24	HLA-A*24:10	144	152	YYHKNNKSW	0.898
A24	HLA-A*24:10	151	159	SWMESEFRV	0.7017
A24	HLA-A*24:10	159	168	VYSSANNCTF	0.9516
A24	HLA-A*24:10	167	175	TFEYVSQPF	0.6744
A24	HLA-A*24:10	168	177	FEYVSQPFLM	0.5891
A24	HLA-A*24:10	169	177	EYVSQPFLM	0.8711
A24	HLA-A*24:10	193	201	VFKNIDGYF	0.8319
A24	HLA-A*24:10	203	212	IYSKHTPINL	0.7032
A24	HLA-A*24:10	247	258	SYLTPGDSSSGW	0.6388
A24	HLA-A*24:10	264	275	AYYVGYLQPRTF	0.8043
A24	HLA-A*24:10	265	275	YYVGYLQPRTF	0.8996
A24	HLA-A*24:10	265	276	YYVGYLQPRTFL	0.5933
A24	HLA-A*24:10	267	276	VGYLQPRTFL	0.6401
A24	HLA-A*24:10	268	275	GYLQPRTF	0.6374
A24	HLA-A*24:10	268	276	GYLQPRTFL	0.8962
A24	HLA-A*24:10	268	277	GYLQPRTFLL	0.8395
A24	HLA-A*24:10	269	277	YLQPRTFLL	0.5904
A24	HLA-A*24:10	312	320	IYQTSNFRV	0.8099
A24	HLA-A*24:10	328	338	RFPNITNLCPF	0.8972
A24	HLA-A*24:10	346	353	RFASVYAW	0.5964
A24	HLA-A*24:10	368	377	LYNSASFSTF	0.9153
A24	HLA-A*24:10	395	402	VYADSFVI	0.6432
A24	HLA-A*24:10	448	456	NYNYLYRLF	0.9031
A24	HLA-A*24:10	488	497	CYFPLQSYGF	0.6933
A24	HLA-A*24:10	489	497	YFPLQSYGF	0.9707
A24	HLA-A*24:10	504	512	GYQPYRVVV	0.6833
A24	HLA-A*24:10	504	513	GYQPYRVVVL	0.6625
A24	HLA-A*24:10	504	515	GYQPYRVVVLSF	0.7723
A24	HLA-A*24:10	507	515	PYRVVVLSF	0.7928
A24	HLA-A*24:10	558	565	KFLPFQQF	0.7572

A24	HLA-A*24:10	634	643	RVYSTGSNVF	0.7497
A24	HLA-A*24:10	635	643	VYSTGSNVF	0.9761
A24	HLA-A*24:10	659	670	SYECDIPIGAGI	0.5966
A24	HLA-A*24:10	706	714	AYSNNSIAI	0.8424
A24	HLA-A*24:10	755	763	QYGSFCTQL	0.7571
A24	HLA-A*24:10	788	797	IYKTPPIKDF	0.8924
A24	HLA-A*24:10	816	823	SFIEDLLF	0.8218
A24	HLA-A*24:10	880	888	GTITSGWTF	0.6067
A24	HLA-A*24:10	1051	1062	SFPQSAPHGVVF	0.8219
A24	HLA-A*24:10	1066	1075	TYVPAQEKNF	0.9499
A24	HLA-A*24:10	1094	1102	VFVSNGTHW	0.8862
A24	HLA-A*24:10	1094	1103	VFVSNGTHWF	0.8275
A24	HLA-A*24:10	1101	1109	HWFVTQRNF	0.8486
A24	HLA-A*24:10	1137	1145	VYDPLQPEL	0.9463
A24	HLA-A*24:10	1137	1148	VYDPLQPELDSF	0.8552
A24	HLA-A*24:10	1147	1156	SFKEELDKYF	0.621
A24	HLA-A*24:10	1208	1216	QYIKWPWYI	0.9619
A24	HLA-A*24:10	1208	1217	QYIKWPWYIW	0.6962
A24	HLA-A*24:10	1211	1220	KWPWYIWLGF	0.6791
A24	HLA-A*24:10	1216	1224	IWLGFIAGL	0.748
A24	HLA-A*24:13	57	65	PFFSNVTWF	0.9141
A24	HLA-A*24:13	78	86	RFDNPVLPF	0.8396
A24	HLA-A*24:13	143	152	VYYHKNNKSW	0.7142
A24	HLA-A*24:13	144	152	YYHKNNKSW	0.8036
A24	HLA-A*24:13	159	168	VYSSANNCTF	0.917
A24	HLA-A*24:13	169	177	EYVSQPFLM	0.8499
A24	HLA-A*24:13	193	201	VFKNIDGYF	0.7818
A24	HLA-A*24:13	203	212	IYSKHTPINL	0.6028
A24	HLA-A*24:13	264	275	AYYVGYLQPRTF	0.7912
A24	HLA-A*24:13	265	275	YYVGYLQPRTF	0.879
A24	HLA-A*24:13	268	277	GYLQPRTFLL	0.7046
A24	HLA-A*24:13	269	277	YLQPRTFLL	0.5573
A24	HLA-A*24:13	312	320	IYQTSNFRV	0.6966
A24	HLA-A*24:13	328	338	RFPNITNLCPF	0.8158
A24	HLA-A*24:13	368	377	LYNSASFSTF	0.8553
A24	HLA-A*24:13	395	402	VYADSFVI	0.5149
A24	HLA-A*24:13	448	456	NYNYLYRLF	0.8881
A24	HLA-A*24:13	488	497	CYFPLQSYGF	0.6136
A24	HLA-A*24:13	489	497	YFPLQSYGF	0.9108
A24	HLA-A*24:13	504	515	GYQPYRVVVLSF	0.667
A24	HLA-A*24:13	507	515	PYRVVVLSF	0.8298
A24	HLA-A*24:13	558	565	KFLPFQQF	0.7881
A24	HLA-A*24:13	634	643	RVYSTGSNVF	0.5782
A24	HLA-A*24:13	635	643	VYSTGSNVF	0.934
A24	HLA-A*24:13	788	797	IYKTPPIKDF	0.9066
A24	HLA-A*24:13	816	823	SFIEDLLF	0.7909
A24	HLA-A*24:13	880	888	GTITSGWTF	0.5317
A24	HLA-A*24:13	898	906	FAMQMAYRF	0.5028
A24	HLA-A*24:13	1066	1075	TYVPAQEKNF	0.9478
A24	HLA-A*24:13	1094	1102	VFVSNGTHW	0.8761

A24	HLA-A*24:13	1094	1103	VFVSNGTHWF	0.8258
A24	HLA-A*24:13	1101	1109	HWFVTQRNF	0.7845
A24	HLA-A*24:13	1137	1148	VYDPLQPELDSF	0.7515
A24	HLA-A*24:13	1208	1216	QYIKWPWYI	0.9638
A24	HLA-A*24:13	1208	1217	QYIKWPWYIW	0.7814
A24	HLA-A*24:13	1211	1220	KWPWYIWLGF	0.6037
A24	HLA-A*24:13	1216	1224	IWLGFIAGL	0.5925
A24	HLA-A*24:18	36	43	VYYPDKVF	0.8173
A24	HLA-A*24:18	57	65	PFFSNVTWF	0.7076
A24	HLA-A*24:18	77	86	KRFDNPVLPF	0.5982
A24	HLA-A*24:18	78	86	RFDNPVLPF	0.8452
A24	HLA-A*24:18	143	152	VYYHKNNKSW	0.6438
A24	HLA-A*24:18	144	152	YYHKNNKSW	0.7986
A24	HLA-A*24:18	159	168	VYSSANNCTF	0.8212
A24	HLA-A*24:18	169	177	EYVSQPFLM	0.6396
A24	HLA-A*24:18	193	201	VFKNIDGYF	0.8479
A24	HLA-A*24:18	203	212	IYSKHTPINL	0.5908
A24	HLA-A*24:18	264	275	AYYVGYLQPRTF	0.7906
A24	HLA-A*24:18	265	275	YYVGYLQPRTF	0.7951
A24	HLA-A*24:18	267	276	VGYLQPRTFL	0.5749
A24	HLA-A*24:18	268	275	GYLQPRTF	0.7049
A24	HLA-A*24:18	268	276	GYLQPRTFL	0.8826
A24	HLA-A*24:18	268	277	GYLQPRTFLL	0.7029
A24	HLA-A*24:18	312	320	IYQTSNFRV	0.625
A24	HLA-A*24:18	328	338	RFPNITNLCPF	0.6955
A24	HLA-A*24:18	350	358	VYAWNRKRI	0.5642
A24	HLA-A*24:18	368	377	LYNSASFSTF	0.7395
A24	HLA-A*24:18	448	456	NYNYLYRLF	0.8086
A24	HLA-A*24:18	488	497	CYFPLQSYGF	0.5236
A24	HLA-A*24:18	489	497	YFPLQSYGF	0.8335
A24	HLA-A*24:18	504	512	GYQPYRVVV	0.659
A24	HLA-A*24:18	504	513	GYQPYRVVVL	0.5063
A24	HLA-A*24:18	504	515	GYQPYRVVVLSF	0.6043
A24	HLA-A*24:18	507	515	PYRVVVLSF	0.7472
A24	HLA-A*24:18	558	565	KFLPFQQF	0.7919
A24	HLA-A*24:18	634	643	RVYSTGSNVF	0.6968
A24	HLA-A*24:18	635	643	VYSTGSNVF	0.9155
A24	HLA-A*24:18	706	714	AYSNNSIAI	0.6343
A24	HLA-A*24:18	755	763	QYGSFCTQL	0.5402
A24	HLA-A*24:18	788	797	IYKTPPIKDF	0.8671
A24	HLA-A*24:18	1066	1075	TYVPAQEKNF	0.7987
A24	HLA-A*24:18	1094	1102	VFVSNGTHW	0.7787
A24	HLA-A*24:18	1094	1103	VFVSNGTHWF	0.6654
A24	HLA-A*24:18	1101	1109	HWFVTQRNF	0.8544
A24	HLA-A*24:18	1137	1145	VYDPLQPEL	0.8216
A24	HLA-A*24:18	1208	1216	QYIKWPWYI	0.8782
A24	HLA-A*24:18	1211	1220	KWPWYIWLGF	0.5123
A24	HLA-A*24:18	1216	1224	IWLGFIAGL	0.7231
A24	HLA-A*24:20	37	48	YYPDKVFRSSVL	0.601
A24	HLA-A*24:20	57	65	PFFSNVTWF	0.8728

A24	HLA-A*24:20	78	86	RFDNPVLPF	0.8538
A24	HLA-A*24:20	143	152	VYYHKNNKSW	0.7378
A24	HLA-A*24:20	144	152	YYHKNNKSW	0.8416
A24	HLA-A*24:20	151	159	SWMESEFRV	0.5046
A24	HLA-A*24:20	159	168	VYSSANNCTF	0.9582
A24	HLA-A*24:20	167	175	TFEYVSQPF	0.5365
A24	HLA-A*24:20	169	177	EYVSQPFLM	0.8484
A24	HLA-A*24:20	203	212	IYSKHTPINL	0.7085
A24	HLA-A*24:20	264	275	AYYVGYLQPRTF	0.8037
A24	HLA-A*24:20	265	275	YYVGYLQPRTF	0.9005
A24	HLA-A*24:20	268	277	GYLQPRTFLL	0.731
A24	HLA-A*24:20	269	277	YLQPRTFLL	0.5359
A24	HLA-A*24:20	312	320	IYQTSNFRV	0.785
A24	HLA-A*24:20	328	338	RFPNITNLCPF	0.8789
A24	HLA-A*24:20	346	353	RFASVYAW	0.521
A24	HLA-A*24:20	368	377	LYNSASFSTF	0.914
A24	HLA-A*24:20	395	402	VYADSFVI	0.6394
A24	HLA-A*24:20	448	456	NYNYLYRLF	0.9353
A24	HLA-A*24:20	488	497	CYFPLQSYGF	0.5885
A24	HLA-A*24:20	489	497	YFPLQSYGF	0.9442
A24	HLA-A*24:20	504	515	GYQPYRVVVLSF	0.6707
A24	HLA-A*24:20	507	515	PYRVVVLSF	0.8166
A24	HLA-A*24:20	558	565	KFLPFQQF	0.6948
A24	HLA-A*24:20	634	643	RVYSTGSNVF	0.7146
A24	HLA-A*24:20	635	643	VYSTGSNVF	0.9696
A24	HLA-A*24:20	755	763	QYGSFCTQL	0.5614
A24	HLA-A*24:20	788	797	IYKTPPIKDF	0.9226
A24	HLA-A*24:20	816	823	SFIEDLLF	0.7253
A24	HLA-A*24:20	1066	1075	TYVPAQEKNF	0.9479
A24	HLA-A*24:20	1094	1102	VFVSNGTHW	0.8658
A24	HLA-A*24:20	1094	1103	VFVSNGTHWF	0.8335
A24	HLA-A*24:20	1101	1109	HWFVTQRNF	0.754
A24	HLA-A*24:20	1137	1148	VYDPLQPELDSF	0.8174
A24	HLA-A*24:20	1208	1216	QYIKWPWYI	0.9613
A24	HLA-A*24:20	1208	1217	QYIKWPWYIW	0.7582
A24	HLA-A*24:20	1211	1220	KWPWYIWLGF	0.6582
A24	HLA-A*24:21	37	48	YYPDKVFRSSVL	0.601
A24	HLA-A*24:21	57	65	PFFSNVTWF	0.8728
A24	HLA-A*24:21	78	86	RFDNPVLPF	0.8538
A24	HLA-A*24:21	143	152	VYYHKNNKSW	0.7378
A24	HLA-A*24:21	144	152	YYHKNNKSW	0.8416
A24	HLA-A*24:21	151	159	SWMESEFRV	0.5046
A24	HLA-A*24:21	159	168	VYSSANNCTF	0.9582
A24	HLA-A*24:21	167	175	TFEYVSQPF	0.5365
A24	HLA-A*24:21	169	177	EYVSQPFLM	0.8484
A24	HLA-A*24:21	203	212	IYSKHTPINL	0.7085
A24	HLA-A*24:21	264	275	AYYVGYLQPRTF	0.8037
A24	HLA-A*24:21	265	275	YYVGYLQPRTF	0.9005
A24	HLA-A*24:21	268	277	GYLQPRTFLL	0.731
A24	HLA-A*24:21	269	277	YLQPRTFLL	0.5359

A24	HLA-A*24:21	312	320	IYQTSNFRV	0.785
A24	HLA-A*24:21	328	338	RFPNITNLCPF	0.8789
A24	HLA-A*24:21	346	353	RFASVYAW	0.521
A24	HLA-A*24:21	368	377	LYNSASFSTF	0.914
A24	HLA-A*24:21	395	402	VYADSFVI	0.6394
A24	HLA-A*24:21	448	456	NYNYLYRLF	0.9353
A24	HLA-A*24:21	488	497	CYFPLQSYGF	0.5885
A24	HLA-A*24:21	489	497	YFPLQSYGF	0.9442
A24	HLA-A*24:21	504	515	GYQPYRVVVLSF	0.6707
A24	HLA-A*24:21	507	515	PYRVVVLSF	0.8166
A24	HLA-A*24:21	558	565	KFLPFQQF	0.6948
A24	HLA-A*24:21	634	643	RVYSTGSNVF	0.7146
A24	HLA-A*24:21	635	643	VYSTGSNVF	0.9696
A24	HLA-A*24:21	755	763	QYGSFCTQL	0.5614
A24	HLA-A*24:21	788	797	IYKTPPIKDF	0.9226
A24	HLA-A*24:21	816	823	SFIEDLLF	0.7253
A24	HLA-A*24:21	1066	1075	TYVPAQEKNF	0.9479
A24	HLA-A*24:21	1094	1102	VFVSNGTHW	0.8658
A24	HLA-A*24:21	1094	1103	VFVSNGTHWF	0.8335
A24	HLA-A*24:21	1101	1109	HWFVTQRNF	0.754
A24	HLA-A*24:21	1137	1148	VYDPLQPELDSF	0.8174
A24	HLA-A*24:21	1208	1216	QYIKWPWYI	0.9613
A24	HLA-A*24:21	1208	1217	QYIKWPWYIW	0.7582
A24	HLA-A*24:21	1211	1220	KWPWYIWLGF	0.6582
A24	HLA-A*24:22	23	32	QLPPAYTNSF	0.6934
A24	HLA-A*24:22	36	43	VYYPDKVF	0.8478
A24	HLA-A*24:22	37	48	YYPDKVFRSSVL	0.692
A24	HLA-A*24:22	47	55	VLHSTQDLF	0.6614
A24	HLA-A*24:22	57	65	PFFSNVTWF	0.8749
A24	HLA-A*24:22	77	86	KRFDNPVLPF	0.6655
A24	HLA-A*24:22	78	86	RFDNPVLPF	0.861
A24	HLA-A*24:22	143	152	VYYHKNNKSW	0.8066
A24	HLA-A*24:22	144	152	YYHKNNKSW	0.9429
A24	HLA-A*24:22	151	159	SWMESEFRV	0.6473
A24	HLA-A*24:22	159	168	VYSSANNCTF	0.9584
A24	HLA-A*24:22	167	175	TFEYVSQPF	0.6382
A24	HLA-A*24:22	169	177	EYVSQPFLM	0.8978
A24	HLA-A*24:22	193	201	VFKNIDGYF	0.9224
A24	HLA-A*24:22	203	212	IYSKHTPINL	0.7532
A24	HLA-A*24:22	247	258	SYLTPGDSSSGW	0.7072
A24	HLA-A*24:22	264	275	AYYVGYLQPRTF	0.8149
A24	HLA-A*24:22	265	275	YYVGYLQPRTF	0.9218
A24	HLA-A*24:22	268	276	GYLQPRTFL	0.8871
A24	HLA-A*24:22	268	277	GYLQPRTFLL	0.7943
A24	HLA-A*24:22	269	277	YLQPRTFLL	0.6428
A24	HLA-A*24:22	312	320	IYQTSNFRV	0.7597
A24	HLA-A*24:22	328	338	RFPNITNLCPF	0.8917
A24	HLA-A*24:22	346	353	RFASVYAW	0.6744
A24	HLA-A*24:22	350	358	VYAWNRKRI	0.6447
A24	HLA-A*24:22	368	377	LYNSASFSTF	0.9108

A24	HLA-A*24:22	369	377	YNSASFSTF	0.6618
A24	HLA-A*24:22	379	387	CYGVSPTKL	0.6159
A24	HLA-A*24:22	448	456	NYNYLYRLF	0.9199
A24	HLA-A*24:22	488	497	CYFPLQSYGF	0.766
A24	HLA-A*24:22	489	497	YFPLQSYGF	0.9702
A24	HLA-A*24:22	504	512	GYQPYRVVV	0.6915
A24	HLA-A*24:22	504	513	GYQPYRVVVL	0.6647
A24	HLA-A*24:22	504	515	GYQPYRVVVLSF	0.7575
A24	HLA-A*24:22	507	515	PYRVVVLSF	0.8211
A24	HLA-A*24:22	558	565	KFLPFQQF	0.7981
A24	HLA-A*24:22	634	643	RVYSTGSNVF	0.8412
A24	HLA-A*24:22	635	643	VYSTGSNVF	0.9766
A24	HLA-A*24:22	659	670	SYECDIPIGAGI	0.6229
A24	HLA-A*24:22	706	714	AYSNNSIAI	0.7925
A24	HLA-A*24:22	755	763	QYGSFCTQL	0.7423
A24	HLA-A*24:22	788	797	IYKTPPIKDF	0.9284
A24	HLA-A*24:22	816	823	SFIEDLLF	0.7943
A24	HLA-A*24:22	880	888	GTITSGWTF	0.6649
A24	HLA-A*24:22	1051	1062	SFPQSAPHGVVF	0.8172
A24	HLA-A*24:22	1066	1075	TYVPAQEKNF	0.9704
A24	HLA-A*24:22	1094	1102	VFVSNGTHW	0.9217
A24	HLA-A*24:22	1094	1103	VFVSNGTHWF	0.8558
A24	HLA-A*24:22	1095	1103	FVSNGTHWF	0.6853
A24	HLA-A*24:22	1101	1109	HWFVTQRNF	0.884
A24	HLA-A*24:22	1137	1145	VYDPLQPEL	0.9126
A24	HLA-A*24:22	1137	1148	VYDPLQPELDSF	0.8166
A24	HLA-A*24:22	1147	1156	SFKEELDKYF	0.7313
A24	HLA-A*24:22	1208	1216	QYIKWPWYI	0.9408
A24	HLA-A*24:22	1208	1217	QYIKWPWYIW	0.7516
A24	HLA-A*24:22	1211	1220	KWPWYIWLGF	0.6645
A24	HLA-A*24:22	1216	1224	IWLGFIAGL	0.7116
A24	HLA-A*24:23	36	43	VYYPDKVF	0.8969
A24	HLA-A*24:23	37	48	YYPDKVFRSSVL	0.7149
A24	HLA-A*24:23	57	65	PFFSNVTWF	0.8822
A24	HLA-A*24:23	77	86	KRFDNPVLPF	0.6134
A24	HLA-A*24:23	78	86	RFDNPVLPF	0.9523
A24	HLA-A*24:23	143	152	VYYHKNNKSW	0.8351
A24	HLA-A*24:23	144	152	YYHKNNKSW	0.9523
A24	HLA-A*24:23	151	159	SWMESEFRV	0.7048
A24	HLA-A*24:23	159	168	VYSSANNCTF	0.9732
A24	HLA-A*24:23	167	175	TFEYVSQPF	0.6889
A24	HLA-A*24:23	169	177	EYVSQPFLM	0.8798
A24	HLA-A*24:23	193	201	VFKNIDGYF	0.8984
A24	HLA-A*24:23	203	212	IYSKHTPINL	0.8029
A24	HLA-A*24:23	247	258	SYLTPGDSSSGW	0.7197
A24	HLA-A*24:23	264	275	AYYVGYLQPRTF	0.8966
A24	HLA-A*24:23	265	275	YYVGYLQPRTF	0.9506
A24	HLA-A*24:23	265	276	YYVGYLQPRTFL	0.6242
A24	HLA-A*24:23	267	276	VGYLQPRTFL	0.7184
A24	HLA-A*24:23	268	275	GYLQPRTF	0.7874

A24	HLA-A*24:23	268	276	GYLQPRTFL	0.9337
A24	HLA-A*24:23	268	277	GYLQPRTFLL	0.8923
A24	HLA-A*24:23	312	320	IYQTSNFRV	0.8841
A24	HLA-A*24:23	328	338	RFPNITNLCPF	0.9389
A24	HLA-A*24:23	346	353	RFASVYAW	0.7335
A24	HLA-A*24:23	350	358	VYAWNRKRI	0.6686
A24	HLA-A*24:23	368	377	LYNSASFSTF	0.9453
A24	HLA-A*24:23	379	387	CYGVSPTKL	0.6202
A24	HLA-A*24:23	395	402	VYADSFVI	0.6956
A24	HLA-A*24:23	448	456	NYNYLYRLF	0.9379
A24	HLA-A*24:23	488	497	CYFPLQSYGF	0.7404
A24	HLA-A*24:23	489	497	YFPLQSYGF	0.9778
A24	HLA-A*24:23	504	512	GYQPYRVVV	0.7982
A24	HLA-A*24:23	504	513	GYQPYRVVVL	0.7444
A24	HLA-A*24:23	504	515	GYQPYRVVVLSF	0.8581
A24	HLA-A*24:23	507	515	PYRVVVLSF	0.8533
A24	HLA-A*24:23	558	565	KFLPFQQF	0.8636
A24	HLA-A*24:23	632	643	TWRVYSTGSNVF	0.5957
A24	HLA-A*24:23	634	643	RVYSTGSNVF	0.8822
A24	HLA-A*24:23	635	643	VYSTGSNVF	0.9872
A24	HLA-A*24:23	659	670	SYECDIPIGAGI	0.6187
A24	HLA-A*24:23	706	714	AYSNNSIAI	0.8828
A24	HLA-A*24:23	755	763	QYGSFCTQL	0.7929
A24	HLA-A*24:23	788	797	IYKTPPIKDF	0.9538
A24	HLA-A*24:23	816	823	SFIEDLLF	0.8244
A24	HLA-A*24:23	1051	1062	SFPQSAPHGVVF	0.8397
A24	HLA-A*24:23	1066	1075	TYVPAQEKNF	0.97
A24	HLA-A*24:23	1094	1102	VFVSNGTHW	0.9343
A24	HLA-A*24:23	1094	1103	VFVSNGTHWF	0.8723
A24	HLA-A*24:23	1101	1109	HWFVTQRNF	0.9047
A24	HLA-A*24:23	1137	1145	VYDPLQPEL	0.9496
A24	HLA-A*24:23	1137	1148	VYDPLQPELDSF	0.8822
A24	HLA-A*24:23	1147	1156	SFKEELDKYF	0.6438
A24	HLA-A*24:23	1205	1212	KYEQYIKW	0.6328
A24	HLA-A*24:23	1208	1216	QYIKWPWYI	0.9753
A24	HLA-A*24:23	1208	1217	QYIKWPWYIW	0.7703
A24	HLA-A*24:23	1211	1220	KWPWYIWLGF	0.7747
A24	HLA-A*24:23	1216	1224	IWLGFIAGL	0.7773
A24	HLA-A*24:26	37	48	YYPDKVFRSSVL	0.601
A24	HLA-A*24:26	57	65	PFFSNVTWF	0.8728
A24	HLA-A*24:26	78	86	RFDNPVLPF	0.8538
A24	HLA-A*24:26	143	152	VYYHKNNKSW	0.7378
A24	HLA-A*24:26	144	152	YYHKNNKSW	0.8416
A24	HLA-A*24:26	151	159	SWMESEFRV	0.5046
A24	HLA-A*24:26	159	168	VYSSANNCTF	0.9582
A24	HLA-A*24:26	167	175	TFEYVSQPF	0.5365
A24	HLA-A*24:26	169	177	EYVSQPFLM	0.8484
A24	HLA-A*24:26	203	212	IYSKHTPINL	0.7085
A24	HLA-A*24:26	264	275	AYYVGYLQPRTF	0.8037
A24	HLA-A*24:26	265	275	YYVGYLQPRTF	0.9005

A24	HLA-A*24:26	268	277	GYLQPRTFLL	0.731
A24	HLA-A*24:26	269	277	YLQPRTFLL	0.5359
A24	HLA-A*24:26	312	320	IYQTSNFRV	0.785
A24	HLA-A*24:26	328	338	RFPNITNLCPF	0.8789
A24	HLA-A*24:26	346	353	RFASVYAW	0.521
A24	HLA-A*24:26	368	377	LYNSASFSTF	0.914
A24	HLA-A*24:26	395	402	VYADSFVI	0.6394
A24	HLA-A*24:26	448	456	NYNYLYRLF	0.9353
A24	HLA-A*24:26	488	497	CYFPLQSYGF	0.5885
A24	HLA-A*24:26	489	497	YFPLQSYGF	0.9442
A24	HLA-A*24:26	504	515	GYQPYRVVVLSF	0.6707
A24	HLA-A*24:26	507	515	PYRVVVLSF	0.8166
A24	HLA-A*24:26	558	565	KFLPFQQF	0.6948
A24	HLA-A*24:26	634	643	RVYSTGSNVF	0.7146
A24	HLA-A*24:26	635	643	VYSTGSNVF	0.9696
A24	HLA-A*24:26	755	763	QYGSFCTQL	0.5614
A24	HLA-A*24:26	788	797	IYKTPPIKDF	0.9226
A24	HLA-A*24:26	816	823	SFIEDLLF	0.7253
A24	HLA-A*24:26	1066	1075	TYVPAQEKNF	0.9479
A24	HLA-A*24:26	1094	1102	VFVSNGTHW	0.8658
A24	HLA-A*24:26	1094	1103	VFVSNGTHWF	0.8335
A24	HLA-A*24:26	1101	1109	HWFVTQRNF	0.754
A24	HLA-A*24:26	1137	1148	VYDPLQPELDSF	0.8174
A24	HLA-A*24:26	1208	1216	QYIKWPWYI	0.9613
A24	HLA-A*24:26	1208	1217	QYIKWPWYIW	0.7582
A24	HLA-A*24:26	1211	1220	KWPWYIWLGF	0.6582
A24	HLA-A*24:27	37	48	YYPDKVFRSSVL	0.601
A24	HLA-A*24:27	57	65	PFFSNVTWF	0.8728
A24	HLA-A*24:27	78	86	RFDNPVLPF	0.8538
A24	HLA-A*24:27	143	152	VYYHKNNKSW	0.7378
A24	HLA-A*24:27	144	152	YYHKNNKSW	0.8416
A24	HLA-A*24:27	151	159	SWMESEFRV	0.5046
A24	HLA-A*24:27	159	168	VYSSANNCTF	0.9582
A24	HLA-A*24:27	167	175	TFEYVSQPF	0.5365
A24	HLA-A*24:27	169	177	EYVSQPFLM	0.8484
A24	HLA-A*24:27	203	212	IYSKHTPINL	0.7085
A24	HLA-A*24:27	264	275	AYYVGYLQPRTF	0.8037
A24	HLA-A*24:27	265	275	YYVGYLQPRTF	0.9005
A24	HLA-A*24:27	268	277	GYLQPRTFLL	0.731
A24	HLA-A*24:27	269	277	YLQPRTFLL	0.5359
A24	HLA-A*24:27	312	320	IYQTSNFRV	0.785
A24	HLA-A*24:27	328	338	RFPNITNLCPF	0.8789
A24	HLA-A*24:27	346	353	RFASVYAW	0.521
A24	HLA-A*24:27	368	377	LYNSASFSTF	0.914
A24	HLA-A*24:27	395	402	VYADSFVI	0.6394
A24	HLA-A*24:27	448	456	NYNYLYRLF	0.9353
A24	HLA-A*24:27	488	497	CYFPLQSYGF	0.5885
A24	HLA-A*24:27	489	497	YFPLQSYGF	0.9442
A24	HLA-A*24:27	504	515	GYQPYRVVVLSF	0.6707
A24	HLA-A*24:27	507	515	PYRVVVLSF	0.8166

A24	HLA-A*24:27	558	565	KFLPFQQF	0.6948
A24	HLA-A*24:27	634	643	RVYSTGSNVF	0.7146
A24	HLA-A*24:27	635	643	VYSTGSNVF	0.9696
A24	HLA-A*24:27	755	763	QYGSFCTQL	0.5614
A24	HLA-A*24:27	788	797	IYKTPPIKDF	0.9226
A24	HLA-A*24:27	816	823	SFIEDLLF	0.7253
A24	HLA-A*24:27	1066	1075	TYVPAQEKNF	0.9479
A24	HLA-A*24:27	1094	1102	VFVSNGTHW	0.8658
A24	HLA-A*24:27	1094	1103	VFVSNGTHWF	0.8335
A24	HLA-A*24:27	1101	1109	HWFVTQRNF	0.754
A24	HLA-A*24:27	1137	1148	VYDPLQPELDSF	0.8174
A24	HLA-A*24:27	1208	1216	QYIKWPWYI	0.9613
A24	HLA-A*24:27	1208	1217	QYIKWPWYIW	0.7582
A24	HLA-A*24:27	1211	1220	KWPWYIWLGF	0.6582
A24	HLA-A*24:28	151	159	SWMESEFRV	0.6568
A24	HLA-A*24:28	269	277	YLQPRTFLL	0.5702
A24	HLA-A*24:28	312	320	IYQTSNFRV	0.5721
A24	HLA-A*24:28	1208	1216	QYIKWPWYI	0.7279
A24	HLA-A*24:28	1216	1224	IWLGFIAGL	0.697
A24	HLA-A*24:29	37	48	YYPDKVFRSSVL	0.601
A24	HLA-A*24:29	57	65	PFFSNVTWF	0.8728
A24	HLA-A*24:29	78	86	RFDNPVLPF	0.8538
A24	HLA-A*24:29	143	152	VYYHKNNKSW	0.7378
A24	HLA-A*24:29	144	152	YYHKNNKSW	0.8416
A24	HLA-A*24:29	151	159	SWMESEFRV	0.5046
A24	HLA-A*24:29	159	168	VYSSANNCTF	0.9582
A24	HLA-A*24:29	167	175	TFEYVSQPF	0.5365
A24	HLA-A*24:29	169	177	EYVSQPFLM	0.8484
A24	HLA-A*24:29	203	212	IYSKHTPINL	0.7085
A24	HLA-A*24:29	264	275	AYYVGYLQPRTF	0.8037
A24	HLA-A*24:29	265	275	YYVGYLQPRTF	0.9005
A24	HLA-A*24:29	268	277	GYLQPRTFLL	0.731
A24	HLA-A*24:29	269	277	YLQPRTFLL	0.5359
A24	HLA-A*24:29	312	320	IYQTSNFRV	0.785
A24	HLA-A*24:29	328	338	RFPNITNLCPF	0.8789
A24	HLA-A*24:29	346	353	RFASVYAW	0.521
A24	HLA-A*24:29	368	377	LYNSASFSTF	0.914
A24	HLA-A*24:29	395	402	VYADSFVI	0.6394
A24	HLA-A*24:29	448	456	NYNYLYRLF	0.9353
A24	HLA-A*24:29	488	497	CYFPLQSYGF	0.5885
A24	HLA-A*24:29	489	497	YFPLQSYGF	0.9442
A24	HLA-A*24:29	504	515	GYQPYRVVVLSF	0.6707
A24	HLA-A*24:29	507	515	PYRVVVLSF	0.8166
A24	HLA-A*24:29	558	565	KFLPFQQF	0.6948
A24	HLA-A*24:29	634	643	RVYSTGSNVF	0.7146
A24	HLA-A*24:29	635	643	VYSTGSNVF	0.9696
A24	HLA-A*24:29	755	763	QYGSFCTQL	0.5614
A24	HLA-A*24:29	788	797	IYKTPPIKDF	0.9226
A24	HLA-A*24:29	816	823	SFIEDLLF	0.7253
A24	HLA-A*24:29	1066	1075	TYVPAQEKNF	0.9479

A24	HLA-A*24:29	1094	1102	VFVSNGTHW	0.8658
A24	HLA-A*24:29	1094	1103	VFVSNGTHWF	0.8335
A24	HLA-A*24:29	1101	1109	HWFVTQRNF	0.754
A24	HLA-A*24:29	1137	1148	VYDPLQPELDSF	0.8174
A24	HLA-A*24:29	1208	1216	QYIKWPWYI	0.9613
A24	HLA-A*24:29	1208	1217	QYIKWPWYIW	0.7582
A24	HLA-A*24:29	1211	1220	KWPWYIWLGF	0.6582
A24	HLA-A*24:33	36	43	VYYPDKVF	0.8969
A24	HLA-A*24:33	37	48	YYPDKVFRSSVL	0.7149
A24	HLA-A*24:33	57	65	PFFSNVTWF	0.8822
A24	HLA-A*24:33	77	86	KRFDNPVLPF	0.6134
A24	HLA-A*24:33	78	86	RFDNPVLPF	0.9523
A24	HLA-A*24:33	143	152	VYYHKNNKSW	0.8351
A24	HLA-A*24:33	144	152	YYHKNNKSW	0.9523
A24	HLA-A*24:33	151	159	SWMESEFRV	0.7048
A24	HLA-A*24:33	159	168	VYSSANNCTF	0.9732
A24	HLA-A*24:33	167	175	TFEYVSQPF	0.6889
A24	HLA-A*24:33	169	177	EYVSQPFLM	0.8798
A24	HLA-A*24:33	193	201	VFKNIDGYF	0.8984
A24	HLA-A*24:33	203	212	IYSKHTPINL	0.8029
A24	HLA-A*24:33	247	258	SYLTPGDSSSGW	0.7197
A24	HLA-A*24:33	264	275	AYYVGYLQPRTF	0.8966
A24	HLA-A*24:33	265	275	YYVGYLQPRTF	0.9506
A24	HLA-A*24:33	265	276	YYVGYLQPRTFL	0.6242
A24	HLA-A*24:33	267	276	VGYLQPRTFL	0.7184
A24	HLA-A*24:33	268	275	GYLQPRTF	0.7874
A24	HLA-A*24:33	268	276	GYLQPRTFL	0.9337
A24	HLA-A*24:33	268	277	GYLQPRTFLL	0.8923
A24	HLA-A*24:33	312	320	IYQTSNFRV	0.8841
A24	HLA-A*24:33	328	338	RFPNITNLCPF	0.9389
A24	HLA-A*24:33	346	353	RFASVYAW	0.7335
A24	HLA-A*24:33	350	358	VYAWNRKRI	0.6686
A24	HLA-A*24:33	368	377	LYNSASFSTF	0.9453
A24	HLA-A*24:33	379	387	CYGVSPTKL	0.6202
A24	HLA-A*24:33	395	402	VYADSFVI	0.6956
A24	HLA-A*24:33	448	456	NYNYLYRLF	0.9379
A24	HLA-A*24:33	488	497	CYFPLQSYGF	0.7404
A24	HLA-A*24:33	489	497	YFPLQSYGF	0.9778
A24	HLA-A*24:33	504	512	GYQPYRVVV	0.7982
A24	HLA-A*24:33	504	513	GYQPYRVVVL	0.7444
A24	HLA-A*24:33	504	515	GYQPYRVVVLSF	0.8581
A24	HLA-A*24:33	507	515	PYRVVVLSF	0.8533
A24	HLA-A*24:33	558	565	KFLPFQQF	0.8636
A24	HLA-A*24:33	632	643	TWRVYSTGSNVF	0.5957
A24	HLA-A*24:33	634	643	RVYSTGSNVF	0.8822
A24	HLA-A*24:33	635	643	VYSTGSNVF	0.9872
A24	HLA-A*24:33	659	670	SYECDIPIGAGI	0.6187
A24	HLA-A*24:33	706	714	AYSNNSIAI	0.8828
A24	HLA-A*24:33	755	763	QYGSFCTQL	0.7929
A24	HLA-A*24:33	788	797	IYKTPPIKDF	0.9538

A24	HLA-A*24:33	816	823	SFIEDLLF	0.8244
A24	HLA-A*24:33	1051	1062	SFPQSAPHGVVF	0.8397
A24	HLA-A*24:33	1066	1075	TYVPAQEKNF	0.97
A24	HLA-A*24:33	1094	1102	VFVSNGTHW	0.9343
A24	HLA-A*24:33	1094	1103	VFVSNGTHWF	0.8723
A24	HLA-A*24:33	1101	1109	HWFVTQRNF	0.9047
A24	HLA-A*24:33	1137	1145	VYDPLQPEL	0.9496
A24	HLA-A*24:33	1137	1148	VYDPLQPELDSF	0.8822
A24	HLA-A*24:33	1147	1156	SFKEELDKYF	0.6438
A24	HLA-A*24:33	1205	1212	KYEQYIKW	0.6328
A24	HLA-A*24:33	1208	1216	QYIKWPWYI	0.9753
A24	HLA-A*24:33	1208	1217	QYIKWPWYIW	0.7703
A24	HLA-A*24:33	1211	1220	KWPWYIWLGF	0.7747
A24	HLA-A*24:33	1216	1224	IWLGFIAGL	0.7773
A24	HLA-A*24:34	37	48	YYPDKVFRSSVL	0.6196
A24	HLA-A*24:34	57	65	PFFSNVTWF	0.8335
A24	HLA-A*24:34	78	86	RFDNPVLPF	0.8388
A24	HLA-A*24:34	143	152	VYYHKNNKSW	0.6844
A24	HLA-A*24:34	144	152	YYHKNNKSW	0.819
A24	HLA-A*24:34	151	159	SWMESEFRV	0.5644
A24	HLA-A*24:34	159	168	VYSSANNCTF	0.9424
A24	HLA-A*24:34	167	175	TFEYVSQPF	0.5518
A24	HLA-A*24:34	169	177	EYVSQPFLM	0.8551
A24	HLA-A*24:34	193	201	VFKNIDGYF	0.7905
A24	HLA-A*24:34	203	212	IYSKHTPINL	0.7288
A24	HLA-A*24:34	264	275	AYYVGYLQPRTF	0.7698
A24	HLA-A*24:34	265	275	YYVGYLQPRTF	0.857
A24	HLA-A*24:34	268	277	GYLQPRTFLL	0.7636
A24	HLA-A*24:34	269	277	YLQPRTFLL	0.6367
A24	HLA-A*24:34	312	320	IYQTSNFRV	0.7925
A24	HLA-A*24:34	328	338	RFPNITNLCPF	0.8538
A24	HLA-A*24:34	350	358	VYAWNRKRI	0.5795
A24	HLA-A*24:34	368	377	LYNSASFSTF	0.9096
A24	HLA-A*24:34	395	402	VYADSFVI	0.6072
A24	HLA-A*24:34	448	456	NYNYLYRLF	0.9345
A24	HLA-A*24:34	488	497	CYFPLQSYGF	0.618
A24	HLA-A*24:34	489	497	YFPLQSYGF	0.9263
A24	HLA-A*24:34	504	515	GYQPYRVVVLSF	0.6666
A24	HLA-A*24:34	507	515	PYRVVVLSF	0.7841
A24	HLA-A*24:34	634	643	RVYSTGSNVF	0.6878
A24	HLA-A*24:34	635	643	VYSTGSNVF	0.9648
A24	HLA-A*24:34	706	714	AYSNNSIAI	0.7097
A24	HLA-A*24:34	755	763	QYGSFCTQL	0.6183
A24	HLA-A*24:34	788	797	IYKTPPIKDF	0.9047
A24	HLA-A*24:34	897	906	PFAMQMAYRF	0.5116
A24	HLA-A*24:34	1066	1075	TYVPAQEKNF	0.9166
A24	HLA-A*24:34	1094	1102	VFVSNGTHW	0.866
A24	HLA-A*24:34	1094	1103	VFVSNGTHWF	0.8212
A24	HLA-A*24:34	1101	1109	HWFVTQRNF	0.7483
A24	HLA-A*24:34	1137	1148	VYDPLQPELDSF	0.7712

A24	HLA-A*24:34	1208	1216	QYIKWPWYI	0.9658
A24	HLA-A*24:34	1208	1217	QYIKWPWYIW	0.7348
A24	HLA-A*24:34	1211	1220	KWPWYIWLGF	0.6265
A24	HLA-A*24:35	37	48	YYPDKVFRSSVL	0.601
A24	HLA-A*24:35	57	65	PFFSNVTWF	0.8728
A24	HLA-A*24:35	78	86	RFDNPVLPF	0.8538
A24	HLA-A*24:35	143	152	VYYHKNNKSW	0.7378
A24	HLA-A*24:35	144	152	YYHKNNKSW	0.8416
A24	HLA-A*24:35	151	159	SWMESEFRV	0.5046
A24	HLA-A*24:35	159	168	VYSSANNCTF	0.9582
A24	HLA-A*24:35	167	175	TFEYVSQPF	0.5365
A24	HLA-A*24:35	169	177	EYVSQPFLM	0.8484
A24	HLA-A*24:35	203	212	IYSKHTPINL	0.7085
A24	HLA-A*24:35	264	275	AYYVGYLQPRTF	0.8037
A24	HLA-A*24:35	265	275	YYVGYLQPRTF	0.9005
A24	HLA-A*24:35	268	277	GYLQPRTFLL	0.731
A24	HLA-A*24:35	269	277	YLQPRTFLL	0.5359
A24	HLA-A*24:35	312	320	IYQTSNFRV	0.785
A24	HLA-A*24:35	328	338	RFPNITNLCPF	0.8789
A24	HLA-A*24:35	346	353	RFASVYAW	0.521
A24	HLA-A*24:35	368	377	LYNSASFSTF	0.914
A24	HLA-A*24:35	395	402	VYADSFVI	0.6394
A24	HLA-A*24:35	448	456	NYNYLYRLF	0.9353
A24	HLA-A*24:35	488	497	CYFPLQSYGF	0.5885
A24	HLA-A*24:35	489	497	YFPLQSYGF	0.9442
A24	HLA-A*24:35	504	515	GYQPYRVVVLSF	0.6707
A24	HLA-A*24:35	507	515	PYRVVVLSF	0.8166
A24	HLA-A*24:35	558	565	KFLPFQQF	0.6948
A24	HLA-A*24:35	634	643	RVYSTGSNVF	0.7146
A24	HLA-A*24:35	635	643	VYSTGSNVF	0.9696
A24	HLA-A*24:35	755	763	QYGSFCTQL	0.5614
A24	HLA-A*24:35	788	797	IYKTPPIKDF	0.9226
A24	HLA-A*24:35	816	823	SFIEDLLF	0.7253
A24	HLA-A*24:35	1066	1075	TYVPAQEKNF	0.9479
A24	HLA-A*24:35	1094	1102	VFVSNGTHW	0.8658
A24	HLA-A*24:35	1094	1103	VFVSNGTHWF	0.8335
A24	HLA-A*24:35	1101	1109	HWFVTQRNF	0.754
A24	HLA-A*24:35	1137	1148	VYDPLQPELDSF	0.8174
A24	HLA-A*24:35	1208	1216	QYIKWPWYI	0.9613
A24	HLA-A*24:35	1208	1217	QYIKWPWYIW	0.7582
A24	HLA-A*24:35	1211	1220	KWPWYIWLGF	0.6582
A24	HLA-A*24:37	37	48	YYPDKVFRSSVL	0.601
A24	HLA-A*24:37	57	65	PFFSNVTWF	0.8728
A24	HLA-A*24:37	78	86	RFDNPVLPF	0.8538
A24	HLA-A*24:37	143	152	VYYHKNNKSW	0.7378
A24	HLA-A*24:37	144	152	YYHKNNKSW	0.8416
A24	HLA-A*24:37	151	159	SWMESEFRV	0.5046
A24	HLA-A*24:37	159	168	VYSSANNCTF	0.9582
A24	HLA-A*24:37	167	175	TFEYVSQPF	0.5365
A24	HLA-A*24:37	169	177	EYVSQPFLM	0.8484

A24	HLA-A*24:37	203	212	IYSKHTPINL	0.7085
A24	HLA-A*24:37	264	275	AYYVGYLQPRTF	0.8037
A24	HLA-A*24:37	265	275	YYVGYLQPRTF	0.9005
A24	HLA-A*24:37	268	277	GYLQPRTFLL	0.731
A24	HLA-A*24:37	269	277	YLQPRTFLL	0.5359
A24	HLA-A*24:37	312	320	IYQTSNFRV	0.785
A24	HLA-A*24:37	328	338	RFPNITNLCPF	0.8789
A24	HLA-A*24:37	346	353	RFASVYAW	0.521
A24	HLA-A*24:37	368	377	LYNSASFSTF	0.914
A24	HLA-A*24:37	395	402	VYADSFVI	0.6394
A24	HLA-A*24:37	448	456	NYNYLYRLF	0.9353
A24	HLA-A*24:37	488	497	CYFPLQSYGF	0.5885
A24	HLA-A*24:37	489	497	YFPLQSYGF	0.9442
A24	HLA-A*24:37	504	515	GYQPYRVVVLSF	0.6707
A24	HLA-A*24:37	507	515	PYRVVVLSF	0.8166
A24	HLA-A*24:37	558	565	KFLPFQQF	0.6948
A24	HLA-A*24:37	634	643	RVYSTGSNVF	0.7146
A24	HLA-A*24:37	635	643	VYSTGSNVF	0.9696
A24	HLA-A*24:37	755	763	QYGSFCTQL	0.5614
A24	HLA-A*24:37	788	797	IYKTPPIKDF	0.9226
A24	HLA-A*24:37	816	823	SFIEDLLF	0.7253
A24	HLA-A*24:37	1066	1075	TYVPAQEKNF	0.9479
A24	HLA-A*24:37	1094	1102	VFVSNGTHW	0.8658
A24	HLA-A*24:37	1094	1103	VFVSNGTHWF	0.8335
A24	HLA-A*24:37	1101	1109	HWFVTQRNF	0.754
A24	HLA-A*24:37	1137	1148	VYDPLQPELDSF	0.8174
A24	HLA-A*24:37	1208	1216	QYIKWPWYI	0.9613
A24	HLA-A*24:37	1208	1217	QYIKWPWYIW	0.7582
A24	HLA-A*24:37	1211	1220	KWPWYIWLGF	0.6582
A24	HLA-A*24:38	37	48	YYPDKVFRSSVL	0.601
A24	HLA-A*24:38	57	65	PFFSNVTWF	0.8728
A24	HLA-A*24:38	78	86	RFDNPVLPF	0.8538
A24	HLA-A*24:38	143	152	VYYHKNNKSW	0.7378
A24	HLA-A*24:38	144	152	YYHKNNKSW	0.8416
A24	HLA-A*24:38	151	159	SWMESEFRV	0.5046
A24	HLA-A*24:38	159	168	VYSSANNCTF	0.9582
A24	HLA-A*24:38	167	175	TFEYVSQPF	0.5365
A24	HLA-A*24:38	169	177	EYVSQPFLM	0.8484
A24	HLA-A*24:38	203	212	IYSKHTPINL	0.7085
A24	HLA-A*24:38	264	275	AYYVGYLQPRTF	0.8037
A24	HLA-A*24:38	265	275	YYVGYLQPRTF	0.9005
A24	HLA-A*24:38	268	277	GYLQPRTFLL	0.731
A24	HLA-A*24:38	269	277	YLQPRTFLL	0.5359
A24	HLA-A*24:38	312	320	IYQTSNFRV	0.785
A24	HLA-A*24:38	328	338	RFPNITNLCPF	0.8789
A24	HLA-A*24:38	346	353	RFASVYAW	0.521
A24	HLA-A*24:38	368	377	LYNSASFSTF	0.914
A24	HLA-A*24:38	395	402	VYADSFVI	0.6394
A24	HLA-A*24:38	448	456	NYNYLYRLF	0.9353
A24	HLA-A*24:38	488	497	CYFPLQSYGF	0.5885

A24	HLA-A*24:38	489	497	YFPLQSYGF	0.9442
A24	HLA-A*24:38	504	515	GYQPYRVVVLSF	0.6707
A24	HLA-A*24:38	507	515	PYRVVVLSF	0.8166
A24	HLA-A*24:38	558	565	KFLPFQQF	0.6948
A24	HLA-A*24:38	634	643	RVYSTGSNVF	0.7146
A24	HLA-A*24:38	635	643	VYSTGSNVF	0.9696
A24	HLA-A*24:38	755	763	QYGSFCTQL	0.5614
A24	HLA-A*24:38	788	797	IYKTPPIKDF	0.9226
A24	HLA-A*24:38	816	823	SFIEDLLF	0.7253
A24	HLA-A*24:38	1066	1075	TYVPAQEKNF	0.9479
A24	HLA-A*24:38	1094	1102	VFVSNGTHW	0.8658
A24	HLA-A*24:38	1094	1103	VFVSNGTHWF	0.8335
A24	HLA-A*24:38	1101	1109	HWFVTQRNF	0.754
A24	HLA-A*24:38	1137	1148	VYDPLQPELDSF	0.8174
A24	HLA-A*24:38	1208	1216	QYIKWPWYI	0.9613
A24	HLA-A*24:38	1208	1217	QYIKWPWYIW	0.7582
A24	HLA-A*24:38	1211	1220	KWPWYIWLGF	0.6582
A24	HLA-A*24:39	37	48	YYPDKVFRSSVL	0.601
A24	HLA-A*24:39	57	65	PFFSNVTWF	0.8728
A24	HLA-A*24:39	78	86	RFDNPVLPF	0.8538
A24	HLA-A*24:39	143	152	VYYHKNNKSW	0.7378
A24	HLA-A*24:39	144	152	YYHKNNKSW	0.8416
A24	HLA-A*24:39	151	159	SWMESEFRV	0.5046
A24	HLA-A*24:39	159	168	VYSSANNCTF	0.9582
A24	HLA-A*24:39	167	175	TFEYVSQPF	0.5365
A24	HLA-A*24:39	169	177	EYVSQPFLM	0.8484
A24	HLA-A*24:39	203	212	IYSKHTPINL	0.7085
A24	HLA-A*24:39	264	275	AYYVGYLQPRTF	0.8037
A24	HLA-A*24:39	265	275	YYVGYLQPRTF	0.9005
A24	HLA-A*24:39	268	277	GYLQPRTFLL	0.731
A24	HLA-A*24:39	269	277	YLQPRTFLL	0.5359
A24	HLA-A*24:39	312	320	IYQTSNFRV	0.785
A24	HLA-A*24:39	328	338	RFPNITNLCPF	0.8789
A24	HLA-A*24:39	346	353	RFASVYAW	0.521
A24	HLA-A*24:39	368	377	LYNSASFSTF	0.914
A24	HLA-A*24:39	395	402	VYADSFVI	0.6394
A24	HLA-A*24:39	448	456	NYNYLYRLF	0.9353
A24	HLA-A*24:39	488	497	CYFPLQSYGF	0.5885
A24	HLA-A*24:39	489	497	YFPLQSYGF	0.9442
A24	HLA-A*24:39	504	515	GYQPYRVVVLSF	0.6707
A24	HLA-A*24:39	507	515	PYRVVVLSF	0.8166
A24	HLA-A*24:39	558	565	KFLPFQQF	0.6948
A24	HLA-A*24:39	634	643	RVYSTGSNVF	0.7146
A24	HLA-A*24:39	635	643	VYSTGSNVF	0.9696
A24	HLA-A*24:39	755	763	QYGSFCTQL	0.5614
A24	HLA-A*24:39	788	797	IYKTPPIKDF	0.9226
A24	HLA-A*24:39	816	823	SFIEDLLF	0.7253
A24	HLA-A*24:39	1066	1075	TYVPAQEKNF	0.9479
A24	HLA-A*24:39	1094	1102	VFVSNGTHW	0.8658
A24	HLA-A*24:39	1094	1103	VFVSNGTHWF	0.8335

A24	HLA-A*24:39	1101	1109	HWFVTQRNF	0.754
A24	HLA-A*24:39	1137	1148	VYDPLQPELDSF	0.8174
A24	HLA-A*24:39	1208	1216	QYIKWPWYI	0.9613
A24	HLA-A*24:39	1208	1217	QYIKWPWYIW	0.7582
A24	HLA-A*24:39	1211	1220	KWPWYIWLGF	0.6582
A24	HLA-A*24:43	37	48	YYPDKVFRSSVL	0.601
A24	HLA-A*24:43	57	65	PFFSNVTWF	0.8728
A24	HLA-A*24:43	78	86	RFDNPVLPF	0.8538
A24	HLA-A*24:43	143	152	VYYHKNNKSW	0.7378
A24	HLA-A*24:43	144	152	YYHKNNKSW	0.8416
A24	HLA-A*24:43	151	159	SWMESEFRV	0.5046
A24	HLA-A*24:43	159	168	VYSSANNCTF	0.9582
A24	HLA-A*24:43	167	175	TFEYVSQPF	0.5365
A24	HLA-A*24:43	169	177	EYVSQPFLM	0.8484
A24	HLA-A*24:43	203	212	IYSKHTPINL	0.7085
A24	HLA-A*24:43	264	275	AYYVGYLQPRTF	0.8037
A24	HLA-A*24:43	265	275	YYVGYLQPRTF	0.9005
A24	HLA-A*24:43	268	277	GYLQPRTFLL	0.731
A24	HLA-A*24:43	269	277	YLQPRTFLL	0.5359
A24	HLA-A*24:43	312	320	IYQTSNFRV	0.785
A24	HLA-A*24:43	328	338	RFPNITNLCPF	0.8789
A24	HLA-A*24:43	346	353	RFASVYAW	0.521
A24	HLA-A*24:43	368	377	LYNSASFSTF	0.914
A24	HLA-A*24:43	395	402	VYADSFVI	0.6394
A24	HLA-A*24:43	448	456	NYNYLYRLF	0.9353
A24	HLA-A*24:43	488	497	CYFPLQSYGF	0.5885
A24	HLA-A*24:43	489	497	YFPLQSYGF	0.9442
A24	HLA-A*24:43	504	515	GYQPYRVVVLSF	0.6707
A24	HLA-A*24:43	507	515	PYRVVVLSF	0.8166
A24	HLA-A*24:43	558	565	KFLPFQQF	0.6948
A24	HLA-A*24:43	634	643	RVYSTGSNVF	0.7146
A24	HLA-A*24:43	635	643	VYSTGSNVF	0.9696
A24	HLA-A*24:43	755	763	QYGSFCTQL	0.5614
A24	HLA-A*24:43	788	797	IYKTPPIKDF	0.9226
A24	HLA-A*24:43	816	823	SFIEDLLF	0.7253
A24	HLA-A*24:43	1066	1075	TYVPAQEKNF	0.9479
A24	HLA-A*24:43	1094	1102	VFVSNGTHW	0.8658
A24	HLA-A*24:43	1094	1103	VFVSNGTHWF	0.8335
A24	HLA-A*24:43	1101	1109	HWFVTQRNF	0.754
A24	HLA-A*24:43	1137	1148	VYDPLQPELDSF	0.8174
A24	HLA-A*24:43	1208	1216	QYIKWPWYI	0.9613
A24	HLA-A*24:43	1208	1217	QYIKWPWYIW	0.7582
A24	HLA-A*24:43	1211	1220	KWPWYIWLGF	0.6582
A24	HLA-A*24:46	37	48	YYPDKVFRSSVL	0.5855
A24	HLA-A*24:46	57	65	PFFSNVTWF	0.8459
A24	HLA-A*24:46	78	86	RFDNPVLPF	0.8229
A24	HLA-A*24:46	159	168	VYSSANNCTF	0.9103
A24	HLA-A*24:46	169	177	EYVSQPFLM	0.851
A24	HLA-A*24:46	264	275	AYYVGYLQPRTF	0.6417
A24	HLA-A*24:46	265	275	YYVGYLQPRTF	0.8194

A24	HLA-A*24:46	268	277	GYLQPRTFLL	0.6533
A24	HLA-A*24:46	269	277	YLQPRTFLL	0.5285
A24	HLA-A*24:46	312	320	IYQTSNFRV	0.6584
A24	HLA-A*24:46	328	338	RFPNITNLCPF	0.7845
A24	HLA-A*24:46	368	377	LYNSASFSTF	0.8513
A24	HLA-A*24:46	395	402	VYADSFVI	0.5561
A24	HLA-A*24:46	448	456	NYNYLYRLF	0.871
A24	HLA-A*24:46	488	497	CYFPLQSYGF	0.5138
A24	HLA-A*24:46	489	497	YFPLQSYGF	0.9325
A24	HLA-A*24:46	504	515	GYQPYRVVVLSF	0.5468
A24	HLA-A*24:46	635	643	VYSTGSNVF	0.9278
A24	HLA-A*24:46	755	763	QYGSFCTQL	0.5152
A24	HLA-A*24:46	816	823	SFIEDLLF	0.7078
A24	HLA-A*24:46	1066	1075	TYVPAQEKNF	0.9054
A24	HLA-A*24:46	1094	1103	VFVSNGTHWF	0.756
A24	HLA-A*24:46	1137	1148	VYDPLQPELDSF	0.7602
A24	HLA-A*24:46	1208	1216	QYIKWPWYI	0.9343
A24	HLA-A*24:46	1208	1217	QYIKWPWYIW	0.6481
A24	HLA-A*24:46	1211	1220	KWPWYIWLGF	0.5239
A24	HLA-A*24:47	37	48	YYPDKVFRSSVL	0.601
A24	HLA-A*24:47	57	65	PFFSNVTWF	0.8728
A24	HLA-A*24:47	78	86	RFDNPVLPF	0.8538
A24	HLA-A*24:47	143	152	VYYHKNNKSW	0.7378
A24	HLA-A*24:47	144	152	YYHKNNKSW	0.8416
A24	HLA-A*24:47	151	159	SWMESEFRV	0.5046
A24	HLA-A*24:47	159	168	VYSSANNCTF	0.9582
A24	HLA-A*24:47	167	175	TFEYVSQPF	0.5365
A24	HLA-A*24:47	169	177	EYVSQPFLM	0.8484
A24	HLA-A*24:47	203	212	IYSKHTPINL	0.7085
A24	HLA-A*24:47	264	275	AYYVGYLQPRTF	0.8037
A24	HLA-A*24:47	265	275	YYVGYLQPRTF	0.9005
A24	HLA-A*24:47	268	277	GYLQPRTFLL	0.731
A24	HLA-A*24:47	269	277	YLQPRTFLL	0.5359
A24	HLA-A*24:47	312	320	IYQTSNFRV	0.785
A24	HLA-A*24:47	328	338	RFPNITNLCPF	0.8789
A24	HLA-A*24:47	346	353	RFASVYAW	0.521
A24	HLA-A*24:47	368	377	LYNSASFSTF	0.914
A24	HLA-A*24:47	395	402	VYADSFVI	0.6394
A24	HLA-A*24:47	448	456	NYNYLYRLF	0.9353
A24	HLA-A*24:47	488	497	CYFPLQSYGF	0.5885
A24	HLA-A*24:47	489	497	YFPLQSYGF	0.9442
A24	HLA-A*24:47	504	515	GYQPYRVVVLSF	0.6707
A24	HLA-A*24:47	507	515	PYRVVVLSF	0.8166
A24	HLA-A*24:47	558	565	KFLPFQQF	0.6948
A24	HLA-A*24:47	634	643	RVYSTGSNVF	0.7146
A24	HLA-A*24:47	635	643	VYSTGSNVF	0.9696
A24	HLA-A*24:47	755	763	QYGSFCTQL	0.5614
A24	HLA-A*24:47	788	797	IYKTPPIKDF	0.9226
A24	HLA-A*24:47	816	823	SFIEDLLF	0.7253
A24	HLA-A*24:47	1066	1075	TYVPAQEKNF	0.9479

A24	HLA-A*24:47	1094	1102	VFVSNGTHW	0.8658
A24	HLA-A*24:47	1094	1103	VFVSNGTHWF	0.8335
A24	HLA-A*24:47	1101	1109	HWFVTQRNF	0.754
A24	HLA-A*24:47	1137	1148	VYDPLQPELDSF	0.8174
A24	HLA-A*24:47	1208	1216	QYIKWPWYI	0.9613
A24	HLA-A*24:47	1208	1217	QYIKWPWYIW	0.7582
A24	HLA-A*24:47	1211	1220	KWPWYIWLGF	0.6582
A24	HLA-A*24:49	37	48	YYPDKVFRSSVL	0.601
A24	HLA-A*24:49	57	65	PFFSNVTWF	0.8728
A24	HLA-A*24:49	78	86	RFDNPVLPF	0.8538
A24	HLA-A*24:49	143	152	VYYHKNNKSW	0.7378
A24	HLA-A*24:49	144	152	YYHKNNKSW	0.8416
A24	HLA-A*24:49	151	159	SWMESEFRV	0.5046
A24	HLA-A*24:49	159	168	VYSSANNCTF	0.9582
A24	HLA-A*24:49	167	175	TFEYVSQPF	0.5365
A24	HLA-A*24:49	169	177	EYVSQPFLM	0.8484
A24	HLA-A*24:49	203	212	IYSKHTPINL	0.7085
A24	HLA-A*24:49	264	275	AYYVGYLQPRTF	0.8037
A24	HLA-A*24:49	265	275	YYVGYLQPRTF	0.9005
A24	HLA-A*24:49	268	277	GYLQPRTFLL	0.731
A24	HLA-A*24:49	269	277	YLQPRTFLL	0.5359
A24	HLA-A*24:49	312	320	IYQTSNFRV	0.785
A24	HLA-A*24:49	328	338	RFPNITNLCPF	0.8789
A24	HLA-A*24:49	346	353	RFASVYAW	0.521
A24	HLA-A*24:49	368	377	LYNSASFSTF	0.914
A24	HLA-A*24:49	395	402	VYADSFVI	0.6394
A24	HLA-A*24:49	448	456	NYNYLYRLF	0.9353
A24	HLA-A*24:49	488	497	CYFPLQSYGF	0.5885
A24	HLA-A*24:49	489	497	YFPLQSYGF	0.9442
A24	HLA-A*24:49	504	515	GYQPYRVVVLSF	0.6707
A24	HLA-A*24:49	507	515	PYRVVVLSF	0.8166
A24	HLA-A*24:49	558	565	KFLPFQQF	0.6948
A24	HLA-A*24:49	634	643	RVYSTGSNVF	0.7146
A24	HLA-A*24:49	635	643	VYSTGSNVF	0.9696
A24	HLA-A*24:49	755	763	QYGSFCTQL	0.5614
A24	HLA-A*24:49	788	797	IYKTPPIKDF	0.9226
A24	HLA-A*24:49	816	823	SFIEDLLF	0.7253
A24	HLA-A*24:49	1066	1075	TYVPAQEKNF	0.9479
A24	HLA-A*24:49	1094	1102	VFVSNGTHW	0.8658
A24	HLA-A*24:49	1094	1103	VFVSNGTHWF	0.8335
A24	HLA-A*24:49	1101	1109	HWFVTQRNF	0.754
A24	HLA-A*24:49	1137	1148	VYDPLQPELDSF	0.8174
A24	HLA-A*24:49	1208	1216	QYIKWPWYI	0.9613
A24	HLA-A*24:49	1208	1217	QYIKWPWYIW	0.7582
A24	HLA-A*24:49	1211	1220	KWPWYIWLGF	0.6582
B07	HLA-B*07:02	24	32	LPPAYTNSF	0.5263
B07	HLA-B*07:02	38	47	YPDKVFRSSV	0.5308
B07	HLA-B*07:02	208	216	TPINLVRDL	0.7332
B07	HLA-B*07:02	216	223	LPQGFSAL	0.6854
B07	HLA-B*07:02	462	472	KPFERDISTEI	0.6344

B07	HLA-B*07:02	506	513	QPYRVVVL	0.6389
B07	HLA-B*07:02	526	534	GPKKSTNLV	0.6037
B07	HLA-B*07:02	620	629	VPVAIHADQL	0.5101
B07	HLA-B*07:02	680	688	SPRRARSVA	0.7296
B07	HLA-B*07:02	714	722	IPTNFTISV	0.6596
B07	HLA-B*07:02	1056	1063	APHGVVFL	0.6399
B07	HLA-B*07:02	1261	1270	SEPVLKGVKL	0.6496
B07	HLA-B*07:03	24	32	LPPAYTNSF	0.6635
B07	HLA-B*07:03	38	47	YPDKVFRSSV	0.6468
B07	HLA-B*07:03	38	48	YPDKVFRSSVL	0.5441
B07	HLA-B*07:03	208	216	TPINLVRDL	0.7801
B07	HLA-B*07:03	216	223	LPQGFSAL	0.8331
B07	HLA-B*07:03	462	472	KPFERDISTEI	0.5705
B07	HLA-B*07:03	506	513	QPYRVVVL	0.8112
B07	HLA-B*07:03	526	533	GPKKSTNL	0.5568
B07	HLA-B*07:03	526	534	GPKKSTNLV	0.6514
B07	HLA-B*07:03	588	597	TPCSFGGVSV	0.525
B07	HLA-B*07:03	620	629	VPVAIHADQL	0.5699
B07	HLA-B*07:03	680	688	SPRRARSVA	0.7613
B07	HLA-B*07:03	714	722	IPTNFTISV	0.7313
B07	HLA-B*07:03	869	877	MIAQYTSAL	0.584
B07	HLA-B*07:03	1052	1060	FPQSAPHGV	0.5776
B07	HLA-B*07:03	1052	1062	FPQSAPHGVVF	0.6134
B07	HLA-B*07:03	1056	1063	APHGVVFL	0.7644
B07	HLA-B*07:03	1261	1270	SEPVLKGVKL	0.7262
B07	HLA-B*07:04	208	216	TPINLVRDL	0.6264
B07	HLA-B*07:04	462	472	KPFERDISTEI	0.5191
B07	HLA-B*07:04	506	513	QPYRVVVL	0.6271
B07	HLA-B*07:04	526	534	GPKKSTNLV	0.5173
B07	HLA-B*07:04	680	688	SPRRARSVA	0.6865
B07	HLA-B*07:04	714	722	IPTNFTISV	0.5207
B07	HLA-B*07:04	1261	1270	SEPVLKGVKL	0.5427
B07	HLA-B*07:05	24	32	LPPAYTNSF	0.5378
B07	HLA-B*07:05	38	47	YPDKVFRSSV	0.6462
B07	HLA-B*07:05	208	216	TPINLVRDL	0.7572
B07	HLA-B*07:05	216	223	LPQGFSAL	0.7578
B07	HLA-B*07:05	462	472	KPFERDISTEI	0.6075
B07	HLA-B*07:05	506	513	QPYRVVVL	0.7004
B07	HLA-B*07:05	526	534	GPKKSTNLV	0.6522
B07	HLA-B*07:05	588	597	TPCSFGGVSV	0.5218
B07	HLA-B*07:05	620	629	VPVAIHADQL	0.5518
B07	HLA-B*07:05	680	688	SPRRARSVA	0.7396
B07	HLA-B*07:05	714	722	IPTNFTISV	0.75
B07	HLA-B*07:05	1056	1063	APHGVVFL	0.6769
B07	HLA-B*07:05	1261	1270	SEPVLKGVKL	0.6685
B07	HLA-B*07:06	24	32	LPPAYTNSF	0.5378
B07	HLA-B*07:06	38	47	YPDKVFRSSV	0.6462
B07	HLA-B*07:06	208	216	TPINLVRDL	0.7572
B07	HLA-B*07:06	216	223	LPQGFSAL	0.7578
B07	HLA-B*07:06	462	472	KPFERDISTEI	0.6075

B07	HLA-B*07:06	506	513	QPYRVVVL	0.7004
B07	HLA-B*07:06	526	534	GPKKSTNLV	0.6522
B07	HLA-B*07:06	588	597	TPCSFGGVSV	0.5218
B07	HLA-B*07:06	620	629	VPVAIHADQL	0.5518
B07	HLA-B*07:06	680	688	SPRRARSVA	0.7396
B07	HLA-B*07:06	714	722	IPTNFTISV	0.75
B07	HLA-B*07:06	1056	1063	APHGVVFL	0.6769
B07	HLA-B*07:06	1261	1270	SEPVLKGVKL	0.6685
B07	HLA-B*07:15	38	47	YPDKVFRSSV	0.6004
B07	HLA-B*07:15	208	216	TPINLVRDL	0.7101
B07	HLA-B*07:15	216	223	LPQGFSAL	0.6649
B07	HLA-B*07:15	506	513	QPYRVVVL	0.6118
B07	HLA-B*07:15	526	534	GPKKSTNLV	0.5338
B07	HLA-B*07:15	680	688	SPRRARSVA	0.6589
B07	HLA-B*07:15	714	722	IPTNFTISV	0.7194
B07	HLA-B*07:15	1052	1060	FPQSAPHGV	0.5075
B07	HLA-B*07:15	1056	1063	APHGVVFL	0.6254
B07	HLA-B*07:15	1261	1270	SEPVLKGVKL	0.5393
B07	HLA-B*07:19	24	32	LPPAYTNSF	0.5493
B07	HLA-B*07:19	208	216	TPINLVRDL	0.7209
B07	HLA-B*07:19	216	223	LPQGFSAL	0.5972
B07	HLA-B*07:19	462	472	KPFERDISTEI	0.5462
B07	HLA-B*07:19	506	513	QPYRVVVL	0.744
B07	HLA-B*07:19	526	534	GPKKSTNLV	0.6123
B07	HLA-B*07:19	680	687	SPRRARSV	0.5347
B07	HLA-B*07:19	680	688	SPRRARSVA	0.7444
B07	HLA-B*07:19	714	722	IPTNFTISV	0.6042
B07	HLA-B*07:19	1056	1063	APHGVVFL	0.5804
B07	HLA-B*07:19	1261	1270	SEPVLKGVKL	0.6196
B07	HLA-B*07:20	24	32	LPPAYTNSF	0.5771
B07	HLA-B*07:20	208	216	TPINLVRDL	0.6858
B07	HLA-B*07:20	216	223	LPQGFSAL	0.5023
B07	HLA-B*07:21	24	32	LPPAYTNSF	0.5263
B07	HLA-B*07:21	38	47	YPDKVFRSSV	0.5308
B07	HLA-B*07:21	208	216	TPINLVRDL	0.7332
B07	HLA-B*07:21	216	223	LPQGFSAL	0.6854
B07	HLA-B*07:21	462	472	KPFERDISTEI	0.6344
B07	HLA-B*07:21	506	513	QPYRVVVL	0.6389
B07	HLA-B*07:21	526	534	GPKKSTNLV	0.6037
B07	HLA-B*07:21	620	629	VPVAIHADQL	0.5101
B07	HLA-B*07:21	680	688	SPRRARSVA	0.7296
B07	HLA-B*07:21	714	722	IPTNFTISV	0.6596
B07	HLA-B*07:21	1056	1063	APHGVVFL	0.6399
B07	HLA-B*07:21	1261	1270	SEPVLKGVKL	0.6496
B07	HLA-B*07:22	24	32	LPPAYTNSF	0.5263
B07	HLA-B*07:22	38	47	YPDKVFRSSV	0.5308
B07	HLA-B*07:22	208	216	TPINLVRDL	0.7332
B07	HLA-B*07:22	216	223	LPQGFSAL	0.6854
B07	HLA-B*07:22	462	472	KPFERDISTEI	0.6344
B07	HLA-B*07:22	506	513	QPYRVVVL	0.6389

B07	HLA-B*07:22	526	534	GPKKSTNLV	0.6037
B07	HLA-B*07:22	620	629	VPVAIHADQL	0.5101
B07	HLA-B*07:22	680	688	SPRRARSVA	0.7296
B07	HLA-B*07:22	714	722	IPTNFTISV	0.6596
B07	HLA-B*07:22	1056	1063	APHGVVFL	0.6399
B07	HLA-B*07:22	1261	1270	SEPVLKGVKL	0.6496
B07	HLA-B*07:24	24	32	LPPAYTNSF	0.6317
B07	HLA-B*07:24	38	47	YPDKVFRSSV	0.5009
B07	HLA-B*07:24	84	92	LPFNDGVYF	0.6784
B07	HLA-B*07:24	208	216	TPINLVRDL	0.787
B07	HLA-B*07:24	216	223	LPQGFSAL	0.6678
B07	HLA-B*07:24	506	513	QPYRVVVL	0.6042
B07	HLA-B*07:24	526	534	GPKKSTNLV	0.5315
B07	HLA-B*07:24	680	688	SPRRARSVA	0.6404
B07	HLA-B*07:24	714	722	IPTNFTISV	0.6579
B07	HLA-B*07:24	1052	1062	FPQSAPHGVVF	0.5403
B07	HLA-B*07:24	1261	1270	SEPVLKGVKL	0.6013
B07	HLA-B*07:25	24	32	LPPAYTNSF	0.6744
B07	HLA-B*07:25	84	92	LPFNDGVYF	0.6682
B07	HLA-B*07:25	208	216	TPINLVRDL	0.7696
B07	HLA-B*07:25	216	223	LPQGFSAL	0.5739
B07	HLA-B*07:25	506	513	QPYRVVVL	0.6556
B07	HLA-B*07:25	620	629	VPVAIHADQL	0.5421
B07	HLA-B*07:25	714	722	IPTNFTISV	0.6917
B07	HLA-B*07:25	1052	1060	FPQSAPHGV	0.5272
B07	HLA-B*07:25	1052	1062	FPQSAPHGVVF	0.524
B07	HLA-B*07:25	1056	1063	APHGVVFL	0.6273
B07	HLA-B*07:25	1261	1270	SEPVLKGVKL	0.6103
B07	HLA-B*07:25	1262	1270	EPVLKGVKL	0.6809
B07	HLA-B*07:26	208	216	TPINLVRDL	0.6472
B07	HLA-B*07:26	506	513	QPYRVVVL	0.5503
B07	HLA-B*07:26	680	688	SPRRARSVA	0.542
B07	HLA-B*07:26	714	722	IPTNFTISV	0.5255
B07	HLA-B*07:30	24	32	LPPAYTNSF	0.5263
B07	HLA-B*07:30	38	47	YPDKVFRSSV	0.5308
B07	HLA-B*07:30	208	216	TPINLVRDL	0.7332
B07	HLA-B*07:30	216	223	LPQGFSAL	0.6854
B07	HLA-B*07:30	462	472	KPFERDISTEI	0.6344
B07	HLA-B*07:30	506	513	QPYRVVVL	0.6389
B07	HLA-B*07:30	526	534	GPKKSTNLV	0.6037
B07	HLA-B*07:30	620	629	VPVAIHADQL	0.5101
B07	HLA-B*07:30	680	688	SPRRARSVA	0.7296
B07	HLA-B*07:30	714	722	IPTNFTISV	0.6596
B07	HLA-B*07:30	1056	1063	APHGVVFL	0.6399
B07	HLA-B*07:30	1261	1270	SEPVLKGVKL	0.6496
B07	HLA-B*07:31	24	32	LPPAYTNSF	0.6652
B07	HLA-B*07:31	38	47	YPDKVFRSSV	0.6505
B07	HLA-B*07:31	84	92	LPFNDGVYF	0.7128
B07	HLA-B*07:31	208	216	TPINLVRDL	0.8331
B07	HLA-B*07:31	216	223	LPQGFSAL	0.8036

B07	HLA-B*07:31	229	238	LPIGINITRF	0.5034
B07	HLA-B*07:31	462	472	KPFERDISTEI	0.6827
B07	HLA-B*07:31	506	513	QPYRVVVL	0.7867
B07	HLA-B*07:31	526	534	GPKKSTNLV	0.7144
B07	HLA-B*07:31	620	629	VPVAIHADQL	0.6023
B07	HLA-B*07:31	680	688	SPRRARSVA	0.8018
B07	HLA-B*07:31	714	722	IPTNFTISV	0.7547
B07	HLA-B*07:31	1052	1060	FPQSAPHGV	0.5262
B07	HLA-B*07:31	1052	1062	FPQSAPHGVVF	0.5664
B07	HLA-B*07:31	1056	1063	APHGVVFL	0.7394
B07	HLA-B*07:31	1261	1270	SEPVLKGVKL	0.7452
B07	HLA-B*07:31	1262	1270	EPVLKGVKL	0.7089
B07	HLA-B*07:33	24	32	LPPAYTNSF	0.5263
B07	HLA-B*07:33	38	47	YPDKVFRSSV	0.5308
B07	HLA-B*07:33	208	216	TPINLVRDL	0.7332
B07	HLA-B*07:33	216	223	LPQGFSAL	0.6854
B07	HLA-B*07:33	462	472	KPFERDISTEI	0.6344
B07	HLA-B*07:33	506	513	QPYRVVVL	0.6389
B07	HLA-B*07:33	526	534	GPKKSTNLV	0.6037
B07	HLA-B*07:33	620	629	VPVAIHADQL	0.5101
B07	HLA-B*07:33	680	688	SPRRARSVA	0.7296
B07	HLA-B*07:33	714	722	IPTNFTISV	0.6596
B07	HLA-B*07:33	1056	1063	APHGVVFL	0.6399
B07	HLA-B*07:33	1261	1270	SEPVLKGVKL	0.6496
B07	HLA-B*07:34	24	32	LPPAYTNSF	0.5137
B07	HLA-B*07:34	84	92	LPFNDGVYF	0.6562
B07	HLA-B*07:34	208	216	TPINLVRDL	0.7702
B07	HLA-B*07:34	216	223	LPQGFSAL	0.6635
B07	HLA-B*07:34	506	513	QPYRVVVL	0.7653
B07	HLA-B*07:34	526	534	GPKKSTNLV	0.5309
B07	HLA-B*07:34	680	688	SPRRARSVA	0.6471
B07	HLA-B*07:34	714	722	IPTNFTISV	0.7183
B07	HLA-B*07:34	1056	1063	APHGVVFL	0.6103
B07	HLA-B*07:34	1261	1270	SEPVLKGVKL	0.5539
B07	HLA-B*07:35	24	32	LPPAYTNSF	0.5263
B07	HLA-B*07:35	38	47	YPDKVFRSSV	0.5308
B07	HLA-B*07:35	208	216	TPINLVRDL	0.7332
B07	HLA-B*07:35	216	223	LPQGFSAL	0.6854
B07	HLA-B*07:35	462	472	KPFERDISTEI	0.6344
B07	HLA-B*07:35	506	513	QPYRVVVL	0.6389
B07	HLA-B*07:35	526	534	GPKKSTNLV	0.6037
B07	HLA-B*07:35	620	629	VPVAIHADQL	0.5101
B07	HLA-B*07:35	680	688	SPRRARSVA	0.7296
B07	HLA-B*07:35	714	722	IPTNFTISV	0.6596
B07	HLA-B*07:35	1056	1063	APHGVVFL	0.6399
B07	HLA-B*07:35	1261	1270	SEPVLKGVKL	0.6496
B07	HLA-B*07:39	24	32	LPPAYTNSF	0.5263
B07	HLA-B*07:39	38	47	YPDKVFRSSV	0.5308
B07	HLA-B*07:39	208	216	TPINLVRDL	0.7332
B07	HLA-B*07:39	216	223	LPQGFSAL	0.6854

B07	HLA-B*07:39	462	472	KPFERDISTEI	0.6344
B07	HLA-B*07:39	506	513	QPYRVVVL	0.6389
B07	HLA-B*07:39	526	534	GPKKSTNLV	0.6037
B07	HLA-B*07:39	620	629	VPVAIHADQL	0.5101
B07	HLA-B*07:39	680	688	SPRRARSVA	0.7296
B07	HLA-B*07:39	714	722	IPTNFTISV	0.6596
B07	HLA-B*07:39	1056	1063	APHGVVFL	0.6399
B07	HLA-B*07:39	1261	1270	SEPVLKGVKL	0.6496
B07	HLA-B*07:40	24	32	LPPAYTNSF	0.5378
B07	HLA-B*07:40	38	47	YPDKVFRSSV	0.6462
B07	HLA-B*07:40	208	216	TPINLVRDL	0.7572
B07	HLA-B*07:40	216	223	LPQGFSAL	0.7578
B07	HLA-B*07:40	462	472	KPFERDISTEI	0.6075
B07	HLA-B*07:40	506	513	QPYRVVVL	0.7004
B07	HLA-B*07:40	526	534	GPKKSTNLV	0.6522
B07	HLA-B*07:40	588	597	TPCSFGGVSV	0.5218
B07	HLA-B*07:40	620	629	VPVAIHADQL	0.5518
B07	HLA-B*07:40	680	688	SPRRARSVA	0.7396
B07	HLA-B*07:40	714	722	IPTNFTISV	0.75
B07	HLA-B*07:40	1056	1063	APHGVVFL	0.6769
B07	HLA-B*07:40	1261	1270	SEPVLKGVKL	0.6685
B07	HLA-B*07:41	24	32	LPPAYTNSF	0.5263
B07	HLA-B*07:41	38	47	YPDKVFRSSV	0.5308
B07	HLA-B*07:41	208	216	TPINLVRDL	0.7332
B07	HLA-B*07:41	216	223	LPQGFSAL	0.6854
B07	HLA-B*07:41	462	472	KPFERDISTEI	0.6344
B07	HLA-B*07:41	506	513	QPYRVVVL	0.6389
B07	HLA-B*07:41	526	534	GPKKSTNLV	0.6037
B07	HLA-B*07:41	620	629	VPVAIHADQL	0.5101
B07	HLA-B*07:41	680	688	SPRRARSVA	0.7296
B07	HLA-B*07:41	714	722	IPTNFTISV	0.6596
B07	HLA-B*07:41	1056	1063	APHGVVFL	0.6399
B07	HLA-B*07:41	1261	1270	SEPVLKGVKL	0.6496
B07	HLA-B*07:42	24	32	LPPAYTNSF	0.5263
B07	HLA-B*07:42	38	47	YPDKVFRSSV	0.5308
B07	HLA-B*07:42	208	216	TPINLVRDL	0.7332
B07	HLA-B*07:42	216	223	LPQGFSAL	0.6854
B07	HLA-B*07:42	462	472	KPFERDISTEI	0.6344
B07	HLA-B*07:42	506	513	QPYRVVVL	0.6389
B07	HLA-B*07:42	526	534	GPKKSTNLV	0.6037
B07	HLA-B*07:42	620	629	VPVAIHADQL	0.5101
B07	HLA-B*07:42	680	688	SPRRARSVA	0.7296
B07	HLA-B*07:42	714	722	IPTNFTISV	0.6596
B07	HLA-B*07:42	1056	1063	APHGVVFL	0.6399
B07	HLA-B*07:42	1261	1270	SEPVLKGVKL	0.6496
B07	HLA-B*07:43	84	92	LPFNDGVYF	0.6105
B07	HLA-B*07:43	208	216	TPINLVRDL	0.7315
B07	HLA-B*07:43	216	223	LPQGFSAL	0.5483
B07	HLA-B*07:43	506	513	QPYRVVVL	0.6776
B07	HLA-B*07:43	680	688	SPRRARSVA	0.6226

B07	HLA-B*07:43	714	722	IPTNFTISV	0.6031
B07	HLA-B*15:08	84	92	LPFNDGVYF	0.6442
B07	HLA-B*15:08	192	200	FVFKNIDGY	0.5399
B07	HLA-B*15:08	687	695	VASQSIIAY	0.7217
B07	HLA-B*15:08	699	707	LGAENSVAY	0.5058
B07	HLA-B*15:08	896	904	IPFAMQMAY	0.7301
B07	HLA-B*15:08	1054	1062	QSAPHGVVF	0.5652
B07	HLA-B*35:01	24	32	LPPAYTNSF	0.8786
B07	HLA-B*35:01	30	38	NSFTRGVYY	0.5335
B07	HLA-B*35:01	56	64	LPFFSNVTW	0.8069
B07	HLA-B*35:01	83	92	VLPFNDGVYF	0.7228
B07	HLA-B*35:01	84	91	LPFNDGVY	0.824
B07	HLA-B*35:01	84	92	LPFNDGVYF	0.9877
B07	HLA-B*35:01	162	170	SANNCTFEY	0.6698
B07	HLA-B*35:01	192	200	FVFKNIDGY	0.7837
B07	HLA-B*35:01	229	238	LPIGINITRF	0.8394
B07	HLA-B*35:01	258	266	WTAGAAAYY	0.5393
B07	HLA-B*35:01	271	279	QPRTFLLKY	0.6565
B07	HLA-B*35:01	321	329	QPTESIVRF	0.9476
B07	HLA-B*35:01	343	351	NATRFASVY	0.7573
B07	HLA-B*35:01	361	369	CVADYSVLY	0.5167
B07	HLA-B*35:01	478	486	TPCNGVEGF	0.5961
B07	HLA-B*35:01	604	612	TSNQVAVLY	0.5492
B07	HLA-B*35:01	625	633	HADQLTPTW	0.7176
B07	HLA-B*35:01	664	674	IPIGAGICASY	0.8514
B07	HLA-B*35:01	687	695	VASQSIIAY	0.9634
B07	HLA-B*35:01	699	707	LGAENSVAY	0.8303
B07	HLA-B*35:01	714	722	IPTNFTISV	0.5007
B07	HLA-B*35:01	861	869	LPPLLTDEM	0.6186
B07	HLA-B*35:01	865	873	LTDEMIAQY	0.6118
B07	HLA-B*35:01	892	900	AALQIPFAM	0.5789
B07	HLA-B*35:01	895	904	QIPFAMQMAY	0.6098
B07	HLA-B*35:01	896	904	IPFAMQMAY	0.9872
B07	HLA-B*35:01	898	906	FAMQMAYRF	0.7448
B07	HLA-B*35:01	1052	1062	FPQSAPHGVVF	0.8504
B07	HLA-B*35:01	1054	1062	QSAPHGVVF	0.6307
B07	HLA-B*35:01	1095	1103	FVSNGTHWF	0.5997
B07	HLA-B*35:03	84	92	LPFNDGVYF	0.8449
B07	HLA-B*35:03	714	722	IPTNFTISV	0.6549
B07	HLA-B*35:03	861	869	LPPLLTDEM	0.674
B07	HLA-B*35:03	1052	1062	FPQSAPHGVVF	0.6557
B07	HLA-B*35:07	24	32	LPPAYTNSF	0.8786
B07	HLA-B*35:07	30	38	NSFTRGVYY	0.5335
B07	HLA-B*35:07	56	64	LPFFSNVTW	0.8069
B07	HLA-B*35:07	83	92	VLPFNDGVYF	0.7228
B07	HLA-B*35:07	84	91	LPFNDGVY	0.824
B07	HLA-B*35:07	84	92	LPFNDGVYF	0.9877
B07	HLA-B*35:07	162	170	SANNCTFEY	0.6698
B07	HLA-B*35:07	192	200	FVFKNIDGY	0.7837
B07	HLA-B*35:07	229	238	LPIGINITRF	0.8394

B07	HLA-B*35:07	258	266	WTAGAAAYY	0.5393
B07	HLA-B*35:07	271	279	QPRTFLLKY	0.6565
B07	HLA-B*35:07	321	329	QPTESIVRF	0.9476
B07	HLA-B*35:07	343	351	NATRFASVY	0.7573
B07	HLA-B*35:07	361	369	CVADYSVLY	0.5167
B07	HLA-B*35:07	478	486	TPCNGVEGF	0.5961
B07	HLA-B*35:07	604	612	TSNQVAVLY	0.5492
B07	HLA-B*35:07	625	633	HADQLTPTW	0.7176
B07	HLA-B*35:07	664	674	IPIGAGICASY	0.8514
B07	HLA-B*35:07	687	695	VASQSIIAY	0.9634
B07	HLA-B*35:07	699	707	LGAENSVAY	0.8303
B07	HLA-B*35:07	714	722	IPTNFTISV	0.5007
B07	HLA-B*35:07	861	869	LPPLLTDEM	0.6186
B07	HLA-B*35:07	865	873	LTDEMIAQY	0.6118
B07	HLA-B*35:07	892	900	AALQIPFAM	0.5789
B07	HLA-B*35:07	895	904	QIPFAMQMAY	0.6098
B07	HLA-B*35:07	896	904	IPFAMQMAY	0.9872
B07	HLA-B*35:07	898	906	FAMQMAYRF	0.7448
B07	HLA-B*35:07	1052	1062	FPQSAPHGVVF	0.8504
B07	HLA-B*35:07	1054	1062	QSAPHGVVF	0.6307
B07	HLA-B*35:07	1095	1103	FVSNGTHWF	0.5997
B07	HLA-B*35:08	24	32	LPPAYTNSF	0.797
B07	HLA-B*35:08	56	64	LPFFSNVTW	0.7097
B07	HLA-B*35:08	84	91	LPFNDGVY	0.6312
B07	HLA-B*35:08	84	92	LPFNDGVYF	0.9511
B07	HLA-B*35:08	162	170	SANNCTFEY	0.5112
B07	HLA-B*35:08	229	238	LPIGINITRF	0.6418
B07	HLA-B*35:08	321	329	QPTESIVRF	0.8721
B07	HLA-B*35:08	343	351	NATRFASVY	0.5359
B07	HLA-B*35:08	664	674	IPIGAGICASY	0.6771
B07	HLA-B*35:08	687	695	VASQSIIAY	0.8861
B07	HLA-B*35:08	699	707	LGAENSVAY	0.6356
B07	HLA-B*35:08	861	869	LPPLLTDEM	0.5504
B07	HLA-B*35:08	896	904	IPFAMQMAY	0.9572
B07	HLA-B*35:08	1052	1062	FPQSAPHGVVF	0.787
B07	HLA-B*35:11	24	32	LPPAYTNSF	0.8352
B07	HLA-B*35:11	30	38	NSFTRGVYY	0.7008
B07	HLA-B*35:11	56	64	LPFFSNVTW	0.6542
B07	HLA-B*35:11	83	92	VLPFNDGVYF	0.6879
B07	HLA-B*35:11	84	91	LPFNDGVY	0.8263
B07	HLA-B*35:11	84	92	LPFNDGVYF	0.9718
B07	HLA-B*35:11	160	168	YSSANNCTF	0.5303
B07	HLA-B*35:11	162	170	SANNCTFEY	0.5536
B07	HLA-B*35:11	192	200	FVFKNIDGY	0.7481
B07	HLA-B*35:11	212	220	LVRDLPQGF	0.575
B07	HLA-B*35:11	229	238	LPIGINITRF	0.775
B07	HLA-B*35:11	258	266	WTAGAAAYY	0.5592
B07	HLA-B*35:11	261	269	GAAAYYVGY	0.5071
B07	HLA-B*35:11	271	279	QPRTFLLKY	0.8033
B07	HLA-B*35:11	321	329	QPTESIVRF	0.879

B07	HLA-B*35:11	343	351	NATRFASVY	0.812
B07	HLA-B*35:11	366	374	SVLYNSASF	0.7009
B07	HLA-B*35:11	604	612	TSNQVAVLY	0.5291
B07	HLA-B*35:11	664	674	IPIGAGICASY	0.8485
B07	HLA-B*35:11	686	695	SVASQSIIAY	0.5827
B07	HLA-B*35:11	687	695	VASQSIIAY	0.9537
B07	HLA-B*35:11	699	707	LGAENSVAY	0.8145
B07	HLA-B*35:11	710	718	NSIAIPTNF	0.608
B07	HLA-B*35:11	865	873	LTDEMIAQY	0.5297
B07	HLA-B*35:11	869	877	MIAQYTSAL	0.5203
B07	HLA-B*35:11	886	894	WTFGAGAAL	0.5144
B07	HLA-B*35:11	892	900	AALQIPFAM	0.6266
B07	HLA-B*35:11	895	904	QIPFAMQMAY	0.6561
B07	HLA-B*35:11	896	904	IPFAMQMAY	0.9842
B07	HLA-B*35:11	898	906	FAMQMAYRF	0.6952
B07	HLA-B*35:11	1021	1029	SANLAATKM	0.6657
B07	HLA-B*35:11	1052	1062	FPQSAPHGVVF	0.7606
B07	HLA-B*35:11	1054	1062	QSAPHGVVF	0.7902
B07	HLA-B*35:11	1095	1103	FVSNGTHWF	0.6488
B07	HLA-B*35:11	1113	1121	QIITTDNTF	0.5819
B07	HLA-B*35:14	24	32	LPPAYTNSF	0.7489
B07	HLA-B*35:14	84	91	LPFNDGVY	0.6173
B07	HLA-B*35:14	84	92	LPFNDGVYF	0.8767
B07	HLA-B*35:14	192	200	FVFKNIDGY	0.677
B07	HLA-B*35:14	229	238	LPIGINITRF	0.6057
B07	HLA-B*35:14	258	266	WTAGAAAYY	0.5528
B07	HLA-B*35:14	343	351	NATRFASVY	0.7039
B07	HLA-B*35:14	366	374	SVLYNSASF	0.5417
B07	HLA-B*35:14	664	674	IPIGAGICASY	0.6706
B07	HLA-B*35:14	686	695	SVASQSIIAY	0.5487
B07	HLA-B*35:14	687	695	VASQSIIAY	0.8589
B07	HLA-B*35:14	699	707	LGAENSVAY	0.6846
B07	HLA-B*35:14	896	904	IPFAMQMAY	0.9083
B07	HLA-B*35:14	898	906	FAMQMAYRF	0.501
B07	HLA-B*35:14	1052	1062	FPQSAPHGVVF	0.5835
B07	HLA-B*35:14	1054	1062	QSAPHGVVF	0.6632
B07	HLA-B*35:14	1095	1103	FVSNGTHWF	0.5633
B07	HLA-B*35:15	24	32	LPPAYTNSF	0.7919
B07	HLA-B*35:15	56	64	LPFFSNVTW	0.7224
B07	HLA-B*35:15	83	92	VLPFNDGVYF	0.6829
B07	HLA-B*35:15	84	91	LPFNDGVY	0.7275
B07	HLA-B*35:15	84	92	LPFNDGVYF	0.9676
B07	HLA-B*35:15	162	170	SANNCTFEY	0.5711
B07	HLA-B*35:15	192	200	FVFKNIDGY	0.5935
B07	HLA-B*35:15	229	238	LPIGINITRF	0.7637
B07	HLA-B*35:15	321	329	QPTESIVRF	0.8872
B07	HLA-B*35:15	343	351	NATRFASVY	0.5685
B07	HLA-B*35:15	664	674	IPIGAGICASY	0.7825
B07	HLA-B*35:15	687	695	VASQSIIAY	0.9029
B07	HLA-B*35:15	699	707	LGAENSVAY	0.6765

B07	HLA-B*35:15	714	722	IPTNFTISV	0.5146
B07	HLA-B*35:15	861	869	LPPLLTDEM	0.5696
B07	HLA-B*35:15	892	900	AALQIPFAM	0.6734
B07	HLA-B*35:15	895	904	QIPFAMQMAY	0.5998
B07	HLA-B*35:15	896	904	IPFAMQMAY	0.9714
B07	HLA-B*35:15	898	906	FAMQMAYRF	0.665
B07	HLA-B*35:15	1052	1062	FPQSAPHGVVF	0.781
B07	HLA-B*35:15	1054	1062	QSAPHGVVF	0.5581
B07	HLA-B*35:21	24	32	LPPAYTNSF	0.8395
B07	HLA-B*35:21	30	38	NSFTRGVYY	0.7343
B07	HLA-B*35:21	56	64	LPFFSNVTW	0.7008
B07	HLA-B*35:21	83	92	VLPFNDGVYF	0.6004
B07	HLA-B*35:21	84	91	LPFNDGVY	0.8356
B07	HLA-B*35:21	84	92	LPFNDGVYF	0.9541
B07	HLA-B*35:21	138	145	DPFLGVYY	0.7423
B07	HLA-B*35:21	192	200	FVFKNIDGY	0.6843
B07	HLA-B*35:21	229	238	LPIGINITRF	0.7683
B07	HLA-B*35:21	271	279	QPRTFLLKY	0.8503
B07	HLA-B*35:21	321	329	QPTESIVRF	0.8652
B07	HLA-B*35:21	343	351	NATRFASVY	0.817
B07	HLA-B*35:21	366	374	SVLYNSASF	0.6264
B07	HLA-B*35:21	487	495	NCYFPLQSY	0.57
B07	HLA-B*35:21	490	497	FPLQSYGF	0.5592
B07	HLA-B*35:21	664	674	IPIGAGICASY	0.8085
B07	HLA-B*35:21	686	695	SVASQSIIAY	0.5007
B07	HLA-B*35:21	687	695	VASQSIIAY	0.9249
B07	HLA-B*35:21	699	707	LGAENSVAY	0.7697
B07	HLA-B*35:21	869	877	MIAQYTSAL	0.5073
B07	HLA-B*35:21	892	900	AALQIPFAM	0.5807
B07	HLA-B*35:21	895	904	QIPFAMQMAY	0.688
B07	HLA-B*35:21	896	904	IPFAMQMAY	0.9842
B07	HLA-B*35:21	897	904	PFAMQMAY	0.6134
B07	HLA-B*35:21	898	906	FAMQMAYRF	0.7194
B07	HLA-B*35:21	1021	1029	SANLAATKM	0.5645
B07	HLA-B*35:21	1052	1062	FPQSAPHGVVF	0.7764
B07	HLA-B*35:21	1054	1062	QSAPHGVVF	0.7008
B07	HLA-B*35:21	1095	1103	FVSNGTHWF	0.5717
B07	HLA-B*35:21	1130	1138	IGIVNNTVY	0.5011
B07	HLA-B*35:22	24	32	LPPAYTNSF	0.6758
B07	HLA-B*35:22	84	92	LPFNDGVYF	0.8301
B07	HLA-B*35:22	216	223	LPQGFSAL	0.5263
B07	HLA-B*35:22	714	722	IPTNFTISV	0.8019
B07	HLA-B*35:22	861	869	LPPLLTDEM	0.6575
B07	HLA-B*35:22	896	904	IPFAMQMAY	0.568
B07	HLA-B*35:22	1052	1060	FPQSAPHGV	0.7661
B07	HLA-B*35:22	1052	1062	FPQSAPHGVVF	0.6166
B07	HLA-B*35:24	24	32	LPPAYTNSF	0.8659
B07	HLA-B*35:24	30	38	NSFTRGVYY	0.6
B07	HLA-B*35:24	56	64	LPFFSNVTW	0.8189
B07	HLA-B*35:24	56	65	LPFFSNVTWF	0.517

B07	HLA-B*35:24	83	92	VLPFNDGVYF	0.6239
B07	HLA-B*35:24	84	91	LPFNDGVY	0.8242
B07	HLA-B*35:24	84	92	LPFNDGVYF	0.9759
B07	HLA-B*35:24	138	145	DPFLGVYY	0.7649
B07	HLA-B*35:24	138	146	DPFLGVYYH	0.6062
B07	HLA-B*35:24	162	170	SANNCTFEY	0.5434
B07	HLA-B*35:24	192	200	FVFKNIDGY	0.6882
B07	HLA-B*35:24	229	238	LPIGINITRF	0.8081
B07	HLA-B*35:24	271	279	QPRTFLLKY	0.7209
B07	HLA-B*35:24	321	329	QPTESIVRF	0.9142
B07	HLA-B*35:24	343	351	NATRFASVY	0.7681
B07	HLA-B*35:24	490	497	FPLQSYGF	0.6001
B07	HLA-B*35:24	604	612	TSNQVAVLY	0.5175
B07	HLA-B*35:24	625	633	HADQLTPTW	0.6622
B07	HLA-B*35:24	664	674	IPIGAGICASY	0.7856
B07	HLA-B*35:24	687	695	VASQSIIAY	0.9318
B07	HLA-B*35:24	699	707	LGAENSVAY	0.7685
B07	HLA-B*35:24	714	722	IPTNFTISV	0.5262
B07	HLA-B*35:24	861	869	LPPLLTDEM	0.5723
B07	HLA-B*35:24	865	873	LTDEMIAQY	0.5411
B07	HLA-B*35:24	892	900	AALQIPFAM	0.5331
B07	HLA-B*35:24	895	904	QIPFAMQMAY	0.6479
B07	HLA-B*35:24	896	904	IPFAMQMAY	0.987
B07	HLA-B*35:24	897	904	PFAMQMAY	0.5448
B07	HLA-B*35:24	898	906	FAMQMAYRF	0.7695
B07	HLA-B*35:24	1052	1062	FPQSAPHGVVF	0.8489
B07	HLA-B*35:24	1054	1062	QSAPHGVVF	0.5377
B07	HLA-B*35:24	1095	1103	FVSNGTHWF	0.5268
B07	HLA-B*35:31	38	48	YPDKVFRSSVL	0.5154
B07	HLA-B*35:31	84	92	LPFNDGVYF	0.7397
B07	HLA-B*35:31	216	223	LPQGFSAL	0.5549
B07	HLA-B*35:31	714	722	IPTNFTISV	0.825
B07	HLA-B*35:31	861	869	LPPLLTDEM	0.5885
B07	HLA-B*35:31	892	900	AALQIPFAM	0.5669
B07	HLA-B*35:31	1052	1060	FPQSAPHGV	0.7222
B07	HLA-B*35:31	1052	1062	FPQSAPHGVVF	0.5623
B07	HLA-B*35:32	24	32	LPPAYTNSF	0.9052
B07	HLA-B*35:32	30	38	NSFTRGVYY	0.5702
B07	HLA-B*35:32	56	64	LPFFSNVTW	0.8087
B07	HLA-B*35:32	56	65	LPFFSNVTWF	0.5164
B07	HLA-B*35:32	83	92	VLPFNDGVYF	0.7561
B07	HLA-B*35:32	84	91	LPFNDGVY	0.8314
B07	HLA-B*35:32	84	92	LPFNDGVYF	0.9881
B07	HLA-B*35:32	160	168	YSSANNCTF	0.5049
B07	HLA-B*35:32	162	170	SANNCTFEY	0.7098
B07	HLA-B*35:32	192	200	FVFKNIDGY	0.8032
B07	HLA-B*35:32	229	238	LPIGINITRF	0.8498
B07	HLA-B*35:32	258	266	WTAGAAAYY	0.5683
B07	HLA-B*35:32	271	279	QPRTFLLKY	0.6724
B07	HLA-B*35:32	321	329	QPTESIVRF	0.9486

B07	HLA-B*35:32	343	351	NATRFASVY	0.7659
B07	HLA-B*35:32	361	369	CVADYSVLY	0.53
B07	HLA-B*35:32	478	486	TPCNGVEGF	0.6358
B07	HLA-B*35:32	490	497	FPLQSYGF	0.5082
B07	HLA-B*35:32	604	612	TSNQVAVLY	0.5842
B07	HLA-B*35:32	625	633	HADQLTPTW	0.7442
B07	HLA-B*35:32	664	674	IPIGAGICASY	0.8564
B07	HLA-B*35:32	686	695	SVASQSIIAY	0.5225
B07	HLA-B*35:32	687	695	VASQSIIAY	0.9698
B07	HLA-B*35:32	699	707	LGAENSVAY	0.863
B07	HLA-B*35:32	710	718	NSIAIPTNF	0.5244
B07	HLA-B*35:32	714	722	IPTNFTISV	0.5438
B07	HLA-B*35:32	861	869	LPPLLTDEM	0.6652
B07	HLA-B*35:32	865	873	LTDEMIAQY	0.6517
B07	HLA-B*35:32	892	900	AALQIPFAM	0.6373
B07	HLA-B*35:32	895	904	QIPFAMQMAY	0.599
B07	HLA-B*35:32	896	904	IPFAMQMAY	0.9853
B07	HLA-B*35:32	898	906	FAMQMAYRF	0.7754
B07	HLA-B*35:32	1021	1029	SANLAATKM	0.5136
B07	HLA-B*35:32	1052	1060	FPQSAPHGV	0.5544
B07	HLA-B*35:32	1052	1062	FPQSAPHGVVF	0.8763
B07	HLA-B*35:32	1054	1062	QSAPHGVVF	0.697
B07	HLA-B*35:32	1095	1103	FVSNGTHWF	0.6496
B07	HLA-B*35:32	1113	1121	QIITTDNTF	0.5211
B07	HLA-B*35:33	714	722	IPTNFTISV	0.6955
B07	HLA-B*35:33	861	869	LPPLLTDEM	0.589
B07	HLA-B*35:35	24	32	LPPAYTNSF	0.8931
B07	HLA-B*35:35	30	38	NSFTRGVYY	0.6432
B07	HLA-B*35:35	38	46	YPDKVFRSS	0.5022
B07	HLA-B*35:35	56	64	LPFFSNVTW	0.8081
B07	HLA-B*35:35	56	65	LPFFSNVTWF	0.5997
B07	HLA-B*35:35	81	92	NPVLPFNDGVYF	0.5813
B07	HLA-B*35:35	83	92	VLPFNDGVYF	0.7798
B07	HLA-B*35:35	84	91	LPFNDGVY	0.8427
B07	HLA-B*35:35	84	92	LPFNDGVYF	0.987
B07	HLA-B*35:35	160	168	YSSANNCTF	0.5537
B07	HLA-B*35:35	162	170	SANNCTFEY	0.7379
B07	HLA-B*35:35	192	200	FVFKNIDGY	0.7847
B07	HLA-B*35:35	196	204	NIDGYFKIY	0.5682
B07	HLA-B*35:35	229	238	LPIGINITRF	0.8518
B07	HLA-B*35:35	258	266	WTAGAAAYY	0.6006
B07	HLA-B*35:35	261	269	GAAAYYVGY	0.5655
B07	HLA-B*35:35	271	279	QPRTFLLKY	0.7481
B07	HLA-B*35:35	321	329	QPTESIVRF	0.9497
B07	HLA-B*35:35	329	338	FPNITNLCPF	0.5517
B07	HLA-B*35:35	343	351	NATRFASVY	0.791
B07	HLA-B*35:35	361	369	CVADYSVLY	0.579
B07	HLA-B*35:35	366	374	SVLYNSASF	0.6081
B07	HLA-B*35:35	392	400	FTNVYADSF	0.5023
B07	HLA-B*35:35	462	473	KPFERDISTEIY	0.6963

B07	HLA-B*35:35	478	486	TPCNGVEGF	0.6736
B07	HLA-B*35:35	490	497	FPLQSYGF	0.6408
B07	HLA-B*35:35	604	612	TSNQVAVLY	0.653
B07	HLA-B*35:35	625	633	HADQLTPTW	0.77
B07	HLA-B*35:35	652	660	GAEHVNNSY	0.6983
B07	HLA-B*35:35	664	674	IPIGAGICASY	0.8472
B07	HLA-B*35:35	686	695	SVASQSIIAY	0.5942
B07	HLA-B*35:35	687	695	VASQSIIAY	0.9671
B07	HLA-B*35:35	699	707	LGAENSVAY	0.8503
B07	HLA-B*35:35	710	718	NSIAIPTNF	0.6147
B07	HLA-B*35:35	714	722	IPTNFTISV	0.6668
B07	HLA-B*35:35	861	869	LPPLLTDEM	0.6989
B07	HLA-B*35:35	865	873	LTDEMIAQY	0.7051
B07	HLA-B*35:35	869	877	MIAQYTSAL	0.5063
B07	HLA-B*35:35	886	894	WTFGAGAAL	0.5733
B07	HLA-B*35:35	892	900	AALQIPFAM	0.7769
B07	HLA-B*35:35	895	904	QIPFAMQMAY	0.6833
B07	HLA-B*35:35	896	904	IPFAMQMAY	0.9858
B07	HLA-B*35:35	897	904	PFAMQMAY	0.5282
B07	HLA-B*35:35	898	906	FAMQMAYRF	0.8293
B07	HLA-B*35:35	1021	1029	SANLAATKM	0.6342
B07	HLA-B*35:35	1052	1060	FPQSAPHGV	0.6179
B07	HLA-B*35:35	1052	1062	FPQSAPHGVVF	0.8964
B07	HLA-B*35:35	1054	1062	QSAPHGVVF	0.7525
B07	HLA-B*35:35	1056	1067	APHGVVFLHVTY	0.6046
B07	HLA-B*35:35	1089	1097	FPREGVFVS	0.5258
B07	HLA-B*35:35	1095	1103	FVSNGTHWF	0.7045
B07	HLA-B*35:35	1113	1121	QIITTDNTF	0.592
B07	HLA-B*35:36	84	92	LPFNDGVYF	0.8449
B07	HLA-B*35:36	714	722	IPTNFTISV	0.6549
B07	HLA-B*35:36	861	869	LPPLLTDEM	0.674
B07	HLA-B*35:36	1052	1062	FPQSAPHGVVF	0.6557
B07	HLA-B*35:38	1052	1062	FPQSAPHGVVF	0.6186
B07	HLA-B*35:41	24	32	LPPAYTNSF	0.9203
B07	HLA-B*35:41	30	38	NSFTRGVYY	0.69
B07	HLA-B*35:41	56	64	LPFFSNVTW	0.8713
B07	HLA-B*35:41	56	65	LPFFSNVTWF	0.6188
B07	HLA-B*35:41	81	92	NPVLPFNDGVYF	0.5272
B07	HLA-B*35:41	83	92	VLPFNDGVYF	0.7855
B07	HLA-B*35:41	84	91	LPFNDGVY	0.8727
B07	HLA-B*35:41	84	92	LPFNDGVYF	0.9927
B07	HLA-B*35:41	160	168	YSSANNCTF	0.5877
B07	HLA-B*35:41	162	170	SANNCTFEY	0.7881
B07	HLA-B*35:41	192	200	FVFKNIDGY	0.8905
B07	HLA-B*35:41	196	204	NIDGYFKIY	0.577
B07	HLA-B*35:41	229	238	LPIGINITRF	0.8935
B07	HLA-B*35:41	258	266	WTAGAAAYY	0.7113
B07	HLA-B*35:41	261	269	GAAAYYVGY	0.5904
B07	HLA-B*35:41	271	279	QPRTFLLKY	0.731
B07	HLA-B*35:41	321	329	QPTESIVRF	0.9644

B07	HLA-B*35:41	329	338	FPNITNLCPF	0.5798
B07	HLA-B*35:41	343	351	NATRFASVY	0.8709
B07	HLA-B*35:41	361	369	CVADYSVLY	0.6962
B07	HLA-B*35:41	366	374	SVLYNSASF	0.6032
B07	HLA-B*35:41	392	400	FTNVYADSF	0.5373
B07	HLA-B*35:41	462	473	KPFERDISTEIY	0.6461
B07	HLA-B*35:41	478	486	TPCNGVEGF	0.6721
B07	HLA-B*35:41	481	489	NGVEGFNCY	0.5089
B07	HLA-B*35:41	490	497	FPLQSYGF	0.5518
B07	HLA-B*35:41	604	612	TSNQVAVLY	0.7229
B07	HLA-B*35:41	625	633	HADQLTPTW	0.7962
B07	HLA-B*35:41	652	660	GAEHVNNSY	0.6893
B07	HLA-B*35:41	664	674	IPIGAGICASY	0.908
B07	HLA-B*35:41	686	695	SVASQSIIAY	0.6499
B07	HLA-B*35:41	687	695	VASQSIIAY	0.9842
B07	HLA-B*35:41	699	707	LGAENSVAY	0.9146
B07	HLA-B*35:41	710	718	NSIAIPTNF	0.6291
B07	HLA-B*35:41	714	722	IPTNFTISV	0.6147
B07	HLA-B*35:41	861	869	LPPLLTDEM	0.695
B07	HLA-B*35:41	865	873	LTDEMIAQY	0.7508
B07	HLA-B*35:41	892	900	AALQIPFAM	0.7116
B07	HLA-B*35:41	895	904	QIPFAMQMAY	0.7205
B07	HLA-B*35:41	896	904	IPFAMQMAY	0.993
B07	HLA-B*35:41	897	904	PFAMQMAY	0.5108
B07	HLA-B*35:41	898	906	FAMQMAYRF	0.8561
B07	HLA-B*35:41	1021	1029	SANLAATKM	0.6172
B07	HLA-B*35:41	1052	1060	FPQSAPHGV	0.5784
B07	HLA-B*35:41	1052	1062	FPQSAPHGVVF	0.8981
B07	HLA-B*35:41	1054	1062	QSAPHGVVF	0.7647
B07	HLA-B*35:41	1056	1067	APHGVVFLHVTY	0.5536
B07	HLA-B*35:41	1089	1097	FPREGVFVS	0.5633
B07	HLA-B*35:41	1095	1103	FVSNGTHWF	0.7432
B07	HLA-B*35:41	1113	1121	QIITTDNTF	0.6101
B07	HLA-B*35:41	1130	1138	IGIVNNTVY	0.512
B07	HLA-B*35:42	24	32	LPPAYTNSF	0.8786
B07	HLA-B*35:42	30	38	NSFTRGVYY	0.5335
B07	HLA-B*35:42	56	64	LPFFSNVTW	0.8069
B07	HLA-B*35:42	83	92	VLPFNDGVYF	0.7228
B07	HLA-B*35:42	84	91	LPFNDGVY	0.824
B07	HLA-B*35:42	84	92	LPFNDGVYF	0.9877
B07	HLA-B*35:42	162	170	SANNCTFEY	0.6698
B07	HLA-B*35:42	192	200	FVFKNIDGY	0.7837
B07	HLA-B*35:42	229	238	LPIGINITRF	0.8394
B07	HLA-B*35:42	258	266	WTAGAAAYY	0.5393
B07	HLA-B*35:42	271	279	QPRTFLLKY	0.6565
B07	HLA-B*35:42	321	329	QPTESIVRF	0.9476
B07	HLA-B*35:42	343	351	NATRFASVY	0.7573
B07	HLA-B*35:42	361	369	CVADYSVLY	0.5167
B07	HLA-B*35:42	478	486	TPCNGVEGF	0.5961
B07	HLA-B*35:42	604	612	TSNQVAVLY	0.5492

B07	HLA-B*35:42	625	633	HADQLTPTW	0.7176
B07	HLA-B*35:42	664	674	IPIGAGICASY	0.8514
B07	HLA-B*35:42	687	695	VASQSIIAY	0.9634
B07	HLA-B*35:42	699	707	LGAENSVAY	0.8303
B07	HLA-B*35:42	714	722	IPTNFTISV	0.5007
B07	HLA-B*35:42	861	869	LPPLLTDEM	0.6186
B07	HLA-B*35:42	865	873	LTDEMIAQY	0.6118
B07	HLA-B*35:42	892	900	AALQIPFAM	0.5789
B07	HLA-B*35:42	895	904	QIPFAMQMAY	0.6098
B07	HLA-B*35:42	896	904	IPFAMQMAY	0.9872
B07	HLA-B*35:42	898	906	FAMQMAYRF	0.7448
B07	HLA-B*35:42	1052	1062	FPQSAPHGVVF	0.8504
B07	HLA-B*35:42	1054	1062	QSAPHGVVF	0.6307
B07	HLA-B*35:42	1095	1103	FVSNGTHWF	0.5997
B07	HLA-B*35:43	24	32	LPPAYTNSF	0.7875
B07	HLA-B*35:43	30	38	NSFTRGVYY	0.5895
B07	HLA-B*35:43	84	91	LPFNDGVY	0.6447
B07	HLA-B*35:43	84	92	LPFNDGVYF	0.8888
B07	HLA-B*35:43	160	168	YSSANNCTF	0.5206
B07	HLA-B*35:43	192	200	FVFKNIDGY	0.7054
B07	HLA-B*35:43	229	238	LPIGINITRF	0.6427
B07	HLA-B*35:43	258	266	WTAGAAAYY	0.5829
B07	HLA-B*35:43	343	351	NATRFASVY	0.7189
B07	HLA-B*35:43	366	374	SVLYNSASF	0.6027
B07	HLA-B*35:43	664	674	IPIGAGICASY	0.6933
B07	HLA-B*35:43	686	695	SVASQSIIAY	0.5892
B07	HLA-B*35:43	687	695	VASQSIIAY	0.8891
B07	HLA-B*35:43	699	707	LGAENSVAY	0.7466
B07	HLA-B*35:43	896	904	IPFAMQMAY	0.9166
B07	HLA-B*35:43	898	906	FAMQMAYRF	0.543
B07	HLA-B*35:43	962	970	LVKQLSSNF	0.5124
B07	HLA-B*35:43	1052	1062	FPQSAPHGVVF	0.6324
B07	HLA-B*35:43	1054	1062	QSAPHGVVF	0.7377
B07	HLA-B*35:43	1095	1103	FVSNGTHWF	0.6086
B07	HLA-B*35:43	1113	1121	QIITTDNTF	0.5611
B07	HLA-B*35:45	84	92	LPFNDGVYF	0.7419
B07	HLA-B*35:45	687	695	VASQSIIAY	0.5885
B07	HLA-B*35:45	896	904	IPFAMQMAY	0.7957
B07	HLA-B*35:46	24	32	LPPAYTNSF	0.6364
B07	HLA-B*35:46	56	64	LPFFSNVTW	0.5165
B07	HLA-B*35:46	84	92	LPFNDGVYF	0.9096
B07	HLA-B*35:46	192	200	FVFKNIDGY	0.5961
B07	HLA-B*35:46	229	238	LPIGINITRF	0.5183
B07	HLA-B*35:46	321	329	QPTESIVRF	0.7199
B07	HLA-B*35:46	343	351	NATRFASVY	0.5076
B07	HLA-B*35:46	664	674	IPIGAGICASY	0.6105
B07	HLA-B*35:46	687	695	VASQSIIAY	0.8432
B07	HLA-B*35:46	699	707	LGAENSVAY	0.6048
B07	HLA-B*35:46	896	904	IPFAMQMAY	0.9239
B07	HLA-B*35:46	1052	1062	FPQSAPHGVVF	0.5441

B07	HLA-B*35:46	1054	1062	QSAPHGVVF	0.5022
B07	HLA-B*35:54	24	32	LPPAYTNSF	0.8786
B07	HLA-B*35:54	30	38	NSFTRGVYY	0.5335
B07	HLA-B*35:54	56	64	LPFFSNVTW	0.8069
B07	HLA-B*35:54	83	92	VLPFNDGVYF	0.7228
B07	HLA-B*35:54	84	91	LPFNDGVY	0.824
B07	HLA-B*35:54	84	92	LPFNDGVYF	0.9877
B07	HLA-B*35:54	162	170	SANNCTFEY	0.6698
B07	HLA-B*35:54	192	200	FVFKNIDGY	0.7837
B07	HLA-B*35:54	229	238	LPIGINITRF	0.8394
B07	HLA-B*35:54	258	266	WTAGAAAYY	0.5393
B07	HLA-B*35:54	271	279	QPRTFLLKY	0.6565
B07	HLA-B*35:54	321	329	QPTESIVRF	0.9476
B07	HLA-B*35:54	343	351	NATRFASVY	0.7573
B07	HLA-B*35:54	361	369	CVADYSVLY	0.5167
B07	HLA-B*35:54	478	486	TPCNGVEGF	0.5961
B07	HLA-B*35:54	604	612	TSNQVAVLY	0.5492
B07	HLA-B*35:54	625	633	HADQLTPTW	0.7176
B07	HLA-B*35:54	664	674	IPIGAGICASY	0.8514
B07	HLA-B*35:54	687	695	VASQSIIAY	0.9634
B07	HLA-B*35:54	699	707	LGAENSVAY	0.8303
B07	HLA-B*35:54	714	722	IPTNFTISV	0.5007
B07	HLA-B*35:54	861	869	LPPLLTDEM	0.6186
B07	HLA-B*35:54	865	873	LTDEMIAQY	0.6118
B07	HLA-B*35:54	892	900	AALQIPFAM	0.5789
B07	HLA-B*35:54	895	904	QIPFAMQMAY	0.6098
B07	HLA-B*35:54	896	904	IPFAMQMAY	0.9872
B07	HLA-B*35:54	898	906	FAMQMAYRF	0.7448
B07	HLA-B*35:54	1052	1062	FPQSAPHGVVF	0.8504
B07	HLA-B*35:54	1054	1062	QSAPHGVVF	0.6307
B07	HLA-B*35:54	1095	1103	FVSNGTHWF	0.5997
B07	HLA-B*35:55	84	92	LPFNDGVYF	0.8449
B07	HLA-B*35:55	714	722	IPTNFTISV	0.6549
B07	HLA-B*35:55	861	869	LPPLLTDEM	0.674
B07	HLA-B*35:55	1052	1062	FPQSAPHGVVF	0.6557
B07	HLA-B*35:57	24	32	LPPAYTNSF	0.8786
B07	HLA-B*35:57	30	38	NSFTRGVYY	0.5335
B07	HLA-B*35:57	56	64	LPFFSNVTW	0.8069
B07	HLA-B*35:57	83	92	VLPFNDGVYF	0.7228
B07	HLA-B*35:57	84	91	LPFNDGVY	0.824
B07	HLA-B*35:57	84	92	LPFNDGVYF	0.9877
B07	HLA-B*35:57	162	170	SANNCTFEY	0.6698
B07	HLA-B*35:57	192	200	FVFKNIDGY	0.7837
B07	HLA-B*35:57	229	238	LPIGINITRF	0.8394
B07	HLA-B*35:57	258	266	WTAGAAAYY	0.5393
B07	HLA-B*35:57	271	279	QPRTFLLKY	0.6565
B07	HLA-B*35:57	321	329	QPTESIVRF	0.9476
B07	HLA-B*35:57	343	351	NATRFASVY	0.7573
B07	HLA-B*35:57	361	369	CVADYSVLY	0.5167
B07	HLA-B*35:57	478	486	TPCNGVEGF	0.5961

B07	HLA-B*35:57	604	612	TSNQVAVLY	0.5492
B07	HLA-B*35:57	625	633	HADQLTPTW	0.7176
B07	HLA-B*35:57	664	674	IPIGAGICASY	0.8514
B07	HLA-B*35:57	687	695	VASQSIIAY	0.9634
B07	HLA-B*35:57	699	707	LGAENSVAY	0.8303
B07	HLA-B*35:57	714	722	IPTNFTISV	0.5007
B07	HLA-B*35:57	861	869	LPPLLTDEM	0.6186
B07	HLA-B*35:57	865	873	LTDEMIAQY	0.6118
B07	HLA-B*35:57	892	900	AALQIPFAM	0.5789
B07	HLA-B*35:57	895	904	QIPFAMQMAY	0.6098
B07	HLA-B*35:57	896	904	IPFAMQMAY	0.9872
B07	HLA-B*35:57	898	906	FAMQMAYRF	0.7448
B07	HLA-B*35:57	1052	1062	FPQSAPHGVVF	0.8504
B07	HLA-B*35:57	1054	1062	QSAPHGVVF	0.6307
B07	HLA-B*35:57	1095	1103	FVSNGTHWF	0.5997
B07	HLA-B*35:61	24	32	LPPAYTNSF	0.797
B07	HLA-B*35:61	56	64	LPFFSNVTW	0.7097
B07	HLA-B*35:61	84	91	LPFNDGVY	0.6312
B07	HLA-B*35:61	84	92	LPFNDGVYF	0.9511
B07	HLA-B*35:61	162	170	SANNCTFEY	0.5112
B07	HLA-B*35:61	229	238	LPIGINITRF	0.6418
B07	HLA-B*35:61	321	329	QPTESIVRF	0.8721
B07	HLA-B*35:61	343	351	NATRFASVY	0.5359
B07	HLA-B*35:61	664	674	IPIGAGICASY	0.6771
B07	HLA-B*35:61	687	695	VASQSIIAY	0.8861
B07	HLA-B*35:61	699	707	LGAENSVAY	0.6356
B07	HLA-B*35:61	861	869	LPPLLTDEM	0.5504
B07	HLA-B*35:61	896	904	IPFAMQMAY	0.9572
B07	HLA-B*35:61	1052	1062	FPQSAPHGVVF	0.787
B07	HLA-B*39:10	24	32	LPPAYTNSF	0.7059
B07	HLA-B*39:10	38	47	YPDKVFRSSV	0.6561
B07	HLA-B*39:10	38	48	YPDKVFRSSVL	0.6644
B07	HLA-B*39:10	84	92	LPFNDGVYF	0.7911
B07	HLA-B*39:10	208	216	TPINLVRDL	0.8013
B07	HLA-B*39:10	216	223	LPQGFSAL	0.7184
B07	HLA-B*39:10	229	238	LPIGINITRF	0.5061
B07	HLA-B*39:10	506	513	QPYRVVVL	0.6651
B07	HLA-B*39:10	714	722	IPTNFTISV	0.8392
B07	HLA-B*39:10	861	869	LPPLLTDEM	0.6235
B07	HLA-B*39:10	869	877	MIAQYTSAL	0.6143
B07	HLA-B*39:10	1052	1060	FPQSAPHGV	0.8631
B07	HLA-B*39:10	1052	1061	FPQSAPHGVV	0.603
B07	HLA-B*39:10	1052	1062	FPQSAPHGVVF	0.7108
B07	HLA-B*39:10	1262	1270	EPVLKGVKL	0.8325
B07	HLA-B*39:16	24	32	LPPAYTNSF	0.7059
B07	HLA-B*39:16	38	47	YPDKVFRSSV	0.6561
B07	HLA-B*39:16	38	48	YPDKVFRSSVL	0.6644
B07	HLA-B*39:16	84	92	LPFNDGVYF	0.7911
B07	HLA-B*39:16	208	216	TPINLVRDL	0.8013
B07	HLA-B*39:16	216	223	LPQGFSAL	0.7184

B07	HLA-B*39:16	229	238	LPIGINITRF	0.5061
B07	HLA-B*39:16	506	513	QPYRVVVL	0.6651
B07	HLA-B*39:16	714	722	IPTNFTISV	0.8392
B07	HLA-B*39:16	861	869	LPPLLTDEM	0.6235
B07	HLA-B*39:16	869	877	MIAQYTSAL	0.6143
B07	HLA-B*39:16	1052	1060	FPQSAPHGV	0.8631
B07	HLA-B*39:16	1052	1061	FPQSAPHGVV	0.603
B07	HLA-B*39:16	1052	1062	FPQSAPHGVVF	0.7108
B07	HLA-B*39:16	1262	1270	EPVLKGVKL	0.8325
B07	HLA-B*42:01	24	32	LPPAYTNSF	0.7878
B07	HLA-B*42:01	38	47	YPDKVFRSSV	0.6536
B07	HLA-B*42:01	84	92	LPFNDGVYF	0.7423
B07	HLA-B*42:01	208	216	TPINLVRDL	0.869
B07	HLA-B*42:01	216	223	LPQGFSAL	0.7808
B07	HLA-B*42:01	229	238	LPIGINITRF	0.5486
B07	HLA-B*42:01	506	513	QPYRVVVL	0.8243
B07	HLA-B*42:01	526	534	GPKKSTNLV	0.6419
B07	HLA-B*42:01	620	629	VPVAIHADQL	0.633
B07	HLA-B*42:01	714	722	IPTNFTISV	0.8435
B07	HLA-B*42:01	869	877	MIAQYTSAL	0.5968
B07	HLA-B*42:01	1052	1060	FPQSAPHGV	0.7467
B07	HLA-B*42:01	1052	1062	FPQSAPHGVVF	0.6491
B07	HLA-B*42:01	1056	1063	APHGVVFL	0.7492
B07	HLA-B*42:01	1261	1270	SEPVLKGVKL	0.7228
B07	HLA-B*42:01	1262	1270	EPVLKGVKL	0.831
B07	HLA-B*42:04	38	47	YPDKVFRSSV	0.7244
B07	HLA-B*42:04	208	216	TPINLVRDL	0.7004
B07	HLA-B*42:04	216	223	LPQGFSAL	0.6226
B07	HLA-B*42:04	462	470	KPFERDIST	0.5873
B07	HLA-B*42:04	506	513	QPYRVVVL	0.6967
B07	HLA-B*42:04	526	534	GPKKSTNLV	0.662
B07	HLA-B*42:04	588	597	TPCSFGGVSV	0.5412
B07	HLA-B*42:04	664	672	IPIGAGICA	0.5648
B07	HLA-B*42:04	680	688	SPRRARSVA	0.6117
B07	HLA-B*42:04	714	722	IPTNFTISV	0.8825
B07	HLA-B*42:04	1052	1060	FPQSAPHGV	0.8239
B07	HLA-B*42:04	1052	1061	FPQSAPHGVV	0.6478
B07	HLA-B*42:04	1089	1096	FPREGVFV	0.655
B07	HLA-B*42:04	1089	1097	FPREGVFVS	0.5819
B07	HLA-B*42:05	24	32	LPPAYTNSF	0.7878
B07	HLA-B*42:05	38	47	YPDKVFRSSV	0.6536
B07	HLA-B*42:05	84	92	LPFNDGVYF	0.7423
B07	HLA-B*42:05	208	216	TPINLVRDL	0.869
B07	HLA-B*42:05	216	223	LPQGFSAL	0.7808
B07	HLA-B*42:05	229	238	LPIGINITRF	0.5486
B07	HLA-B*42:05	506	513	QPYRVVVL	0.8243
B07	HLA-B*42:05	526	534	GPKKSTNLV	0.6419
B07	HLA-B*42:05	620	629	VPVAIHADQL	0.633
B07	HLA-B*42:05	714	722	IPTNFTISV	0.8435
B07	HLA-B*42:05	869	877	MIAQYTSAL	0.5968

B07	HLA-B*42:05	1052	1060	FPQSAPHGV	0.7467
B07	HLA-B*42:05	1052	1062	FPQSAPHGVVF	0.6491
B07	HLA-B*42:05	1056	1063	APHGVVFL	0.7492
B07	HLA-B*42:05	1261	1270	SEPVLKGVKL	0.7228
B07	HLA-B*42:05	1262	1270	EPVLKGVKL	0.831
B07	HLA-B*51:01	712	720	IAIPTNFTI	0.8936
B07	HLA-B*51:01	714	722	IPTNFTISV	0.9219
B07	HLA-B*51:02	712	720	IAIPTNFTI	0.9071
B07	HLA-B*51:02	714	722	IPTNFTISV	0.9166
B07	HLA-B*51:02	923	931	IANQFNSAI	0.7018
B07	HLA-B*51:02	1052	1060	FPQSAPHGV	0.7998
B07	HLA-B*51:02	1052	1061	FPQSAPHGVV	0.6058
B07	HLA-B*51:05	425	434	LPDDFTGCVI	0.5315
B07	HLA-B*51:09	712	720	IAIPTNFTI	0.7984
B07	HLA-B*51:09	714	722	IPTNFTISV	0.7885
B07	HLA-B*51:16	712	720	IAIPTNFTI	0.8496
B07	HLA-B*51:16	714	722	IPTNFTISV	0.882
B07	HLA-B*51:17	712	720	IAIPTNFTI	0.8936
B07	HLA-B*51:17	714	722	IPTNFTISV	0.9219
B07	HLA-B*51:18	712	720	IAIPTNFTI	0.8936
B07	HLA-B*51:18	714	722	IPTNFTISV	0.9219
B07	HLA-B*51:19	56	64	LPFFSNVTW	0.5994
B07	HLA-B*51:19	84	92	LPFNDGVYF	0.7163
B07	HLA-B*51:19	712	720	IAIPTNFTI	0.8629
B07	HLA-B*51:19	714	722	IPTNFTISV	0.8404
B07	HLA-B*51:21	712	720	IAIPTNFTI	0.8586
B07	HLA-B*51:21	714	722	IPTNFTISV	0.8761
B07	HLA-B*51:24	712	720	IAIPTNFTI	0.8936
B07	HLA-B*51:24	714	722	IPTNFTISV	0.9219
B07	HLA-B*51:26	712	720	IAIPTNFTI	0.8936
B07	HLA-B*51:26	714	722	IPTNFTISV	0.9219
B07	HLA-B*51:28	712	720	IAIPTNFTI	0.8936
B07	HLA-B*51:28	714	722	IPTNFTISV	0.9219
B07	HLA-B*51:29	425	434	LPDDFTGCVI	0.6074
B07	HLA-B*51:29	714	722	IPTNFTISV	0.8847
B07	HLA-B*51:30	712	720	IAIPTNFTI	0.8936
B07	HLA-B*51:30	714	722	IPTNFTISV	0.9219
B07	HLA-B*51:31	712	720	IAIPTNFTI	0.8324
B07	HLA-B*51:31	714	722	IPTNFTISV	0.8262
B07	HLA-B*51:32	712	720	IAIPTNFTI	0.8936
B07	HLA-B*51:32	714	722	IPTNFTISV	0.9219
B07	HLA-B*51:33	712	720	IAIPTNFTI	0.8936
B07	HLA-B*51:33	714	722	IPTNFTISV	0.9219
B07	HLA-B*51:34	712	720	IAIPTNFTI	0.8619
B07	HLA-B*51:34	714	722	IPTNFTISV	0.8627
B07	HLA-B*51:34	1052	1061	FPQSAPHGVV	0.5175
B07	HLA-B*51:35	712	720	IAIPTNFTI	0.8936
B07	HLA-B*51:35	714	722	IPTNFTISV	0.9219
B07	HLA-B*51:36	714	722	IPTNFTISV	0.8244
B07	HLA-B*51:36	1052	1061	FPQSAPHGVV	0.5349

B07	HLA-B*51:38	712	720	IAIPTNFTI	0.8936
B07	HLA-B*51:38	714	722	IPTNFTISV	0.9219
B07	HLA-B*53:01	24	32	LPPAYTNSF	0.7097
B07	HLA-B*53:01	55	64	FLPFFSNVTW	0.7688
B07	HLA-B*53:01	56	64	LPFFSNVTW	0.9556
B07	HLA-B*53:01	56	65	LPFFSNVTWF	0.502
B07	HLA-B*53:01	83	92	VLPFNDGVYF	0.7116
B07	HLA-B*53:01	84	92	LPFNDGVYF	0.967
B07	HLA-B*53:01	229	238	LPIGINITRF	0.8328
B07	HLA-B*53:01	250	258	TPGDSSSGW	0.8069
B07	HLA-B*53:01	320	329	VQPTESIVRF	0.5847
B07	HLA-B*53:01	321	329	QPTESIVRF	0.9157
B07	HLA-B*53:01	625	633	HADQLTPTW	0.9493
B07	HLA-B*53:01	714	722	IPTNFTISV	0.5509
B07	HLA-B*53:01	878	886	LAGTITSGW	0.6015
B07	HLA-B*53:01	896	904	IPFAMQMAY	0.8495
B07	HLA-B*53:01	898	906	FAMQMAYRF	0.7049
B07	HLA-B*53:01	1052	1060	FPQSAPHGV	0.6048
B07	HLA-B*53:01	1052	1062	FPQSAPHGVVF	0.6896
B07	HLA-B*53:02	24	32	LPPAYTNSF	0.6754
B07	HLA-B*53:02	53	64	DLFLPFFSNVTW	0.5156
B07	HLA-B*53:02	55	64	FLPFFSNVTW	0.7377
B07	HLA-B*53:02	56	64	LPFFSNVTW	0.9419
B07	HLA-B*53:02	83	92	VLPFNDGVYF	0.5685
B07	HLA-B*53:02	84	92	LPFNDGVYF	0.927
B07	HLA-B*53:02	229	238	LPIGINITRF	0.7712
B07	HLA-B*53:02	321	329	QPTESIVRF	0.8542
B07	HLA-B*53:02	625	633	HADQLTPTW	0.8778
B07	HLA-B*53:02	714	722	IPTNFTISV	0.5046
B07	HLA-B*53:02	896	904	IPFAMQMAY	0.8286
B07	HLA-B*53:02	898	906	FAMQMAYRF	0.7523
B07	HLA-B*53:02	1052	1062	FPQSAPHGVVF	0.6331
B07	HLA-B*53:06	24	32	LPPAYTNSF	0.7435
B07	HLA-B*53:06	55	64	FLPFFSNVTW	0.611
B07	HLA-B*53:06	56	64	LPFFSNVTW	0.897
B07	HLA-B*53:06	84	92	LPFNDGVYF	0.9269
B07	HLA-B*53:06	229	238	LPIGINITRF	0.7787
B07	HLA-B*53:06	321	329	QPTESIVRF	0.8336
B07	HLA-B*53:06	490	497	FPLQSYGF	0.5333
B07	HLA-B*53:06	625	633	HADQLTPTW	0.7925
B07	HLA-B*53:06	896	904	IPFAMQMAY	0.8651
B07	HLA-B*53:06	898	906	FAMQMAYRF	0.7249
B07	HLA-B*53:06	1052	1062	FPQSAPHGVVF	0.6456
B07	HLA-B*53:06	1095	1103	FVSNGTHWF	0.5268
B07	HLA-B*53:08	24	32	LPPAYTNSF	0.7478
B07	HLA-B*53:08	55	64	FLPFFSNVTW	0.634
B07	HLA-B*53:08	56	64	LPFFSNVTW	0.9209
B07	HLA-B*53:08	83	92	VLPFNDGVYF	0.713
B07	HLA-B*53:08	84	92	LPFNDGVYF	0.9579
B07	HLA-B*53:08	229	238	LPIGINITRF	0.8211

B07	HLA-B*53:08	250	258	TPGDSSSGW	0.701
B07	HLA-B*53:08	321	329	QPTESIVRF	0.8829
B07	HLA-B*53:08	625	633	HADQLTPTW	0.8712
B07	HLA-B*53:08	687	695	VASQSIIAY	0.7141
B07	HLA-B*53:08	712	720	IAIPTNFTI	0.5344
B07	HLA-B*53:08	714	722	IPTNFTISV	0.5417
B07	HLA-B*53:08	878	886	LAGTITSGW	0.576
B07	HLA-B*53:08	896	904	IPFAMQMAY	0.8758
B07	HLA-B*53:08	898	906	FAMQMAYRF	0.6831
B07	HLA-B*53:08	1052	1062	FPQSAPHGVVF	0.6472
B07	HLA-B*53:08	1095	1103	FVSNGTHWF	0.6097
B07	HLA-B*53:10	24	32	LPPAYTNSF	0.7097
B07	HLA-B*53:10	55	64	FLPFFSNVTW	0.7688
B07	HLA-B*53:10	56	64	LPFFSNVTW	0.9556
B07	HLA-B*53:10	56	65	LPFFSNVTWF	0.502
B07	HLA-B*53:10	83	92	VLPFNDGVYF	0.7116
B07	HLA-B*53:10	84	92	LPFNDGVYF	0.967
B07	HLA-B*53:10	229	238	LPIGINITRF	0.8328
B07	HLA-B*53:10	250	258	TPGDSSSGW	0.8069
B07	HLA-B*53:10	320	329	VQPTESIVRF	0.5847
B07	HLA-B*53:10	321	329	QPTESIVRF	0.9157
B07	HLA-B*53:10	625	633	HADQLTPTW	0.9493
B07	HLA-B*53:10	714	722	IPTNFTISV	0.5509
B07	HLA-B*53:10	878	886	LAGTITSGW	0.6015
B07	HLA-B*53:10	896	904	IPFAMQMAY	0.8495
B07	HLA-B*53:10	898	906	FAMQMAYRF	0.7049
B07	HLA-B*53:10	1052	1060	FPQSAPHGV	0.6048
B07	HLA-B*53:10	1052	1062	FPQSAPHGVVF	0.6896
B07	HLA-B*54:01	38	46	YPDKVFRSS	0.5631
B07	HLA-B*54:01	38	47	YPDKVFRSSV	0.551
B07	HLA-B*54:01	84	93	LPFNDGVYFA	0.8147
B07	HLA-B*54:01	336	344	CPFGEVFNA	0.675
B07	HLA-B*54:01	506	514	QPYRVVVLS	0.517
B07	HLA-B*54:01	664	672	IPIGAGICA	0.7761
B07	HLA-B*54:01	714	722	IPTNFTISV	0.8556
B07	HLA-B*54:01	1052	1060	FPQSAPHGV	0.7788
B07	HLA-B*54:01	1052	1061	FPQSAPHGVV	0.5637
B07	HLA-B*54:01	1089	1096	FPREGVFV	0.581
B07	HLA-B*54:01	1089	1097	FPREGVFVS	0.826
B07	HLA-B*54:03	38	46	YPDKVFRSS	0.5065
B07	HLA-B*54:03	84	93	LPFNDGVYFA	0.7103
B07	HLA-B*54:03	336	344	CPFGEVFNA	0.5864
B07	HLA-B*54:03	664	672	IPIGAGICA	0.6982
B07	HLA-B*54:03	714	722	IPTNFTISV	0.759
B07	HLA-B*54:03	1052	1060	FPQSAPHGV	0.7099
B07	HLA-B*54:03	1089	1097	FPREGVFVS	0.7861
B07	HLA-B*54:04	38	47	YPDKVFRSSV	0.6067
B07	HLA-B*54:04	84	92	LPFNDGVYF	0.6325
B07	HLA-B*54:04	84	93	LPFNDGVYFA	0.7208
B07	HLA-B*54:04	336	344	CPFGEVFNA	0.5184

B07	HLA-B*54:04	664	672	IPIGAGICA	0.7162
B07	HLA-B*54:04	714	722	IPTNFTISV	0.8759
B07	HLA-B*54:04	1052	1060	FPQSAPHGV	0.8116
B07	HLA-B*54:04	1052	1061	FPQSAPHGVV	0.6221
B07	HLA-B*54:04	1089	1096	FPREGVFV	0.626
B07	HLA-B*54:04	1089	1097	FPREGVFVS	0.7032
B07	HLA-B*54:06	24	32	LPPAYTNSF	0.5438
B07	HLA-B*54:06	84	92	LPFNDGVYF	0.7179
B07	HLA-B*54:06	271	279	QPRTFLLKY	0.5428
B07	HLA-B*54:06	687	695	VASQSIIAY	0.5887
B07	HLA-B*54:06	896	904	IPFAMQMAY	0.8139
B07	HLA-B*54:07	38	46	YPDKVFRSS	0.5631
B07	HLA-B*54:07	38	47	YPDKVFRSSV	0.551
B07	HLA-B*54:07	84	93	LPFNDGVYFA	0.8147
B07	HLA-B*54:07	336	344	CPFGEVFNA	0.675
B07	HLA-B*54:07	506	514	QPYRVVVLS	0.517
B07	HLA-B*54:07	664	672	IPIGAGICA	0.7761
B07	HLA-B*54:07	714	722	IPTNFTISV	0.8556
B07	HLA-B*54:07	1052	1060	FPQSAPHGV	0.7788
B07	HLA-B*54:07	1052	1061	FPQSAPHGVV	0.5637
B07	HLA-B*54:07	1089	1096	FPREGVFV	0.581
B07	HLA-B*54:07	1089	1097	FPREGVFVS	0.826
B07	HLA-B*55:01	664	672	IPIGAGICA	0.566
B07	HLA-B*55:01	680	688	SPRRARSVA	0.6445
B07	HLA-B*55:01	714	722	IPTNFTISV	0.699
B07	HLA-B*55:01	1052	1060	FPQSAPHGV	0.551
B07	HLA-B*55:01	1089	1097	FPREGVFVS	0.6525
B07	HLA-B*55:02	38	46	YPDKVFRSS	0.6589
B07	HLA-B*55:02	38	47	YPDKVFRSSV	0.5249
B07	HLA-B*55:02	84	92	LPFNDGVYF	0.6389
B07	HLA-B*55:02	84	93	LPFNDGVYFA	0.6565
B07	HLA-B*55:02	336	344	CPFGEVFNA	0.5229
B07	HLA-B*55:02	462	470	KPFERDIST	0.6378
B07	HLA-B*55:02	506	514	QPYRVVVLS	0.527
B07	HLA-B*55:02	664	672	IPIGAGICA	0.7531
B07	HLA-B*55:02	714	722	IPTNFTISV	0.8516
B07	HLA-B*55:02	896	904	IPFAMQMAY	0.5572
B07	HLA-B*55:02	1052	1060	FPQSAPHGV	0.758
B07	HLA-B*55:02	1052	1061	FPQSAPHGVV	0.5174
B07	HLA-B*55:02	1089	1096	FPREGVFV	0.5413
B07	HLA-B*55:02	1089	1097	FPREGVFVS	0.8144
B07	HLA-B*55:03	714	722	IPTNFTISV	0.6188
B07	HLA-B*55:03	1052	1060	FPQSAPHGV	0.5151
B07	HLA-B*55:03	1089	1097	FPREGVFVS	0.531
B07	HLA-B*55:04	24	32	LPPAYTNSF	0.7178
B07	HLA-B*55:04	38	47	YPDKVFRSSV	0.6017
B07	HLA-B*55:04	56	64	LPFFSNVTW	0.5466
B07	HLA-B*55:04	83	92	VLPFNDGVYF	0.5131
B07	HLA-B*55:04	84	92	LPFNDGVYF	0.8753
B07	HLA-B*55:04	208	216	TPINLVRDL	0.8684

B07	HLA-B*55:04	216	223	LPQGFSAL	0.6991
B07	HLA-B*55:04	229	238	LPIGINITRF	0.7322
B07	HLA-B*55:04	321	329	QPTESIVRF	0.7159
B07	HLA-B*55:04	462	470	KPFERDIST	0.5424
B07	HLA-B*55:04	506	513	QPYRVVVL	0.7157
B07	HLA-B*55:04	620	629	VPVAIHADQL	0.6147
B07	HLA-B*55:04	714	722	IPTNFTISV	0.896
B07	HLA-B*55:04	869	877	MIAQYTSAL	0.5791
B07	HLA-B*55:04	892	900	AALQIPFAM	0.5124
B07	HLA-B*55:04	896	904	IPFAMQMAY	0.6175
B07	HLA-B*55:04	1052	1060	FPQSAPHGV	0.8055
B07	HLA-B*55:04	1052	1062	FPQSAPHGVVF	0.6991
B07	HLA-B*55:04	1056	1063	APHGVVFL	0.6967
B07	HLA-B*55:04	1089	1097	FPREGVFVS	0.5475
B07	HLA-B*55:04	1261	1270	SEPVLKGVKL	0.5737
B07	HLA-B*55:04	1262	1270	EPVLKGVKL	0.7445
B07	HLA-B*55:05	664	672	IPIGAGICA	0.566
B07	HLA-B*55:05	680	688	SPRRARSVA	0.6445
B07	HLA-B*55:05	714	722	IPTNFTISV	0.699
B07	HLA-B*55:05	1052	1060	FPQSAPHGV	0.551
B07	HLA-B*55:05	1089	1097	FPREGVFVS	0.6525
B07	HLA-B*55:07	38	46	YPDKVFRSS	0.5631
B07	HLA-B*55:07	38	47	YPDKVFRSSV	0.551
B07	HLA-B*55:07	84	93	LPFNDGVYFA	0.8147
B07	HLA-B*55:07	336	344	CPFGEVFNA	0.675
B07	HLA-B*55:07	506	514	QPYRVVVLS	0.517
B07	HLA-B*55:07	664	672	IPIGAGICA	0.7761
B07	HLA-B*55:07	714	722	IPTNFTISV	0.8556
B07	HLA-B*55:07	1052	1060	FPQSAPHGV	0.7788
B07	HLA-B*55:07	1052	1061	FPQSAPHGVV	0.5637
B07	HLA-B*55:07	1089	1096	FPREGVFV	0.581
B07	HLA-B*55:07	1089	1097	FPREGVFVS	0.826
B07	HLA-B*55:09	38	47	YPDKVFRSSV	0.6045
B07	HLA-B*55:09	664	672	IPIGAGICA	0.5924
B07	HLA-B*55:09	680	688	SPRRARSVA	0.7242
B07	HLA-B*55:09	714	722	IPTNFTISV	0.7624
B07	HLA-B*55:09	1052	1060	FPQSAPHGV	0.5713
B07	HLA-B*55:09	1089	1097	FPREGVFVS	0.6196
B07	HLA-B*55:10	38	46	YPDKVFRSS	0.6429
B07	HLA-B*55:10	84	92	LPFNDGVYF	0.6586
B07	HLA-B*55:10	84	93	LPFNDGVYFA	0.567
B07	HLA-B*55:10	462	470	KPFERDIST	0.64
B07	HLA-B*55:10	664	672	IPIGAGICA	0.7168
B07	HLA-B*55:10	714	722	IPTNFTISV	0.8293
B07	HLA-B*55:10	896	904	IPFAMQMAY	0.5528
B07	HLA-B*55:10	1052	1060	FPQSAPHGV	0.723
B07	HLA-B*55:10	1089	1096	FPREGVFV	0.5148
B07	HLA-B*55:10	1089	1097	FPREGVFVS	0.776
B07	HLA-B*55:15	664	672	IPIGAGICA	0.566
B07	HLA-B*55:15	680	688	SPRRARSVA	0.6445

B07	HLA-B*55:15	714	722	IPTNFTISV	0.699
B07	HLA-B*55:15	1052	1060	FPQSAPHGV	0.551
B07	HLA-B*55:15	1089	1097	FPREGVFVS	0.6525
B07	HLA-B*55:17	462	470	KPFERDIST	0.5535
B07	HLA-B*55:17	664	672	IPIGAGICA	0.5355
B07	HLA-B*55:17	680	688	SPRRARSVA	0.6844
B07	HLA-B*55:17	714	722	IPTNFTISV	0.7936
B07	HLA-B*55:17	1052	1060	FPQSAPHGV	0.6104
B07	HLA-B*55:17	1089	1097	FPREGVFVS	0.5754
B07	HLA-B*55:19	38	46	YPDKVFRSS	0.6589
B07	HLA-B*55:19	38	47	YPDKVFRSSV	0.5249
B07	HLA-B*55:19	84	92	LPFNDGVYF	0.6389
B07	HLA-B*55:19	84	93	LPFNDGVYFA	0.6565
B07	HLA-B*55:19	336	344	CPFGEVFNA	0.5229
B07	HLA-B*55:19	462	470	KPFERDIST	0.6378
B07	HLA-B*55:19	506	514	QPYRVVVLS	0.527
B07	HLA-B*55:19	664	672	IPIGAGICA	0.7531
B07	HLA-B*55:19	714	722	IPTNFTISV	0.8516
B07	HLA-B*55:19	896	904	IPFAMQMAY	0.5572
B07	HLA-B*55:19	1052	1060	FPQSAPHGV	0.758
B07	HLA-B*55:19	1052	1061	FPQSAPHGVV	0.5174
B07	HLA-B*55:19	1089	1096	FPREGVFV	0.5413
B07	HLA-B*55:19	1089	1097	FPREGVFVS	0.8144
B07	HLA-B*56:01	38	46	YPDKVFRSS	0.5594
B07	HLA-B*56:01	84	92	LPFNDGVYF	0.6481
B07	HLA-B*56:01	84	93	LPFNDGVYFA	0.5533
B07	HLA-B*56:01	664	672	IPIGAGICA	0.6738
B07	HLA-B*56:01	714	722	IPTNFTISV	0.7754
B07	HLA-B*56:01	896	904	IPFAMQMAY	0.5446
B07	HLA-B*56:01	1052	1060	FPQSAPHGV	0.6791
B07	HLA-B*56:01	1089	1097	FPREGVFVS	0.7781
B07	HLA-B*56:03	84	92	LPFNDGVYF	0.65
B07	HLA-B*56:03	271	279	QPRTFLLKY	0.5652
B07	HLA-B*56:03	687	695	VASQSIIAY	0.5692
B07	HLA-B*56:03	896	904	IPFAMQMAY	0.7714
B07	HLA-B*56:05	664	672	IPIGAGICA	0.503
B07	HLA-B*56:05	680	688	SPRRARSVA	0.5529
B07	HLA-B*56:05	714	722	IPTNFTISV	0.7632
B07	HLA-B*56:05	896	904	IPFAMQMAY	0.5665
B07	HLA-B*56:05	1052	1060	FPQSAPHGV	0.6122
B07	HLA-B*56:05	1089	1096	FPREGVFV	0.5865
B07	HLA-B*56:05	1089	1097	FPREGVFVS	0.6335
B07	HLA-B*56:13	38	46	YPDKVFRSS	0.5603
B07	HLA-B*56:13	38	47	YPDKVFRSSV	0.5158
B07	HLA-B*56:13	664	672	IPIGAGICA	0.6238
B07	HLA-B*56:13	680	688	SPRRARSVA	0.513
B07	HLA-B*56:13	714	722	IPTNFTISV	0.769
B07	HLA-B*56:13	1052	1060	FPQSAPHGV	0.731
B07	HLA-B*56:13	1052	1061	FPQSAPHGVV	0.5165
B07	HLA-B*56:13	1089	1096	FPREGVFV	0.5099

B07	HLA-B*56:13	1089	1097	FPREGVFVS	0.715
B07	HLA-B*56:15	84	92	LPFNDGVYF	0.7262
B07	HLA-B*56:15	664	672	IPIGAGICA	0.6282
B07	HLA-B*56:15	714	722	IPTNFTISV	0.8617
B07	HLA-B*56:15	1052	1060	FPQSAPHGV	0.7753
B07	HLA-B*56:15	1052	1061	FPQSAPHGVV	0.5072
B07	HLA-B*56:15	1089	1097	FPREGVFVS	0.685
B07	HLA-B*56:16	38	46	YPDKVFRSS	0.5423
B07	HLA-B*56:16	84	92	LPFNDGVYF	0.6574
B07	HLA-B*56:16	664	672	IPIGAGICA	0.6319
B07	HLA-B*56:16	714	722	IPTNFTISV	0.7528
B07	HLA-B*56:16	896	904	IPFAMQMAY	0.5261
B07	HLA-B*56:16	1052	1060	FPQSAPHGV	0.6463
B07	HLA-B*56:16	1089	1097	FPREGVFVS	0.7472
B07	HLA-B*67:01	24	32	LPPAYTNSF	0.6526
B07	HLA-B*67:01	38	47	YPDKVFRSSV	0.5867
B07	HLA-B*67:01	38	48	YPDKVFRSSVL	0.5124
B07	HLA-B*67:01	84	92	LPFNDGVYF	0.8292
B07	HLA-B*67:01	208	216	TPINLVRDL	0.7949
B07	HLA-B*67:01	216	223	LPQGFSAL	0.5784
B07	HLA-B*67:01	229	238	LPIGINITRF	0.5824
B07	HLA-B*67:01	321	329	QPTESIVRF	0.6782
B07	HLA-B*67:01	506	513	QPYRVVVL	0.5066
B07	HLA-B*67:01	714	722	IPTNFTISV	0.8295
B07	HLA-B*67:01	861	869	LPPLLTDEM	0.5088
B07	HLA-B*67:01	896	904	IPFAMQMAY	0.5126
B07	HLA-B*67:01	1052	1060	FPQSAPHGV	0.7937
B07	HLA-B*67:01	1052	1062	FPQSAPHGVVF	0.6798
B07	HLA-B*67:01	1262	1270	EPVLKGVKL	0.7213
B07	HLA-B*78:01	714	722	IPTNFTISV	0.7595
B07	HLA-B*78:01	1052	1060	FPQSAPHGV	0.6988
B07	HLA-B*78:01	1089	1096	FPREGVFV	0.6628
B07	HLA-B*78:01	1089	1097	FPREGVFVS	0.5573
B07	HLA-B*78:02	714	722	IPTNFTISV	0.7684
B07	HLA-B*78:04	714	722	IPTNFTISV	0.7551
B07	HLA-B*78:04	1089	1097	FPREGVFVS	0.5305
B08	HLA-B*08:01	17	24	NLTTRTQL	0.5899
B08	HLA-B*08:01	233	241	INITRFQTL	0.7017
B08	HLA-B*08:01	234	241	NITRFQTL	0.7511
B08	HLA-B*08:01	241	249	LLALHRSYL	0.5553
B08	HLA-B*08:01	269	276	YLQPRTFL	0.7406
B08	HLA-B*08:01	269	277	YLQPRTFLL	0.7867
B08	HLA-B*08:01	506	513	QPYRVVVL	0.766
B08	HLA-B*08:01	821	828	LLFNKVTL	0.6195
B08	HLA-B*08:01	996	1004	LITGRLQSL	0.5644
B08	HLA-B*08:07	233	241	INITRFQTL	0.6349
B08	HLA-B*08:07	234	241	NITRFQTL	0.6414
B08	HLA-B*08:07	269	276	YLQPRTFL	0.6444
B08	HLA-B*08:07	269	277	YLQPRTFLL	0.7258
B08	HLA-B*08:07	506	513	QPYRVVVL	0.7156

B08	HLA-B*08:07	821	828	LLFNKVTL	0.5358
B08	HLA-B*08:07	996	1004	LITGRLQSL	0.5316
B08	HLA-B*08:09	269	277	YLQPRTFLL	0.5745
B08	HLA-B*08:11	17	24	NLTTRTQL	0.5719
B08	HLA-B*08:11	233	241	INITRFQTL	0.6926
B08	HLA-B*08:11	234	241	NITRFQTL	0.7381
B08	HLA-B*08:11	241	249	LLALHRSYL	0.5524
B08	HLA-B*08:11	269	276	YLQPRTFL	0.7196
B08	HLA-B*08:11	269	277	YLQPRTFLL	0.7806
B08	HLA-B*08:11	506	513	QPYRVVVL	0.7665
B08	HLA-B*08:11	821	828	LLFNKVTL	0.5958
B08	HLA-B*08:11	996	1004	LITGRLQSL	0.5708
B08	HLA-B*08:13	233	241	INITRFQTL	0.5821
B08	HLA-B*08:13	269	276	YLQPRTFL	0.5148
B08	HLA-B*08:13	269	277	YLQPRTFLL	0.6693
B08	HLA-B*08:13	506	513	QPYRVVVL	0.6559
B08	HLA-B*08:15	233	241	INITRFQTL	0.6284
B08	HLA-B*08:15	234	241	NITRFQTL	0.5714
B08	HLA-B*08:15	269	276	YLQPRTFL	0.6627
B08	HLA-B*08:15	269	277	YLQPRTFLL	0.7736
B08	HLA-B*08:15	821	828	LLFNKVTL	0.507
B08	HLA-B*08:18	17	24	NLTTRTQL	0.5899
B08	HLA-B*08:18	233	241	INITRFQTL	0.7017
B08	HLA-B*08:18	234	241	NITRFQTL	0.7511
B08	HLA-B*08:18	241	249	LLALHRSYL	0.5553
B08	HLA-B*08:18	269	276	YLQPRTFL	0.7406
B08	HLA-B*08:18	269	277	YLQPRTFLL	0.7867
B08	HLA-B*08:18	506	513	QPYRVVVL	0.766
B08	HLA-B*08:18	821	828	LLFNKVTL	0.6195
B08	HLA-B*08:18	996	1004	LITGRLQSL	0.5644
B08	HLA-B*08:20	241	249	LLALHRSYL	0.5123
B08	HLA-B*08:20	269	276	YLQPRTFL	0.5485
B08	HLA-B*08:20	269	277	YLQPRTFLL	0.6503
B08	HLA-B*08:20	869	877	MIAQYTSAL	0.5115
B08	HLA-B*08:21	233	241	INITRFQTL	0.5468
B08	HLA-B*08:21	234	241	NITRFQTL	0.598
B08	HLA-B*08:21	269	276	YLQPRTFL	0.5663
B08	HLA-B*08:21	269	277	YLQPRTFLL	0.6617
B08	HLA-B*08:21	506	513	QPYRVVVL	0.6419
B08	HLA-B*08:22	17	24	NLTTRTQL	0.5899
B08	HLA-B*08:22	233	241	INITRFQTL	0.7017
B08	HLA-B*08:22	234	241	NITRFQTL	0.7511
B08	HLA-B*08:22	241	249	LLALHRSYL	0.5553
B08	HLA-B*08:22	269	276	YLQPRTFL	0.7406
B08	HLA-B*08:22	269	277	YLQPRTFLL	0.7867
B08	HLA-B*08:22	506	513	QPYRVVVL	0.766
B08	HLA-B*08:22	821	828	LLFNKVTL	0.6195
B08	HLA-B*08:22	996	1004	LITGRLQSL	0.5644
B08	HLA-B*08:23	269	276	YLQPRTFL	0.5575
B08	HLA-B*08:23	269	277	YLQPRTFLL	0.6446

B08	HLA-B*08:23	821	828	LLFNKVTL	0.5015
B08	HLA-B*08:24	17	24	NLTTRTQL	0.5899
B08	HLA-B*08:24	233	241	INITRFQTL	0.7017
B08	HLA-B*08:24	234	241	NITRFQTL	0.7511
B08	HLA-B*08:24	241	249	LLALHRSYL	0.5553
B08	HLA-B*08:24	269	276	YLQPRTFL	0.7406
B08	HLA-B*08:24	269	277	YLQPRTFLL	0.7867
B08	HLA-B*08:24	506	513	QPYRVVVL	0.766
B08	HLA-B*08:24	821	828	LLFNKVTL	0.6195
B08	HLA-B*08:24	996	1004	LITGRLQSL	0.5644
B08	HLA-B*08:25	269	277	YLQPRTFLL	0.5151
B27	HLA-B*14:01	236	244	TRFQTLLAL	0.8018
B27	HLA-B*14:01	453	461	YRLFRKSNL	0.6967
B27	HLA-B*14:02	236	244	TRFQTLLAL	0.8018
B27	HLA-B*14:02	453	461	YRLFRKSNL	0.6967
B27	HLA-B*14:03	236	244	TRFQTLLAL	0.6892
B27	HLA-B*14:03	453	461	YRLFRKSNL	0.576
B27	HLA-B*14:06	236	244	TRFQTLLAL	0.8675
B27	HLA-B*14:06	764	772	NRALTGIAV	0.7167
B27	HLA-B*15:03	20	28	TRTQLPPAY	0.7211
B27	HLA-B*15:03	22	32	TQLPPAYTNSF	0.6455
B27	HLA-B*15:03	35	43	GVYYPDKVF	0.6663
B27	HLA-B*15:03	47	55	VLHSTQDLF	0.5243
B27	HLA-B*15:03	77	86	KRFDNPVLPF	0.8296
B27	HLA-B*15:03	184	192	GNFKNLREF	0.6499
B27	HLA-B*15:03	186	194	FKNLREFVF	0.5195
B27	HLA-B*15:03	192	200	FVFKNIDGY	0.5282
B27	HLA-B*15:03	212	220	LVRDLPQGF	0.576
B27	HLA-B*15:03	236	244	TRFQTLLAL	0.608
B27	HLA-B*15:03	240	248	TLLALHRSY	0.6413
B27	HLA-B*15:03	261	269	GAAAYYVGY	0.5148
B27	HLA-B*15:03	267	275	VGYLQPRTF	0.643
B27	HLA-B*15:03	320	329	VQPTESIVRF	0.5258
B27	HLA-B*15:03	327	335	VRFPNITNL	0.8039
B27	HLA-B*15:03	339	347	GEVFNATRF	0.7209
B27	HLA-B*15:03	345	353	TRFASVYAW	0.6043
B27	HLA-B*15:03	366	374	SVLYNSASF	0.5032
B27	HLA-B*15:03	369	377	YNSASFSTF	0.7633
B27	HLA-B*15:03	408	416	RQIAPGQTG	0.5804
B27	HLA-B*15:03	413	421	GQTGKIADY	0.8037
B27	HLA-B*15:03	443	451	SKVGGNYNY	0.9112
B27	HLA-B*15:03	456	464	FRKSNLKPF	0.5235
B27	HLA-B*15:03	464	473	FERDISTEIY	0.635
B27	HLA-B*15:03	497	505	FQPTNGVGY	0.8242
B27	HLA-B*15:03	557	565	KKFLPFQQF	0.9568
B27	HLA-B*15:03	628	636	QLTPTWRVY	0.5065
B27	HLA-B*15:03	634	643	RVYSTGSNVF	0.7212
B27	HLA-B*15:03	687	695	VASQSIIAY	0.7907
B27	HLA-B*15:03	689	697	SQSIIAYTM	0.7655
B27	HLA-B*15:03	699	707	LGAENSVAY	0.5647

B27	HLA-B*15:03	710	718	NSIAIPTNF	0.5451
B27	HLA-B*15:03	789	797	YKTPPIKDF	0.8036
B27	HLA-B*15:03	794	802	IKDFGGFNF	0.7241
B27	HLA-B*15:03	815	823	RSFIEDLLF	0.624
B27	HLA-B*15:03	852	860	AQKFNGLTV	0.6121
B27	HLA-B*15:03	852	861	AQKFNGLTVL	0.5743
B27	HLA-B*15:03	853	861	QKFNGLTVL	0.7866
B27	HLA-B*15:03	880	888	GTITSGWTF	0.5063
B27	HLA-B*15:03	893	902	ALQIPFAMQM	0.5298
B27	HLA-B*15:03	894	902	LQIPFAMQM	0.9192
B27	HLA-B*15:03	919	927	NQKLIANQF	0.8596
B27	HLA-B*15:03	999	1007	GRLQSLQTY	0.8486
B27	HLA-B*15:03	1044	1052	GKGYHLMSF	0.7673
B27	HLA-B*15:03	1054	1062	QSAPHGVVF	0.8355
B27	HLA-B*15:03	1087	1095	AHFPREGVF	0.7921
B27	HLA-B*15:03	1113	1121	QIITTDNTF	0.5046
B27	HLA-B*15:03	1130	1138	IGIVNNTVY	0.5224
B27	HLA-B*15:03	1264	1272	VLKGVKLHY	0.6224
B27	HLA-B*15:18	20	28	TRTQLPPAY	0.7269
B27	HLA-B*15:18	236	244	TRFQTLLAL	0.6174
B27	HLA-B*15:18	1087	1095	AHFPREGVF	0.6545
B27	HLA-B*15:47	77	86	KRFDNPVLPF	0.5237
B27	HLA-B*15:47	339	347	GEVFNATRF	0.6636
B27	HLA-B*15:47	369	377	YNSASFSTF	0.5152
B27	HLA-B*15:47	413	421	GQTGKIADY	0.5603
B27	HLA-B*15:47	464	473	FERDISTEIY	0.5227
B27	HLA-B*15:47	557	565	KKFLPFQQF	0.7088
B27	HLA-B*15:47	687	695	VASQSIIAY	0.5883
B27	HLA-B*15:47	689	697	SQSIIAYTM	0.6903
B27	HLA-B*15:47	852	861	AQKFNGLTVL	0.503
B27	HLA-B*15:47	894	902	LQIPFAMQM	0.6781
B27	HLA-B*15:47	919	927	NQKLIANQF	0.6441
B27	HLA-B*15:47	1054	1062	QSAPHGVVF	0.5973
B27	HLA-B*15:47	1087	1095	AHFPREGVF	0.5178
B27	HLA-B*15:49	77	86	KRFDNPVLPF	0.5237
B27	HLA-B*15:49	339	347	GEVFNATRF	0.6636
B27	HLA-B*15:49	369	377	YNSASFSTF	0.5152
B27	HLA-B*15:49	413	421	GQTGKIADY	0.5603
B27	HLA-B*15:49	464	473	FERDISTEIY	0.5227
B27	HLA-B*15:49	557	565	KKFLPFQQF	0.7088
B27	HLA-B*15:49	687	695	VASQSIIAY	0.5883
B27	HLA-B*15:49	689	697	SQSIIAYTM	0.6903
B27	HLA-B*15:49	852	861	AQKFNGLTVL	0.503
B27	HLA-B*15:49	894	902	LQIPFAMQM	0.6781
B27	HLA-B*15:49	919	927	NQKLIANQF	0.6441
B27	HLA-B*15:49	1054	1062	QSAPHGVVF	0.5973
B27	HLA-B*15:49	1087	1095	AHFPREGVF	0.5178
B27	HLA-B*15:52	236	244	TRFQTLLAL	0.6881
B27	HLA-B*15:54	20	28	TRTQLPPAY	0.6536
B27	HLA-B*15:54	22	32	TQLPPAYTNSF	0.5407

B27	HLA-B*15:54	35	43	GVYYPDKVF	0.5415
B27	HLA-B*15:54	77	86	KRFDNPVLPF	0.707
B27	HLA-B*15:54	212	220	LVRDLPQGF	0.5164
B27	HLA-B*15:54	240	248	TLLALHRSY	0.5397
B27	HLA-B*15:54	320	329	VQPTESIVRF	0.5256
B27	HLA-B*15:54	339	347	GEVFNATRF	0.706
B27	HLA-B*15:54	369	377	YNSASFSTF	0.659
B27	HLA-B*15:54	408	416	RQIAPGQTG	0.5392
B27	HLA-B*15:54	413	421	GQTGKIADY	0.8083
B27	HLA-B*15:54	443	451	SKVGGNYNY	0.849
B27	HLA-B*15:54	456	464	FRKSNLKPF	0.5182
B27	HLA-B*15:54	464	473	FERDISTEIY	0.7035
B27	HLA-B*15:54	497	505	FQPTNGVGY	0.7908
B27	HLA-B*15:54	557	565	KKFLPFQQF	0.8533
B27	HLA-B*15:54	634	643	RVYSTGSNVF	0.6621
B27	HLA-B*15:54	687	695	VASQSIIAY	0.6625
B27	HLA-B*15:54	689	697	SQSIIAYTM	0.6728
B27	HLA-B*15:54	789	797	YKTPPIKDF	0.6416
B27	HLA-B*15:54	852	860	AQKFNGLTV	0.5786
B27	HLA-B*15:54	852	861	AQKFNGLTVL	0.5715
B27	HLA-B*15:54	853	861	QKFNGLTVL	0.5953
B27	HLA-B*15:54	894	902	LQIPFAMQM	0.7958
B27	HLA-B*15:54	919	927	NQKLIANQF	0.8585
B27	HLA-B*15:54	999	1007	GRLQSLQTY	0.757
B27	HLA-B*15:54	1044	1052	GKGYHLMSF	0.6503
B27	HLA-B*15:54	1054	1062	QSAPHGVVF	0.7441
B27	HLA-B*15:54	1087	1095	AHFPREGVF	0.6276
B27	HLA-B*15:54	1264	1272	VLKGVKLHY	0.6417
B27	HLA-B*15:61	20	28	TRTQLPPAY	0.771
B27	HLA-B*15:61	35	43	GVYYPDKVF	0.5503
B27	HLA-B*15:61	77	86	KRFDNPVLPF	0.8652
B27	HLA-B*15:61	236	244	TRFQTLLAL	0.7603
B27	HLA-B*15:61	318	326	FRVQPTESI	0.5563
B27	HLA-B*15:61	327	335	VRFPNITNL	0.9057
B27	HLA-B*15:61	345	353	TRFASVYAW	0.6322
B27	HLA-B*15:61	369	377	YNSASFSTF	0.5292
B27	HLA-B*15:61	413	421	GQTGKIADY	0.6318
B27	HLA-B*15:61	443	451	SKVGGNYNY	0.8055
B27	HLA-B*15:61	456	464	FRKSNLKPF	0.5874
B27	HLA-B*15:61	497	505	FQPTNGVGY	0.6258
B27	HLA-B*15:61	557	565	KKFLPFQQF	0.895
B27	HLA-B*15:61	634	643	RVYSTGSNVF	0.5973
B27	HLA-B*15:61	687	695	VASQSIIAY	0.5972
B27	HLA-B*15:61	689	697	SQSIIAYTM	0.6401
B27	HLA-B*15:61	789	797	YKTPPIKDF	0.7085
B27	HLA-B*15:61	794	802	IKDFGGFNF	0.5487
B27	HLA-B*15:61	852	860	AQKFNGLTV	0.6105
B27	HLA-B*15:61	852	861	AQKFNGLTVL	0.5775
B27	HLA-B*15:61	853	861	QKFNGLTVL	0.7774
B27	HLA-B*15:61	894	902	LQIPFAMQM	0.7963

B27	HLA-B*15:61	919	927	NQKLIANQF	0.6902
B27	HLA-B*15:61	999	1007	GRLQSLQTY	0.8572
B27	HLA-B*15:61	1038	1047	KRVDFCGKGY	0.5634
B27	HLA-B*15:61	1044	1052	GKGYHLMSF	0.636
B27	HLA-B*15:61	1054	1062	QSAPHGVVF	0.6853
B27	HLA-B*15:61	1087	1095	AHFPREGVF	0.6534
B27	HLA-B*15:61	1264	1272	VLKGVKLHY	0.5156
B27	HLA-B*15:62	20	28	TRTQLPPAY	0.7053
B27	HLA-B*15:62	22	32	TQLPPAYTNSF	0.6112
B27	HLA-B*15:62	30	38	NSFTRGVYY	0.5218
B27	HLA-B*15:62	35	43	GVYYPDKVF	0.6492
B27	HLA-B*15:62	47	55	VLHSTQDLF	0.5047
B27	HLA-B*15:62	77	86	KRFDNPVLPF	0.7733
B27	HLA-B*15:62	184	192	GNFKNLREF	0.6307
B27	HLA-B*15:62	192	200	FVFKNIDGY	0.5448
B27	HLA-B*15:62	212	220	LVRDLPQGF	0.5648
B27	HLA-B*15:62	236	244	TRFQTLLAL	0.573
B27	HLA-B*15:62	240	248	TLLALHRSY	0.6832
B27	HLA-B*15:62	261	269	GAAAYYVGY	0.5324
B27	HLA-B*15:62	267	275	VGYLQPRTF	0.6274
B27	HLA-B*15:62	327	335	VRFPNITNL	0.7421
B27	HLA-B*15:62	339	347	GEVFNATRF	0.7077
B27	HLA-B*15:62	345	353	TRFASVYAW	0.577
B27	HLA-B*15:62	366	374	SVLYNSASF	0.5129
B27	HLA-B*15:62	369	377	YNSASFSTF	0.746
B27	HLA-B*15:62	408	416	RQIAPGQTG	0.5385
B27	HLA-B*15:62	413	421	GQTGKIADY	0.813
B27	HLA-B*15:62	443	451	SKVGGNYNY	0.9183
B27	HLA-B*15:62	464	473	FERDISTEIY	0.6286
B27	HLA-B*15:62	497	505	FQPTNGVGY	0.8232
B27	HLA-B*15:62	557	565	KKFLPFQQF	0.9461
B27	HLA-B*15:62	628	636	QLTPTWRVY	0.5393
B27	HLA-B*15:62	634	643	RVYSTGSNVF	0.7129
B27	HLA-B*15:62	687	695	VASQSIIAY	0.7985
B27	HLA-B*15:62	689	697	SQSIIAYTM	0.7527
B27	HLA-B*15:62	699	707	LGAENSVAY	0.5751
B27	HLA-B*15:62	710	718	NSIAIPTNF	0.5361
B27	HLA-B*15:62	789	797	YKTPPIKDF	0.7655
B27	HLA-B*15:62	794	802	IKDFGGFNF	0.6781
B27	HLA-B*15:62	815	823	RSFIEDLLF	0.5936
B27	HLA-B*15:62	852	860	AQKFNGLTV	0.582
B27	HLA-B*15:62	852	861	AQKFNGLTVL	0.5232
B27	HLA-B*15:62	853	861	QKFNGLTVL	0.7613
B27	HLA-B*15:62	893	902	ALQIPFAMQM	0.5135
B27	HLA-B*15:62	894	902	LQIPFAMQM	0.911
B27	HLA-B*15:62	896	904	IPFAMQMAY	0.5327
B27	HLA-B*15:62	919	927	NQKLIANQF	0.8564
B27	HLA-B*15:62	999	1007	GRLQSLQTY	0.8305
B27	HLA-B*15:62	1044	1052	GKGYHLMSF	0.7316
B27	HLA-B*15:62	1054	1062	QSAPHGVVF	0.8256

B27	HLA-B*15:62	1087	1095	AHFPREGVF	0.7587
B27	HLA-B*15:62	1113	1121	QIITTDNTF	0.5006
B27	HLA-B*15:62	1130	1138	IGIVNNTVY	0.5213
B27	HLA-B*15:62	1264	1272	VLKGVKLHY	0.645
B27	HLA-B*15:69	20	28	TRTQLPPAY	0.7227
B27	HLA-B*15:69	22	32	TQLPPAYTNSF	0.5591
B27	HLA-B*15:69	35	43	GVYYPDKVF	0.5772
B27	HLA-B*15:69	77	86	KRFDNPVLPF	0.851
B27	HLA-B*15:69	184	192	GNFKNLREF	0.546
B27	HLA-B*15:69	236	244	TRFQTLLAL	0.6858
B27	HLA-B*15:69	240	248	TLLALHRSY	0.525
B27	HLA-B*15:69	267	275	VGYLQPRTF	0.5372
B27	HLA-B*15:69	327	335	VRFPNITNL	0.8345
B27	HLA-B*15:69	339	347	GEVFNATRF	0.7224
B27	HLA-B*15:69	345	353	TRFASVYAW	0.5818
B27	HLA-B*15:69	369	377	YNSASFSTF	0.6438
B27	HLA-B*15:69	408	416	RQIAPGQTG	0.5308
B27	HLA-B*15:69	413	421	GQTGKIADY	0.7354
B27	HLA-B*15:69	443	451	SKVGGNYNY	0.8229
B27	HLA-B*15:69	464	473	FERDISTEIY	0.6128
B27	HLA-B*15:69	497	505	FQPTNGVGY	0.674
B27	HLA-B*15:69	505	513	YQPYRVVVL	0.5216
B27	HLA-B*15:69	557	565	KKFLPFQQF	0.9341
B27	HLA-B*15:69	634	643	RVYSTGSNVF	0.6281
B27	HLA-B*15:69	687	695	VASQSIIAY	0.6873
B27	HLA-B*15:69	689	697	SQSIIAYTM	0.7524
B27	HLA-B*15:69	789	797	YKTPPIKDF	0.6877
B27	HLA-B*15:69	794	802	IKDFGGFNF	0.691
B27	HLA-B*15:69	815	823	RSFIEDLLF	0.5525
B27	HLA-B*15:69	852	860	AQKFNGLTV	0.584
B27	HLA-B*15:69	852	861	AQKFNGLTVL	0.6049
B27	HLA-B*15:69	853	861	QKFNGLTVL	0.7612
B27	HLA-B*15:69	893	902	ALQIPFAMQM	0.5311
B27	HLA-B*15:69	894	902	LQIPFAMQM	0.8912
B27	HLA-B*15:69	919	927	NQKLIANQF	0.8102
B27	HLA-B*15:69	999	1007	GRLQSLQTY	0.8376
B27	HLA-B*15:69	1016	1024	AEIRASANL	0.5219
B27	HLA-B*15:69	1038	1047	KRVDFCGKGY	0.5143
B27	HLA-B*15:69	1044	1052	GKGYHLMSF	0.7181
B27	HLA-B*15:69	1054	1062	QSAPHGVVF	0.7141
B27	HLA-B*15:69	1087	1095	AHFPREGVF	0.769
B27	HLA-B*15:69	1264	1272	VLKGVKLHY	0.5553
B27	HLA-B*15:72	20	28	TRTQLPPAY	0.7269
B27	HLA-B*15:72	236	244	TRFQTLLAL	0.6174
B27	HLA-B*15:72	1087	1095	AHFPREGVF	0.6545
B27	HLA-B*15:74	20	28	TRTQLPPAY	0.712
B27	HLA-B*15:74	22	32	TQLPPAYTNSF	0.6447
B27	HLA-B*15:74	30	38	NSFTRGVYY	0.5264
B27	HLA-B*15:74	35	43	GVYYPDKVF	0.6849
B27	HLA-B*15:74	47	55	VLHSTQDLF	0.519

B27	HLA-B*15:74	77	86	KRFDNPVLPF	0.8142
B27	HLA-B*15:74	84	92	LPFNDGVYF	0.5129
B27	HLA-B*15:74	184	192	GNFKNLREF	0.6596
B27	HLA-B*15:74	186	194	FKNLREFVF	0.5086
B27	HLA-B*15:74	192	200	FVFKNIDGY	0.5716
B27	HLA-B*15:74	212	220	LVRDLPQGF	0.5986
B27	HLA-B*15:74	236	244	TRFQTLLAL	0.6029
B27	HLA-B*15:74	240	248	TLLALHRSY	0.6632
B27	HLA-B*15:74	261	269	GAAAYYVGY	0.5496
B27	HLA-B*15:74	267	275	VGYLQPRTF	0.6398
B27	HLA-B*15:74	320	329	VQPTESIVRF	0.5264
B27	HLA-B*15:74	327	335	VRFPNITNL	0.7868
B27	HLA-B*15:74	339	347	GEVFNATRF	0.7102
B27	HLA-B*15:74	345	353	TRFASVYAW	0.583
B27	HLA-B*15:74	366	374	SVLYNSASF	0.5392
B27	HLA-B*15:74	369	377	YNSASFSTF	0.7528
B27	HLA-B*15:74	408	416	RQIAPGQTG	0.5683
B27	HLA-B*15:74	413	421	GQTGKIADY	0.7923
B27	HLA-B*15:74	443	451	SKVGGNYNY	0.9075
B27	HLA-B*15:74	456	464	FRKSNLKPF	0.5033
B27	HLA-B*15:74	464	473	FERDISTEIY	0.6276
B27	HLA-B*15:74	497	505	FQPTNGVGY	0.8159
B27	HLA-B*15:74	557	565	KKFLPFQQF	0.96
B27	HLA-B*15:74	628	636	QLTPTWRVY	0.5215
B27	HLA-B*15:74	634	643	RVYSTGSNVF	0.7445
B27	HLA-B*15:74	687	695	VASQSIIAY	0.7995
B27	HLA-B*15:74	689	697	SQSIIAYTM	0.7813
B27	HLA-B*15:74	699	707	LGAENSVAY	0.5653
B27	HLA-B*15:74	710	718	NSIAIPTNF	0.5635
B27	HLA-B*15:74	789	797	YKTPPIKDF	0.7759
B27	HLA-B*15:74	794	802	IKDFGGFNF	0.6901
B27	HLA-B*15:74	815	823	RSFIEDLLF	0.6421
B27	HLA-B*15:74	852	860	AQKFNGLTV	0.6162
B27	HLA-B*15:74	852	861	AQKFNGLTVL	0.5897
B27	HLA-B*15:74	853	861	QKFNGLTVL	0.772
B27	HLA-B*15:74	880	888	GTITSGWTF	0.5407
B27	HLA-B*15:74	893	902	ALQIPFAMQM	0.57
B27	HLA-B*15:74	894	902	LQIPFAMQM	0.9324
B27	HLA-B*15:74	896	904	IPFAMQMAY	0.5172
B27	HLA-B*15:74	919	927	NQKLIANQF	0.843
B27	HLA-B*15:74	999	1007	GRLQSLQTY	0.8373
B27	HLA-B*15:74	1044	1052	GKGYHLMSF	0.7534
B27	HLA-B*15:74	1054	1062	QSAPHGVVF	0.8478
B27	HLA-B*15:74	1087	1095	AHFPREGVF	0.7577
B27	HLA-B*15:74	1113	1121	QIITTDNTF	0.5173
B27	HLA-B*15:74	1130	1138	IGIVNNTVY	0.5267
B27	HLA-B*15:74	1264	1272	VLKGVKLHY	0.6447
B27	HLA-B*15:80	20	28	TRTQLPPAY	0.7191
B27	HLA-B*15:80	236	244	TRFQTLLAL	0.5772
B27	HLA-B*15:80	1087	1095	AHFPREGVF	0.5968

B27	HLA-B*15:91	153	160	MESEFRVY	0.6327
B27	HLA-B*15:91	297	306	SETKCTLKSF	0.5716
B27	HLA-B*15:91	339	347	GEVFNATRF	0.8899
B27	HLA-B*15:91	464	473	FERDISTEIY	0.7127
B27	HLA-B*15:91	653	660	AEHVNNSY	0.6902
B27	HLA-B*15:91	689	697	SQSIIAYTM	0.5235
B27	HLA-B*15:91	919	927	NQKLIANQF	0.6049
B27	HLA-B*15:91	1016	1024	AEIRASANL	0.7495
B27	HLA-B*15:91	1201	1209	QELGKYEQY	0.8926
B27	HLA-B*15:93	236	244	TRFQTLLAL	0.7254
B27	HLA-B*15:93	1087	1095	AHFPREGVF	0.7077
B27	HLA-B*15:98	20	28	TRTQLPPAY	0.7211
B27	HLA-B*15:98	22	32	TQLPPAYTNSF	0.6455
B27	HLA-B*15:98	35	43	GVYYPDKVF	0.6663
B27	HLA-B*15:98	47	55	VLHSTQDLF	0.5243
B27	HLA-B*15:98	77	86	KRFDNPVLPF	0.8296
B27	HLA-B*15:98	184	192	GNFKNLREF	0.6499
B27	HLA-B*15:98	186	194	FKNLREFVF	0.5195
B27	HLA-B*15:98	192	200	FVFKNIDGY	0.5282
B27	HLA-B*15:98	212	220	LVRDLPQGF	0.576
B27	HLA-B*15:98	236	244	TRFQTLLAL	0.608
B27	HLA-B*15:98	240	248	TLLALHRSY	0.6413
B27	HLA-B*15:98	261	269	GAAAYYVGY	0.5148
B27	HLA-B*15:98	267	275	VGYLQPRTF	0.643
B27	HLA-B*15:98	320	329	VQPTESIVRF	0.5258
B27	HLA-B*15:98	327	335	VRFPNITNL	0.8039
B27	HLA-B*15:98	339	347	GEVFNATRF	0.7209
B27	HLA-B*15:98	345	353	TRFASVYAW	0.6043
B27	HLA-B*15:98	366	374	SVLYNSASF	0.5032
B27	HLA-B*15:98	369	377	YNSASFSTF	0.7633
B27	HLA-B*15:98	408	416	RQIAPGQTG	0.5804
B27	HLA-B*15:98	413	421	GQTGKIADY	0.8037
B27	HLA-B*15:98	443	451	SKVGGNYNY	0.9112
B27	HLA-B*15:98	456	464	FRKSNLKPF	0.5235
B27	HLA-B*15:98	464	473	FERDISTEIY	0.635
B27	HLA-B*15:98	497	505	FQPTNGVGY	0.8242
B27	HLA-B*15:98	557	565	KKFLPFQQF	0.9568
B27	HLA-B*15:98	628	636	QLTPTWRVY	0.5065
B27	HLA-B*15:98	634	643	RVYSTGSNVF	0.7212
B27	HLA-B*15:98	687	695	VASQSIIAY	0.7907
B27	HLA-B*15:98	689	697	SQSIIAYTM	0.7655
B27	HLA-B*15:98	699	707	LGAENSVAY	0.5647
B27	HLA-B*15:98	710	718	NSIAIPTNF	0.5451
B27	HLA-B*15:98	789	797	YKTPPIKDF	0.8036
B27	HLA-B*15:98	794	802	IKDFGGFNF	0.7241
B27	HLA-B*15:98	815	823	RSFIEDLLF	0.624
B27	HLA-B*15:98	852	860	AQKFNGLTV	0.6121
B27	HLA-B*15:98	852	861	AQKFNGLTVL	0.5743
B27	HLA-B*15:98	853	861	QKFNGLTVL	0.7866
B27	HLA-B*15:98	880	888	GTITSGWTF	0.5063

B27	HLA-B*15:98	893	902	ALQIPFAMQM	0.5298
B27	HLA-B*15:98	894	902	LQIPFAMQM	0.9192
B27	HLA-B*15:98	919	927	NQKLIANQF	0.8596
B27	HLA-B*15:98	999	1007	GRLQSLQTY	0.8486
B27	HLA-B*15:98	1044	1052	GKGYHLMSF	0.7673
B27	HLA-B*15:98	1054	1062	QSAPHGVVF	0.8355
B27	HLA-B*15:98	1087	1095	AHFPREGVF	0.7921
B27	HLA-B*15:98	1113	1121	QIITTDNTF	0.5046
B27	HLA-B*15:98	1130	1138	IGIVNNTVY	0.5224
B27	HLA-B*15:98	1264	1272	VLKGVKLHY	0.6224
B27	HLA-B*27:02	77	86	KRFDNPVLPF	0.9936
B27	HLA-B*27:02	236	244	TRFQTLLAL	0.9672
B27	HLA-B*27:02	342	353	FNATRFASVYAW	0.5105
B27	HLA-B*27:02	343	353	NATRFASVYAW	0.7021
B27	HLA-B*27:02	344	353	ATRFASVYAW	0.9567
B27	HLA-B*27:02	345	353	TRFASVYAW	0.9908
B27	HLA-B*27:02	356	365	KRISNCVADY	0.7362
B27	HLA-B*27:02	814	823	KRSFIEDLLF	0.9772
B27	HLA-B*27:02	846	855	ARDLICAQKF	0.8438
B27	HLA-B*27:02	999	1007	GRLQSLQTY	0.9922
B27	HLA-B*27:03	77	86	KRFDNPVLPF	0.9834
B27	HLA-B*27:03	814	823	KRSFIEDLLF	0.8897
B27	HLA-B*27:04	77	86	KRFDNPVLPF	0.986
B27	HLA-B*27:04	235	244	ITRFQTLLAL	0.5856
B27	HLA-B*27:04	236	244	TRFQTLLAL	0.9822
B27	HLA-B*27:04	327	335	VRFPNITNL	0.9799
B27	HLA-B*27:04	453	461	YRLFRKSNL	0.6317
B27	HLA-B*27:04	814	823	KRSFIEDLLF	0.8383
B27	HLA-B*27:04	904	912	YRFNGIGVT	0.6001
B27	HLA-B*27:04	999	1007	GRLQSLQTY	0.967
B27	HLA-B*27:05	77	86	KRFDNPVLPF	0.9904
B27	HLA-B*27:05	101	110	IRGWIFGTTL	0.6849
B27	HLA-B*27:05	235	244	ITRFQTLLAL	0.5227
B27	HLA-B*27:05	236	244	TRFQTLLAL	0.9782
B27	HLA-B*27:05	318	326	FRVQPTESI	0.6722
B27	HLA-B*27:05	327	335	VRFPNITNL	0.9818
B27	HLA-B*27:05	344	353	ATRFASVYAW	0.7783
B27	HLA-B*27:05	345	353	TRFASVYAW	0.9414
B27	HLA-B*27:05	356	365	KRISNCVADY	0.6725
B27	HLA-B*27:05	453	461	YRLFRKSNL	0.6377
B27	HLA-B*27:05	814	822	KRSFIEDLL	0.8024
B27	HLA-B*27:05	814	823	KRSFIEDLLF	0.9257
B27	HLA-B*27:05	846	855	ARDLICAQKF	0.5398
B27	HLA-B*27:05	904	913	YRFNGIGVTQ	0.7685
B27	HLA-B*27:05	999	1007	GRLQSLQTY	0.9825
B27	HLA-B*27:05	1038	1047	KRVDFCGKGY	0.8924
B27	HLA-B*27:05	1038	1049	KRVDFCGKGYHL	0.663
B27	HLA-B*27:06	77	84	KRFDNPVL	0.7543
B27	HLA-B*27:06	77	86	KRFDNPVLPF	0.9867
B27	HLA-B*27:06	101	110	IRGWIFGTTL	0.7577

B27	HLA-B*27:06	235	244	ITRFQTLLAL	0.6798
B27	HLA-B*27:06	236	244	TRFQTLLAL	0.9905
B27	HLA-B*27:06	318	326	FRVQPTESI	0.887
B27	HLA-B*27:06	325	335	SIVRFPNITNL	0.8466
B27	HLA-B*27:06	326	335	IVRFPNITNL	0.8762
B27	HLA-B*27:06	327	335	VRFPNITNL	0.9922
B27	HLA-B*27:06	453	461	YRLFRKSNL	0.7499
B27	HLA-B*27:06	684	692	ARSVASQSI	0.6368
B27	HLA-B*27:06	764	772	NRALTGIAV	0.588
B27	HLA-B*27:06	814	822	KRSFIEDLL	0.749
B27	HLA-B*27:06	814	823	KRSFIEDLLF	0.6968
B27	HLA-B*27:06	853	861	QKFNGLTVL	0.5745
B27	HLA-B*27:06	904	911	YRFNGIGV	0.617
B27	HLA-B*27:06	904	915	YRFNGIGVTQNV	0.5027
B27	HLA-B*27:06	1038	1049	KRVDFCGKGYHL	0.6164
B27	HLA-B*27:06	1106	1114	QRNFYEPQI	0.601
B27	HLA-B*27:07	77	84	KRFDNPVL	0.7135
B27	HLA-B*27:07	77	86	KRFDNPVLPF	0.9902
B27	HLA-B*27:07	101	110	IRGWIFGTTL	0.8223
B27	HLA-B*27:07	235	244	ITRFQTLLAL	0.6211
B27	HLA-B*27:07	236	244	TRFQTLLAL	0.9877
B27	HLA-B*27:07	318	326	FRVQPTESI	0.8291
B27	HLA-B*27:07	325	335	SIVRFPNITNL	0.8921
B27	HLA-B*27:07	326	335	IVRFPNITNL	0.8934
B27	HLA-B*27:07	327	335	VRFPNITNL	0.9922
B27	HLA-B*27:07	327	338	VRFPNITNLCPF	0.7232
B27	HLA-B*27:07	344	353	ATRFASVYAW	0.5458
B27	HLA-B*27:07	345	353	TRFASVYAW	0.8735
B27	HLA-B*27:07	453	461	YRLFRKSNL	0.6719
B27	HLA-B*27:07	557	565	KKFLPFQQF	0.6105
B27	HLA-B*27:07	684	692	ARSVASQSI	0.577
B27	HLA-B*27:07	764	772	NRALTGIAV	0.5708
B27	HLA-B*27:07	814	822	KRSFIEDLL	0.9203
B27	HLA-B*27:07	814	823	KRSFIEDLLF	0.8818
B27	HLA-B*27:07	904	911	YRFNGIGV	0.614
B27	HLA-B*27:07	904	915	YRFNGIGVTQNV	0.5884
B27	HLA-B*27:07	999	1008	GRLQSLQTYV	0.526
B27	HLA-B*27:07	1038	1049	KRVDFCGKGYHL	0.8228
B27	HLA-B*27:07	1106	1114	QRNFYEPQI	0.7313
B27	HLA-B*27:09	77	84	KRFDNPVL	0.7057
B27	HLA-B*27:09	77	86	KRFDNPVLPF	0.9931
B27	HLA-B*27:09	101	110	IRGWIFGTTL	0.8159
B27	HLA-B*27:09	235	244	ITRFQTLLAL	0.6732
B27	HLA-B*27:09	236	244	TRFQTLLAL	0.9861
B27	HLA-B*27:09	318	326	FRVQPTESI	0.8298
B27	HLA-B*27:09	325	335	SIVRFPNITNL	0.8994
B27	HLA-B*27:09	326	335	IVRFPNITNL	0.9018
B27	HLA-B*27:09	327	335	VRFPNITNL	0.9903
B27	HLA-B*27:09	327	338	VRFPNITNLCPF	0.7706
B27	HLA-B*27:09	344	353	ATRFASVYAW	0.6784

B27	HLA-B*27:09	345	353	TRFASVYAW	0.904
B27	HLA-B*27:09	453	461	YRLFRKSNL	0.6207
B27	HLA-B*27:09	764	772	NRALTGIAV	0.51
B27	HLA-B*27:09	814	822	KRSFIEDLL	0.9093
B27	HLA-B*27:09	814	823	KRSFIEDLLF	0.925
B27	HLA-B*27:09	846	855	ARDLICAQKF	0.5004
B27	HLA-B*27:09	904	911	YRFNGIGV	0.6307
B27	HLA-B*27:09	904	912	YRFNGIGVT	0.5198
B27	HLA-B*27:09	904	915	YRFNGIGVTQNV	0.6738
B27	HLA-B*27:09	999	1008	GRLQSLQTYV	0.5888
B27	HLA-B*27:09	1038	1049	KRVDFCGKGYHL	0.8281
B27	HLA-B*27:09	1106	1114	QRNFYEPQI	0.7026
B27	HLA-B*27:10	77	86	KRFDNPVLPF	0.9542
B27	HLA-B*27:10	236	244	TRFQTLLAL	0.9226
B27	HLA-B*27:10	453	461	YRLFRKSNL	0.5229
B27	HLA-B*27:13	77	86	KRFDNPVLPF	0.9904
B27	HLA-B*27:13	101	110	IRGWIFGTTL	0.6849
B27	HLA-B*27:13	235	244	ITRFQTLLAL	0.5227
B27	HLA-B*27:13	236	244	TRFQTLLAL	0.9782
B27	HLA-B*27:13	318	326	FRVQPTESI	0.6722
B27	HLA-B*27:13	327	335	VRFPNITNL	0.9818
B27	HLA-B*27:13	344	353	ATRFASVYAW	0.7783
B27	HLA-B*27:13	345	353	TRFASVYAW	0.9414
B27	HLA-B*27:13	356	365	KRISNCVADY	0.6725
B27	HLA-B*27:13	453	461	YRLFRKSNL	0.6377
B27	HLA-B*27:13	814	822	KRSFIEDLL	0.8024
B27	HLA-B*27:13	814	823	KRSFIEDLLF	0.9257
B27	HLA-B*27:13	846	855	ARDLICAQKF	0.5398
B27	HLA-B*27:13	904	913	YRFNGIGVTQ	0.7685
B27	HLA-B*27:13	999	1007	GRLQSLQTY	0.9825
B27	HLA-B*27:13	1038	1047	KRVDFCGKGY	0.8924
B27	HLA-B*27:13	1038	1049	KRVDFCGKGYHL	0.663
B27	HLA-B*27:15	77	86	KRFDNPVLPF	0.9827
B27	HLA-B*27:15	235	244	ITRFQTLLAL	0.5206
B27	HLA-B*27:15	236	244	TRFQTLLAL	0.9763
B27	HLA-B*27:15	327	335	VRFPNITNL	0.9756
B27	HLA-B*27:15	453	461	YRLFRKSNL	0.6738
B27	HLA-B*27:15	814	823	KRSFIEDLLF	0.8178
B27	HLA-B*27:15	904	912	YRFNGIGVT	0.6068
B27	HLA-B*27:15	999	1007	GRLQSLQTY	0.9613
B27	HLA-B*27:17	20	28	TRTQLPPAY	0.9
B27	HLA-B*27:17	77	84	KRFDNPVL	0.65
B27	HLA-B*27:17	77	86	KRFDNPVLPF	0.9915
B27	HLA-B*27:17	101	110	IRGWIFGTTL	0.7045
B27	HLA-B*27:17	235	244	ITRFQTLLAL	0.545
B27	HLA-B*27:17	236	244	TRFQTLLAL	0.9819
B27	HLA-B*27:17	318	326	FRVQPTESI	0.7209
B27	HLA-B*27:17	325	335	SIVRFPNITNL	0.8395
B27	HLA-B*27:17	326	335	IVRFPNITNL	0.8595
B27	HLA-B*27:17	327	335	VRFPNITNL	0.9862

B27	HLA-B*27:17	327	338	VRFPNITNLCPF	0.7682
B27	HLA-B*27:17	344	353	ATRFASVYAW	0.782
B27	HLA-B*27:17	345	353	TRFASVYAW	0.9514
B27	HLA-B*27:17	356	365	KRISNCVADY	0.6994
B27	HLA-B*27:17	453	461	YRLFRKSNL	0.6674
B27	HLA-B*27:17	508	515	YRVVVLSF	0.6075
B27	HLA-B*27:17	814	822	KRSFIEDLL	0.8281
B27	HLA-B*27:17	814	823	KRSFIEDLLF	0.9299
B27	HLA-B*27:17	846	855	ARDLICAQKF	0.5844
B27	HLA-B*27:17	904	911	YRFNGIGV	0.5341
B27	HLA-B*27:17	904	912	YRFNGIGVT	0.5417
B27	HLA-B*27:17	904	913	YRFNGIGVTQ	0.802
B27	HLA-B*27:17	998	1007	TGRLQSLQTY	0.6318
B27	HLA-B*27:17	999	1007	GRLQSLQTY	0.9859
B27	HLA-B*27:17	1038	1047	KRVDFCGKGY	0.9087
B27	HLA-B*27:17	1038	1049	KRVDFCGKGYHL	0.6876
B27	HLA-B*27:17	1106	1114	QRNFYEPQI	0.5937
B27	HLA-B*27:25	77	86	KRFDNPVLPF	0.8627
B27	HLA-B*27:25	236	244	TRFQTLLAL	0.8437
B27	HLA-B*27:28	77	86	KRFDNPVLPF	0.9752
B27	HLA-B*27:28	101	110	IRGWIFGTTL	0.6664
B27	HLA-B*27:28	236	244	TRFQTLLAL	0.9591
B27	HLA-B*27:28	327	335	VRFPNITNL	0.961
B27	HLA-B*27:28	344	353	ATRFASVYAW	0.754
B27	HLA-B*27:28	345	353	TRFASVYAW	0.9368
B27	HLA-B*27:28	356	365	KRISNCVADY	0.6169
B27	HLA-B*27:28	453	461	YRLFRKSNL	0.6569
B27	HLA-B*27:28	508	515	YRVVVLSF	0.6768
B27	HLA-B*27:28	814	823	KRSFIEDLLF	0.8481
B27	HLA-B*27:28	846	855	ARDLICAQKF	0.5402
B27	HLA-B*27:28	904	911	YRFNGIGV	0.5641
B27	HLA-B*27:28	904	913	YRFNGIGVTQ	0.7159
B27	HLA-B*27:28	999	1007	GRLQSLQTY	0.9569
B27	HLA-B*27:28	1038	1047	KRVDFCGKGY	0.8473
B27	HLA-B*38:01	236	244	TRFQTLLAL	0.761
B27	HLA-B*38:05	236	244	TRFQTLLAL	0.761
B27	HLA-B*38:09	236	244	TRFQTLLAL	0.761
B27	HLA-B*38:11	236	244	TRFQTLLAL	0.761
B27	HLA-B*39:01	236	244	TRFQTLLAL	0.9488
B27	HLA-B*39:01	318	326	FRVQPTESI	0.8358
B27	HLA-B*39:01	505	513	YQPYRVVVL	0.5961
B27	HLA-B*39:01	576	584	VRDPQTLEI	0.7426
B27	HLA-B*39:01	689	697	SQSIIAYTM	0.557
B27	HLA-B*39:01	764	772	NRALTGIAV	0.8592
B27	HLA-B*39:01	853	861	QKFNGLTVL	0.7764
B27	HLA-B*39:01	1158	1166	NHTSPDVDL	0.7288
B27	HLA-B*39:02	168	176	FEYVSQPFL	0.6813
B27	HLA-B*39:02	236	244	TRFQTLLAL	0.7521
B27	HLA-B*39:02	318	326	FRVQPTESI	0.588
B27	HLA-B*39:02	464	472	FERDISTEI	0.7304

B27	HLA-B*39:02	505	513	YQPYRVVVL	0.7112
B27	HLA-B*39:02	689	697	SQSIIAYTM	0.6777
B27	HLA-B*39:02	852	860	AQKFNGLTV	0.5032
B27	HLA-B*39:02	852	861	AQKFNGLTVL	0.6857
B27	HLA-B*39:02	853	861	QKFNGLTVL	0.8116
B27	HLA-B*39:02	894	902	LQIPFAMQM	0.6107
B27	HLA-B*39:02	1016	1024	AEIRASANL	0.7178
B27	HLA-B*39:02	1181	1189	KEIDRLNEV	0.7871
B27	HLA-B*39:04	236	244	TRFQTLLAL	0.9488
B27	HLA-B*39:04	318	326	FRVQPTESI	0.8358
B27	HLA-B*39:04	505	513	YQPYRVVVL	0.5961
B27	HLA-B*39:04	576	584	VRDPQTLEI	0.7426
B27	HLA-B*39:04	689	697	SQSIIAYTM	0.557
B27	HLA-B*39:04	764	772	NRALTGIAV	0.8592
B27	HLA-B*39:04	853	861	QKFNGLTVL	0.7764
B27	HLA-B*39:04	1158	1166	NHTSPDVDL	0.7288
B27	HLA-B*39:07	20	28	TRTQLPPAY	0.7614
B27	HLA-B*39:07	236	244	TRFQTLLAL	0.7603
B27	HLA-B*39:07	345	353	TRFASVYAW	0.7796
B27	HLA-B*39:07	465	473	ERDISTEIY	0.6857
B27	HLA-B*39:07	689	697	SQSIIAYTM	0.5704
B27	HLA-B*39:07	794	802	IKDFGGFNF	0.5866
B27	HLA-B*39:07	853	861	QKFNGLTVL	0.5625
B27	HLA-B*39:07	894	902	LQIPFAMQM	0.5211
B27	HLA-B*39:07	1087	1095	AHFPREGVF	0.6804
B27	HLA-B*39:09	48	56	LHSTQDLFL	0.5309
B27	HLA-B*39:09	236	244	TRFQTLLAL	0.9121
B27	HLA-B*39:09	269	277	YLQPRTFLL	0.5194
B27	HLA-B*39:09	318	326	FRVQPTESI	0.7813
B27	HLA-B*39:09	505	513	YQPYRVVVL	0.812
B27	HLA-B*39:09	689	697	SQSIIAYTM	0.6287
B27	HLA-B*39:09	764	772	NRALTGIAV	0.7776
B27	HLA-B*39:09	853	861	QKFNGLTVL	0.766
B27	HLA-B*39:09	1158	1166	NHTSPDVDL	0.7704
B27	HLA-B*39:14	236	244	TRFQTLLAL	0.9724
B27	HLA-B*39:14	318	326	FRVQPTESI	0.8722
B27	HLA-B*39:14	327	335	VRFPNITNL	0.9443
B27	HLA-B*39:14	453	461	YRLFRKSNL	0.7017
B27	HLA-B*39:14	505	513	YQPYRVVVL	0.7017
B27	HLA-B*39:14	576	584	VRDPQTLEI	0.769
B27	HLA-B*39:14	689	697	SQSIIAYTM	0.5743
B27	HLA-B*39:14	764	772	NRALTGIAV	0.865
B27	HLA-B*39:14	853	861	QKFNGLTVL	0.8612
B27	HLA-B*39:14	1158	1166	NHTSPDVDL	0.6977
B27	HLA-B*39:18	236	244	TRFQTLLAL	0.7919
B27	HLA-B*39:18	764	772	NRALTGIAV	0.7027
B27	HLA-B*39:23	168	176	FEYVSQPFL	0.6813
B27	HLA-B*39:23	236	244	TRFQTLLAL	0.7521
B27	HLA-B*39:23	318	326	FRVQPTESI	0.588
B27	HLA-B*39:23	464	472	FERDISTEI	0.7304

B27	HLA-B*39:23	505	513	YQPYRVVVL	0.7112
B27	HLA-B*39:23	689	697	SQSIIAYTM	0.6777
B27	HLA-B*39:23	852	860	AQKFNGLTV	0.5032
B27	HLA-B*39:23	852	861	AQKFNGLTVL	0.6857
B27	HLA-B*39:23	853	861	QKFNGLTVL	0.8116
B27	HLA-B*39:23	894	902	LQIPFAMQM	0.6107
B27	HLA-B*39:23	1016	1024	AEIRASANL	0.7178
B27	HLA-B*39:23	1181	1189	KEIDRLNEV	0.7871
B27	HLA-B*39:26	236	244	TRFQTLLAL	0.9488
B27	HLA-B*39:26	318	326	FRVQPTESI	0.8358
B27	HLA-B*39:26	505	513	YQPYRVVVL	0.5961
B27	HLA-B*39:26	576	584	VRDPQTLEI	0.7426
B27	HLA-B*39:26	689	697	SQSIIAYTM	0.557
B27	HLA-B*39:26	764	772	NRALTGIAV	0.8592
B27	HLA-B*39:26	853	861	QKFNGLTVL	0.7764
B27	HLA-B*39:26	1158	1166	NHTSPDVDL	0.7288
B27	HLA-B*39:27	236	244	TRFQTLLAL	0.9298
B27	HLA-B*39:27	318	326	FRVQPTESI	0.7781
B27	HLA-B*39:27	327	335	VRFPNITNL	0.8349
B27	HLA-B*39:27	505	513	YQPYRVVVL	0.5535
B27	HLA-B*39:27	576	584	VRDPQTLEI	0.726
B27	HLA-B*39:27	764	772	NRALTGIAV	0.8242
B27	HLA-B*39:27	853	861	QKFNGLTVL	0.6621
B27	HLA-B*39:27	1158	1166	NHTSPDVDL	0.5984
B27	HLA-B*39:29	236	244	TRFQTLLAL	0.9638
B27	HLA-B*39:29	318	326	FRVQPTESI	0.8376
B27	HLA-B*39:29	327	335	VRFPNITNL	0.9218
B27	HLA-B*39:29	453	461	YRLFRKSNL	0.6499
B27	HLA-B*39:29	505	513	YQPYRVVVL	0.5823
B27	HLA-B*39:29	764	772	NRALTGIAV	0.8145
B27	HLA-B*39:29	853	861	QKFNGLTVL	0.8131
B27	HLA-B*39:29	1158	1166	NHTSPDVDL	0.5748
B27	HLA-B*39:30	236	244	TRFQTLLAL	0.8953
B27	HLA-B*39:30	318	326	FRVQPTESI	0.6956
B27	HLA-B*39:30	764	772	NRALTGIAV	0.7463
B27	HLA-B*39:30	853	861	QKFNGLTVL	0.6386
B27	HLA-B*39:30	1158	1166	NHTSPDVDL	0.6332
B27	HLA-B*39:32	236	244	TRFQTLLAL	0.8554
B27	HLA-B*39:32	505	513	YQPYRVVVL	0.5179
B27	HLA-B*39:32	764	772	NRALTGIAV	0.7295
B27	HLA-B*39:32	1158	1166	NHTSPDVDL	0.5368
B27	HLA-B*40:12	168	176	FEYVSQPFL	0.5354
B27	HLA-B*40:12	689	697	SQSIIAYTM	0.5826
B27	HLA-B*40:12	852	861	AQKFNGLTVL	0.6787
B27	HLA-B*48:01	852	861	AQKFNGLTVL	0.6838
B27	HLA-B*48:02	22	32	TQLPPAYTNSF	0.5241
B27	HLA-B*48:02	77	86	KRFDNPVLPF	0.5598
B27	HLA-B*48:02	339	347	GEVFNATRF	0.7713
B27	HLA-B*48:02	345	353	TRFASVYAW	0.5657
B27	HLA-B*48:02	369	377	YNSASFSTF	0.5533

B27	HLA-B*48:02	413	421	GQTGKIADY	0.6026
B27	HLA-B*48:02	443	451	SKVGGNYNY	0.8498
B27	HLA-B*48:02	464	473	FERDISTEIY	0.6629
B27	HLA-B*48:02	497	505	FQPTNGVGY	0.6828
B27	HLA-B*48:02	557	565	KKFLPFQQF	0.7916
B27	HLA-B*48:02	687	695	VASQSIIAY	0.6788
B27	HLA-B*48:02	689	697	SQSIIAYTM	0.6856
B27	HLA-B*48:02	789	797	YKTPPIKDF	0.5888
B27	HLA-B*48:02	794	802	IKDFGGFNF	0.6372
B27	HLA-B*48:02	853	861	QKFNGLTVL	0.5314
B27	HLA-B*48:02	894	902	LQIPFAMQM	0.7465
B27	HLA-B*48:02	919	927	NQKLIANQF	0.7202
B27	HLA-B*48:02	999	1007	GRLQSLQTY	0.6453
B27	HLA-B*48:02	1054	1062	QSAPHGVVF	0.5772
B27	HLA-B*48:02	1087	1095	AHFPREGVF	0.5026
B27	HLA-B*48:02	1201	1209	QELGKYEQY	0.6932
B27	HLA-B*48:03	168	176	FEYVSQPFL	0.5354
B27	HLA-B*48:03	689	697	SQSIIAYTM	0.5826
B27	HLA-B*48:03	852	861	AQKFNGLTVL	0.6787
B27	HLA-B*48:04	852	861	AQKFNGLTVL	0.6838
B27	HLA-B*48:05	852	860	AQKFNGLTV	0.5272
B27	HLA-B*48:05	852	861	AQKFNGLTVL	0.5881
B27	HLA-B*48:09	852	861	AQKFNGLTVL	0.6838
B27	HLA-B*48:10	168	176	FEYVSQPFL	0.5246
B27	HLA-B*48:10	852	861	AQKFNGLTVL	0.6418
B27	HLA-B*48:10	853	861	QKFNGLTVL	0.614
B27	HLA-B*48:10	894	902	LQIPFAMQM	0.5173
B27	HLA-B*48:12	168	176	FEYVSQPFL	0.5246
B27	HLA-B*48:12	852	861	AQKFNGLTVL	0.6418
B27	HLA-B*48:12	853	861	QKFNGLTVL	0.614
B27	HLA-B*48:12	894	902	LQIPFAMQM	0.5173
B27	HLA-B*48:13	168	176	FEYVSQPFL	0.5246
B27	HLA-B*48:13	852	861	AQKFNGLTVL	0.6418
B27	HLA-B*48:13	853	861	QKFNGLTVL	0.614
B27	HLA-B*48:13	894	902	LQIPFAMQM	0.5173
B27	HLA-B*73:01	236	244	TRFQTLLAL	0.8088
B27	HLA-B*73:01	764	772	NRALTGIAV	0.6815
B44	HLA-B*15:53	153	160	MESEFRVY	0.5616
B44	HLA-B*15:53	339	347	GEVFNATRF	0.8089
B44	HLA-B*15:53	369	377	YNSASFSTF	0.5654
B44	HLA-B*15:53	413	421	GQTGKIADY	0.7749
B44	HLA-B*15:53	443	451	SKVGGNYNY	0.7828
B44	HLA-B*15:53	464	472	FERDISTEI	0.5031
B44	HLA-B*15:53	464	473	FERDISTEIY	0.8541
B44	HLA-B*15:53	497	505	FQPTNGVGY	0.7516
B44	HLA-B*15:53	557	565	KKFLPFQQF	0.6864
B44	HLA-B*15:53	634	643	RVYSTGSNVF	0.5726
B44	HLA-B*15:53	653	660	AEHVNNSY	0.6845
B44	HLA-B*15:53	687	695	VASQSIIAY	0.6214
B44	HLA-B*15:53	689	697	SQSIIAYTM	0.6354

B44	HLA-B*15:53	852	860	AQKFNGLTV	0.5366
B44	HLA-B*15:53	852	861	AQKFNGLTVL	0.5324
B44	HLA-B*15:53	894	902	LQIPFAMQM	0.7568
B44	HLA-B*15:53	919	927	NQKLIANQF	0.8277
B44	HLA-B*15:53	1016	1024	AEIRASANL	0.5785
B44	HLA-B*15:53	1054	1062	QSAPHGVVF	0.6409
B44	HLA-B*15:53	1264	1272	VLKGVKLHY	0.583
B44	HLA-B*18:01	153	160	MESEFRVY	0.8458
B44	HLA-B*18:01	168	175	FEYVSQPF	0.5811
B44	HLA-B*18:01	464	473	FERDISTEIY	0.7821
B44	HLA-B*18:01	724	731	TEILPVSM	0.6734
B44	HLA-B*18:01	1201	1209	QELGKYEQY	0.8379
B44	HLA-B*18:03	153	160	MESEFRVY	0.5628
B44	HLA-B*18:03	464	472	FERDISTEI	0.5105
B44	HLA-B*18:03	464	473	FERDISTEIY	0.5603
B44	HLA-B*18:05	153	160	MESEFRVY	0.8458
B44	HLA-B*18:05	168	175	FEYVSQPF	0.5811
B44	HLA-B*18:05	464	473	FERDISTEIY	0.7821
B44	HLA-B*18:05	724	731	TEILPVSM	0.6734
B44	HLA-B*18:05	1201	1209	QELGKYEQY	0.8379
B44	HLA-B*18:06	153	160	MESEFRVY	0.6821
B44	HLA-B*18:06	464	473	FERDISTEIY	0.5613
B44	HLA-B*18:10	153	160	MESEFRVY	0.6833
B44	HLA-B*18:10	339	347	GEVFNATRF	0.6957
B44	HLA-B*18:10	464	473	FERDISTEIY	0.7407
B44	HLA-B*18:10	724	731	TEILPVSM	0.6043
B44	HLA-B*18:10	1201	1209	QELGKYEQY	0.7825
B44	HLA-B*18:11	153	160	MESEFRVY	0.7741
B44	HLA-B*18:11	339	347	GEVFNATRF	0.7018
B44	HLA-B*18:11	464	472	FERDISTEI	0.5685
B44	HLA-B*18:11	464	473	FERDISTEIY	0.799
B44	HLA-B*18:11	724	731	TEILPVSM	0.6215
B44	HLA-B*18:11	1201	1209	QELGKYEQY	0.834
B44	HLA-B*18:13	153	160	MESEFRVY	0.8429
B44	HLA-B*18:13	168	175	FEYVSQPF	0.5593
B44	HLA-B*18:13	388	396	NDLCFTNVY	0.5383
B44	HLA-B*18:13	464	473	FERDISTEIY	0.7752
B44	HLA-B*18:13	1201	1209	QELGKYEQY	0.7889
B44	HLA-B*18:13	1206	1214	YEQYIKWPW	0.5226
B44	HLA-B*18:15	153	160	MESEFRVY	0.7703
B44	HLA-B*18:15	168	175	FEYVSQPF	0.5051
B44	HLA-B*18:15	464	473	FERDISTEIY	0.7513
B44	HLA-B*18:15	896	904	IPFAMQMAY	0.5009
B44	HLA-B*18:19	153	160	MESEFRVY	0.6571
B44	HLA-B*18:19	464	473	FERDISTEIY	0.725
B44	HLA-B*18:20	153	160	MESEFRVY	0.8458
B44	HLA-B*18:20	168	175	FEYVSQPF	0.5811
B44	HLA-B*18:20	464	473	FERDISTEIY	0.7821
B44	HLA-B*18:20	724	731	TEILPVSM	0.6734
B44	HLA-B*18:20	1201	1209	QELGKYEQY	0.8379

B44	HLA-B*37:01	168	176	FEYVSQPFL	0.6847
B44	HLA-B*37:01	1181	1189	KEIDRLNEV	0.8589
B44	HLA-B*37:04	168	176	FEYVSQPFL	0.6712
B44	HLA-B*40:01	168	176	FEYVSQPFL	0.9485
B44	HLA-B*40:01	168	177	FEYVSQPFLM	0.5398
B44	HLA-B*40:01	339	347	GEVFNATRF	0.8888
B44	HLA-B*40:01	464	472	FERDISTEI	0.9094
B44	HLA-B*40:01	660	670	YECDIPIGAGI	0.5931
B44	HLA-B*40:01	818	826	IEDLLFNKV	0.639
B44	HLA-B*40:01	987	996	VEAEVQIDRL	0.8709
B44	HLA-B*40:01	989	997	AEVQIDRLI	0.9071
B44	HLA-B*40:01	1016	1024	AEIRASANL	0.9748
B44	HLA-B*40:01	1181	1189	KEIDRLNEV	0.906
B44	HLA-B*40:01	1194	1203	NESLIDLQEL	0.5405
B44	HLA-B*40:01	1256	1265	FDEDDSEPVL	0.5944
B44	HLA-B*40:01	1261	1270	SEPVLKGVKL	0.736
B44	HLA-B*40:02	155	163	SEFRVYSSA	0.652
B44	HLA-B*40:02	168	176	FEYVSQPFL	0.8998
B44	HLA-B*40:02	190	197	REFVFKNI	0.6104
B44	HLA-B*40:02	339	347	GEVFNATRF	0.776
B44	HLA-B*40:02	464	472	FERDISTEI	0.8995
B44	HLA-B*40:02	660	668	YECDIPIGA	0.512
B44	HLA-B*40:02	724	731	TEILPVSM	0.8512
B44	HLA-B*40:02	818	826	IEDLLFNKV	0.6624
B44	HLA-B*40:02	987	996	VEAEVQIDRL	0.7072
B44	HLA-B*40:02	989	996	AEVQIDRL	0.7511
B44	HLA-B*40:02	989	997	AEVQIDRLI	0.8417
B44	HLA-B*40:02	1016	1024	AEIRASANL	0.9365
B44	HLA-B*40:02	1181	1189	KEIDRLNEV	0.9443
B44	HLA-B*40:02	1261	1270	SEPVLKGVKL	0.654
B44	HLA-B*40:05	155	163	SEFRVYSSA	0.5629
B44	HLA-B*40:05	168	176	FEYVSQPFL	0.7104
B44	HLA-B*40:05	464	472	FERDISTEI	0.8023
B44	HLA-B*40:05	1016	1024	AEIRASANL	0.8349
B44	HLA-B*40:05	1181	1189	KEIDRLNEV	0.879
B44	HLA-B*40:06	155	163	SEFRVYSSA	0.7601
B44	HLA-B*40:06	168	176	FEYVSQPFL	0.7587
B44	HLA-B*40:06	464	472	FERDISTEI	0.7272
B44	HLA-B*40:06	618	626	TEVPVAIHA	0.7591
B44	HLA-B*40:06	660	668	YECDIPIGA	0.6484
B44	HLA-B*40:06	818	826	IEDLLFNKV	0.6059
B44	HLA-B*40:06	1016	1024	AEIRASANL	0.6441
B44	HLA-B*40:06	1181	1189	KEIDRLNEV	0.765
B44	HLA-B*40:11	168	176	FEYVSQPFL	0.905
B44	HLA-B*40:11	339	347	GEVFNATRF	0.8092
B44	HLA-B*40:11	464	472	FERDISTEI	0.8897
B44	HLA-B*40:11	618	626	TEVPVAIHA	0.6448
B44	HLA-B*40:11	724	731	TEILPVSM	0.7985
B44	HLA-B*40:11	818	826	IEDLLFNKV	0.6927
B44	HLA-B*40:11	987	996	VEAEVQIDRL	0.7437

B44	HLA-B*40:11	989	996	AEVQIDRL	0.7049
B44	HLA-B*40:11	989	997	AEVQIDRLI	0.8855
B44	HLA-B*40:11	1016	1024	AEIRASANL	0.9391
B44	HLA-B*40:11	1181	1189	KEIDRLNEV	0.9057
B44	HLA-B*40:11	1256	1265	FDEDDSEPVL	0.5382
B44	HLA-B*40:11	1261	1270	SEPVLKGVKL	0.6019
B44	HLA-B*40:14	168	176	FEYVSQPFL	0.905
B44	HLA-B*40:14	339	347	GEVFNATRF	0.8092
B44	HLA-B*40:14	464	472	FERDISTEI	0.8897
B44	HLA-B*40:14	618	626	TEVPVAIHA	0.6448
B44	HLA-B*40:14	724	731	TEILPVSM	0.7985
B44	HLA-B*40:14	818	826	IEDLLFNKV	0.6927
B44	HLA-B*40:14	987	996	VEAEVQIDRL	0.7437
B44	HLA-B*40:14	989	996	AEVQIDRL	0.7049
B44	HLA-B*40:14	989	997	AEVQIDRLI	0.8855
B44	HLA-B*40:14	1016	1024	AEIRASANL	0.9391
B44	HLA-B*40:14	1181	1189	KEIDRLNEV	0.9057
B44	HLA-B*40:14	1256	1265	FDEDDSEPVL	0.5382
B44	HLA-B*40:14	1261	1270	SEPVLKGVKL	0.6019
B44	HLA-B*40:15	155	163	SEFRVYSSA	0.5585
B44	HLA-B*40:15	168	176	FEYVSQPFL	0.7239
B44	HLA-B*40:15	214	223	RDLPQGFSAL	0.5122
B44	HLA-B*40:15	464	472	FERDISTEI	0.8506
B44	HLA-B*40:15	1016	1024	AEIRASANL	0.8988
B44	HLA-B*40:15	1181	1189	KEIDRLNEV	0.8892
B44	HLA-B*40:16	155	163	SEFRVYSSA	0.5585
B44	HLA-B*40:16	168	176	FEYVSQPFL	0.7239
B44	HLA-B*40:16	214	223	RDLPQGFSAL	0.5122
B44	HLA-B*40:16	464	472	FERDISTEI	0.8506
B44	HLA-B*40:16	1016	1024	AEIRASANL	0.8988
B44	HLA-B*40:16	1181	1189	KEIDRLNEV	0.8892
B44	HLA-B*40:20	153	160	MESEFRVY	0.5689
B44	HLA-B*40:20	168	176	FEYVSQPFL	0.55
B44	HLA-B*40:20	190	200	REFVFKNIDGY	0.5832
B44	HLA-B*40:20	339	347	GEVFNATRF	0.906
B44	HLA-B*40:20	464	472	FERDISTEI	0.6148
B44	HLA-B*40:20	464	473	FERDISTEIY	0.806
B44	HLA-B*40:20	653	660	AEHVNNSY	0.7263
B44	HLA-B*40:20	724	731	TEILPVSM	0.5311
B44	HLA-B*40:20	989	997	AEVQIDRLI	0.6133
B44	HLA-B*40:20	1016	1024	AEIRASANL	0.7146
B44	HLA-B*40:20	1181	1189	KEIDRLNEV	0.6384
B44	HLA-B*40:20	1201	1209	QELGKYEQY	0.8567
B44	HLA-B*40:26	155	163	SEFRVYSSA	0.5119
B44	HLA-B*40:26	168	176	FEYVSQPFL	0.7736
B44	HLA-B*40:26	339	347	GEVFNATRF	0.7063
B44	HLA-B*40:26	464	472	FERDISTEI	0.8764
B44	HLA-B*40:26	724	731	TEILPVSM	0.6548
B44	HLA-B*40:26	989	997	AEVQIDRLI	0.7297
B44	HLA-B*40:26	1016	1024	AEIRASANL	0.9027

B44	HLA-B*40:26	1181	1189	KEIDRLNEV	0.9114
B44	HLA-B*40:29	155	163	SEFRVYSSA	0.6037
B44	HLA-B*40:29	168	176	FEYVSQPFL	0.838
B44	HLA-B*40:29	190	197	REFVFKNI	0.5309
B44	HLA-B*40:29	339	347	GEVFNATRF	0.7589
B44	HLA-B*40:29	464	472	FERDISTEI	0.8407
B44	HLA-B*40:29	724	731	TEILPVSM	0.764
B44	HLA-B*40:29	818	826	IEDLLFNKV	0.5439
B44	HLA-B*40:29	987	996	VEAEVQIDRL	0.6192
B44	HLA-B*40:29	989	996	AEVQIDRL	0.6685
B44	HLA-B*40:29	989	997	AEVQIDRLI	0.7966
B44	HLA-B*40:29	1016	1024	AEIRASANL	0.9162
B44	HLA-B*40:29	1181	1189	KEIDRLNEV	0.9308
B44	HLA-B*40:29	1261	1270	SEPVLKGVKL	0.5302
B44	HLA-B*40:35	155	163	SEFRVYSSA	0.652
B44	HLA-B*40:35	168	176	FEYVSQPFL	0.8998
B44	HLA-B*40:35	190	197	REFVFKNI	0.6104
B44	HLA-B*40:35	339	347	GEVFNATRF	0.776
B44	HLA-B*40:35	464	472	FERDISTEI	0.8995
B44	HLA-B*40:35	660	668	YECDIPIGA	0.512
B44	HLA-B*40:35	724	731	TEILPVSM	0.8512
B44	HLA-B*40:35	818	826	IEDLLFNKV	0.6624
B44	HLA-B*40:35	987	996	VEAEVQIDRL	0.7072
B44	HLA-B*40:35	989	996	AEVQIDRL	0.7511
B44	HLA-B*40:35	989	997	AEVQIDRLI	0.8417
B44	HLA-B*40:35	1016	1024	AEIRASANL	0.9365
B44	HLA-B*40:35	1181	1189	KEIDRLNEV	0.9443
B44	HLA-B*40:35	1261	1270	SEPVLKGVKL	0.654
B44	HLA-B*40:39	155	163	SEFRVYSSA	0.667
B44	HLA-B*40:39	168	176	FEYVSQPFL	0.8825
B44	HLA-B*40:39	190	197	REFVFKNI	0.6464
B44	HLA-B*40:39	339	347	GEVFNATRF	0.7646
B44	HLA-B*40:39	464	472	FERDISTEI	0.911
B44	HLA-B*40:39	660	668	YECDIPIGA	0.5358
B44	HLA-B*40:39	724	731	TEILPVSM	0.8562
B44	HLA-B*40:39	818	826	IEDLLFNKV	0.6636
B44	HLA-B*40:39	987	996	VEAEVQIDRL	0.6426
B44	HLA-B*40:39	989	996	AEVQIDRL	0.757
B44	HLA-B*40:39	989	997	AEVQIDRLI	0.8286
B44	HLA-B*40:39	1016	1024	AEIRASANL	0.9217
B44	HLA-B*40:39	1181	1189	KEIDRLNEV	0.944
B44	HLA-B*40:39	1261	1270	SEPVLKGVKL	0.621
B44	HLA-B*40:40	168	176	FEYVSQPFL	0.7627
B44	HLA-B*40:40	464	472	FERDISTEI	0.7123
B44	HLA-B*40:40	852	861	AQKFNGLTVL	0.5879
B44	HLA-B*40:40	1016	1024	AEIRASANL	0.7906
B44	HLA-B*40:40	1181	1189	KEIDRLNEV	0.8178
B44	HLA-B*40:49	168	176	FEYVSQPFL	0.7985
B44	HLA-B*40:49	689	697	SQSIIAYTM	0.587
B44	HLA-B*40:49	852	861	AQKFNGLTVL	0.6038

B44	HLA-B*40:49	1016	1024	AEIRASANL	0.8824
B44	HLA-B*40:50	168	176	FEYVSQPFL	0.7931
B44	HLA-B*40:50	464	472	FERDISTEI	0.8322
B44	HLA-B*40:50	724	731	TEILPVSM	0.5961
B44	HLA-B*40:50	989	997	AEVQIDRLI	0.69
B44	HLA-B*40:50	1016	1024	AEIRASANL	0.8418
B44	HLA-B*40:50	1181	1189	KEIDRLNEV	0.9058
B44	HLA-B*40:53	155	163	SEFRVYSSA	0.7601
B44	HLA-B*40:53	168	176	FEYVSQPFL	0.7587
B44	HLA-B*40:53	464	472	FERDISTEI	0.7272
B44	HLA-B*40:53	618	626	TEVPVAIHA	0.7591
B44	HLA-B*40:53	660	668	YECDIPIGA	0.6484
B44	HLA-B*40:53	818	826	IEDLLFNKV	0.6059
B44	HLA-B*40:53	1016	1024	AEIRASANL	0.6441
B44	HLA-B*40:53	1181	1189	KEIDRLNEV	0.765
B44	HLA-B*40:54	168	176	FEYVSQPFL	0.9485
B44	HLA-B*40:54	168	177	FEYVSQPFLM	0.5398
B44	HLA-B*40:54	339	347	GEVFNATRF	0.8888
B44	HLA-B*40:54	464	472	FERDISTEI	0.9094
B44	HLA-B*40:54	660	670	YECDIPIGAGI	0.5931
B44	HLA-B*40:54	818	826	IEDLLFNKV	0.639
B44	HLA-B*40:54	987	996	VEAEVQIDRL	0.8709
B44	HLA-B*40:54	989	997	AEVQIDRLI	0.9071
B44	HLA-B*40:54	1016	1024	AEIRASANL	0.9748
B44	HLA-B*40:54	1181	1189	KEIDRLNEV	0.906
B44	HLA-B*40:54	1194	1203	NESLIDLQEL	0.5405
B44	HLA-B*40:54	1256	1265	FDEDDSEPVL	0.5944
B44	HLA-B*40:54	1261	1270	SEPVLKGVKL	0.736
B44	HLA-B*40:55	168	176	FEYVSQPFL	0.9485
B44	HLA-B*40:55	168	177	FEYVSQPFLM	0.5398
B44	HLA-B*40:55	339	347	GEVFNATRF	0.8888
B44	HLA-B*40:55	464	472	FERDISTEI	0.9094
B44	HLA-B*40:55	660	670	YECDIPIGAGI	0.5931
B44	HLA-B*40:55	818	826	IEDLLFNKV	0.639
B44	HLA-B*40:55	987	996	VEAEVQIDRL	0.8709
B44	HLA-B*40:55	989	997	AEVQIDRLI	0.9071
B44	HLA-B*40:55	1016	1024	AEIRASANL	0.9748
B44	HLA-B*40:55	1181	1189	KEIDRLNEV	0.906
B44	HLA-B*40:55	1194	1203	NESLIDLQEL	0.5405
B44	HLA-B*40:55	1256	1265	FDEDDSEPVL	0.5944
B44	HLA-B*40:55	1261	1270	SEPVLKGVKL	0.736
B44	HLA-B*40:56	155	163	SEFRVYSSA	0.652
B44	HLA-B*40:56	168	176	FEYVSQPFL	0.8998
B44	HLA-B*40:56	190	197	REFVFKNI	0.6104
B44	HLA-B*40:56	339	347	GEVFNATRF	0.776
B44	HLA-B*40:56	464	472	FERDISTEI	0.8995
B44	HLA-B*40:56	660	668	YECDIPIGA	0.512
B44	HLA-B*40:56	724	731	TEILPVSM	0.8512
B44	HLA-B*40:56	818	826	IEDLLFNKV	0.6624
B44	HLA-B*40:56	987	996	VEAEVQIDRL	0.7072

B44	HLA-B*40:56	989	996	AEVQIDRL	0.7511
B44	HLA-B*40:56	989	997	AEVQIDRLI	0.8417
B44	HLA-B*40:56	1016	1024	AEIRASANL	0.9365
B44	HLA-B*40:56	1181	1189	KEIDRLNEV	0.9443
B44	HLA-B*40:56	1261	1270	SEPVLKGVKL	0.654
B44	HLA-B*40:57	155	163	SEFRVYSSA	0.652
B44	HLA-B*40:57	168	176	FEYVSQPFL	0.8998
B44	HLA-B*40:57	190	197	REFVFKNI	0.6104
B44	HLA-B*40:57	339	347	GEVFNATRF	0.776
B44	HLA-B*40:57	464	472	FERDISTEI	0.8995
B44	HLA-B*40:57	660	668	YECDIPIGA	0.512
B44	HLA-B*40:57	724	731	TEILPVSM	0.8512
B44	HLA-B*40:57	818	826	IEDLLFNKV	0.6624
B44	HLA-B*40:57	987	996	VEAEVQIDRL	0.7072
B44	HLA-B*40:57	989	996	AEVQIDRL	0.7511
B44	HLA-B*40:57	989	997	AEVQIDRLI	0.8417
B44	HLA-B*40:57	1016	1024	AEIRASANL	0.9365
B44	HLA-B*40:57	1181	1189	KEIDRLNEV	0.9443
B44	HLA-B*40:57	1261	1270	SEPVLKGVKL	0.654
B44	HLA-B*41:02	155	163	SEFRVYSSA	0.6407
B44	HLA-B*41:02	168	176	FEYVSQPFL	0.8406
B44	HLA-B*41:02	190	197	REFVFKNI	0.6992
B44	HLA-B*41:02	339	347	GEVFNATRF	0.7419
B44	HLA-B*41:02	464	472	FERDISTEI	0.9149
B44	HLA-B*41:02	724	731	TEILPVSM	0.8338
B44	HLA-B*41:02	818	826	IEDLLFNKV	0.7103
B44	HLA-B*41:02	987	996	VEAEVQIDRL	0.6717
B44	HLA-B*41:02	989	996	AEVQIDRL	0.7643
B44	HLA-B*41:02	989	997	AEVQIDRLI	0.847
B44	HLA-B*41:02	1016	1024	AEIRASANL	0.9273
B44	HLA-B*41:02	1181	1189	KEIDRLNEV	0.9555
B44	HLA-B*41:02	1261	1270	SEPVLKGVKL	0.7634
B44	HLA-B*41:03	168	176	FEYVSQPFL	0.8113
B44	HLA-B*41:03	339	347	GEVFNATRF	0.7437
B44	HLA-B*41:03	464	472	FERDISTEI	0.8985
B44	HLA-B*41:03	724	731	TEILPVSM	0.7399
B44	HLA-B*41:03	818	826	IEDLLFNKV	0.706
B44	HLA-B*41:03	987	996	VEAEVQIDRL	0.6522
B44	HLA-B*41:03	989	996	AEVQIDRL	0.6693
B44	HLA-B*41:03	989	997	AEVQIDRLI	0.8767
B44	HLA-B*41:03	1016	1024	AEIRASANL	0.9094
B44	HLA-B*41:03	1181	1189	KEIDRLNEV	0.9207
B44	HLA-B*41:03	1261	1270	SEPVLKGVKL	0.6936
B44	HLA-B*44:02	95	104	TEKSNIIRGW	0.9813
B44	HLA-B*44:02	153	160	MESEFRVY	0.5131
B44	HLA-B*44:02	297	306	SETKCTLKSF	0.7692
B44	HLA-B*44:02	339	347	GEVFNATRF	0.8968
B44	HLA-B*44:02	779	789	QEVFAQVKQIY	0.7113
B44	HLA-B*44:02	989	997	AEVQIDRLI	0.9059
B44	HLA-B*44:02	1016	1024	AEIRASANL	0.8002

B44	HLA-B*44:02	1091	1102	REGVFVSNGTHW	0.792
B44	HLA-B*44:02	1201	1209	QELGKYEQY	0.9415
B44	HLA-B*44:02	1201	1212	QELGKYEQYIKW	0.7097
B44	HLA-B*44:02	1206	1214	YEQYIKWPW	0.7519
B44	HLA-B*44:03	95	104	TEKSNIIRGW	0.9553
B44	HLA-B*44:03	153	160	MESEFRVY	0.5502
B44	HLA-B*44:03	297	306	SETKCTLKSF	0.7221
B44	HLA-B*44:03	339	347	GEVFNATRF	0.9332
B44	HLA-B*44:03	747	756	TECSNLLLQY	0.6025
B44	HLA-B*44:03	779	789	QEVFAQVKQIY	0.7054
B44	HLA-B*44:03	989	997	AEVQIDRLI	0.922
B44	HLA-B*44:03	1016	1024	AEIRASANL	0.8443
B44	HLA-B*44:03	1091	1102	REGVFVSNGTHW	0.7134
B44	HLA-B*44:03	1201	1209	QELGKYEQY	0.9687
B44	HLA-B*44:03	1201	1212	QELGKYEQYIKW	0.6195
B44	HLA-B*44:03	1206	1214	YEQYIKWPW	0.671
B44	HLA-B*44:04	95	104	TEKSNIIRGW	0.9072
B44	HLA-B*44:04	297	306	SETKCTLKSF	0.6094
B44	HLA-B*44:04	339	347	GEVFNATRF	0.8061
B44	HLA-B*44:04	747	756	TECSNLLLQY	0.5668
B44	HLA-B*44:04	989	997	AEVQIDRLI	0.7773
B44	HLA-B*44:04	1016	1024	AEIRASANL	0.6706
B44	HLA-B*44:04	1091	1102	REGVFVSNGTHW	0.6043
B44	HLA-B*44:04	1201	1209	QELGKYEQY	0.8836
B44	HLA-B*44:04	1206	1214	YEQYIKWPW	0.6802
B44	HLA-B*44:07	95	104	TEKSNIIRGW	0.9553
B44	HLA-B*44:07	153	160	MESEFRVY	0.5502
B44	HLA-B*44:07	297	306	SETKCTLKSF	0.7221
B44	HLA-B*44:07	339	347	GEVFNATRF	0.9332
B44	HLA-B*44:07	747	756	TECSNLLLQY	0.6025
B44	HLA-B*44:07	779	789	QEVFAQVKQIY	0.7054
B44	HLA-B*44:07	989	997	AEVQIDRLI	0.922
B44	HLA-B*44:07	1016	1024	AEIRASANL	0.8443
B44	HLA-B*44:07	1091	1102	REGVFVSNGTHW	0.7134
B44	HLA-B*44:07	1201	1209	QELGKYEQY	0.9687
B44	HLA-B*44:07	1201	1212	QELGKYEQYIKW	0.6195
B44	HLA-B*44:07	1206	1214	YEQYIKWPW	0.671
B44	HLA-B*44:13	95	104	TEKSNIIRGW	0.9553
B44	HLA-B*44:13	153	160	MESEFRVY	0.5502
B44	HLA-B*44:13	297	306	SETKCTLKSF	0.7221
B44	HLA-B*44:13	339	347	GEVFNATRF	0.9332
B44	HLA-B*44:13	747	756	TECSNLLLQY	0.6025
B44	HLA-B*44:13	779	789	QEVFAQVKQIY	0.7054
B44	HLA-B*44:13	989	997	AEVQIDRLI	0.922
B44	HLA-B*44:13	1016	1024	AEIRASANL	0.8443
B44	HLA-B*44:13	1091	1102	REGVFVSNGTHW	0.7134
B44	HLA-B*44:13	1201	1209	QELGKYEQY	0.9687
B44	HLA-B*44:13	1201	1212	QELGKYEQYIKW	0.6195
B44	HLA-B*44:13	1206	1214	YEQYIKWPW	0.671
B44	HLA-B*44:16	95	104	TEKSNIIRGW	0.8679

B44	HLA-B*44:16	153	160	MESEFRVY	0.5269
B44	HLA-B*44:16	190	200	REFVFKNIDGY	0.5278
B44	HLA-B*44:16	297	306	SETKCTLKSF	0.5822
B44	HLA-B*44:16	339	347	GEVFNATRF	0.8995
B44	HLA-B*44:16	464	473	FERDISTEIY	0.6219
B44	HLA-B*44:16	653	660	AEHVNNSY	0.6553
B44	HLA-B*44:16	747	756	TECSNLLLQY	0.5015
B44	HLA-B*44:16	779	789	QEVFAQVKQIY	0.5029
B44	HLA-B*44:16	829	837	ADAGFIKQY	0.8245
B44	HLA-B*44:16	917	927	YENQKLIANQF	0.5047
B44	HLA-B*44:16	989	997	AEVQIDRLI	0.7927
B44	HLA-B*44:16	1016	1024	AEIRASANL	0.745
B44	HLA-B*44:16	1091	1102	REGVFVSNGTHW	0.7416
B44	HLA-B*44:16	1181	1189	KEIDRLNEV	0.6515
B44	HLA-B*44:16	1201	1209	QELGKYEQY	0.8678
B44	HLA-B*44:16	1201	1212	QELGKYEQYIKW	0.5106
B44	HLA-B*44:16	1206	1214	YEQYIKWPW	0.6551
B44	HLA-B*44:21	95	104	TEKSNIIRGW	0.94
B44	HLA-B*44:21	297	306	SETKCTLKSF	0.6607
B44	HLA-B*44:21	339	347	GEVFNATRF	0.8236
B44	HLA-B*44:21	989	997	AEVQIDRLI	0.8099
B44	HLA-B*44:21	1016	1024	AEIRASANL	0.6996
B44	HLA-B*44:21	1091	1102	REGVFVSNGTHW	0.682
B44	HLA-B*44:21	1201	1209	QELGKYEQY	0.8862
B44	HLA-B*44:21	1201	1212	QELGKYEQYIKW	0.6123
B44	HLA-B*44:21	1206	1214	YEQYIKWPW	0.6936
B44	HLA-B*44:22	95	104	TEKSNIIRGW	0.9813
B44	HLA-B*44:22	153	160	MESEFRVY	0.5131
B44	HLA-B*44:22	297	306	SETKCTLKSF	0.7692
B44	HLA-B*44:22	339	347	GEVFNATRF	0.8968
B44	HLA-B*44:22	779	789	QEVFAQVKQIY	0.7113
B44	HLA-B*44:22	989	997	AEVQIDRLI	0.9059
B44	HLA-B*44:22	1016	1024	AEIRASANL	0.8002
B44	HLA-B*44:22	1091	1102	REGVFVSNGTHW	0.792
B44	HLA-B*44:22	1201	1209	QELGKYEQY	0.9415
B44	HLA-B*44:22	1201	1212	QELGKYEQYIKW	0.7097
B44	HLA-B*44:22	1206	1214	YEQYIKWPW	0.7519
B44	HLA-B*44:24	95	104	TEKSNIIRGW	0.9813
B44	HLA-B*44:24	153	160	MESEFRVY	0.5131
B44	HLA-B*44:24	297	306	SETKCTLKSF	0.7692
B44	HLA-B*44:24	339	347	GEVFNATRF	0.8968
B44	HLA-B*44:24	779	789	QEVFAQVKQIY	0.7113
B44	HLA-B*44:24	989	997	AEVQIDRLI	0.9059
B44	HLA-B*44:24	1016	1024	AEIRASANL	0.8002
B44	HLA-B*44:24	1091	1102	REGVFVSNGTHW	0.792
B44	HLA-B*44:24	1201	1209	QELGKYEQY	0.9415
B44	HLA-B*44:24	1201	1212	QELGKYEQYIKW	0.7097
B44	HLA-B*44:24	1206	1214	YEQYIKWPW	0.7519
B44	HLA-B*44:26	95	104	TEKSNIIRGW	0.9553
B44	HLA-B*44:26	153	160	MESEFRVY	0.5502

B44	HLA-B*44:26	297	306	SETKCTLKSF	0.7221
B44	HLA-B*44:26	339	347	GEVFNATRF	0.9332
B44	HLA-B*44:26	747	756	TECSNLLLQY	0.6025
B44	HLA-B*44:26	779	789	QEVFAQVKQIY	0.7054
B44	HLA-B*44:26	989	997	AEVQIDRLI	0.922
B44	HLA-B*44:26	1016	1024	AEIRASANL	0.8443
B44	HLA-B*44:26	1091	1102	REGVFVSNGTHW	0.7134
B44	HLA-B*44:26	1201	1209	QELGKYEQY	0.9687
B44	HLA-B*44:26	1201	1212	QELGKYEQYIKW	0.6195
B44	HLA-B*44:26	1206	1214	YEQYIKWPW	0.671
B44	HLA-B*44:27	95	104	TEKSNIIRGW	0.9813
B44	HLA-B*44:27	153	160	MESEFRVY	0.5131
B44	HLA-B*44:27	297	306	SETKCTLKSF	0.7692
B44	HLA-B*44:27	339	347	GEVFNATRF	0.8968
B44	HLA-B*44:27	779	789	QEVFAQVKQIY	0.7113
B44	HLA-B*44:27	989	997	AEVQIDRLI	0.9059
B44	HLA-B*44:27	1016	1024	AEIRASANL	0.8002
B44	HLA-B*44:27	1091	1102	REGVFVSNGTHW	0.792
B44	HLA-B*44:27	1201	1209	QELGKYEQY	0.9415
B44	HLA-B*44:27	1201	1212	QELGKYEQYIKW	0.7097
B44	HLA-B*44:27	1206	1214	YEQYIKWPW	0.7519
B44	HLA-B*44:28	95	104	TEKSNIIRGW	0.9464
B44	HLA-B*44:28	297	306	SETKCTLKSF	0.6716
B44	HLA-B*44:28	339	347	GEVFNATRF	0.8558
B44	HLA-B*44:28	747	756	TECSNLLLQY	0.5764
B44	HLA-B*44:28	989	997	AEVQIDRLI	0.8322
B44	HLA-B*44:28	1016	1024	AEIRASANL	0.7302
B44	HLA-B*44:28	1091	1102	REGVFVSNGTHW	0.6683
B44	HLA-B*44:28	1201	1209	QELGKYEQY	0.92
B44	HLA-B*44:28	1201	1212	QELGKYEQYIKW	0.5544
B44	HLA-B*44:28	1206	1214	YEQYIKWPW	0.7125
B44	HLA-B*44:29	95	104	TEKSNIIRGW	0.9629
B44	HLA-B*44:29	153	160	MESEFRVY	0.6747
B44	HLA-B*44:29	190	200	REFVFKNIDGY	0.5841
B44	HLA-B*44:29	297	306	SETKCTLKSF	0.8275
B44	HLA-B*44:29	339	347	GEVFNATRF	0.9564
B44	HLA-B*44:29	464	473	FERDISTEIY	0.7133
B44	HLA-B*44:29	653	660	AEHVNNSY	0.8118
B44	HLA-B*44:29	747	756	TECSNLLLQY	0.7163
B44	HLA-B*44:29	779	789	QEVFAQVKQIY	0.8073
B44	HLA-B*44:29	829	837	ADAGFIKQY	0.8988
B44	HLA-B*44:29	917	927	YENQKLIANQF	0.5716
B44	HLA-B*44:29	989	997	AEVQIDRLI	0.9447
B44	HLA-B*44:29	1016	1024	AEIRASANL	0.8969
B44	HLA-B*44:29	1091	1102	REGVFVSNGTHW	0.8063
B44	HLA-B*44:29	1201	1209	QELGKYEQY	0.9796
B44	HLA-B*44:29	1201	1212	QELGKYEQYIKW	0.6973
B44	HLA-B*44:29	1206	1214	YEQYIKWPW	0.7407
B44	HLA-B*44:30	95	104	TEKSNIIRGW	0.9553
B44	HLA-B*44:30	153	160	MESEFRVY	0.5502

B44	HLA-B*44:30	297	306	SETKCTLKSF	0.7221
B44	HLA-B*44:30	339	347	GEVFNATRF	0.9332
B44	HLA-B*44:30	747	756	TECSNLLLQY	0.6025
B44	HLA-B*44:30	779	789	QEVFAQVKQIY	0.7054
B44	HLA-B*44:30	989	997	AEVQIDRLI	0.922
B44	HLA-B*44:30	1016	1024	AEIRASANL	0.8443
B44	HLA-B*44:30	1091	1102	REGVFVSNGTHW	0.7134
B44	HLA-B*44:30	1201	1209	QELGKYEQY	0.9687
B44	HLA-B*44:30	1201	1212	QELGKYEQYIKW	0.6195
B44	HLA-B*44:30	1206	1214	YEQYIKWPW	0.671
B44	HLA-B*44:32	95	104	TEKSNIIRGW	0.938
B44	HLA-B*44:32	297	306	SETKCTLKSF	0.6498
B44	HLA-B*44:32	339	347	GEVFNATRF	0.8844
B44	HLA-B*44:32	747	756	TECSNLLLQY	0.513
B44	HLA-B*44:32	779	789	QEVFAQVKQIY	0.6238
B44	HLA-B*44:32	989	997	AEVQIDRLI	0.8801
B44	HLA-B*44:32	1016	1024	AEIRASANL	0.796
B44	HLA-B*44:32	1091	1102	REGVFVSNGTHW	0.7563
B44	HLA-B*44:32	1201	1209	QELGKYEQY	0.9492
B44	HLA-B*44:32	1201	1212	QELGKYEQYIKW	0.5458
B44	HLA-B*44:32	1206	1214	YEQYIKWPW	0.6216
B44	HLA-B*44:33	95	104	TEKSNIIRGW	0.9813
B44	HLA-B*44:33	153	160	MESEFRVY	0.5131
B44	HLA-B*44:33	297	306	SETKCTLKSF	0.7692
B44	HLA-B*44:33	339	347	GEVFNATRF	0.8968
B44	HLA-B*44:33	779	789	QEVFAQVKQIY	0.7113
B44	HLA-B*44:33	989	997	AEVQIDRLI	0.9059
B44	HLA-B*44:33	1016	1024	AEIRASANL	0.8002
B44	HLA-B*44:33	1091	1102	REGVFVSNGTHW	0.792
B44	HLA-B*44:33	1201	1209	QELGKYEQY	0.9415
B44	HLA-B*44:33	1201	1212	QELGKYEQYIKW	0.7097
B44	HLA-B*44:33	1206	1214	YEQYIKWPW	0.7519
B44	HLA-B*44:35	95	104	TEKSNIIRGW	0.975
B44	HLA-B*44:35	153	160	MESEFRVY	0.5617
B44	HLA-B*44:35	297	306	SETKCTLKSF	0.7874
B44	HLA-B*44:35	339	347	GEVFNATRF	0.9155
B44	HLA-B*44:35	747	756	TECSNLLLQY	0.5932
B44	HLA-B*44:35	779	789	QEVFAQVKQIY	0.7582
B44	HLA-B*44:35	989	997	AEVQIDRLI	0.9053
B44	HLA-B*44:35	1016	1024	AEIRASANL	0.8196
B44	HLA-B*44:35	1091	1102	REGVFVSNGTHW	0.782
B44	HLA-B*44:35	1201	1209	QELGKYEQY	0.9534
B44	HLA-B*44:35	1201	1212	QELGKYEQYIKW	0.6824
B44	HLA-B*44:35	1206	1214	YEQYIKWPW	0.7492
B44	HLA-B*44:36	95	104	TEKSNIIRGW	0.9553
B44	HLA-B*44:36	153	160	MESEFRVY	0.5502
B44	HLA-B*44:36	297	306	SETKCTLKSF	0.7221
B44	HLA-B*44:36	339	347	GEVFNATRF	0.9332
B44	HLA-B*44:36	747	756	TECSNLLLQY	0.6025
B44	HLA-B*44:36	779	789	QEVFAQVKQIY	0.7054

B44	HLA-B*44:36	989	997	AEVQIDRLI	0.922
B44	HLA-B*44:36	1016	1024	AEIRASANL	0.8443
B44	HLA-B*44:36	1091	1102	REGVFVSNGTHW	0.7134
B44	HLA-B*44:36	1201	1209	QELGKYEQY	0.9687
B44	HLA-B*44:36	1201	1212	QELGKYEQYIKW	0.6195
B44	HLA-B*44:36	1206	1214	YEQYIKWPW	0.671
B44	HLA-B*44:37	95	104	TEKSNIIRGW	0.826
B44	HLA-B*44:37	153	160	MESEFRVY	0.5576
B44	HLA-B*44:37	190	200	REFVFKNIDGY	0.5135
B44	HLA-B*44:37	339	347	GEVFNATRF	0.9143
B44	HLA-B*44:37	464	473	FERDISTEIY	0.7083
B44	HLA-B*44:37	829	837	ADAGFIKQY	0.8697
B44	HLA-B*44:37	989	997	AEVQIDRLI	0.7629
B44	HLA-B*44:37	1016	1024	AEIRASANL	0.7138
B44	HLA-B*44:37	1091	1102	REGVFVSNGTHW	0.6777
B44	HLA-B*44:37	1201	1209	QELGKYEQY	0.902
B44	HLA-B*44:37	1206	1214	YEQYIKWPW	0.5676
B44	HLA-B*44:38	95	104	TEKSNIIRGW	0.9553
B44	HLA-B*44:38	153	160	MESEFRVY	0.5502
B44	HLA-B*44:38	297	306	SETKCTLKSF	0.7221
B44	HLA-B*44:38	339	347	GEVFNATRF	0.9332
B44	HLA-B*44:38	747	756	TECSNLLLQY	0.6025
B44	HLA-B*44:38	779	789	QEVFAQVKQIY	0.7054
B44	HLA-B*44:38	989	997	AEVQIDRLI	0.922
B44	HLA-B*44:38	1016	1024	AEIRASANL	0.8443
B44	HLA-B*44:38	1091	1102	REGVFVSNGTHW	0.7134
B44	HLA-B*44:38	1201	1209	QELGKYEQY	0.9687
B44	HLA-B*44:38	1201	1212	QELGKYEQYIKW	0.6195
B44	HLA-B*44:38	1206	1214	YEQYIKWPW	0.671
B44	HLA-B*45:01	153	161	MESEFRVYS	0.6419
B44	HLA-B*45:01	155	163	SEFRVYSSA	0.8907
B44	HLA-B*45:01	280	288	NENGTITDA	0.7132
B44	HLA-B*45:01	618	626	TEVPVAIHA	0.9036
B44	HLA-B*45:01	867	876	DEMIAQYTSA	0.5083
B44	HLA-B*45:01	1016	1024	AEIRASANL	0.678
B44	HLA-B*45:01	1016	1025	AEIRASANLA	0.8233
B44	HLA-B*45:01	1016	1026	AEIRASANLAA	0.6215
B44	HLA-B*45:01	1071	1079	QEKNFTTAP	0.5423
B44	HLA-B*45:01	1071	1080	QEKNFTTAPA	0.5531
B44	HLA-B*45:01	1181	1189	KEIDRLNEV	0.7707
B44	HLA-B*45:01	1181	1190	KEIDRLNEVA	0.6837
B44	HLA-B*45:03	153	161	MESEFRVYS	0.6419
B44	HLA-B*45:03	155	163	SEFRVYSSA	0.8907
B44	HLA-B*45:03	280	288	NENGTITDA	0.7132
B44	HLA-B*45:03	618	626	TEVPVAIHA	0.9036
B44	HLA-B*45:03	867	876	DEMIAQYTSA	0.5083
B44	HLA-B*45:03	1016	1024	AEIRASANL	0.678
B44	HLA-B*45:03	1016	1025	AEIRASANLA	0.8233
B44	HLA-B*45:03	1016	1026	AEIRASANLAA	0.6215
B44	HLA-B*45:03	1071	1079	QEKNFTTAP	0.5423

B44	HLA-B*45:03	1071	1080	QEKNFTTAPA	0.5531
B44	HLA-B*45:03	1181	1189	KEIDRLNEV	0.7707
B44	HLA-B*45:03	1181	1190	KEIDRLNEVA	0.6837
B44	HLA-B*45:04	153	161	MESEFRVYS	0.6668
B44	HLA-B*45:04	155	163	SEFRVYSSA	0.8728
B44	HLA-B*45:04	168	176	FEYVSQPFL	0.6477
B44	HLA-B*45:04	280	288	NENGTITDA	0.6169
B44	HLA-B*45:04	339	348	GEVFNATRFA	0.5181
B44	HLA-B*45:04	464	472	FERDISTEI	0.7909
B44	HLA-B*45:04	515	523	FELLHAPAT	0.6115
B44	HLA-B*45:04	618	626	TEVPVAIHA	0.9037
B44	HLA-B*45:04	660	668	YECDIPIGA	0.7754
B44	HLA-B*45:04	724	732	TEILPVSMT	0.6427
B44	HLA-B*45:04	818	826	IEDLLFNKV	0.696
B44	HLA-B*45:04	989	997	AEVQIDRLI	0.7778
B44	HLA-B*45:04	1016	1024	AEIRASANL	0.7431
B44	HLA-B*45:04	1016	1025	AEIRASANLA	0.7358
B44	HLA-B*45:04	1016	1026	AEIRASANLAA	0.6111
B44	HLA-B*45:04	1181	1189	KEIDRLNEV	0.8925
B44	HLA-B*45:04	1181	1190	KEIDRLNEVA	0.783
B44	HLA-B*45:05	153	161	MESEFRVYS	0.7073
B44	HLA-B*45:05	155	163	SEFRVYSSA	0.887
B44	HLA-B*45:05	280	288	NENGTITDA	0.7827
B44	HLA-B*45:05	280	289	NENGTITDAV	0.5501
B44	HLA-B*45:05	339	348	GEVFNATRFA	0.5214
B44	HLA-B*45:05	618	626	TEVPVAIHA	0.9095
B44	HLA-B*45:05	660	668	YECDIPIGA	0.6478
B44	HLA-B*45:05	867	875	DEMIAQYTS	0.5654
B44	HLA-B*45:05	867	876	DEMIAQYTSA	0.6146
B44	HLA-B*45:05	989	997	AEVQIDRLI	0.7684
B44	HLA-B*45:05	1016	1024	AEIRASANL	0.6972
B44	HLA-B*45:05	1016	1025	AEIRASANLA	0.8381
B44	HLA-B*45:05	1016	1026	AEIRASANLAA	0.6471
B44	HLA-B*45:05	1071	1079	QEKNFTTAP	0.638
B44	HLA-B*45:05	1071	1080	QEKNFTTAPA	0.6366
B44	HLA-B*45:05	1181	1189	KEIDRLNEV	0.7759
B44	HLA-B*45:05	1181	1190	KEIDRLNEVA	0.7116
B44	HLA-B*45:07	153	161	MESEFRVYS	0.6419
B44	HLA-B*45:07	155	163	SEFRVYSSA	0.8907
B44	HLA-B*45:07	280	288	NENGTITDA	0.7132
B44	HLA-B*45:07	618	626	TEVPVAIHA	0.9036
B44	HLA-B*45:07	867	876	DEMIAQYTSA	0.5083
B44	HLA-B*45:07	1016	1024	AEIRASANL	0.678
B44	HLA-B*45:07	1016	1025	AEIRASANLA	0.8233
B44	HLA-B*45:07	1016	1026	AEIRASANLAA	0.6215
B44	HLA-B*45:07	1071	1079	QEKNFTTAP	0.5423
B44	HLA-B*45:07	1071	1080	QEKNFTTAPA	0.5531
B44	HLA-B*45:07	1181	1189	KEIDRLNEV	0.7707
B44	HLA-B*45:07	1181	1190	KEIDRLNEVA	0.6837
B44	HLA-B*49:04	339	347	GEVFNATRF	0.7684

B44	HLA-B*49:04	464	472	FERDISTEI	0.6468
B44	HLA-B*49:04	989	997	AEVQIDRLI	0.7729
B44	HLA-B*50:01	153	161	MESEFRVYS	0.5279
B44	HLA-B*50:01	155	163	SEFRVYSSA	0.9313
B44	HLA-B*50:01	168	176	FEYVSQPFL	0.5682
B44	HLA-B*50:01	464	472	FERDISTEI	0.8164
B44	HLA-B*50:01	515	523	FELLHAPAT	0.5692
B44	HLA-B*50:01	618	626	TEVPVAIHA	0.8958
B44	HLA-B*50:01	660	668	YECDIPIGA	0.7228
B44	HLA-B*50:01	1016	1024	AEIRASANL	0.635
B44	HLA-B*50:01	1016	1025	AEIRASANLA	0.6321
B44	HLA-B*50:01	1181	1189	KEIDRLNEV	0.8625
B44	HLA-B*50:01	1181	1190	KEIDRLNEVA	0.7661
B44	HLA-B*50:02	155	163	SEFRVYSSA	0.8534
B44	HLA-B*50:02	280	288	NENGTITDA	0.5939
B44	HLA-B*50:02	618	626	TEVPVAIHA	0.8364
B44	HLA-B*50:02	1016	1025	AEIRASANLA	0.6537
B44	HLA-B*50:02	1181	1190	KEIDRLNEVA	0.5821
B44	HLA-B*50:04	153	161	MESEFRVYS	0.5279
B44	HLA-B*50:04	155	163	SEFRVYSSA	0.9313
B44	HLA-B*50:04	168	176	FEYVSQPFL	0.5682
B44	HLA-B*50:04	464	472	FERDISTEI	0.8164
B44	HLA-B*50:04	515	523	FELLHAPAT	0.5692
B44	HLA-B*50:04	618	626	TEVPVAIHA	0.8958
B44	HLA-B*50:04	660	668	YECDIPIGA	0.7228
B44	HLA-B*50:04	1016	1024	AEIRASANL	0.635
B44	HLA-B*50:04	1016	1025	AEIRASANLA	0.6321
B44	HLA-B*50:04	1181	1189	KEIDRLNEV	0.8625
B44	HLA-B*50:04	1181	1190	KEIDRLNEVA	0.7661
B58	HLA-B*15:16	34	43	RGVYYPDKVF	0.6031
B58	HLA-B*15:16	50	58	STQDLFLPF	0.5575
B58	HLA-B*15:16	160	168	YSSANNCTF	0.7436
B58	HLA-B*15:16	258	266	WTAGAAAYY	0.6769
B58	HLA-B*15:16	267	275	VGYLQPRTF	0.6255
B58	HLA-B*15:16	304	312	KSFTVEKGI	0.6627
B58	HLA-B*15:16	344	353	ATRFASVYAW	0.5424
B58	HLA-B*15:16	372	380	ASFSTFKCY	0.5797
B58	HLA-B*15:16	392	400	FTNVYADSF	0.6268
B58	HLA-B*15:16	590	598	CSFGGVSVI	0.5898
B58	HLA-B*15:16	604	612	TSNQVAVLY	0.7291
B58	HLA-B*15:16	634	642	RVYSTGSNV	0.5284
B58	HLA-B*15:16	634	643	RVYSTGSNVF	0.7452
B58	HLA-B*15:16	685	693	RSVASQSII	0.7268
B58	HLA-B*15:16	687	695	VASQSIIAY	0.7282
B58	HLA-B*15:16	710	718	NSIAIPTNF	0.7782
B58	HLA-B*15:16	712	720	IAIPTNFTI	0.8768
B58	HLA-B*15:16	718	726	FTISVTTEI	0.7002
B58	HLA-B*15:16	733	741	KTSVDCTMY	0.5204
B58	HLA-B*15:16	814	823	KRSFIEDLLF	0.6401
B58	HLA-B*15:16	815	823	RSFIEDLLF	0.9347

B58	HLA-B*15:16	825	833	KVTLADAGF	0.5684
B58	HLA-B*15:16	865	873	LTDEMIAQY	0.7522
B58	HLA-B*15:16	878	886	LAGTITSGW	0.5789
B58	HLA-B*15:16	879	888	AGTITSGWTF	0.542
B58	HLA-B*15:16	880	888	GTITSGWTF	0.8294
B58	HLA-B*15:16	886	894	WTFGAGAAL	0.5705
B58	HLA-B*15:16	898	906	FAMQMAYRF	0.655
B58	HLA-B*15:16	923	931	IANQFNSAI	0.5744
B58	HLA-B*15:16	1005	1013	QTYVTQQLI	0.6943
B58	HLA-B*15:16	1054	1062	QSAPHGVVF	0.8856
B58	HLA-B*15:16	1086	1095	KAHFPREGVF	0.5268
B58	HLA-B*15:16	1093	1102	GVFVSNGTHW	0.5731
B58	HLA-B*15:17	21	28	RTQLPPAY	0.729
B58	HLA-B*15:17	28	37	YTNSFTRGVY	0.712
B58	HLA-B*15:17	29	38	TNSFTRGVYY	0.6428
B58	HLA-B*15:17	30	38	NSFTRGVYY	0.8886
B58	HLA-B*15:17	34	43	RGVYYPDKVF	0.7986
B58	HLA-B*15:17	35	43	GVYYPDKVF	0.8911
B58	HLA-B*15:17	50	58	STQDLFLPF	0.7926
B58	HLA-B*15:17	158	168	RVYSSANNCTF	0.6804
B58	HLA-B*15:17	160	168	YSSANNCTF	0.8168
B58	HLA-B*15:17	192	200	FVFKNIDGY	0.8376
B58	HLA-B*15:17	204	212	YSKHTPINL	0.7655
B58	HLA-B*15:17	212	220	LVRDLPQGF	0.867
B58	HLA-B*15:17	257	266	GWTAGAAAYY	0.6825
B58	HLA-B*15:17	258	266	WTAGAAAYY	0.9236
B58	HLA-B*15:17	261	269	GAAAYYVGY	0.8951
B58	HLA-B*15:17	267	275	VGYLQPRTF	0.7451
B58	HLA-B*15:17	304	312	KSFTVEKGI	0.6634
B58	HLA-B*15:17	304	313	KSFTVEKGIY	0.7603
B58	HLA-B*15:17	310	318	KGIYQTSNF	0.719
B58	HLA-B*15:17	344	353	ATRFASVYAW	0.7702
B58	HLA-B*15:17	361	369	CVADYSVLY	0.6496
B58	HLA-B*15:17	366	374	SVLYNSASF	0.8204
B58	HLA-B*15:17	372	380	ASFSTFKCY	0.828
B58	HLA-B*15:17	392	400	FTNVYADSF	0.7806
B58	HLA-B*15:17	604	612	TSNQVAVLY	0.9455
B58	HLA-B*15:17	625	633	HADQLTPTW	0.846
B58	HLA-B*15:17	634	642	RVYSTGSNV	0.6546
B58	HLA-B*15:17	634	643	RVYSTGSNVF	0.9115
B58	HLA-B*15:17	685	693	RSVASQSII	0.7537
B58	HLA-B*15:17	687	695	VASQSIIAY	0.9477
B58	HLA-B*15:17	710	718	NSIAIPTNF	0.9195
B58	HLA-B*15:17	712	720	IAIPTNFTI	0.867
B58	HLA-B*15:17	718	726	FTISVTTEI	0.7184
B58	HLA-B*15:17	733	741	KTSVDCTMY	0.8517
B58	HLA-B*15:17	814	823	KRSFIEDLLF	0.7282
B58	HLA-B*15:17	815	823	RSFIEDLLF	0.9846
B58	HLA-B*15:17	825	833	KVTLADAGF	0.8775
B58	HLA-B*15:17	865	873	LTDEMIAQY	0.9166

B58	HLA-B*15:17	878	886	LAGTITSGW	0.7922
B58	HLA-B*15:17	879	888	AGTITSGWTF	0.7247
B58	HLA-B*15:17	880	888	GTITSGWTF	0.9585
B58	HLA-B*15:17	886	894	WTFGAGAAL	0.7745
B58	HLA-B*15:17	892	900	AALQIPFAM	0.6696
B58	HLA-B*15:17	898	906	FAMQMAYRF	0.7486
B58	HLA-B*15:17	940	948	STASALGKL	0.7384
B58	HLA-B*15:17	962	970	LVKQLSSNF	0.7867
B58	HLA-B*15:17	1005	1013	QTYVTQQLI	0.7371
B58	HLA-B*15:17	1021	1029	SANLAATKM	0.6738
B58	HLA-B*15:17	1054	1062	QSAPHGVVF	0.9815
B58	HLA-B*15:17	1086	1095	KAHFPREGVF	0.7038
B58	HLA-B*15:17	1093	1102	GVFVSNGTHW	0.7853
B58	HLA-B*15:17	1095	1103	FVSNGTHWF	0.6814
B58	HLA-B*15:67	34	43	RGVYYPDKVF	0.6031
B58	HLA-B*15:67	50	58	STQDLFLPF	0.5575
B58	HLA-B*15:67	160	168	YSSANNCTF	0.7436
B58	HLA-B*15:67	258	266	WTAGAAAYY	0.6769
B58	HLA-B*15:67	267	275	VGYLQPRTF	0.6255
B58	HLA-B*15:67	304	312	KSFTVEKGI	0.6627
B58	HLA-B*15:67	344	353	ATRFASVYAW	0.5424
B58	HLA-B*15:67	372	380	ASFSTFKCY	0.5797
B58	HLA-B*15:67	392	400	FTNVYADSF	0.6268
B58	HLA-B*15:67	590	598	CSFGGVSVI	0.5898
B58	HLA-B*15:67	604	612	TSNQVAVLY	0.7291
B58	HLA-B*15:67	634	642	RVYSTGSNV	0.5284
B58	HLA-B*15:67	634	643	RVYSTGSNVF	0.7452
B58	HLA-B*15:67	685	693	RSVASQSII	0.7268
B58	HLA-B*15:67	687	695	VASQSIIAY	0.7282
B58	HLA-B*15:67	710	718	NSIAIPTNF	0.7782
B58	HLA-B*15:67	712	720	IAIPTNFTI	0.8768
B58	HLA-B*15:67	718	726	FTISVTTEI	0.7002
B58	HLA-B*15:67	733	741	KTSVDCTMY	0.5204
B58	HLA-B*15:67	814	823	KRSFIEDLLF	0.6401
B58	HLA-B*15:67	815	823	RSFIEDLLF	0.9347
B58	HLA-B*15:67	825	833	KVTLADAGF	0.5684
B58	HLA-B*15:67	865	873	LTDEMIAQY	0.7522
B58	HLA-B*15:67	878	886	LAGTITSGW	0.5789
B58	HLA-B*15:67	879	888	AGTITSGWTF	0.542
B58	HLA-B*15:67	880	888	GTITSGWTF	0.8294
B58	HLA-B*15:67	886	894	WTFGAGAAL	0.5705
B58	HLA-B*15:67	898	906	FAMQMAYRF	0.655
B58	HLA-B*15:67	923	931	IANQFNSAI	0.5744
B58	HLA-B*15:67	1005	1013	QTYVTQQLI	0.6943
B58	HLA-B*15:67	1054	1062	QSAPHGVVF	0.8856
B58	HLA-B*15:67	1086	1095	KAHFPREGVF	0.5268
B58	HLA-B*15:67	1093	1102	GVFVSNGTHW	0.5731
B58	HLA-B*15:95	34	43	RGVYYPDKVF	0.5969
B58	HLA-B*15:95	50	58	STQDLFLPF	0.5282
B58	HLA-B*15:95	158	168	RVYSSANNCTF	0.5058

B58	HLA-B*15:95	160	168	YSSANNCTF	0.7058
B58	HLA-B*15:95	258	266	WTAGAAAYY	0.6382
B58	HLA-B*15:95	267	275	VGYLQPRTF	0.6579
B58	HLA-B*15:95	304	312	KSFTVEKGI	0.6907
B58	HLA-B*15:95	304	313	KSFTVEKGIY	0.5666
B58	HLA-B*15:95	310	318	KGIYQTSNF	0.5569
B58	HLA-B*15:95	344	353	ATRFASVYAW	0.5798
B58	HLA-B*15:95	372	380	ASFSTFKCY	0.6011
B58	HLA-B*15:95	392	400	FTNVYADSF	0.5426
B58	HLA-B*15:95	590	598	CSFGGVSVI	0.5003
B58	HLA-B*15:95	604	612	TSNQVAVLY	0.7366
B58	HLA-B*15:95	625	633	HADQLTPTW	0.7356
B58	HLA-B*15:95	634	643	RVYSTGSNVF	0.7402
B58	HLA-B*15:95	685	693	RSVASQSII	0.7531
B58	HLA-B*15:95	687	695	VASQSIIAY	0.7264
B58	HLA-B*15:95	710	718	NSIAIPTNF	0.7623
B58	HLA-B*15:95	712	720	IAIPTNFTI	0.8481
B58	HLA-B*15:95	718	726	FTISVTTEI	0.5787
B58	HLA-B*15:95	733	741	KTSVDCTMY	0.5963
B58	HLA-B*15:95	814	823	KRSFIEDLLF	0.6782
B58	HLA-B*15:95	815	823	RSFIEDLLF	0.9594
B58	HLA-B*15:95	825	833	KVTLADAGF	0.5677
B58	HLA-B*15:95	878	886	LAGTITSGW	0.5751
B58	HLA-B*15:95	879	888	AGTITSGWTF	0.5289
B58	HLA-B*15:95	880	888	GTITSGWTF	0.8368
B58	HLA-B*15:95	898	906	FAMQMAYRF	0.5752
B58	HLA-B*15:95	923	931	IANQFNSAI	0.5088
B58	HLA-B*15:95	1005	1013	QTYVTQQLI	0.6101
B58	HLA-B*15:95	1054	1062	QSAPHGVVF	0.8744
B58	HLA-B*15:95	1086	1095	KAHFPREGVF	0.5522
B58	HLA-B*15:95	1093	1102	GVFVSNGTHW	0.6012
B58	HLA-B*57:01	97	104	KSNIIRGW	0.8688
B58	HLA-B*57:01	304	312	KSFTVEKGI	0.5385
B58	HLA-B*57:01	344	353	ATRFASVYAW	0.7634
B58	HLA-B*57:01	622	633	VAIHADQLTPTW	0.5506
B58	HLA-B*57:01	625	633	HADQLTPTW	0.8783
B58	HLA-B*57:01	712	720	IAIPTNFTI	0.5812
B58	HLA-B*57:01	815	823	RSFIEDLLF	0.868
B58	HLA-B*57:01	878	886	LAGTITSGW	0.7806
B58	HLA-B*57:01	880	888	GTITSGWTF	0.7834
B58	HLA-B*57:01	1093	1102	GVFVSNGTHW	0.7168
B58	HLA-B*57:02	160	168	YSSANNCTF	0.5613
B58	HLA-B*57:02	249	258	LTPGDSSSGW	0.5051
B58	HLA-B*57:02	344	353	ATRFASVYAW	0.6112
B58	HLA-B*57:02	622	633	VAIHADQLTPTW	0.6009
B58	HLA-B*57:02	624	633	IHADQLTPTW	0.6898
B58	HLA-B*57:02	625	633	HADQLTPTW	0.9339
B58	HLA-B*57:02	712	720	IAIPTNFTI	0.6703
B58	HLA-B*57:02	815	823	RSFIEDLLF	0.8365
B58	HLA-B*57:02	878	886	LAGTITSGW	0.7731

B58	HLA-B*57:02	880	888	GTITSGWTF	0.7332
B58	HLA-B*57:02	1054	1062	QSAPHGVVF	0.8599
B58	HLA-B*57:03	97	104	KSNIIRGW	0.5712
B58	HLA-B*57:03	160	168	YSSANNCTF	0.52
B58	HLA-B*57:03	304	312	KSFTVEKGI	0.5693
B58	HLA-B*57:03	344	353	ATRFASVYAW	0.6947
B58	HLA-B*57:03	622	633	VAIHADQLTPTW	0.6476
B58	HLA-B*57:03	624	633	IHADQLTPTW	0.6328
B58	HLA-B*57:03	625	633	HADQLTPTW	0.9284
B58	HLA-B*57:03	634	643	RVYSTGSNVF	0.5104
B58	HLA-B*57:03	685	693	RSVASQSII	0.56
B58	HLA-B*57:03	710	718	NSIAIPTNF	0.7756
B58	HLA-B*57:03	712	720	IAIPTNFTI	0.8166
B58	HLA-B*57:03	814	823	KRSFIEDLLF	0.5259
B58	HLA-B*57:03	815	823	RSFIEDLLF	0.9134
B58	HLA-B*57:03	825	833	KVTLADAGF	0.5236
B58	HLA-B*57:03	878	886	LAGTITSGW	0.816
B58	HLA-B*57:03	880	888	GTITSGWTF	0.8295
B58	HLA-B*57:03	898	906	FAMQMAYRF	0.5657
B58	HLA-B*57:03	1054	1062	QSAPHGVVF	0.8674
B58	HLA-B*57:03	1093	1102	GVFVSNGTHW	0.6139
B58	HLA-B*57:07	344	353	ATRFASVYAW	0.5647
B58	HLA-B*57:07	625	633	HADQLTPTW	0.8776
B58	HLA-B*57:07	712	720	IAIPTNFTI	0.6961
B58	HLA-B*57:07	815	823	RSFIEDLLF	0.7639
B58	HLA-B*57:07	878	886	LAGTITSGW	0.6899
B58	HLA-B*57:07	880	888	GTITSGWTF	0.6913
B58	HLA-B*57:07	898	906	FAMQMAYRF	0.5331
B58	HLA-B*57:08	97	104	KSNIIRGW	0.8688
B58	HLA-B*57:08	304	312	KSFTVEKGI	0.5385
B58	HLA-B*57:08	344	353	ATRFASVYAW	0.7634
B58	HLA-B*57:08	622	633	VAIHADQLTPTW	0.5506
B58	HLA-B*57:08	625	633	HADQLTPTW	0.8783
B58	HLA-B*57:08	712	720	IAIPTNFTI	0.5812
B58	HLA-B*57:08	815	823	RSFIEDLLF	0.868
B58	HLA-B*57:08	878	886	LAGTITSGW	0.7806
B58	HLA-B*57:08	880	888	GTITSGWTF	0.7834
B58	HLA-B*57:08	1093	1102	GVFVSNGTHW	0.7168
B58	HLA-B*57:09	344	353	ATRFASVYAW	0.5174
B58	HLA-B*57:09	634	643	RVYSTGSNVF	0.591
B58	HLA-B*57:09	712	720	IAIPTNFTI	0.5816
B58	HLA-B*57:09	815	823	RSFIEDLLF	0.7555
B58	HLA-B*57:09	878	886	LAGTITSGW	0.5423
B58	HLA-B*57:09	880	888	GTITSGWTF	0.7045
B58	HLA-B*57:09	1054	1062	QSAPHGVVF	0.7826
B58	HLA-B*57:09	1086	1095	KAHFPREGVF	0.5053
B58	HLA-B*58:01	97	104	KSNIIRGW	0.6994
B58	HLA-B*58:01	160	168	YSSANNCTF	0.5597
B58	HLA-B*58:01	344	353	ATRFASVYAW	0.7778
B58	HLA-B*58:01	604	612	TSNQVAVLY	0.7554

B58	HLA-B*58:01	622	633	VAIHADQLTPTW	0.7607
B58	HLA-B*58:01	624	633	IHADQLTPTW	0.7759
B58	HLA-B*58:01	625	633	HADQLTPTW	0.9737
B58	HLA-B*58:01	687	695	VASQSIIAY	0.6802
B58	HLA-B*58:01	710	718	NSIAIPTNF	0.8133
B58	HLA-B*58:01	712	720	IAIPTNFTI	0.8079
B58	HLA-B*58:01	814	823	KRSFIEDLLF	0.5656
B58	HLA-B*58:01	815	823	RSFIEDLLF	0.9373
B58	HLA-B*58:01	878	886	LAGTITSGW	0.8666
B58	HLA-B*58:01	880	888	GTITSGWTF	0.879
B58	HLA-B*58:01	898	906	FAMQMAYRF	0.6403
B58	HLA-B*58:01	1054	1062	QSAPHGVVF	0.8091
B58	HLA-B*58:01	1093	1102	GVFVSNGTHW	0.7252
B58	HLA-B*58:02	815	823	RSFIEDLLF	0.6532
B58	HLA-B*58:04	56	64	LPFFSNVTW	0.5448
B58	HLA-B*58:04	97	104	KSNIIRGW	0.6084
B58	HLA-B*58:04	160	168	YSSANNCTF	0.5817
B58	HLA-B*58:04	344	353	ATRFASVYAW	0.7355
B58	HLA-B*58:04	604	612	TSNQVAVLY	0.7164
B58	HLA-B*58:04	622	633	VAIHADQLTPTW	0.7105
B58	HLA-B*58:04	624	633	IHADQLTPTW	0.7479
B58	HLA-B*58:04	625	633	HADQLTPTW	0.9681
B58	HLA-B*58:04	687	695	VASQSIIAY	0.648
B58	HLA-B*58:04	710	718	NSIAIPTNF	0.7658
B58	HLA-B*58:04	712	720	IAIPTNFTI	0.7552
B58	HLA-B*58:04	814	823	KRSFIEDLLF	0.5733
B58	HLA-B*58:04	815	823	RSFIEDLLF	0.9452
B58	HLA-B*58:04	878	886	LAGTITSGW	0.801
B58	HLA-B*58:04	880	888	GTITSGWTF	0.8598
B58	HLA-B*58:04	892	900	AALQIPFAM	0.5093
B58	HLA-B*58:04	898	906	FAMQMAYRF	0.6552
B58	HLA-B*58:04	1054	1062	QSAPHGVVF	0.808
B58	HLA-B*58:04	1093	1102	GVFVSNGTHW	0.6243
B58	HLA-B*58:06	815	823	RSFIEDLLF	0.7373
B58	HLA-B*58:08	62	70	VTWFHAIHV	0.5966
B58	HLA-B*58:08	304	312	KSFTVEKGI	0.7929
B58	HLA-B*58:08	590	598	CSFGGVSVI	0.664
B58	HLA-B*58:08	685	692	RSVASQSI	0.5963
B58	HLA-B*58:08	685	693	RSVASQSII	0.7492
B58	HLA-B*58:08	697	705	MSLGAENSV	0.5739
B58	HLA-B*58:08	711	720	SIAIPTNFTI	0.6417
B58	HLA-B*58:08	712	720	IAIPTNFTI	0.9331
B58	HLA-B*58:08	718	726	FTISVTTEI	0.7015
B58	HLA-B*58:08	815	823	RSFIEDLLF	0.7859
B58	HLA-B*58:08	923	931	IANQFNSAI	0.6908
B58	HLA-B*58:08	1005	1013	QTYVTQQLI	0.8005
B58	HLA-B*58:09	56	64	LPFFSNVTW	0.6098
B58	HLA-B*58:09	97	104	KSNIIRGW	0.7131
B58	HLA-B*58:09	160	168	YSSANNCTF	0.5758
B58	HLA-B*58:09	258	266	WTAGAAAYY	0.5718

B58	HLA-B*58:09	304	313	KSFTVEKGIY	0.5095
B58	HLA-B*58:09	344	353	ATRFASVYAW	0.7902
B58	HLA-B*58:09	604	612	TSNQVAVLY	0.8181
B58	HLA-B*58:09	622	633	VAIHADQLTPTW	0.8017
B58	HLA-B*58:09	623	633	AIHADQLTPTW	0.5232
B58	HLA-B*58:09	624	633	IHADQLTPTW	0.8068
B58	HLA-B*58:09	625	633	HADQLTPTW	0.969
B58	HLA-B*58:09	687	695	VASQSIIAY	0.7266
B58	HLA-B*58:09	710	718	NSIAIPTNF	0.8466
B58	HLA-B*58:09	712	720	IAIPTNFTI	0.8112
B58	HLA-B*58:09	733	741	KTSVDCTMY	0.5183
B58	HLA-B*58:09	814	823	KRSFIEDLLF	0.5561
B58	HLA-B*58:09	815	823	RSFIEDLLF	0.8962
B58	HLA-B*58:09	878	886	LAGTITSGW	0.8826
B58	HLA-B*58:09	879	888	AGTITSGWTF	0.5228
B58	HLA-B*58:09	880	888	GTITSGWTF	0.86
B58	HLA-B*58:09	892	900	AALQIPFAM	0.5678
B58	HLA-B*58:09	898	906	FAMQMAYRF	0.7372
B58	HLA-B*58:09	1054	1062	QSAPHGVVF	0.8091
B58	HLA-B*58:09	1093	1102	GVFVSNGTHW	0.7325
B58	HLA-B*58:11	97	104	KSNIIRGW	0.6994
B58	HLA-B*58:11	160	168	YSSANNCTF	0.5597
B58	HLA-B*58:11	344	353	ATRFASVYAW	0.7778
B58	HLA-B*58:11	604	612	TSNQVAVLY	0.7554
B58	HLA-B*58:11	622	633	VAIHADQLTPTW	0.7607
B58	HLA-B*58:11	624	633	IHADQLTPTW	0.7759
B58	HLA-B*58:11	625	633	HADQLTPTW	0.9737
B58	HLA-B*58:11	687	695	VASQSIIAY	0.6802
B58	HLA-B*58:11	710	718	NSIAIPTNF	0.8133
B58	HLA-B*58:11	712	720	IAIPTNFTI	0.8079
B58	HLA-B*58:11	814	823	KRSFIEDLLF	0.5656
B58	HLA-B*58:11	815	823	RSFIEDLLF	0.9373
B58	HLA-B*58:11	878	886	LAGTITSGW	0.8666
B58	HLA-B*58:11	880	888	GTITSGWTF	0.879
B58	HLA-B*58:11	898	906	FAMQMAYRF	0.6403
B58	HLA-B*58:11	1054	1062	QSAPHGVVF	0.8091
B58	HLA-B*58:11	1093	1102	GVFVSNGTHW	0.7252
B62	HLA-B*15:01	35	43	GVYYPDKVF	0.677
B62	HLA-B*15:01	47	55	VLHSTQDLF	0.5635
B62	HLA-B*15:01	192	200	FVFKNIDGY	0.678
B62	HLA-B*15:01	212	220	LVRDLPQGF	0.7136
B62	HLA-B*15:01	240	248	TLLALHRSY	0.6805
B62	HLA-B*15:01	258	266	WTAGAAAYY	0.5072
B62	HLA-B*15:01	261	269	GAAAYYVGY	0.52
B62	HLA-B*15:01	366	374	SVLYNSASF	0.5995
B62	HLA-B*15:01	413	421	GQTGKIADY	0.764
B62	HLA-B*15:01	497	505	FQPTNGVGY	0.7039
B62	HLA-B*15:01	628	636	QLTPTWRVY	0.6565
B62	HLA-B*15:01	634	643	RVYSTGSNVF	0.8491
B62	HLA-B*15:01	686	695	SVASQSIIAY	0.5924

B62	HLA-B*15:01	687	695	VASQSIIAY	0.7822
B62	HLA-B*15:01	698	707	SLGAENSVAY	0.6839
B62	HLA-B*15:01	880	888	GTITSGWTF	0.6048
B62	HLA-B*15:01	894	902	LQIPFAMQM	0.6403
B62	HLA-B*15:01	919	927	NQKLIANQF	0.7035
B62	HLA-B*15:01	962	970	LVKQLSSNF	0.7084
B62	HLA-B*15:01	1000	1007	RLQSLQTY	0.5767
B62	HLA-B*15:01	1054	1062	QSAPHGVVF	0.7211
B62	HLA-B*15:01	1113	1121	QIITTDNTF	0.6229
B62	HLA-B*15:01	1264	1272	VLKGVKLHY	0.8811
B62	HLA-B*15:02	84	92	LPFNDGVYF	0.5012
B62	HLA-B*15:02	192	200	FVFKNIDGY	0.6067
B62	HLA-B*15:02	212	220	LVRDLPQGF	0.5599
B62	HLA-B*15:02	240	248	TLLALHRSY	0.6225
B62	HLA-B*15:02	366	374	SVLYNSASF	0.593
B62	HLA-B*15:02	628	636	QLTPTWRVY	0.6554
B62	HLA-B*15:02	687	695	VASQSIIAY	0.8247
B62	HLA-B*15:02	698	707	SLGAENSVAY	0.593
B62	HLA-B*15:02	699	707	LGAENSVAY	0.5304
B62	HLA-B*15:02	896	904	IPFAMQMAY	0.6693
B62	HLA-B*15:02	1054	1062	QSAPHGVVF	0.678
B62	HLA-B*15:02	1113	1121	QIITTDNTF	0.6078
B62	HLA-B*15:05	192	200	FVFKNIDGY	0.568
B62	HLA-B*15:05	240	248	TLLALHRSY	0.5065
B62	HLA-B*15:05	413	421	GQTGKIADY	0.5265
B62	HLA-B*15:05	497	505	FQPTNGVGY	0.5981
B62	HLA-B*15:05	634	643	RVYSTGSNVF	0.5053
B62	HLA-B*15:05	687	695	VASQSIIAY	0.7516
B62	HLA-B*15:05	689	697	SQSIIAYTM	0.5439
B62	HLA-B*15:05	880	888	GTITSGWTF	0.603
B62	HLA-B*15:05	894	902	LQIPFAMQM	0.5925
B62	HLA-B*15:05	1054	1062	QSAPHGVVF	0.588
B62	HLA-B*15:05	1113	1121	QIITTDNTF	0.5456
B62	HLA-B*15:12	628	636	QLTPTWRVY	0.5076
B62	HLA-B*15:12	634	643	RVYSTGSNVF	0.5866
B62	HLA-B*15:12	687	695	VASQSIIAY	0.588
B62	HLA-B*15:12	1054	1062	QSAPHGVVF	0.5578
B62	HLA-B*15:14	634	643	RVYSTGSNVF	0.5315
B62	HLA-B*15:15	192	200	FVFKNIDGY	0.6152
B62	HLA-B*15:15	212	220	LVRDLPQGF	0.6015
B62	HLA-B*15:15	240	248	TLLALHRSY	0.5454
B62	HLA-B*15:15	366	374	SVLYNSASF	0.5295
B62	HLA-B*15:15	628	636	QLTPTWRVY	0.5711
B62	HLA-B*15:15	634	643	RVYSTGSNVF	0.5219
B62	HLA-B*15:15	686	695	SVASQSIIAY	0.5315
B62	HLA-B*15:15	687	695	VASQSIIAY	0.7424
B62	HLA-B*15:15	698	707	SLGAENSVAY	0.6339
B62	HLA-B*15:15	699	707	LGAENSVAY	0.5063
B62	HLA-B*15:15	919	927	NQKLIANQF	0.6734
B62	HLA-B*15:15	962	970	LVKQLSSNF	0.5773

B62	HLA-B*15:15	1054	1062	QSAPHGVVF	0.6835
B62	HLA-B*15:15	1113	1121	QIITTDNTF	0.6196
B62	HLA-B*15:19	628	636	QLTPTWRVY	0.5076
B62	HLA-B*15:19	634	643	RVYSTGSNVF	0.5866
B62	HLA-B*15:19	687	695	VASQSIIAY	0.588
B62	HLA-B*15:19	1054	1062	QSAPHGVVF	0.5578
B62	HLA-B*15:20	192	200	FVFKNIDGY	0.6015
B62	HLA-B*15:20	240	248	TLLALHRSY	0.5374
B62	HLA-B*15:20	497	505	FQPTNGVGY	0.5717
B62	HLA-B*15:20	628	636	QLTPTWRVY	0.5008
B62	HLA-B*15:20	687	695	VASQSIIAY	0.764
B62	HLA-B*15:20	689	697	SQSIIAYTM	0.525
B62	HLA-B*15:20	698	707	SLGAENSVAY	0.5119
B62	HLA-B*15:20	880	888	GTITSGWTF	0.5777
B62	HLA-B*15:20	894	902	LQIPFAMQM	0.5729
B62	HLA-B*15:20	1054	1062	QSAPHGVVF	0.5705
B62	HLA-B*15:20	1113	1121	QIITTDNTF	0.55
B62	HLA-B*15:25	30	38	NSFTRGVYY	0.5102
B62	HLA-B*15:25	35	43	GVYYPDKVF	0.7166
B62	HLA-B*15:25	47	55	VLHSTQDLF	0.5497
B62	HLA-B*15:25	152	160	WMESEFRVY	0.5406
B62	HLA-B*15:25	192	200	FVFKNIDGY	0.6774
B62	HLA-B*15:25	212	220	LVRDLPQGF	0.6749
B62	HLA-B*15:25	240	248	TLLALHRSY	0.7448
B62	HLA-B*15:25	261	269	GAAAYYVGY	0.5614
B62	HLA-B*15:25	366	374	SVLYNSASF	0.6588
B62	HLA-B*15:25	413	421	GQTGKIADY	0.6454
B62	HLA-B*15:25	497	505	FQPTNGVGY	0.6173
B62	HLA-B*15:25	628	636	QLTPTWRVY	0.7063
B62	HLA-B*15:25	634	643	RVYSTGSNVF	0.8174
B62	HLA-B*15:25	686	695	SVASQSIIAY	0.5343
B62	HLA-B*15:25	687	695	VASQSIIAY	0.8425
B62	HLA-B*15:25	698	707	SLGAENSVAY	0.6499
B62	HLA-B*15:25	699	707	LGAENSVAY	0.5125
B62	HLA-B*15:25	880	888	GTITSGWTF	0.6109
B62	HLA-B*15:25	894	902	LQIPFAMQM	0.6635
B62	HLA-B*15:25	962	970	LVKQLSSNF	0.5897
B62	HLA-B*15:25	1000	1007	RLQSLQTY	0.5751
B62	HLA-B*15:25	1054	1062	QSAPHGVVF	0.6973
B62	HLA-B*15:25	1113	1121	QIITTDNTF	0.6015
B62	HLA-B*15:25	1264	1272	VLKGVKLHY	0.8551
B62	HLA-B*15:28	35	43	GVYYPDKVF	0.677
B62	HLA-B*15:28	47	55	VLHSTQDLF	0.5635
B62	HLA-B*15:28	192	200	FVFKNIDGY	0.678
B62	HLA-B*15:28	212	220	LVRDLPQGF	0.7136
B62	HLA-B*15:28	240	248	TLLALHRSY	0.6805
B62	HLA-B*15:28	258	266	WTAGAAAYY	0.5072
B62	HLA-B*15:28	261	269	GAAAYYVGY	0.52
B62	HLA-B*15:28	366	374	SVLYNSASF	0.5995
B62	HLA-B*15:28	413	421	GQTGKIADY	0.764

B62	HLA-B*15:28	497	505	FQPTNGVGY	0.7039
B62	HLA-B*15:28	628	636	QLTPTWRVY	0.6565
B62	HLA-B*15:28	634	643	RVYSTGSNVF	0.8491
B62	HLA-B*15:28	686	695	SVASQSIIAY	0.5924
B62	HLA-B*15:28	687	695	VASQSIIAY	0.7822
B62	HLA-B*15:28	698	707	SLGAENSVAY	0.6839
B62	HLA-B*15:28	880	888	GTITSGWTF	0.6048
B62	HLA-B*15:28	894	902	LQIPFAMQM	0.6403
B62	HLA-B*15:28	919	927	NQKLIANQF	0.7035
B62	HLA-B*15:28	962	970	LVKQLSSNF	0.7084
B62	HLA-B*15:28	1000	1007	RLQSLQTY	0.5767
B62	HLA-B*15:28	1054	1062	QSAPHGVVF	0.7211
B62	HLA-B*15:28	1113	1121	QIITTDNTF	0.6229
B62	HLA-B*15:28	1264	1272	VLKGVKLHY	0.8811
B62	HLA-B*15:31	84	92	LPFNDGVYF	0.5604
B62	HLA-B*15:31	192	200	FVFKNIDGY	0.5375
B62	HLA-B*15:31	687	695	VASQSIIAY	0.7264
B62	HLA-B*15:31	896	904	IPFAMQMAY	0.5929
B62	HLA-B*15:31	1054	1062	QSAPHGVVF	0.5241
B62	HLA-B*15:31	1113	1121	QIITTDNTF	0.5135
B62	HLA-B*15:33	35	43	GVYYPDKVF	0.677
B62	HLA-B*15:33	47	55	VLHSTQDLF	0.5635
B62	HLA-B*15:33	192	200	FVFKNIDGY	0.678
B62	HLA-B*15:33	212	220	LVRDLPQGF	0.7136
B62	HLA-B*15:33	240	248	TLLALHRSY	0.6805
B62	HLA-B*15:33	258	266	WTAGAAAYY	0.5072
B62	HLA-B*15:33	261	269	GAAAYYVGY	0.52
B62	HLA-B*15:33	366	374	SVLYNSASF	0.5995
B62	HLA-B*15:33	413	421	GQTGKIADY	0.764
B62	HLA-B*15:33	497	505	FQPTNGVGY	0.7039
B62	HLA-B*15:33	628	636	QLTPTWRVY	0.6565
B62	HLA-B*15:33	634	643	RVYSTGSNVF	0.8491
B62	HLA-B*15:33	686	695	SVASQSIIAY	0.5924
B62	HLA-B*15:33	687	695	VASQSIIAY	0.7822
B62	HLA-B*15:33	698	707	SLGAENSVAY	0.6839
B62	HLA-B*15:33	880	888	GTITSGWTF	0.6048
B62	HLA-B*15:33	894	902	LQIPFAMQM	0.6403
B62	HLA-B*15:33	919	927	NQKLIANQF	0.7035
B62	HLA-B*15:33	962	970	LVKQLSSNF	0.7084
B62	HLA-B*15:33	1000	1007	RLQSLQTY	0.5767
B62	HLA-B*15:33	1054	1062	QSAPHGVVF	0.7211
B62	HLA-B*15:33	1113	1121	QIITTDNTF	0.6229
B62	HLA-B*15:33	1264	1272	VLKGVKLHY	0.8811
B62	HLA-B*15:34	35	43	GVYYPDKVF	0.677
B62	HLA-B*15:34	47	55	VLHSTQDLF	0.5635
B62	HLA-B*15:34	192	200	FVFKNIDGY	0.678
B62	HLA-B*15:34	212	220	LVRDLPQGF	0.7136
B62	HLA-B*15:34	240	248	TLLALHRSY	0.6805
B62	HLA-B*15:34	258	266	WTAGAAAYY	0.5072
B62	HLA-B*15:34	261	269	GAAAYYVGY	0.52

B62	HLA-B*15:34	366	374	SVLYNSASF	0.5995
B62	HLA-B*15:34	413	421	GQTGKIADY	0.764
B62	HLA-B*15:34	497	505	FQPTNGVGY	0.7039
B62	HLA-B*15:34	628	636	QLTPTWRVY	0.6565
B62	HLA-B*15:34	634	643	RVYSTGSNVF	0.8491
B62	HLA-B*15:34	686	695	SVASQSIIAY	0.5924
B62	HLA-B*15:34	687	695	VASQSIIAY	0.7822
B62	HLA-B*15:34	698	707	SLGAENSVAY	0.6839
B62	HLA-B*15:34	880	888	GTITSGWTF	0.6048
B62	HLA-B*15:34	894	902	LQIPFAMQM	0.6403
B62	HLA-B*15:34	919	927	NQKLIANQF	0.7035
B62	HLA-B*15:34	962	970	LVKQLSSNF	0.7084
B62	HLA-B*15:34	1000	1007	RLQSLQTY	0.5767
B62	HLA-B*15:34	1054	1062	QSAPHGVVF	0.7211
B62	HLA-B*15:34	1113	1121	QIITTDNTF	0.6229
B62	HLA-B*15:34	1264	1272	VLKGVKLHY	0.8811
B62	HLA-B*15:38	30	38	NSFTRGVYY	0.5586
B62	HLA-B*15:38	152	160	WMESEFRVY	0.5815
B62	HLA-B*15:38	192	200	FVFKNIDGY	0.6613
B62	HLA-B*15:38	212	220	LVRDLPQGF	0.6736
B62	HLA-B*15:38	240	248	TLLALHRSY	0.7304
B62	HLA-B*15:38	261	269	GAAAYYVGY	0.5782
B62	HLA-B*15:38	366	374	SVLYNSASF	0.5604
B62	HLA-B*15:38	413	421	GQTGKIADY	0.7293
B62	HLA-B*15:38	464	473	FERDISTEIY	0.5752
B62	HLA-B*15:38	497	505	FQPTNGVGY	0.705
B62	HLA-B*15:38	628	636	QLTPTWRVY	0.676
B62	HLA-B*15:38	634	643	RVYSTGSNVF	0.7511
B62	HLA-B*15:38	686	695	SVASQSIIAY	0.5671
B62	HLA-B*15:38	687	695	VASQSIIAY	0.7799
B62	HLA-B*15:38	689	697	SQSIIAYTM	0.5356
B62	HLA-B*15:38	698	707	SLGAENSVAY	0.6347
B62	HLA-B*15:38	699	707	LGAENSVAY	0.5322
B62	HLA-B*15:38	880	888	GTITSGWTF	0.5811
B62	HLA-B*15:38	894	902	LQIPFAMQM	0.6945
B62	HLA-B*15:38	919	927	NQKLIANQF	0.8136
B62	HLA-B*15:38	962	970	LVKQLSSNF	0.7271
B62	HLA-B*15:38	1000	1007	RLQSLQTY	0.5657
B62	HLA-B*15:38	1054	1062	QSAPHGVVF	0.711
B62	HLA-B*15:38	1113	1121	QIITTDNTF	0.5803
B62	HLA-B*15:38	1264	1272	VLKGVKLHY	0.8714
B62	HLA-B*15:39	35	43	GVYYPDKVF	0.7375
B62	HLA-B*15:39	47	55	VLHSTQDLF	0.5828
B62	HLA-B*15:39	83	92	VLPFNDGVYF	0.5256
B62	HLA-B*15:39	152	160	WMESEFRVY	0.5416
B62	HLA-B*15:39	192	200	FVFKNIDGY	0.6491
B62	HLA-B*15:39	212	220	LVRDLPQGF	0.6773
B62	HLA-B*15:39	240	248	TLLALHRSY	0.7211
B62	HLA-B*15:39	261	269	GAAAYYVGY	0.5543
B62	HLA-B*15:39	366	374	SVLYNSASF	0.6531

B62	HLA-B*15:39	413	421	GQTGKIADY	0.6727
B62	HLA-B*15:39	497	505	FQPTNGVGY	0.6539
B62	HLA-B*15:39	628	636	QLTPTWRVY	0.6869
B62	HLA-B*15:39	634	643	RVYSTGSNVF	0.8327
B62	HLA-B*15:39	686	695	SVASQSIIAY	0.5034
B62	HLA-B*15:39	687	695	VASQSIIAY	0.8337
B62	HLA-B*15:39	689	697	SQSIIAYTM	0.5045
B62	HLA-B*15:39	698	707	SLGAENSVAY	0.6247
B62	HLA-B*15:39	699	707	LGAENSVAY	0.5064
B62	HLA-B*15:39	815	823	RSFIEDLLF	0.5266
B62	HLA-B*15:39	880	888	GTITSGWTF	0.6421
B62	HLA-B*15:39	894	902	LQIPFAMQM	0.6884
B62	HLA-B*15:39	919	927	NQKLIANQF	0.5508
B62	HLA-B*15:39	962	970	LVKQLSSNF	0.5971
B62	HLA-B*15:39	1000	1007	RLQSLQTY	0.582
B62	HLA-B*15:39	1054	1062	QSAPHGVVF	0.7223
B62	HLA-B*15:39	1113	1121	QIITTDNTF	0.6116
B62	HLA-B*15:39	1264	1272	VLKGVKLHY	0.8466
B62	HLA-B*15:40	634	643	RVYSTGSNVF	0.6651
B62	HLA-B*15:40	687	695	VASQSIIAY	0.5923
B62	HLA-B*15:40	894	902	LQIPFAMQM	0.564
B62	HLA-B*15:40	1264	1272	VLKGVKLHY	0.65
B62	HLA-B*15:50	192	200	FVFKNIDGY	0.5522
B62	HLA-B*15:50	212	220	LVRDLPQGF	0.5998
B62	HLA-B*15:50	240	248	TLLALHRSY	0.595
B62	HLA-B*15:50	413	421	GQTGKIADY	0.6557
B62	HLA-B*15:50	628	636	QLTPTWRVY	0.567
B62	HLA-B*15:50	634	643	RVYSTGSNVF	0.7616
B62	HLA-B*15:50	687	695	VASQSIIAY	0.7011
B62	HLA-B*15:50	698	707	SLGAENSVAY	0.5791
B62	HLA-B*15:50	880	888	GTITSGWTF	0.5108
B62	HLA-B*15:50	894	902	LQIPFAMQM	0.5936
B62	HLA-B*15:50	919	927	NQKLIANQF	0.635
B62	HLA-B*15:50	962	970	LVKQLSSNF	0.5684
B62	HLA-B*15:50	1000	1007	RLQSLQTY	0.504
B62	HLA-B*15:50	1054	1062	QSAPHGVVF	0.6031
B62	HLA-B*15:50	1264	1272	VLKGVKLHY	0.8458
B62	HLA-B*15:60	35	43	GVYYPDKVF	0.677
B62	HLA-B*15:60	47	55	VLHSTQDLF	0.5635
B62	HLA-B*15:60	192	200	FVFKNIDGY	0.678
B62	HLA-B*15:60	212	220	LVRDLPQGF	0.7136
B62	HLA-B*15:60	240	248	TLLALHRSY	0.6805
B62	HLA-B*15:60	258	266	WTAGAAAYY	0.5072
B62	HLA-B*15:60	261	269	GAAAYYVGY	0.52
B62	HLA-B*15:60	366	374	SVLYNSASF	0.5995
B62	HLA-B*15:60	413	421	GQTGKIADY	0.764
B62	HLA-B*15:60	497	505	FQPTNGVGY	0.7039
B62	HLA-B*15:60	628	636	QLTPTWRVY	0.6565
B62	HLA-B*15:60	634	643	RVYSTGSNVF	0.8491
B62	HLA-B*15:60	686	695	SVASQSIIAY	0.5924

B62	HLA-B*15:60	687	695	VASQSIIAY	0.7822
B62	HLA-B*15:60	698	707	SLGAENSVAY	0.6839
B62	HLA-B*15:60	880	888	GTITSGWTF	0.6048
B62	HLA-B*15:60	894	902	LQIPFAMQM	0.6403
B62	HLA-B*15:60	919	927	NQKLIANQF	0.7035
B62	HLA-B*15:60	962	970	LVKQLSSNF	0.7084
B62	HLA-B*15:60	1000	1007	RLQSLQTY	0.5767
B62	HLA-B*15:60	1054	1062	QSAPHGVVF	0.7211
B62	HLA-B*15:60	1113	1121	QIITTDNTF	0.6229
B62	HLA-B*15:60	1264	1272	VLKGVKLHY	0.8811
B62	HLA-B*15:65	35	43	GVYYPDKVF	0.5822
B62	HLA-B*15:65	47	55	VLHSTQDLF	0.6073
B62	HLA-B*15:65	152	160	WMESEFRVY	0.5864
B62	HLA-B*15:65	192	200	FVFKNIDGY	0.5038
B62	HLA-B*15:65	212	220	LVRDLPQGF	0.5359
B62	HLA-B*15:65	240	248	TLLALHRSY	0.6159
B62	HLA-B*15:65	366	374	SVLYNSASF	0.6187
B62	HLA-B*15:65	584	592	ILDITPCSF	0.5073
B62	HLA-B*15:65	628	636	QLTPTWRVY	0.6602
B62	HLA-B*15:65	634	643	RVYSTGSNVF	0.7599
B62	HLA-B*15:65	686	695	SVASQSIIAY	0.5527
B62	HLA-B*15:65	687	695	VASQSIIAY	0.792
B62	HLA-B*15:65	698	707	SLGAENSVAY	0.6847
B62	HLA-B*15:65	880	888	GTITSGWTF	0.5709
B62	HLA-B*15:65	1000	1007	RLQSLQTY	0.5594
B62	HLA-B*15:65	1054	1062	QSAPHGVVF	0.7159
B62	HLA-B*15:65	1113	1121	QIITTDNTF	0.5676
B62	HLA-B*15:65	1264	1272	VLKGVKLHY	0.7435
B62	HLA-B*15:70	192	200	FVFKNIDGY	0.5037
B62	HLA-B*15:70	212	220	LVRDLPQGF	0.5805
B62	HLA-B*15:70	413	421	GQTGKIADY	0.5593
B62	HLA-B*15:70	634	643	RVYSTGSNVF	0.6634
B62	HLA-B*15:70	687	695	VASQSIIAY	0.563
B62	HLA-B*15:70	962	970	LVKQLSSNF	0.5026
B62	HLA-B*15:70	1054	1062	QSAPHGVVF	0.5214
B62	HLA-B*15:70	1264	1272	VLKGVKLHY	0.7409
B62	HLA-B*15:75	35	43	GVYYPDKVF	0.6355
B62	HLA-B*15:75	47	55	VLHSTQDLF	0.524
B62	HLA-B*15:75	192	200	FVFKNIDGY	0.652
B62	HLA-B*15:75	212	220	LVRDLPQGF	0.6707
B62	HLA-B*15:75	240	248	TLLALHRSY	0.6492
B62	HLA-B*15:75	366	374	SVLYNSASF	0.5638
B62	HLA-B*15:75	413	421	GQTGKIADY	0.7207
B62	HLA-B*15:75	497	505	FQPTNGVGY	0.656
B62	HLA-B*15:75	628	636	QLTPTWRVY	0.6233
B62	HLA-B*15:75	634	643	RVYSTGSNVF	0.8275
B62	HLA-B*15:75	686	695	SVASQSIIAY	0.5578
B62	HLA-B*15:75	687	695	VASQSIIAY	0.7575
B62	HLA-B*15:75	698	707	SLGAENSVAY	0.6521
B62	HLA-B*15:75	880	888	GTITSGWTF	0.5821

B62	HLA-B*15:75	894	902	LQIPFAMQM	0.6094
B62	HLA-B*15:75	919	927	NQKLIANQF	0.6621
B62	HLA-B*15:75	962	970	LVKQLSSNF	0.6585
B62	HLA-B*15:75	1000	1007	RLQSLQTY	0.533
B62	HLA-B*15:75	1054	1062	QSAPHGVVF	0.6833
B62	HLA-B*15:75	1113	1121	QIITTDNTF	0.5867
B62	HLA-B*15:75	1264	1272	VLKGVKLHY	0.8622
B62	HLA-B*15:78	35	43	GVYYPDKVF	0.677
B62	HLA-B*15:78	47	55	VLHSTQDLF	0.5635
B62	HLA-B*15:78	192	200	FVFKNIDGY	0.678
B62	HLA-B*15:78	212	220	LVRDLPQGF	0.7136
B62	HLA-B*15:78	240	248	TLLALHRSY	0.6805
B62	HLA-B*15:78	258	266	WTAGAAAYY	0.5072
B62	HLA-B*15:78	261	269	GAAAYYVGY	0.52
B62	HLA-B*15:78	366	374	SVLYNSASF	0.5995
B62	HLA-B*15:78	413	421	GQTGKIADY	0.764
B62	HLA-B*15:78	497	505	FQPTNGVGY	0.7039
B62	HLA-B*15:78	628	636	QLTPTWRVY	0.6565
B62	HLA-B*15:78	634	643	RVYSTGSNVF	0.8491
B62	HLA-B*15:78	686	695	SVASQSIIAY	0.5924
B62	HLA-B*15:78	687	695	VASQSIIAY	0.7822
B62	HLA-B*15:78	698	707	SLGAENSVAY	0.6839
B62	HLA-B*15:78	880	888	GTITSGWTF	0.6048
B62	HLA-B*15:78	894	902	LQIPFAMQM	0.6403
B62	HLA-B*15:78	919	927	NQKLIANQF	0.7035
B62	HLA-B*15:78	962	970	LVKQLSSNF	0.7084
B62	HLA-B*15:78	1000	1007	RLQSLQTY	0.5767
B62	HLA-B*15:78	1054	1062	QSAPHGVVF	0.7211
B62	HLA-B*15:78	1113	1121	QIITTDNTF	0.6229
B62	HLA-B*15:78	1264	1272	VLKGVKLHY	0.8811
B62	HLA-B*15:81	35	43	GVYYPDKVF	0.677
B62	HLA-B*15:81	47	55	VLHSTQDLF	0.5635
B62	HLA-B*15:81	192	200	FVFKNIDGY	0.678
B62	HLA-B*15:81	212	220	LVRDLPQGF	0.7136
B62	HLA-B*15:81	240	248	TLLALHRSY	0.6805
B62	HLA-B*15:81	258	266	WTAGAAAYY	0.5072
B62	HLA-B*15:81	261	269	GAAAYYVGY	0.52
B62	HLA-B*15:81	366	374	SVLYNSASF	0.5995
B62	HLA-B*15:81	413	421	GQTGKIADY	0.764
B62	HLA-B*15:81	497	505	FQPTNGVGY	0.7039
B62	HLA-B*15:81	628	636	QLTPTWRVY	0.6565
B62	HLA-B*15:81	634	643	RVYSTGSNVF	0.8491
B62	HLA-B*15:81	686	695	SVASQSIIAY	0.5924
B62	HLA-B*15:81	687	695	VASQSIIAY	0.7822
B62	HLA-B*15:81	698	707	SLGAENSVAY	0.6839
B62	HLA-B*15:81	880	888	GTITSGWTF	0.6048
B62	HLA-B*15:81	894	902	LQIPFAMQM	0.6403
B62	HLA-B*15:81	919	927	NQKLIANQF	0.7035
B62	HLA-B*15:81	962	970	LVKQLSSNF	0.7084
B62	HLA-B*15:81	1000	1007	RLQSLQTY	0.5767

B62	HLA-B*15:81	1054	1062	QSAPHGVVF	0.7211
B62	HLA-B*15:81	1113	1121	QIITTDNTF	0.6229
B62	HLA-B*15:81	1264	1272	VLKGVKLHY	0.8811
B62	HLA-B*15:82	35	43	GVYYPDKVF	0.677
B62	HLA-B*15:82	47	55	VLHSTQDLF	0.5635
B62	HLA-B*15:82	192	200	FVFKNIDGY	0.678
B62	HLA-B*15:82	212	220	LVRDLPQGF	0.7136
B62	HLA-B*15:82	240	248	TLLALHRSY	0.6805
B62	HLA-B*15:82	258	266	WTAGAAAYY	0.5072
B62	HLA-B*15:82	261	269	GAAAYYVGY	0.52
B62	HLA-B*15:82	366	374	SVLYNSASF	0.5995
B62	HLA-B*15:82	413	421	GQTGKIADY	0.764
B62	HLA-B*15:82	497	505	FQPTNGVGY	0.7039
B62	HLA-B*15:82	628	636	QLTPTWRVY	0.6565
B62	HLA-B*15:82	634	643	RVYSTGSNVF	0.8491
B62	HLA-B*15:82	686	695	SVASQSIIAY	0.5924
B62	HLA-B*15:82	687	695	VASQSIIAY	0.7822
B62	HLA-B*15:82	698	707	SLGAENSVAY	0.6839
B62	HLA-B*15:82	880	888	GTITSGWTF	0.6048
B62	HLA-B*15:82	894	902	LQIPFAMQM	0.6403
B62	HLA-B*15:82	919	927	NQKLIANQF	0.7035
B62	HLA-B*15:82	962	970	LVKQLSSNF	0.7084
B62	HLA-B*15:82	1000	1007	RLQSLQTY	0.5767
B62	HLA-B*15:82	1054	1062	QSAPHGVVF	0.7211
B62	HLA-B*15:82	1113	1121	QIITTDNTF	0.6229
B62	HLA-B*15:82	1264	1272	VLKGVKLHY	0.8811
B62	HLA-B*15:85	35	43	GVYYPDKVF	0.6525
B62	HLA-B*15:85	47	55	VLHSTQDLF	0.5234
B62	HLA-B*15:85	192	200	FVFKNIDGY	0.6835
B62	HLA-B*15:85	212	220	LVRDLPQGF	0.6903
B62	HLA-B*15:85	240	248	TLLALHRSY	0.6924
B62	HLA-B*15:85	258	266	WTAGAAAYY	0.5057
B62	HLA-B*15:85	261	269	GAAAYYVGY	0.5182
B62	HLA-B*15:85	366	374	SVLYNSASF	0.5905
B62	HLA-B*15:85	413	421	GQTGKIADY	0.7286
B62	HLA-B*15:85	497	505	FQPTNGVGY	0.6701
B62	HLA-B*15:85	628	636	QLTPTWRVY	0.6639
B62	HLA-B*15:85	634	643	RVYSTGSNVF	0.8172
B62	HLA-B*15:85	686	695	SVASQSIIAY	0.6057
B62	HLA-B*15:85	687	695	VASQSIIAY	0.7768
B62	HLA-B*15:85	698	707	SLGAENSVAY	0.6849
B62	HLA-B*15:85	880	888	GTITSGWTF	0.5734
B62	HLA-B*15:85	894	902	LQIPFAMQM	0.5976
B62	HLA-B*15:85	919	927	NQKLIANQF	0.6783
B62	HLA-B*15:85	962	970	LVKQLSSNF	0.6864
B62	HLA-B*15:85	1000	1007	RLQSLQTY	0.5531
B62	HLA-B*15:85	1054	1062	QSAPHGVVF	0.6958
B62	HLA-B*15:85	1113	1121	QIITTDNTF	0.608
B62	HLA-B*15:85	1264	1272	VLKGVKLHY	0.8756
B62	HLA-B*15:88	84	92	LPFNDGVYF	0.5189

B62	HLA-B*15:88	192	200	FVFKNIDGY	0.5876
B62	HLA-B*15:88	240	248	TLLALHRSY	0.5087
B62	HLA-B*15:88	628	636	QLTPTWRVY	0.53
B62	HLA-B*15:88	687	695	VASQSIIAY	0.7704
B62	HLA-B*15:88	698	707	SLGAENSVAY	0.5239
B62	HLA-B*15:88	699	707	LGAENSVAY	0.5042
B62	HLA-B*15:88	896	904	IPFAMQMAY	0.6381
B62	HLA-B*15:88	1054	1062	QSAPHGVVF	0.5587
B62	HLA-B*15:88	1113	1121	QIITTDNTF	0.5554
B62	HLA-B*15:92	35	43	GVYYPDKVF	0.677
B62	HLA-B*15:92	47	55	VLHSTQDLF	0.5635
B62	HLA-B*15:92	192	200	FVFKNIDGY	0.678
B62	HLA-B*15:92	212	220	LVRDLPQGF	0.7136
B62	HLA-B*15:92	240	248	TLLALHRSY	0.6805
B62	HLA-B*15:92	258	266	WTAGAAAYY	0.5072
B62	HLA-B*15:92	261	269	GAAAYYVGY	0.52
B62	HLA-B*15:92	366	374	SVLYNSASF	0.5995
B62	HLA-B*15:92	413	421	GQTGKIADY	0.764
B62	HLA-B*15:92	497	505	FQPTNGVGY	0.7039
B62	HLA-B*15:92	628	636	QLTPTWRVY	0.6565
B62	HLA-B*15:92	634	643	RVYSTGSNVF	0.8491
B62	HLA-B*15:92	686	695	SVASQSIIAY	0.5924
B62	HLA-B*15:92	687	695	VASQSIIAY	0.7822
B62	HLA-B*15:92	698	707	SLGAENSVAY	0.6839
B62	HLA-B*15:92	880	888	GTITSGWTF	0.6048
B62	HLA-B*15:92	894	902	LQIPFAMQM	0.6403
B62	HLA-B*15:92	919	927	NQKLIANQF	0.7035
B62	HLA-B*15:92	962	970	LVKQLSSNF	0.7084
B62	HLA-B*15:92	1000	1007	RLQSLQTY	0.5767
B62	HLA-B*15:92	1054	1062	QSAPHGVVF	0.7211
B62	HLA-B*15:92	1113	1121	QIITTDNTF	0.6229
B62	HLA-B*15:92	1264	1272	VLKGVKLHY	0.8811
B62	HLA-B*15:96	35	43	GVYYPDKVF	0.677
B62	HLA-B*15:96	47	55	VLHSTQDLF	0.5635
B62	HLA-B*15:96	192	200	FVFKNIDGY	0.678
B62	HLA-B*15:96	212	220	LVRDLPQGF	0.7136
B62	HLA-B*15:96	240	248	TLLALHRSY	0.6805
B62	HLA-B*15:96	258	266	WTAGAAAYY	0.5072
B62	HLA-B*15:96	261	269	GAAAYYVGY	0.52
B62	HLA-B*15:96	366	374	SVLYNSASF	0.5995
B62	HLA-B*15:96	413	421	GQTGKIADY	0.764
B62	HLA-B*15:96	497	505	FQPTNGVGY	0.7039
B62	HLA-B*15:96	628	636	QLTPTWRVY	0.6565
B62	HLA-B*15:96	634	643	RVYSTGSNVF	0.8491
B62	HLA-B*15:96	686	695	SVASQSIIAY	0.5924
B62	HLA-B*15:96	687	695	VASQSIIAY	0.7822
B62	HLA-B*15:96	698	707	SLGAENSVAY	0.6839
B62	HLA-B*15:96	880	888	GTITSGWTF	0.6048
B62	HLA-B*15:96	894	902	LQIPFAMQM	0.6403
B62	HLA-B*15:96	919	927	NQKLIANQF	0.7035

B62	HLA-B*15:96	962	970	LVKQLSSNF	0.7084
B62	HLA-B*15:96	1000	1007	RLQSLQTY	0.5767
B62	HLA-B*15:96	1054	1062	QSAPHGVVF	0.7211
B62	HLA-B*15:96	1113	1121	QIITTDNTF	0.6229
B62	HLA-B*15:96	1264	1272	VLKGVKLHY	0.8811
B62	HLA-B*15:97	35	43	GVYYPDKVF	0.677
B62	HLA-B*15:97	47	55	VLHSTQDLF	0.5635
B62	HLA-B*15:97	192	200	FVFKNIDGY	0.678
B62	HLA-B*15:97	212	220	LVRDLPQGF	0.7136
B62	HLA-B*15:97	240	248	TLLALHRSY	0.6805
B62	HLA-B*15:97	258	266	WTAGAAAYY	0.5072
B62	HLA-B*15:97	261	269	GAAAYYVGY	0.52
B62	HLA-B*15:97	366	374	SVLYNSASF	0.5995
B62	HLA-B*15:97	413	421	GQTGKIADY	0.764
B62	HLA-B*15:97	497	505	FQPTNGVGY	0.7039
B62	HLA-B*15:97	628	636	QLTPTWRVY	0.6565
B62	HLA-B*15:97	634	643	RVYSTGSNVF	0.8491
B62	HLA-B*15:97	686	695	SVASQSIIAY	0.5924
B62	HLA-B*15:97	687	695	VASQSIIAY	0.7822
B62	HLA-B*15:97	698	707	SLGAENSVAY	0.6839
B62	HLA-B*15:97	880	888	GTITSGWTF	0.6048
B62	HLA-B*15:97	894	902	LQIPFAMQM	0.6403
B62	HLA-B*15:97	919	927	NQKLIANQF	0.7035
B62	HLA-B*15:97	962	970	LVKQLSSNF	0.7084
B62	HLA-B*15:97	1000	1007	RLQSLQTY	0.5767
B62	HLA-B*15:97	1054	1062	QSAPHGVVF	0.7211
B62	HLA-B*15:97	1113	1121	QIITTDNTF	0.6229
B62	HLA-B*15:97	1264	1272	VLKGVKLHY	0.8811
B62	HLA-B*35:28	192	200	FVFKNIDGY	0.6199
B62	HLA-B*35:28	240	248	TLLALHRSY	0.5051
B62	HLA-B*35:28	261	269	GAAAYYVGY	0.5209
B62	HLA-B*35:28	339	347	GEVFNATRF	0.6317
B62	HLA-B*35:28	413	421	GQTGKIADY	0.5514
B62	HLA-B*35:28	443	451	SKVGGNYNY	0.6002
B62	HLA-B*35:28	464	473	FERDISTEIY	0.5471
B62	HLA-B*35:28	497	505	FQPTNGVGY	0.6463
B62	HLA-B*35:28	687	695	VASQSIIAY	0.7882
B62	HLA-B*35:28	689	697	SQSIIAYTM	0.6456
B62	HLA-B*35:28	699	707	LGAENSVAY	0.546
B62	HLA-B*35:28	815	823	RSFIEDLLF	0.5236
B62	HLA-B*35:28	880	888	GTITSGWTF	0.5709
B62	HLA-B*35:28	894	902	LQIPFAMQM	0.7173
B62	HLA-B*35:28	919	927	NQKLIANQF	0.5854
B62	HLA-B*35:28	1054	1062	QSAPHGVVF	0.6134
B62	HLA-B*35:28	1113	1121	QIITTDNTF	0.5096
B62	HLA-B*52:01	712	720	IAIPTNFTI	0.7144
B62	HLA-B*52:02	712	720	IAIPTNFTI	0.7268
B62	HLA-B*52:03	712	720	IAIPTNFTI	0.7225
B62	HLA-B*52:04	712	720	IAIPTNFTI	0.7144
B62	HLA-B*52:05	712	720	IAIPTNFTI	0.7144

B62	HLA-B*52:07	712	720	IAIPTNFTI	0.7144

Note: The epitopes conserved among sarbecoviruses are indicated in red
